# Stereodivergent Access to Aliphatic Nitro Compounds Bearing Multi‐Contiguous Stereocenters via Sequential Catalysis

**DOI:** 10.1002/advs.202519142

**Published:** 2026-02-03

**Authors:** Jia‐Hao Xie, Yi‐Ming Hou, Zhi‐Jie Wu, Chao Zheng, Shu‐Li You

**Affiliations:** ^1^ New Cornerstone Science Laboratory State Key Laboratory of Organometallic Chemistry Shanghai Institute of Organic Chemistry University of Chinese Academy of Sciences Chinese Academy of Sciences Shanghai China

**Keywords:** aliphatic nitro compounds, allylic substitution, conjugate addition, stereodivergent, transition metal‐catalyzed

## Abstract

Herein, we report a highly efficient synthesis of aliphatic nitro compounds bearing multi‐contiguous stereocenters in good yields (up to 72%) with excellent diastereo‐ and enantio‐selectivities (up to 12:1 dr, and >99% ee) by combining copper‐catalyzed asymmetric conjugate addition of dialkylzinc reagents to nitroalkenes with iridium‐catalyzed asymmetric allylic substitution reaction. Stereodivergent construction of nonadjacent stereocenters (1,3‐positions) has been achieved by combining two chiral catalysts with different enantiomers. By first introducing a chiral center at the β‐position of the nitro group, highly diastereoselective control has been achieved in iridium‐catalyzed allylic substitution reaction of prochiral nitro compounds.

## Graphical abstract

Herein, we report a highly efficient synthesis of aliphatic nitro compounds bearing multi‐contiguous stereocenters in good yields with excellent diastereo‐ and enantio‐selectivities by combining copper‐catalyzed asymmetric conjugate addition of dialkylzinc reagents to nitroalkenes with iridium‐catalyzed asymmetric allylic substitution reaction. Stereodivergent construction of nonadjacent stereocenters (1,3‐positions) has been achieved.

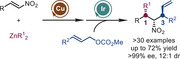

## 1 Introduction

**SCHEME 1:**
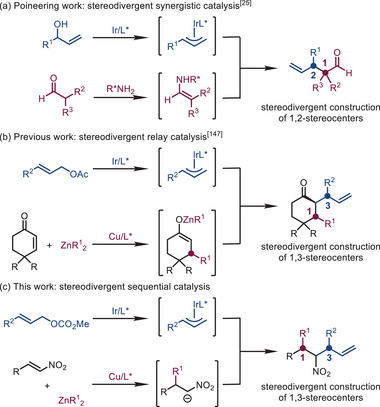
Stereodivergent dual catalysis.

Compounds with multiple stereocenters are widely distributed in pharmaceuticals and natural products. Given the fact that the biological activities of these molecules are frequently influenced by their relative and absolute configurations, it is meaningful to develop methods to construct different stereoisomers precisely [1, 2, 3]. Among the existing methods, stereodivergent dual catalysis, including synergistic catalysis, relay catalysis, and sequential catalysis, is one of the most effective methods to synthesize different stereoisomers from the same starting materials [4, 5, 6, 7, 8, 9, 10, 11, 12, 13, 14, 15, 16, 17, 18, 19, 20, 21, 22, 23, 24] (For Selected Reviews on Asymmetric Stereodivergent Catalysis). Since the pioneering work of Carreira and co‐workers in 2013 (Scheme 1a) [25], stereodivergent synthesis via synergistic catalysis has received much attention, and a series of elegant works have been reported [26, 27, 28, 29, 30, 31, 32, 33, 34, 35, 36, 37, 38, 39, 40, 41, 42, 43, 44, 45, 46, 47, 48, 49, 50] (For Selected Recent Examples on Synergistic Catalysis). On the contrary, relay catalysis and sequential catalysis where multiple stereocenters established in different stages remain relatively underdeveloped, mainly due to the stereocenter formed in the first stage might affect the asymmetric induction of the second stage, making it rather difficult to achieve high level of stereochemical control for the overall reaction [51, 52, 53, 54, 55, 56, 57, 58, 59, 60, 61, 62, 63, 64, 65, 66, 67, 68] (For Selected Recent Examples on Relay Catalysis and Sequential Catalysis). Each catalytic process proceeding in a highly stereospecific manner without stereochemical interfere would be the key to solve this problem.

Over the past two decades, iridium‐catalyzed asymmetric allylic substitution reaction has become one of the most efficient methods for the synthesis of various chiral molecules bearing an allylic stereocenter with high regio‐ and enantio‐selectivities [69, 70, 71, 72, 73, 74, 75, 76, 77, 78, 79, 80] (For Selected Reviews on Ir‐Catalyzed Asymmetric Allylic Substitution Reactions), [81, 82, 83, 84, 85, 86, 87, 88, 89, 90, 91, 92, 93, 94, 95, 96, 97, 98, 99, 100, 101, 102, 103, 104, 105, 106, 107, 108, 109] (For Selected Recent Examples on Ir‐Catalyzed Asymmetric Allylic Substitution Reactions). Although this reaction manifold has been widely employed in the stereodivergent dual catalysis, most examples were focused on the construction of two adjacent stereocenters [110, 111, 112, 113, 114, 115, 116, 117, 118, 119, 120, 121, 122, 123, 124, 125, 126, 127, 128, 129, 130, 131, 132, 133, 134, 135, 136, 137, 138]. The attempts for establishing non‐adjacent, or multi‐contiguous stereocenters, which are frequently encountered in numerous natural products and biologically active compounds [139, 140, 141, 142, 143, 144, 145] (For a Review and Selected Recent Examples on the Construction of Nonadjacent Stereocenters), still remain scarce [146, 147, 148].

Recently, we reported a stereodivergent synthesis of an array of chiral cyclohexanones via relay catalysis (Scheme 1b) [147]. Encouraged by these results, we speculated that aliphatic nitro compounds, [149, 150, 151, 152, 153, 154] (For Selected Examples of The Utility of Nitro Compounds), bearing multi‐contiguous stereocenters could be accessed in a stereodivergent manner by combining copper‐catalyzed asymmetric conjugate addition of dialkylzinc reagents to nitroalkenes with iridium‐catalyzed asymmetric allylic substitution reaction (Scheme 1c). Notably, iridium‐catalyzed allylic substitution reactions at the α‐position of the nitro group were rarely reported, and the control of diastereoselectivity in such reactions remains an unsolved problem [155, 156]. Herein, we report the results on addressing this issue by sequential asymmetric catalysis.

## 2 Results and Discussion

2.1

We initiated our studies by testing the model reaction of *β*‐nitrostyrene (**1a**, 0.1 mmol), diethylzinc (**2a**, 1.2 equiv), and cinnamyl methyl carbonate (**3a**, 1.2 equiv) (Table 1). Two chiral catalytic systems were employed, one consisting of Cu(OTf)_2_ (10 mol%) and dipeptide phosphine ligand (*S*,*S*)‐**L1** (10 mol%) for promoting the asymmetric conjugate addition of **1a** to **2a**, and the other consisting of [Ir(cod)Cl]_2_ (2 mol%) and Feringa ligand (*S,S,S_a_*)‐**L2** (4 mol%) for promoting the asymmetric allylic substitution with **3a**. Theoretically, the designed reaction can lead to a series of diastereoisomers due to the existence of three contiguous stereocenters. However, the two‐step reaction on the *β*‐ and *α*‐positions of **1a** made the Et and NO_2_ groups adopting the *syn* configuration predominately (for **4a** and **4a′**), while the amount of other diastereoisomers with the Et and NO_2_ in the *anti* configuration (like **4a″**) was insignificant. Compounds **4a** and **4a′** were further distinguished according to the relative configuration with the third stereocenter at the allylic position. ^1^H NMR spectrum of the crude reaction mixture indicated a 6.4:1 ratio between **4a** and **4a′**, and 64% yield of the former. The ee values of **4a** and **4a′** were determined as 98% and 26%, respectively (Entry 1, see the Supporting Information for more details). Next, the effects of various chiral phosphoramidites (**L3**–**L9**) as the ligand for the Ir‐catalyst were evaluated (Entries 2–8). It was found that (*S,S,S_a_*)‐**L9** derived from (*S*)‐VANOL gave similar outcomes compared with those obtained using (*S,S,S_a_*)‐**L2**, while the other ligands gave no better results. It is well known that different pre‐preparation methods might also influence the performance of the Ir‐catalysts [157, 158, 159, 160, 161] (For Using Cyclometallated Ir‐Complex As A Catalyst). Compared with the in situ preparation of the Ir‐catalyst *via ^n^* PrNH_2_ activation [157], the utilization of cyclometallated Ir‐complex (*S,S,S_a_*)‐**K1** derived from (*S,S,S_a_*)‐**L2** improved the reaction efficiency (Entry 9). When the reaction was conducted with the enantiomeric iridium catalyst (*R*, *R*, *R_a_*)‐**K1**, the diastereoselectivity was altered dramatically (**4a**:**4a′**:**4a″** = 2:10:1), leading to **4a′** as the major diastereomer in 20% NMR yield and 98% ee (Entry 10). The utilization of a diastereomeric iridium catalyst (*S*,*S*,*R_a_*)‐**K1′** totally shut down the reactivity (Entry 11). Although the detailed mechanism remains unclear, the temperature in the first step was crucial for the reaction. When the temperature was increased from −30°C to 0°C and rt, respectively, **4a** could be obtained in improved NMR yields (70%–81%), diastereoselectivity (10:1 dr), and enantioselectivity (>99% ee) (Entries 12 and 13). Further studies on the concentration of substrates and catalyst loading indicated that the utilization of decreased loadings of Cu(OTf)_2_ (5 mol%) and (*S*,*S*)‐**L1** (5 mol%) at a lower concentration (*c* = 0.05 m for **1a**) afforded **4a** with better results (63% isolated yield, 12:1 dr, and 99% ee, Entry 18).

**TABLE 1:** Optimization of reaction conditions.^a^

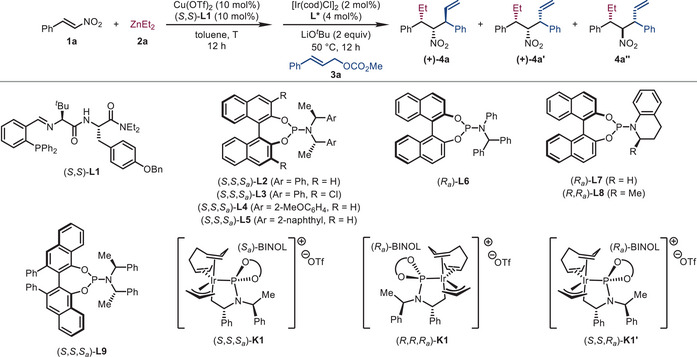						
Entry	L*	T (°C)	yield of 4a (%)^b^	dr (4a:4a′)^b^	ee of 4a (%)^c^	ee of 4a′ (%)^c^
1	(*S*,*S*,*S_a_*)‐**L2**	−30	64	6.4:1	98 (+)	26 (−)
2	(*S*,*S*,*S_a_*)‐**L3**	−30	15	3:1	97 (+)	63 (−)
3	(*S*,*S*,*S_a_*)‐**L4**	−30	42	6:1	98 (+)	9 (+)
4	(*S*,*S*,*S_a_*)‐**L5**	−30	48	6:1	98 (+)	40 (−)
5	(*R_a_*)‐**L6**	−30	22	1:1.1	53 (−)	79 (−)
6	(*R_a_*)‐**L7**	−30	13	1.1:1	86 (+)	27 (+)
7	(*R*,*R_a_*)‐**L8**	−30	18	1.4:1	89 (+)	42 (+)
8	(*S*,*S*,*S_a_*)‐**L9**	−30	63	6.3:1	98 (+)	56 (−)
9^d^	(*S*,*S*,*S_a_*)‐**K1**	−30	72	6.6:1	98 (+)	35 (−)
10^d^	(*R*,*R*,*R_a_*)‐**K1**	−30	20^e^	2:10:1^f^	45 (−)	98 (+)
11^d^	(*S*,*S*,*R_a_*)‐**K1′**	−30	trace	N.D.^g^	N.D.^g^	N.D.^g^
12^d^	(*S*,*S*,*S_a_*)‐**K1**	0	70	10:1	>99 (+)	8 (+)
13^d^	(*S*,*S*,*S_a_*)‐**K1**	Rt	81	10:1	>99 (+)	3 (−)
14^d^, ^h^	(*S*,*S*,*S_a_*)‐**K1**	Rt	76	11:1	>99 (+)	2 (−)
15^d^, ^i^	(*S*,*S*,*S_a_*)‐**K1**	Rt	55	7.9:1	99 (+)	4 (−)
16^d^, ^h^, ^j^	(*S*,*S*,*S_a_*)‐**K1**	Rt	74	9.3:1	99 (+)	28 (−)
17^d^, ^h^, ^k^	(*S*,*S*,*S_a_*)‐**K1**	Rt	64	9.1:1	99 (+)	21 (−)
18^d^, ^h^, ^i^	(*S*,*S*,*S_a_*)‐**K1**	Rt	73 (63**^n^**)	12:1	99 (+)	N.D.^g^
19^d^, ^h^, ^m^	(*S*,*S*,*S_a_*)‐**K1**	Rt	73 (62^n^)	10:1	98 (+)	N.D.^g^

^a^ Reaction conditions: **1a** (0.1 mmol), **2a** (0.12 mmol), Cu(OTf)_2_ (10 mol%), (*S*,*S*)‐**L1** (10 mol%) in toluene (1 mL) at −30°C for 12 h, then [Ir(cod)Cl]_2_ (2 mol%), L* (4 mol%), LiO*^t^* Bu (0.2 mmol) and **3a** (0.12 mmol) were added and stirred at 50°C for 12 h. Iridium catalyst was prepared via *^n^* PrNH_2_ activation [157].

^b^ Determined by ^1^H NMR analysis of the crude reaction mixture using mesitylene as an internal standard.

^c^ Determined by HPLC analysis with a chiral stationary phase.

^d^ Independently prepared iridium complex **K** (4 mol%) was used.

^e^ The yield of **4a′**.

^f^ The ratio of **4a**:**4a′**:**4a″** is shown. The ee value of **4a″** was not determined.

^g^ N.D.: Not determined.

^h^ In toluene (2.0 mL).

^i^ In toluene (4.0 mL).

^j^ With (*S,S,S_a_*)‐**K1** (2 mol%).

^k^ With (*S,S,S_a_*)‐**K1** (1 mol%).

^l^ With Cu(OTf)_2_ (5 mol%) and (*S,S*)‐**L1** (5 mol%) in a 0.3 mmol‐scale reaction.

^m^ With Cu(OTf)_2_ (2 mol%) and (*S,S*)‐**L1** (2 mol%) in a 0.3 mmol‐scale reaction.

^n^ Isolated yield.

With the optimized reaction conditions in hand (Entry 18, Table 1), we next investigated the scope of allylic carbonates (Table 2). Generally, cinnamyl carbonates **3** bearing substituents with varied electronic properties (fluorine, chlorine, bromine, methyl, methoxy, methylenedioxy, trifluoromethyl, and phenyl) at all positions around the phenyl ring were well tolerated, and the desired products (**4b**–**4q**) were obtained in reasonable yields (59%–70%) with excellent diastereo‐ and enantio‐selectivities (8:1–12:1 dr, and 97 to >99% ee). Notably, the unfavorable *ortho* substituent effect, commonly seen in Ir‐catalyzed allylic substitution reactions with the Feringa‐type ligands [162, 163, 164, 165, 166, 167, 168, 169, 170, 171], was not observed, and **4b** (*o*‐methyl) was obtained in 66% yield, 10:1 dr, and 99% ee. Furthermore, the reaction was compatible with 2‐naphthyl, 2‐pyridyl, and 1‐thienyl allyl carbonates, leading to their corresponding products smoothly (**4r**–**4t**, 63%–72% yields, 9:1–11:1 dr, and 98%–99% ee).

**TABLE 2:** Substrate scope: Allylic carbonates.^a^

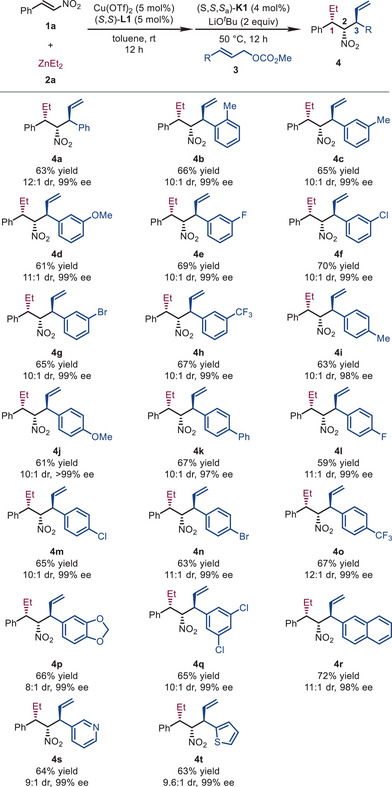

^a^ Reaction conditions: **1a** (0.3 mmol), **2a** (1.2 equiv), Cu(OTf)_2_ (5 mol%), (*S*,*S*)‐**L1** (5 mol%) in toluene (6.0 mL) at rt for 12 h, then (*S*,*S*,*S_a_*)‐**K1** (4 mol%), LiO*^t^* Bu (2.0 equiv) and **3** (1.2 equiv) were added and stirred at 50°C for 12 h. The dr values were determined by ^1^H NMR analysis of the crude reaction mixtures. The ee values were determined by HPLC analysis with a chiral stationary phase.

Then, the scope of nitroalkenes and organozinc reagents was tested (Table 3). Halogen‐substituted (fluorine, chlorine, bromine, or iodine) nitrostyrenes were compatible with the reaction conditions, and their corresponding products (**5a**, **5c**–**5d** and **5g**–**5j**) were obtained in good yields (52%–64%) with excellent diastereo‐ and enantio‐selectivities (7.2:1–12:1 dr, and 98%–99% ee). Nitrostyrenes bearing an electron‐donating (methyl and methoxy) group at the *meta*‐ and *para*‐positions of the phenyl ring also performed well, affording the products (**5b**, **5e**–**5f,** and **5k**) in good results (52%–66% yields, 8:1–12:1 dr, and 98% to >99% ee). When 2‐naphthyl and 1‐thienyl nitroalkenes were tested, the desired products **5l** (69% yield, 10:1 dr, and 99% ee) and **5m** (59% yield, 9:1 dr, and 99% ee) were obtained smoothly. Instead of ZnEt_2_, ZnMe_2_ was well compatible, which gave **5n** in 58% yield, 10:1 dr, and 99% ee. The absolute configuration of **5n** (1*S*,2*R*,3*S*) was determined by X‐ray crystallographic analysis unambiguously [172]. The absolute configuration of other products was assigned by analogy.

**TABLE 3:** Substrate scope: Nitroalkenes and organozinc reagents.^a^

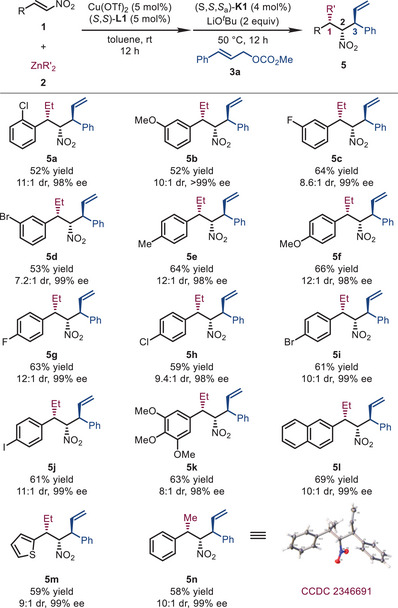

^a^ Reaction conditions: **1** (0.3 mmol), **2** (1.2 equiv), Cu(OTf)_2_ (5 mol%), (*S*,*S*)‐**L1** (5 mol%) in toluene (6.0 mL) at rt for 12 h, then (*S*,*S*,*S_a_*)‐**K1** (4 mol%), LiO*^t^* Bu (2.0 equiv), and **3a** (1.2 equiv) were added and stirred at 50°C for 12 h. The dr values were determined by ^1^H NMR analysis of the crude reaction mixtures. The ee values were determined by HPLC analysis with a chiral stationary phase.

**SCHEME 2:**
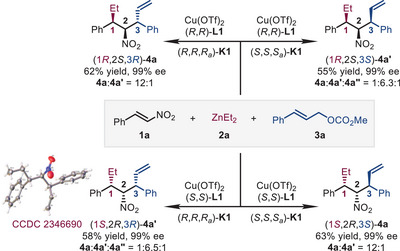
Stereodivergent accesses to four stereoisomers with multi‐contiguous stereocenters.

To demonstrate the stereodivergent synthesis of aliphatic nitro compounds bearing multi‐contiguous stereocenters, the reactions of **1a**, **2a,** and **3a** using chiral catalysts with varied configurations were performed (Scheme 2). Four diastereomers of the target molecules, **4a** in (1*R*,2*S*,3*R*) or (1*S*,2*R*,3*S*) configuration and **4a′** in (1*R*,2*S*,3*S*) or (1*S*,2*R*,3*R*) configuration, obtained as the major product, respectively, in 55%–63% yields and 99% ee. Notably, a small amount of diastereomer **4a″** was observed when the chiral source of the catalysts was composed of (*S*,*S*)‐**L1** and (*R*,*R*,*R_a_*)‐**K1**, or their enantiomers, (*R*,*R*)‐**L1** and (*S*,*S*,*S_a_*)‐**K1**. The absolute configuration of (1*S*,2*R*,3*R*)‐**4a′** was confirmed by X‐ray crystallographic analysis [172].

**SCHEME 3:**
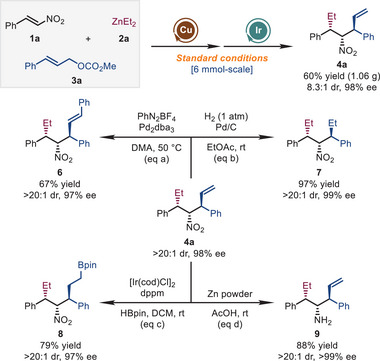
Gram‐scale synthesis and transformations of **4a**.

The practicality and utility of this method were demonstrated by gram‐scale synthesis and diverse transformations of the product (Scheme 3). When the reaction was performed on a 6 mmol‐scale, product **4a** was delivered in 60% yield (1.06 g), 8.3:1 dr, and 98% ee. In addition, several transformations of **4a** were conducted. First, palladium‐catalyzed Heck reaction of **4a** with PhN_2_BF_4_ gave product **6** in 67% yield (eq a). Hydrogenation of the terminal double bond in **4a** by Pd/C led to product **7** in 97% yield (eq b). Furthermore, iridium‐catalyzed *anti*‐Markovnikov hydroboration afforded the alkyl boric ester **8** in 79% yield (eq c). Finally, the nitro group in **4a** could be readily reduced with Zn in AcOH to obtain amine **9** in 88% yield (eq d). Notably, all the products were obtained without losing the integrity of stereoselectivity (>20:1 dr and 97 to >99% ee) in these transformations.

## 3 Conclusions

In summary, we reported a stereodivergent synthesis of aliphatic nitro compounds bearing multi‐contiguous stereocenters by combining copper‐catalyzed asymmetric conjugate addition of dialkylzinc reagents to nitroalkenes with iridium‐catalyzed asymmetric allylic substitution reaction. The reaction generally proceeds in good yields with excellent diastereo‐ and enantio‐selectivities, enabling the stereodivergent synthesis of nonadjacent stereocenters (1,3‐positions). Gram‐scale synthesis and transformations of products further enhance the potential utility of this method.

## Conflicts of Interest

The authors declare no conflicts of interest.

## Supporting information




**Supporting File**: advs72419‐sup‐0001‐SuppMat.docx.

## Data Availability

The data that support the findings of this study are available in the supplementary material of this article.;
