# Supplementary material for: Stereodivergent Access to Aliphatic Nitro Compounds Bearing Multi‐Contiguous Stereocenters via Sequential Catalysis
Source: Adv Sci (Weinh). 2026 Feb 3;13(14):e19142. doi: 10.1002/advs.202519142 (PMC12970203; doi:10.1002/advs.202519142)
Supplement: Supplementary file 1 — Supporting File: advs72419‐sup‐0001‐SuppMat.docx. [file ADVS-13-e19142-s001.docx]

**Table of contents**

1. [Materials and methods](#_Toc121338347) S2

[2. Optimization of the reaction conditions S4](#_Toc121338348)

[3. General procedure for the synthesis of aliphatic nitro compounds bearing multi- contiguous stereocenters S11](#_Toc121338349)

[4. Full characterization of products S13](#_Toc121338350)

[5. General procedure for the stereodivergent synthesis S30](#_Toc121338351)

[6. Gram-scale reaction and product transformations S33](#_Toc121338352)

[7. X-Ray crystal structures of **4a′** and **5n** and the assignment of configuration of **4a′′** S38](#_Toc121338353)

[8. References S43](#_Toc121338354)

[9. Copies of NMR spectra S44](#_Toc121338355)

[10. Copies of HPLC chromatograms S130](#_Toc121338442)

## Materials and methods

1. Techniques

^1^H and ^13^C NMR spectra were recorded on a Varian instrument (400 MHz and 100 MHz) or an Agilent instrument (400 MHz and 100 MHz) or a Bruker instrument (400 MHz and 100 MHz) and internally referenced to tetramethylsilane signal or residual protio solvent signals. ^19^F NMR spectra were recorded on an Agilent instrument (376 MHz) or a Bruker instrument (376 MHz) and externally referenced to CFCl_3_. Data for ^1^H NMR are recorded as follows: chemical shift (δ, ppm), multiplicity (s = singlet, d = doublet, t = triplet, m = multiplet or unresolved, coupling constant(s) in Hz, integration). Data for ^13^C NMR and ^19^F NMR are reported regarding chemical shift (δ, ppm). Enantiomeric excess values were determined by HPLC analysis on a chiral stationary phase on Waters 2489 UV/Visible detector, Waters 1525 binary HPLC pump and Waters 2707 autosampler or supercritical fluid chromatography (SFC) analysis using Waters UPC2 instruments. Optical rotations were measured in CHCl_3_ on a Rudolph Autopol I/II/III or Rudolph APVI polarimeter with a sodium lamp of wavelength 589 nm. IR spectra were obtained on Bruker Tensor 27 instruments with Bruker Platinum ATR accessory. Melting points were measured on Shenguang X-4 melting point apparatus. ESI and DART were recorded on Agilent 6224 TOF LC/MS and Thermo Fisher Scientific LTQ FTICR-MS. X-Ray crystallographic analyses were performed on a Bruker APEX2 at 293 K.

1. Reagents and solvents

Unless stated otherwise, all reactions were carried out in flame-dried glassware under a dry argon atmosphere. All solvents were purified and dried according to standard methods before use.

Substrate **1a** was commercially available and used after recrystallization from ethanol; substrates **1b**-**1n** were synthesized according to the known literature (**Figure S1**).^[1]^ Substrates **2a** (diethylzinc, 1M in n-hexane) and **2b** (dimethylzinc, 1M in toluene) were commercially available and used directly without purification. According to the known literature, compounds **3** (allylic carbonates) were synthesized.^[2]^

Figure S1. Nitroalkene substrates.

(c) Purification

Analytical thin layer chromatography (TLC) was performed on silica gel-coated glass plate obtained from Huanghai and visualized with 254 nm light. Flash chromatography was performed using silica gel obtained from Silicycle (230-400 mesh). Preparative thin-layer chromatography (PTLC) was performed on gel-coated glass plate obtained from Huanghai and visualized with 254 nm light.

# Optimization of the reaction conditions

**Table S1.** Screening of iridium catalyst.^[a]^

| entry | **LIr** | yield of **4a** (%)^[b]^ | dr (**4a**:**4a′**)^[b]^ | ee of **4a** (%)^[c]^ | ee of **4a′** (%)^[c]^ |
| --- | --- | --- | --- | --- | --- |
| 1 | (*S*,*S*,*S_a_*)-**L2** | 64 | 6.4:1 | 98 (+) | 26 (-) |
| 2 | (*S*,*S*,*S_a_*)-**L3** | 15 | 3.0:1 | 97 (+) | 63 (-) |
| 3 | (*S*,*S*,*S_a_*)-**L4** | 42 | 6.0:1 | 98 (+) | 9 (+) |
| 4 | (*S*,*S*,*S_a_*)-**L5** | 48 | 6.0:1 | 98 (+) | 40 (-) |
| 5 | (*R_a_*)-**L6** | 22 | 1:1.1 | 53 (-) | 79 (-) |
| 6 | (*R_a_*)-**L7** | 13 | 1.1:1 | 86 (+) | 27 (+) |
| 7 | (*R*,*R_a_*)-**L8** | 18 | 1.4:1 | 89 (+) | 42 (+) |
| 8 | (*S*,*S*,*S_a_*)-**L9** | 63 | 6.3:1 | 98 (+) | 56 (-) |
| 9^[d]^ | (*S*,*S*,*S_a_*)-**K1** | 72 | 6.6:1 | 98 (+) | 35 (-) |
| 10^[d]^ | (*R*,*R*,*R_a_*)-**K1** | 20^[e]^ | 2:10:1^[f]^ | 45 (-) | 98 (+) |
| 11^[d]^ | (*S*,*S*,*R_a_*)-**K1′** | trace | N.D.^[g]^ | N.D.^[g]^ | N.D.^[g]^ |

[a] Reaction conditions: **1a** (0.1 mmol), **2a** (0.12 mmol), Cu(OTf)_2_ (10 mol%), (*S*,*S*)-**L1** (10 mol%) in toluene (1.0 mL) at -30 °C for 12 h, then [Ir(cod)Cl]_2_ (2 mol%), **LIr** (4 mol%), LiO*^t^*Bu (0.2 mmol) and **3a** (0.12 mmol) were added and stirred at 50 °C for 12 h. Iridium catalyst was prepared via *^n^*PrNH_2_ activation.^[3]^ [b] Determined by ^1^H NMR of the crude reaction mixture using mesitylene as an internal standard. [c] Determined by HPLC analysis with a chiral stationary phase. [d] Independently prepared iridium complex **K** (4 mol%) was used. [e] The yield of **4a′**. [f] dr = **4a**:**4a′**:**4a′′**, the ee value of product **4a′′** was not determined. [g] N.D.: Not determined.

**Table S2.** Screening of base.^[a]^

| entry | base | yield of **4a** (%)^[b]^ | dr (**4a**:**4a′**)^[b]^ | ee of **4a** (%)^[c]^ | ee of **4a′** (%)^[c]^ |
| --- | --- | --- | --- | --- | --- |
| 1 | No | N.R.^[d]^ | N.D.^[e]^ | N.D.^[e]^ | N.D.^[e]^ |
| 2 | LiO*^t^*Bu | 72 | 6.6:1 | 98 (+) | 35 (-) |
| 3 | NaO*^t^*Bu | 17 | 3.4:1 | 99 (+) | 54 (-) |
| 4 | KO*^t^*Bu | 17 | 3.4:1 | 99 (+) | 68 (-) |
| 5 | Cs_2_CO_3_ | 45 | 6.4:1 | 98 (+) | 52 (-) |
| 6 | DABCO | 26 | 6.5:1 | 99 (+) | 65 (-) |
| 7 | NEt_3_ | 12 | 4.0:1 | 89 (+) | 13 (+) |
| 8 | DBU | 13 | 6.5:1 | 89 (+) | 89 (-) |
| 9 | LiHMDS | 9 | 1.0:1 | 98 (+) | 13 (-) |

[a] Reaction conditions: **1a** (0.1 mmol), **2a** (0.12 mmol), Cu(OTf)_2_ (10 mol%), (*S*,*S*)-**L1** (10 mol%) in toluene (1.0 mL) at -30 °C for 12 h, then (*S*,*S*,*S_a_*)-**K1** (4 mol%), base (0.2 mmol) and **3a** (0.12 mmol) were added and stirred at 50 °C for 12 h. [b] Determined by ^1^H NMR of the crude reaction mixture using mesitylene as an internal standard. [c] Determined by HPLC analysis with a chiral stationary phase. [d] N.R.: No reaction. [e] N.D.: Not determined.

**Table S3.** Screening of solvent.^[a]^

| entry | solvent | yield of **4a** (%)^[b]^ | dr (**4a**:**4a′**)^[b]^ | ee of **4a** (%)^[c]^ | ee of **4a′** (%)^[c]^ |
| --- | --- | --- | --- | --- | --- |
| 1 | toluene | 72 | 6.6:1 | 98 (+) | 35 (-) |
| 2 | mesitylene | 34 | 4.9:1 | 98 (+) | 56 (-) |
| 3 | PhCl | 71 | 5.9:1 | 97 (+) | 26 (-) |
| 4 | PhCF_3_ | 62 | 4.8:1 | 96 (+) | 27 (-) |
| 5 | *^t^*BuOMe | 40 | 5.0:1 | 97 (+) | 62 (-) |
| 6 | THF | 40 | 7.4:4.8:1^[d]^ | 90 (+) | 90 (-) |
| 7 | DCM | 55 | 5.0:1 | 96 (+) | 49 (-) |
| 8 | MeCN | N.R.^[e]^ | N.D.^[f]^ | N.D.^[f]^ | N.D.^[f]^ |

[a] Reaction conditions: **1a** (0.1 mmol), **2a** (0.12 mmol), Cu(OTf)_2_ (10 mol%), (*S*,*S*)-**L1** (10 mol%) in solvent (1.0 mL) at -30 °C for 12 h, then (*S*,*S*,*S_a_*)-**K1** (4 mol%), LiO*^t^*Bu (0.2 mmol) and **3a** (0.12 mmol) were added and stirred at 50 °C for 12 h. [b] Determined by ^1^H NMR of the crude reaction mixture using mesitylene as an internal standard. [c] Determined by HPLC analysis with a chiral stationary phase. [d] dr = **4a**:**4a′**:**4a″**, the ee value of product **4a″** was not determined. [e] N.R.: No reaction. [f] N.D.: Not determined.

**Table S4.** Screening of ligand for copper catalyst.^[a]^

| entry | **LCu** | yield of **4a** (%)^[b]^ | dr (**4a**:**4a′**)^[b]^ | ee of **4a** (%)^[c]^ | ee of **4a′** (%)^[c]^ |
| --- | --- | --- | --- | --- | --- |
| 1 | (*S*,*S*)-**L1** | 72 | 6.6:1 | 98 (+) | 35 (-) |
| 2^[d]^ | (*R*,*R*)-**L10** | messy | N.D.^[e]^ | N.D.^[e]^ | N.D.^[e]^ |
| 3^[f]^ | (*S*,*S*)-**L11** | 52 | 6.5:1 | 98 (+) | 0 |
| 4^[f]^ | (*S*,*S*,*R_a_*)-**L12** | 18 | 9:10:1^[g]^ | 76 (+) | 90 (-) |
| 5^[f]^ | (*R_a_*)**-L6** | N.R.^[h]^ | N.D.^[e]^ | N.D.^[e]^ | N.D.^[e]^ |
| 6^[f]^ | (*R*,*R*)-**L13** | 43 | 3.6:1 | 94 (+) | 52 (-) |

[a] Reaction conditions: **1a** (0.1 mmol), **2a** (0.12 mmol), Cu(OTf)_2_ (10 mol%), **LCu** (10 mol%) in toluene (1.0 mL) at -30 °C for 12 h, then (*S*,*S*,*S_a_*)-**K1** (4 mol%), LiO*^t^*Bu (0.2 mmol) and **3a** (0.12 mmol) were added and stirred at 50 °C for 12 h. [b] Determined by ^1^H NMR of the crude reaction mixture using mesitylene as an internal standard. [c] Determined by HPLC analysis with a chiral stationary phase. [d] With **LCu** (40 mol%). [e] N.D.: Not determined. [f] With **LCu** (20 mol%). [g] dr = **4a**:**4a′**:**4a″**, the ee value of product **4a″** was not determined. [h] N.R.: No reaction.

**Table S5.** Screening of temperature.^[a]^

| entry | T (°C) | yield of **4a** (%)^[b]^ | dr (**4a**:**4a′**)^[b]^ | ee of **4a** (%)^[c]^ | ee of **4a′** (%)^[c]^ |
| --- | --- | --- | --- | --- | --- |
| 1 | rt | 81 | 10:1 | >99 (+) | 3 (-) |
| 2 | 0 | 70 | 10:1 | >99 (+) | 8 (+) |
| 3 | -30 | 72 | 6.6:1 | 98 (+) | 35 (-) |
| 4 | -45 | 52 | 3.5:1 | 95 (+) | 60 (-) |
| 5 | -78 | 52 | 13:5.7:1^[d]^ | 91 (+) | 76 (-) |

[a] Reaction conditions: **1a** (0.1 mmol), **2a** (0.12 mmol), Cu(OTf)_2_ (10 mol%), (*S*,*S*)-**L1** (10 mol%) in toluene (1.0 mL) at T °C for 12 h, then (*S*,*S*,*S_a_*)-**K1** (4 mol%), LiO*^t^*Bu (0.2 mmol) and **3a** (0.12 mmol) were added and stirred at 50 °C for 12 h. [b] Determined by ^1^H NMR of the crude reaction mixture using mesitylene as an internal standard. [c] Determined by HPLC analysis with a chiral stationary phase. [d] dr = **4a**:**4a′**:**4a″**, the ee value of product **4a″** was not determined.

**Table S6.** Screening of copper source.^[a]^

| entry | [Cu] | yield of **4a** (%)^[b]^ | dr (**4a**:**4a′**)^[b]^ | ee of **4a** (%)^[c]^ | ee of **4a′** (%)^[c]^ |
| --- | --- | --- | --- | --- | --- |
| 1 | CuCl | 47 | 14:6.9:1^[d]^ | 83 (+) | 70 (-) |
| 2 | CuBr | 32 | 18:7.7:1^[d]^ | 89 (+) | 54 (-) |
| 3 | CuI | 40 | 4.0:1 | 94 (+) | 29 (-) |
| 4 | Cu(MeCN)_4_BF_4_ | 71 | 8.9:1 | 99 (+) | 8 (-) |
| 5 | (CuOTf)_2_**⸱**toluene | 73 | 9.1:1 | 99 (+) | 8 (+) |
| 6 | Cu(OTf)_2_ | 81 | 10:1 | >99 (+) | 3 (-) |
| 7 | Cu(OAc)_2_ | 61 | 3.2:1 | 93 (+) | 59 (-) |

[a] Reaction conditions: **1a** (0.1 mmol), **2a** (0.12 mmol), [Cu] (10 mol%), (*S*,*S*)-**L1** (10 mol%) in toluene (1.0 mL) at rt for 12 h, then (*S*,*S*,*S_a_*)-**K1** (4 mol%), LiO*^t^*Bu (0.2 mmol) and **3a** (0.12 mmol) were added and stirred at 50 °C for 12 h. [b] Determined by ^1^H NMR of the crude reaction mixture using mesitylene as an internal standard. [c] Determined by HPLC analysis with a chiral stationary phase. [d] dr = **4a**:**4a′**:**4a″**, the ee value of product **4a″** was not determined.

**Table S7.** Screening of other conditions.^[a]^

| entry | x | y | yield of **4a** (%)^[b]^ | dr (**4a**:**4a′**)^[b]^ | ee of **4a** (%)^[c]^ | ee of **4a′** (%)^[c]^ |
| --- | --- | --- | --- | --- | --- | --- |
| 1^[d]^ | 10 | 4 | 80 | 10:1 | 99 (+) | 5 (+) |
| 2 | 10 | 4 | 81 | 10:1 | >99 (+) | 3 (-) |
| 3^[e]^ | 10 | 4 | 76 | 11:1 | >99 (+) | 2 (-) |
| 4^[f]^ | 10 | 4 | 55 | 7.9:1 | 99 (+) | 4 (-) |
| 5^[e]^ | 10 | 2 | 74 | 9.3:1 | 99 (+) | 28 (-) |
| 6^[e]^ | 10 | 1 | 64 | 9.1:1 | 99 (+) | 21 (-) |
| 7^[e,g]^ | 5 | 4 | 73 (63^[h]^) | 12:1 | 99 (+) | N.D.^[i]^ |
| 8^[e,g]^ | 2 | 4 | 73 (62^[h]^) | 10:1 | 98 (+) | N.D.^[i]^ |

[a] Reaction conditions: **1a** (0.1 mmol), **2a** (0.12 mmol), Cu(OTf)_2_ (x mol%), (*S*,*S*)-**L1** (x mol%) in toluene (1.0 mL) at rt for 12 h, then (*S*,*S*,*S_a_*)-**K1** (y mol%), LiO*^t^*Bu (0.2 mmol) and **3a** (0.12 mmol) were added and stirred at 50 °C for 12 h. [b] Determined by ^1^H NMR of the crude reaction mixture using mesitylene as an internal standard. [c] Determined by HPLC analysis with a chiral stationary phase. [d] In toluene (0.5 mL). [e] In toluene (2.0 mL). [f] In toluene (4.0 mL). [g] 0.3 mmol scale. [h] Isolated yield. [i] N.D.: Not determined.

# 3. General procedure for the synthesis of aliphatic nitro compounds bearing multi-contiguous stereocenters

1）Preparation of enantioenriched products of **4** and **5**

Under Ar atmosphere, Cu(OTf)_2_ (5.4 mg, 0.015 mmol, 5 mol%) and (*S*,*S*)-**L1** (10.7 mg, 0.015 mmol, 5 mol%) were added to an oven-dried Schlenk tube. The reaction tube was evacuated and refilled with argon three times. Freshly distilled toluene (6.0 mL) was added to the flask. After the mixture was stirred for 30 min, **1** (0.30 mmol, 1.0 equiv) and **2** (0.36 mmol, 1.2 equiv) were successively added to the flask. The reaction was stirred at rt for 12 h, (*S*,*S*,*S_a_*)-**K1** (12.3 mg, 0.012 mmol, 4 mol%), LiO*^t^*Bu (48.0 mg, 0.6 mmol, 2.0 equiv) and **3** (0.36 mmol, 1.2 equiv) were then added to the flask. The reaction was stirred at 50 ºC for 12 h and quenched with saturated NH_4_Cl solution (15 mL), and then the mixture was extracted with EtOAc (15 mL x 3). The combined organic fractions were washed with brine (15 mL), dried over Na_2_SO_4_, filtered, and concentrated by rotary evaporation. The diastereomeric ratio was determined by ^1^H NMR of the crude reaction mixture. Then the residue was purified by silica gel column chromatography (PE/EtOAc = 100/1-40/1) or PTLC (PE/EtOAc = 40/1) to afford the desired products **4** and **5**.

2）Preparation of racemic products of **4** and **5**

Under Ar atmosphere, Cu(OTf)_2_ (3.6 mg, 0.01 mmol, 5 mol%) and (*rac*)-**L2** (10.8 mg, 0.02 mmol, 10 mol%) were added to an oven-dried Schlenk tube. The reaction tube was evacuated and refilled with argon three times. Freshly distilled toluene (2.0 mL) was added to the flask. After the mixture was stirred for 30 min, **1** (0.20 mmol, 1.0 equiv) and **2** (0.24 mmol, 1.2 equiv) were successively added to the flask. The reaction was stirred at rt for 12 h, (*rac*)-**K1** (8.2 mg, 0.008 mmol, 4 mol%), LiO*^t^*Bu (32.0 mg, 0.4 mmol, 2.0 equiv) and **3** (0.24 mmol, 1.2 equiv) were then added to the flask. The reaction was stirred at 50 ºC for 12 h and quenched with saturated NH_4_Cl solution (10 mL), and then the mixture was extracted with EtOAc (10 mL x 3). The combined organic fractions were washed with brine (10 mL), dried over Na_2_SO_4_, filtered, and concentrated by rotary evaporation. The diastereomeric ratio was determined by ^1^H NMR of the crude reaction mixture. Then the residue was purified by silica gel column chromatography (PE/EtOAc = 100/1-40/1) or PTLC (PE/EtOAc = 40/1) to afford the desired products **4** and **5**.

# Full characterization of products

12:1 dr, for major diastereomer, **4a**, light yellow solid, m.p. = 54.6–55.3 °C, 55.7 mg, 63% yield, 99% ee. [Daicel Chiralcel IG (0.46 cm × 25 cm), *n*-hexane/2-propanol = 99/1, *v* = 1.0 mL·min^–1^, λ = 214 nm, t (major) = 6.18 min, t (minor) = 7.51 min]. [α]_D_^30^ = +83.5 (c = 1.0, CHCl_3_). ^1^H NMR (400 MHz, CDCl_3_) δ 7.38-7.29 (m, 2H), 7.29-7.17 (m, 4H), 7.16-7.11 (m, 2H), 7.11-7.06 (m, 2H), 5.84 (app-dt, *J* = 16.8, 9.6 Hz, 1H), 5.12 (d, *J* = 16.8 Hz, 1H), 5.06 (d, *J* = 10.0 Hz, 1H), 5.06-5.02 (m, 1H), 3.94 (app-t, *J* = 9.2 Hz, 1H), 3.15-3.01 (m, 1H), 1.96-1.82 (m, 1H), 1.82-1.64 (m, 1H), 0.71 (t, *J* = 7.2 Hz, 3H). ^13^C NMR (100 MHz, CDCl_3_) δ 139.3, 138.7, 136.2, 128.9, 128.8, 128.6, 128.0, 127.7, 127.6, 118.0, 96.6, 52.4, 48.9, 23.1, 12.0. IR (thin film): ν_max_ (cm^-1^) = 3028, 2970, 2934, 2902, 2879, 1545, 1493, 1452, 1369, 1306, 1250, 1071, 1032, 997, 939, 757, 699, 675, 577, 518. HRMS (DART) calcd for C_19_H_22_NO_2_ [M+H]^+^: 296.1645. Found: 296.1645.

10:1 dr, for major diastereomer, **4b**, light yellow oil, 60.8 mg, 66% yield, 99% ee. [Daicel Chiralcel OD-H (0.46 cm × 25 cm), *n*-hexane/2-propanol = 99/1, *v* = 1.0 mL·min^–1^, λ = 214 nm, t (major) = 6.19 min, t (minor) = 7.08 min]. [α]_D_^28^ = +119.2 (c = 1.0, CHCl_3_). ^1^H NMR (400 MHz, CDCl_3_) δ 7.36-7.23 (m, 3H), 7.19-7.03 (m, 6H), 5.62 (app-dt, *J* = 16.8, 9.6 Hz, 1H), 5.10 (d, *J* = 17.2 Hz, 1H), 5.16-5.09 (m, 1H), 4.99 (d, *J* = 10.0 Hz, 1H), 4.38 (app-t, *J* = 10.0 Hz, 1H), 3.16 (ddd, *J* = 11.6, 5.6, 3.2 Hz, 1H), 2.32 (s, 3H), 2.06-1.90 (m, 1H), 1.90-1.71 (m, 1H), 0.75 (t, *J* = 7.2 Hz, 3H). ^13^C NMR (100 MHz, CDCl_3_) δ 139.5, 137.1, 136.2, 136.0, 131.1, 128.9, 128.5, 127.6, 127.2, 126.5, 125.9, 117.7, 96.0, 49.2, 47.7, 22.3, 19.6, 12.2. IR (thin film): ν_max_ (cm^-1^) = 3063, 3026, 2968, 2933, 2876, 1546, 1492, 1455, 1368, 1321, 991, 922, 752, 726, 699, 664, 577, 547, 454. HRMS (DART) calcd for C_20_H_24_NO_2_ [M+H]^+^: 310.1802. Found: 310.1801.

10:1 dr, for major diastereomer, **4c**, light yellow oil, 60.1 mg, 65% yield, 99% ee. [Waters upc, SFC system, IC-3 (0.30 cm × 15 cm), CO_2_/MeOH = 98/2, *v* = 1.0 mL•min^-1^, λ = 214 nm, t (major) = 2.69 min, t (minor) = 2.80 min]. [α]_D_^29^ = +84.7 (c = 1.0, CHCl_3_). ^1^H NMR (400 MHz, CDCl_3_) δ 7.37-7.28 (m, 2H), 7.31-7.22 (m, 1H), 7.18-7.09 (m, 3H), 7.01 (d, *J* = 7.6 Hz, 1H), 6.94-6.85 (m, 2H), 5.83 (ddd, *J* = 16.8, 10.0, 9.2 Hz, 1H), 5.12 (d, *J* = 16.8 Hz, 1H), 5.05 (d, *J* = 10.0 Hz, 1H), 5.05-5.00 (m, 1H), 3.91 (app-t, *J* = 9.2 Hz, 1H), 3.08 (ddd, *J* = 11.2, 6.8, 3.2 Hz, 1H), 2.27 (s, 3H), 1.95-1.83 (m, 1H), 1.82-1.67 (m, 1H), 0.71 (t, *J* = 7.2 Hz, 3H). ^13^C NMR (100 MHz, CDCl_3_) δ 139.3, 138.6, 138.4, 136.3, 128.9, 128.70, 128.68, 128.6, 128.4, 127.6, 124.9, 117.8, 96.6, 52.4, 48.9, 23.0, 21.5, 12.0. IR (thin film): ν_max_ (cm^-1^) = 3028, 2968, 2932, 2876, 1606, 1546, 1492, 1453, 1368, 991, 924, 771, 750, 700, 583, 554, 442. HRMS (DART) calcd for C_20_H_24_NO_2_ [M+H]^+^: 310.1802. Found: 310.1801.

11:1 dr, for major diastereomer, **4d**, light yellow oil, 60.0 mg, 61% yield, 99% ee. [Daicel Chiralcel OD-H (0.46 cm × 25 cm), *n*-hexane/2-propanol = 99/1, *v* = 1.0 mL·min^–1^, λ = 214 nm, t (minor) = 9.26 min, t (major) = 12.71 min]. [α]_D_^29^ = +84.2 (c = 1.0, CHCl_3_). ^1^H NMR (400 MHz, CDCl_3_) δ 7.37-7.23 (m, 3H), 7.21-7.09 (m, 3H), 6.74 (dd, *J* = 8.0, 2.8 Hz, 1H), 6.69 (d, *J* = 8.0 Hz, 1H), 6.65-6.60 (m, 1H), 5.82 (app-dt, *J* = 16.8, 9.6 Hz, 1H), 5.13 (d, *J* = 16.8 Hz, 1H), 5.07-5.00 (m, 1H), 5.06 (d, *J* = 9.6 Hz, 1H), 3.92 (app-t, *J* = 9.2 Hz, 1H), 3.73 (s, 3H), 3.09 (ddd, *J* = 11.6, 6.8, 3.2 Hz, 1H), 1.96-1.82 (m, 1H), 1.82-1.66 (m, 1H), 0.71 (t, *J* = 7.2 Hz, 3H). ^13^C NMR (100 MHz, CDCl_3_) δ 159.8, 140.2, 139.3, 136.0, 129.8, 128.9, 128.6, 127.6, 120.1, 118.0, 114.0, 112.9, 96.5, 55.2, 52.4, 48.9, 23.0, 12.0. IR (thin film): ν_max_ (cm^-1^) = 3028, 2967, 2935, 2876, 2836, 1601, 1586, 1546, 1490, 1453, 1368, 1320, 1262, 1155, 1043, 992, 925, 872, 772, 750, 699, 570, 465. HRMS (ESI) calcd for C_20_H_23_NO_3_Na [M+Na]^+^: 348.1570. Found: 348.1577.

10:1 dr, for major diastereomer, **4e**, light yellow oil, 65.2 mg, 69% yield, 99% ee. [Daicel Chiralcel OD-H (0.46 cm × 25 cm), *n*-hexane/2-propanol = 99/1, *v* = 1.0 mL·min^–1^, λ = 214 nm, t (major) = 6.50 min, t (minor) = 8.14 min]. [α]_D_^29^ = +69.0 (c = 1.0, CHCl_3_). ^1^H NMR (400 MHz, CDCl_3_) δ 7.37-7.24 (m, 3H), 7.24-7.17 (m, 1H), 7.14-7.08 (m, 2H), 6.94-6.85 (m, 2H), 6.83-6.77 (m, 1H), 5.80 (ddd, *J* = 17.2, 10.0, 9.2 Hz, 1H), 5.12 (d, *J* = 17.2 Hz, 1H), 5.08 (d, *J* = 10.0 Hz, 1H), 5.03 (dd, *J* = 9.2, 6.8 Hz, 1H), 3.93 (app-t, *J* = 9.2 Hz, 1H), 3.08 (ddd, *J* = 10.8, 6.8, 3.2 Hz, 1H), 1.93-1.80 (m, 1H), 1.80-1.65 (m, 1H), 0.71 (t, *J* = 7.2 Hz, 3H). ^13^C NMR (100 MHz, CDCl_3_) δ 162.8 (d, *J* = 245.1 Hz), 141.1 (d, *J* = 6.9 Hz), 139.0, 135.5, 130.3 (d, *J* = 8.2 Hz), 129.0, 128.6, 127.7, 123.7 (d, *J* = 2.9 Hz), 118.5, 115.1 (d, *J* = 21.7 Hz), 114.7 (d, *J* = 20.8 Hz), 96.4, 52.0 (d, *J* = 1.8 Hz), 48.9, 23.3, 11.9. ^19^F NMR (376 MHz, CDCl_3_) δ -112.4 (m). IR (thin film): ν_max_ (cm^-1^) = 3029, 2969, 2934, 2877, 1613, 1590, 1546, 1489, 1451, 1367, 1244, 1147, 990, 927, 874, 778, 750, 698, 569, 522, 460. HRMS (DART) calcd for C_19_H_21_NO_2_F [M+H]^+^: 314.1551. Found: 314.1551.

10:1 dr, for major diastereomer, **4f**, light yellow oil, 68.9 mg, 70% yield, 99% ee. [Daicel Chiralcel OD-H (0.46 cm × 25 cm), *n*-hexane/2-propanol = 99/1, *v* = 1.0 mL·min^–1^, λ = 214 nm, t (major) = 6.27 min, t (minor) = 8.43 min]. [α]_D_^29^ = +73.5 (c = 1.0, CHCl_3_). ^1^H NMR (400 MHz, CDCl_3_) δ 7.36-7.24 (m, 3H), 7.21-7.14 (m, 2H), 7.14-7.08 (m, 2H), 7.08-7.03 (m, 1H), 7.02-6.94 (m, 1H), 5.79 (ddd, *J* = 16.8, 10.0, 9.2 Hz, 1H), 5.11 (d, *J* = 16.8 Hz, 1H), 5.08 (d, *J* = 10.0 Hz, 1H), 5.03 (dd, *J* = 9.2, 7.2 Hz, 1H), 3.90 (app-t, *J* = 9.2 Hz, 1H), 3.15-2.98 (m, 1H), 1.92-1.80 (m, 1H), 1.80-1.64 (m, 1H), 0.70 (t, *J* = 7.2 Hz, 3H). ^13^C NMR (100 MHz, CDCl_3_) δ 140.6, 138.9, 135.3, 134.5, 130.0, 129.0, 128.6, 128.3, 127.9, 127.7, 126.2, 118.6, 96.3, 51.9, 48.8, 23.3, 11.9. IR (thin film): ν_max_ (cm^-1^) = 3063, 3029, 2969, 2933, 2876, 1595, 1574, 1546, 1476, 1454, 1432, 1366, 1081, 990, 928, 783, 745, 722, 696, 585, 557, 443. HRMS (DART) calcd for C_19_H_24_N_2_O_2_Cl [M+NH_4_]^+^: 347.1521. Found: 347.1520.

10:1 dr, for major diastereomer, **4g**, light yellow oil, 72.7 mg, 65% yield, 99% ee. [Daicel Chiralcel OD-H (0.46 cm × 25 cm), *n*-hexane/2-propanol = 99/1, *v* = 1.0 mL·min^–1^, λ = 214 nm, t (major) = 4.55 min, t (minor) = 6.39 min]. [α]_D_^29^ = +67.3 (c = 1.0, CHCl_3_). ^1^H NMR (400 MHz, CDCl_3_) δ 7.39-7.23 (m, 4H), 7.22-7.17 (m, 1H), 7.16-7.07 (m, 3H), 7.07-6.99 (m, 1H), 5.79 (ddd, *J* = 16.8, 10.0, 9.2 Hz, 1H), 5.12 (d, *J* = 16.8 Hz, 1H), 5.09 (d, *J* = 10.0 Hz, 1H), 5.03 (dd, *J* = 9.2, 6.8 Hz, 1H), 3.88 (app-t, *J* = 8.8 Hz, 1H), 3.11-2.99 (m, 1H), 1.93-1.78 (m, 1H), 1.80-1.63 (m, 1H), 0.70 (t, *J* = 7.2 Hz, 3H). ^13^C NMR (100 MHz, CDCl_3_) δ 140.8, 138.9, 135.3, 131.3, 130.8, 130.3, 129.0, 128.6, 127.8, 126.6, 122.7, 118.7, 96.3, 51.9, 48.8, 23.4, 11.9. IR (thin film): ν_max_ (cm^-1^) = 3028, 2968, 2932, 2875, 1592, 1569, 1546, 1494, 1474, 1454, 1429, 1364, 1074, 994, 927, 781, 748, 699, 583, 551, 521, 437. HRMS (DART) calcd for C_19_H_21_NO_2_Br [M+H]^+^: 374.0750. Found: 374.0750.

10:1 dr, for major diastereomer, **4h**, light yellow oil, 72.8 mg, 67% yield, 99% ee. [Daicel Chiralcel OD-H (0.46 cm × 15 cm), *n*-hexane/2-propanol = 99/1, *v* = 1.0 mL·min^–1^, λ = 214 nm, t (major) = 3.35 min, t (minor) = 4.23 min]. [α]_D_^29^ = +57.1 (c = 1.0, CHCl_3_). ^1^H NMR (400 MHz, CDCl_3_) δ 7.49 (d, *J* = 7.6 Hz, 1H), 7.42-7.26 (m, 6H), 7.15-7.07 (m, 2H), 5.86 (ddd, *J* = 16.8, 10.0, 8.8 Hz, 1H), 5.17-5.05 (m, 3H), 3.96 (app-t, *J* = 8.8 Hz, 1H), 3.11-2.99 (m, 1H), 1.90-1.78 (m, 1H), 1.78-1.63 (m, 1H), 0.70 (t, *J* = 7.2 Hz, 3H). ^13^C NMR (100 MHz, CDCl_3_) δ 139.4, 138.8, 135.3, 131.5 (q, *J* = 1.4 Hz), 131.0 (q, *J* = 32.1 Hz), 129.2, 129.1, 128.6, 127.9, 125.3 (q, *J* = 3.8 Hz), 124.6 (q, *J* = 3.8 Hz), 122.7, 118.9, 96.3, 51.9, 48.8, 23.8, 11.8. ^19^F NMR (376 MHz, CDCl_3_) δ -62.6 (m). IR (thin film): ν_max_ (cm^-1^) = 3030, 2971, 2934, 2879, 1548, 1494, 1452, 1367, 1327, 1254, 1164, 1123, 1074, 990, 928, 799, 750, 701, 657, 578, 525. HRMS (DART) calcd for C_20_H_21_NO_2_F_3_ [M+H]^+^: 364.1519. Found: 364.1518.

10:1 dr, for major diastereomer, **4i**, light yellow oil, 58.1 mg, 63% yield, 98% ee. [Waters upc, SFC system, OD-3 (0.30 cm × 15 cm), CO_2_/MeOH = 98/2, *v* = 1.0 mL•min^-1^, λ = 214 nm, t (major) = 3.58 min, t (minor) = 4.24 min]. [α]_D_^26^ = +90.9 (c = 1.0, CHCl_3_). ^1^H NMR (400 MHz, CDCl_3_) δ 7.36-7.29 (m, 2H), 7.29-7.23 (m, 1H), 7.17-7.10 (m, 2H), 7.06 (d, *J* = 8.0 Hz, 2H), 7.01-6.94 (m, 2H), 5.82 (ddd, *J* = 16.8, 10.0, 9.2 Hz, 1H), 5.10 (d, *J* = 16.8 Hz, 1H), 5.032 (dd, *J* = 10.0, 1.2 Hz, 1H), 5.030 (dd, *J* = 9.2, 6.8 Hz, 1H), 3.90 (app-t, *J* = 9.2 Hz, 1H), 3.07 (ddd, *J* = 11.2, 6.8, 3.2 Hz, 1H), 2.27 (s, 3H), 1.96-1.82 (m, 1H), 1.82-1.65 (m, 1H), 0.70 (t, *J* = 7.2 Hz, 3H). ^13^C NMR (100 MHz, CDCl_3_) δ 139.3, 137.3, 136.4, 135.6, 129.5, 128.9, 128.6, 127.8, 127.6, 117.7, 96.7, 52.0, 48.9, 23.1, 21.1, 12.0. IR (thin film): ν_max_ (cm^-1^) = 3027, 2968, 2930, 2876, 1546, 1513, 1494, 1453, 1367, 991, 923, 815, 798, 749, 700, 651, 571, 521. HRMS (DART) calcd for C_20_H_24_NO_2_ [M+H]^+^: 310.1802. Found: 310.1801.

10:1 dr, for major diastereomer, **4j**, light yellow oil, 59.4 mg, 61% yield, >99% ee. [Daicel Chiralcel OD-H (0.46 cm × 25 cm), *n*-hexane/2-propanol = 99/1, *v* = 1.0 mL·min^–1^, λ = 214 nm, t (major) = 8.64 min, t (minor) = 12.13 min]. [α]_D_^27^ = +85.4 (c = 1.0, CHCl_3_). ^1^H NMR (400 MHz, CDCl_3_) δ 7.37-7.29 (m, 2H), 7.31-7.22 (m, 1H), 7.16-7.08 (m, 2H), 7.04-6.95 (m, 2H), 6.82-6.75 (m, 2H), 5.84 (ddd, *J* = 16.8, 10.0, 8.8 Hz, 1H), 5.09 (d, *J* = 16.8 Hz, 1H), 5.04 (d, *J* = 10.0 Hz, 1H), 5.01 (dd, *J* = 8.8 ,6.8 Hz, 1H), 3.87 (app-t, *J* = 8.8 Hz, 1H), 3.73 (s, 3H), 3.11-3.01 (m, 1H), 1.96-1.81 (m, 1H), 1.81-1.64 (m, 1H), 0.70 (t, *J* = 7.2 Hz, 3H). ^13^C NMR (100 MHz, CDCl_3_) δ 159.0, 139.3, 136.5, 130.5, 129.1, 128.9, 128.6, 127.6, 117.6, 114.2, 96.8, 55.2, 51.4, 48.8, 23.2, 12.0. IR (thin film): ν_max_ (cm^-1^) = 3030, 2967, 2934, 2876, 2837, 1610, 1546, 1511, 1455, 1367, 1304, 1247, 1180, 1032, 992, 924, 829, 802, 750, 701, 651, 576, 536. HRMS (DART) calcd for C_20_H_27_N_2_O_3_ [M+NH_4_]^+^: 343.2016. Found: 343.2016.

10:1 dr, for major diastereomer, **4k**, white solid, m.p. = 123.3–124.6 ^o^C, 74.4 mg, 67% yield, 97% ee. [Daicel Chiralcel IG (0.46 cm × 25 cm), *n*-hexane/2-propanol = 98/2, *v* = 1.0 mL·min^–1^, λ = 214 nm, t (major) = 8.01 min, t (minor) = 10.47 min]. [α]_D_^28^ = +99.6 (c = 1.0, CHCl_3_). ^1^H NMR (400 MHz, CDCl_3_) δ 7.56-7.50 (m, 2H), 7.50-7.45 (m, 2H), 7.43-7.36 (m, 2H), 7.36-7.24 (m, 4H), 7.18-7.11 (m, 4H), 5.87 (ddd, *J* = 16.8, 10.0, 9.2 Hz, 1H), 5.14 (d, *J* = 16.8 Hz, 1H), 5.11-5.04 (m, 2H), 3.98 (app-t, *J* = 9.2 Hz, 1H), 3.20-3.05 (m, 1H), 1.97-1.84 (m, 1H), 1.82-1.65 (m, 1H), 0.72 (t, *J* = 7.2 Hz, 3H). ^13^C NMR (100 MHz, CDCl_3_) δ 140.6, 140.5, 139.2, 137.6, 136.1, 129.0, 128.8, 128.6, 128.4, 127.7, 127.5, 127.4, 127.1, 118.1, 96.6, 52.0, 48.9, 23.2, 12.0. IR (thin film): ν_max_ (cm^-1^) = 3031, 2968, 2934, 2876, 1546, 1486, 1453, 1409, 1369, 1320, 1250, 1139, 1075, 993, 929, 834, 798, 762, 741, 723, 693, 659, 594, 574, 554, 534, 495. HRMS (DART) calcd for C_25_H_29_N_2_O_2_ [M+NH_4_]^+^: 389.2224. Found: 389.2224.

11:1 dr, for major diastereomer, **4l**, light yellow oil, 55.8 mg, 59% yield, 99% ee. [Daicel Chiralcel OD-H (0.46 cm × 25 cm), *n*-hexane/2-propanol = 99/1, *v* = 1.0 mL·min^–1^, λ = 214 nm, t (major) = 5.62 min, t (minor) = 12.37 min]. [α]_D_^28^ = +69.1 (c = 1.0, CHCl_3_). ^1^H NMR (400 MHz, CDCl_3_) δ 7.38-7.24 (m, 3H), 7.15-7.08 (m, 2H), 7.08-7.00 (m, 2H), 6.99-6.89 (m, 2H), 5.82 (ddd, *J* = 16.8, 10.4, 8.8 Hz, 1H), 5.09 (d, *J* = 16.8 Hz, 1H), 5.07 (d, *J* = 10.4 Hz, 1H), 5.02 (dd, *J* = 8.8, 7.2 Hz, 1H), 3.91 (app-t, *J* = 8.8 Hz, 1H), 3.12-3.00 (m, 1H), 1.92-1.80 (m, 1H), 1.80-1.63 (m, 1H), 0.70 (t, *J* = 7.2 Hz, 3H). ^13^C NMR (100 MHz, CDCl_3_) δ 162.2 (d, *J* = 244.9 Hz), 139.1, 135.9, 134.3 (d, *J* = 3.2 Hz), 129.7 (d, *J* = 8.0 Hz), 129.0, 128.6, 127.7, 118.1, 115.7 (d, *J* = 21.4 Hz), 96.6, 51.4, 48.8, 23.4, 11.9. ^19^F NMR (376 MHz, CDCl_3_) δ -114.6 (m). IR (thin film): ν_max_ (cm^-1^) = 3030, 2969, 2933, 2877, 1604, 1547, 1509, 1454, 1368, 1225, 1161, 992, 927, 833, 792, 750, 701, 650, 572, 529. HRMS (DART) calcd for C_19_H_21_NO_2_F [M+H]^+^: 314.1551. Found: 314.1550.

10:1 dr, for major diastereomer, **4m**, light yellow oil, 64.6 mg, 65% yield, 99% ee. [Daicel Chiralcel OD-H (0.46 cm × 25 cm), *n*-hexane/2-propanol = 99/1, *v* = 1.0 mL·min^–1^, λ = 214 nm, t (major) = 5.47 min, t (minor) = 8.46 min]. [α]_D_^28^ = +82.4 (c = 1.0, CHCl_3_). ^1^H NMR (400 MHz, CDCl_3_) δ 7.38-7.24 (m, 3H), 7.28-7.18 (m, 2H), 7.14-7.07 (m, 2H), 7.05-6.97 (m, 2H), 5.80 (ddd, *J* = 16.8, 10.0, 8.8 Hz, 1H), 5.09 (d, *J* = 16.8 Hz, 1H), 5.07 (d, *J* = 10.0 Hz, 1H), 5.02 (dd, *J* = 8.8, 7.2 Hz, 1H), 3.89 (app-t, *J* = 8.8 Hz, 1H), 3.10-3.00 (m, 1H), 1.92-1.78 (m, 1H), 1.79-1.63 (m, 1H), 0.70 (t, *J* = 7.2 Hz, 3H). ^13^C NMR (100 MHz, CDCl_3_) δ 139.0, 137.1, 135.7, 133.5, 129.5, 128.99, 128.95, 128.6, 127.7, 118.4, 96.4, 51.6, 48.8, 23.5, 11.9. IR (thin film): ν_max_ (cm^-1^) = 3029, 2969, 2932, 2876, 1546, 1491, 1454, 1409, 1365, 1091, 1014, 990, 927, 824, 797, 750, 700, 622, 520. HRMS (DART) calcd for C_19_H_24_N_2_O_2_Cl [M+NH_4_]^+^: 347.1521. Found: 347.1519.

11:1 dr, for major diastereomer, **4n**, light yellow oil, 71.2 mg, 63% yield, 99% ee. [Daicel Chiralcel OD-H (0.46 cm × 25 cm), *n*-hexane/2-propanol = 99/1, *v* = 1.0 mL·min^–1^, λ = 214 nm, t (major) = 5.65 min, t (minor) = 8.48 min]. [α]_D_^29^ = +77.3 (c = 1.0, CHCl_3_). ^1^H NMR (400 MHz, CDCl_3_) δ 7.41-7.24 (m, 5H), 7.11 (d, *J* = 6.8 Hz, 2H), 6.95 (d, *J* = 8.8 Hz, 2H), 5.79 (ddd, *J* = 16.8, 10.0, 8.8 Hz, 1H), 5.09 (d, *J* = 16.8 Hz, 1H), 5.07 (d, *J* = 10.0 Hz, 1H), 5.02 (dd, *J* = 8.8, 7.2 Hz, 1H), 3.88 (app-t, *J* = 8.8 Hz, 1H), 3.10-3.01 (m, 1H), 1.91-1.78 (m, 1H), 1.78-1.63 (m, 1H), 0.70 (t, *J* = 7.2 Hz, 3H). ^13^C NMR (100 MHz, CDCl_3_) δ 139.0, 137.6, 135.6, 131.9, 129.8, 129.0, 128.6, 127.7, 121.7, 118.4, 96.3, 51.6, 48.8, 23.4, 11.9. IR (thin film): ν_max_ (cm^-1^) = 3028, 2968, 2932, 2876, 1546, 1488, 1454, 1406, 1364, 1074, 1009, 927, 819, 796, 750, 720, 700, 614, 518. HRMS (DART) calcd for C_19_H_21_NO_2_^79^Br [M+H]^+^: 374.0750. Found: 374.0749.

12:1 dr, for major diastereomer, **4o**, light yellow oil, 73.3 mg, 67% yield, 99% ee. [Daicel Chiralcel OD-H (0.46 cm × 15 cm), *n*-hexane/2-propanol = 99/1, *v* = 1.0 mL·min^–1^, λ = 214 nm, t (major) = 3.22 min, t (minor) = 3.90 min]. [α]_D_^29^ = +61.5 (c = 1.0, CHCl_3_). ^1^H NMR (400 MHz, CDCl_3_) δ 7.51 (d, *J* = 8.0 Hz, 2H), 7.38-7.25 (m, 3H), 7.20 (d, *J* = 8.0 Hz, 2H), 7.16-7.09 (m, 2H), 5.82 (ddd, *J* = 16.8, 10.0, 8.8 Hz, 1H), 5.15-5.05 (m, 3H), 4.00 (app-t, *J* = 8.8 Hz, 1H), 3.13-3.03 (m, 1H), 1.93-1.80 (m, 1H), 1.80-1.65 (m, 1H), 0.71 (t, *J* = 7.2 Hz, 3H). ^13^C NMR (100 MHz, CDCl_3_) δ 142.7 (q, *J* = 1.4 Hz), 138.9, 135.2, 129.9 (q, *J* = 32.5 Hz), 129.0, 128.6, 128.5, 127.8, 125.7 (q, *J* = 3.7 Hz), 122.7, 118.9, 96.2, 52.1, 48.9, 23.5, 11.9. ^19^F NMR (376 MHz, CDCl_3_) δ -62.6 (m). IR (thin film): ν_max_ (cm^-1^) = 3030, 2971, 2935, 2879, 1619, 1547, 1495, 1454, 1416, 1368, 1324, 1165, 1121, 1067, 1018, 991, 929, 838, 800, 750, 700, 617, 598, 518. HRMS (DART) calcd for C_20_H_21_NO_2_F_3_ [M+H]^+^: 364.1519. Found: 364.1518.

8:1 dr, for major diastereomer, **4p**, light yellow oil, 66.7 mg, 66% yield, 99% ee. [Daicel Chiralcel OD-H (0.46 cm × 25 cm), *n*-hexane/2-propanol = 99/1, *v* = 1.0 mL·min^–1^, λ = 214 nm, t (major) = 12.72 min, t (minor) = 15.80 min]. [α]_D_^29^ = +80.9 (c = 1.0, CHCl_3_). ^1^H NMR (400 MHz, CDCl_3_) δ 7.36-7.29 (m, 2H), 7.29-7.23 (m, 1H), 7.15-7.09 (m, 2H), 6.68 (d, *J* = 8.0 Hz, 1H), 6.59 (d, *J* = 1.6 Hz, 1H), 6.54 (dd, *J* = 8.0, 2.0 Hz, 1H), 5.89 (s, 2H), 5.79 (app-dt, *J* = 16.8, 9.6 Hz, 1H), 5.10 (d, *J* = 16.8 Hz, 1H), 5.05 (d, *J* = 10.0 Hz, 1H), 4.98 (dd, *J* = 9.2, 6.8 Hz, 1H), 3.85 (app-t, *J* = 9.2 Hz, 1H), 3.12-3.02 (m, 1H), 1.94-1.81 (m, 1H), 1.81-1.65 (m, 1H), 0.71 (t, *J* = 7.2 Hz, 3H). ^13^C NMR (100 MHz, CDCl_3_) δ 147.9, 147.0, 139.2, 136.2, 132.4, 128.9, 128.6, 127.6, 121.3, 117.8, 108.5, 108.3, 101.2, 96.7, 52.0, 48.9, 23.1, 12.0. IR (thin film): ν_max_ (cm^-1^) = 2969, 2932, 2897, 2878, 1546, 1503, 1487, 1443, 1368, 1238, 1188, 1125, 1037, 992, 925, 808, 792, 749, 700, 677, 636, 578. HRMS (DART) calcd for C_20_H_25_N_2_O_4_ [M+H]^+^: 357.1809. Found: 357.1807.

10:1 dr, for major diastereomer, **4q**, light yellow oil, 70.8 mg, 65% yield, 99% ee. [Daicel Chiralcel OD-H (0.46 cm × 25 cm), *n*-hexane/2-propanol = 99/1, *v* = 1.0 mL·min^–1^, λ = 214 nm, t (major) = 5.62 min, t (minor) = 6.69 min]. [α]_D_^29^ = +70.9 (c = 1.0, CHCl_3_). ^1^H NMR (400 MHz, CDCl_3_) δ 7.40-7.26 (m, 3H), 7.21 (t, *J* = 2.0 Hz, 1H), 7.12-7.06 (m, 2H), 6.95 (d, *J* = 2.0 Hz, 2H), 5.76 (ddd, *J* = 16.8, 10.0, 8.8 Hz, 1H), 5.13 (d, *J* = 16.0 Hz, 1H), 5.12 (d, *J* = 10.8 Hz, 1H), 5.01 (dd, *J* = 8.8, 7.2 Hz, 1H), 3.86 (app-t, *J* = 8.8 Hz, 1H), 3.10-3.00 (m, 1H), 1.88-1.77 (m, 1H), 1.77-1.63 (m, 1H), 0.71 (t, *J* = 7.2 Hz, 3H). ^13^C NMR (100 MHz, CDCl_3_) δ 141.9, 138.6, 135.2, 134.6, 129.0, 128.6, 128.0, 127.9, 126.8, 119.3, 96.0, 51.7, 48.8, 23.6, 11.9. IR (thin film): ν_max_ (cm^-1^) = 3029, 2968, 2932, 2876, 1587, 1569, 1547, 1494, 1454, 1432, 1365, 1250, 1200, 1122, 1100, 989, 929, 856, 797, 744, 700, 682, 586, 560, 522, 432. HRMS (DART) calcd for C_19_H_23_N_2_O_2_Cl_2_ [M+NH_4_]^+^: 381.1131. Found: 381.1128.

11:1 dr, for major diastereomer, **4r**, light yellow solid, m.p. = 105.4–106.7 ^o^C, 74.6 mg, 72% yield, 98% ee. [Daicel Chiralcel OD-H (0.46 cm × 25 cm), *n*-hexane/2-propanol = 99/1, *v* = 1.0 mL·min^–1^, λ = 214 nm, t (major) = 7.00 min, t (minor) = 8.50 min]. [α]_D_^29^ = +95.9 (c = 1.0, CHCl_3_). ^1^H NMR (400 MHz, CDCl_3_) δ 7.82-7.71 (m, 3H), 7.52 (s, 1H), 7.48-7.40 (m, 2H), 7.39-7.33 (m, 2H), 7.32-7.28 (m, 1H), 7.25-7.21 (m, 1H), 7.20-7.11 (m, 2H), 5.94 (ddd, *J* = 16.8, 10.0, 8.8 Hz, 1H), 5.21-5.14 (m, 2H), 5.11 (d, *J* = 10.0 Hz, 1H), 4.11 (app-t, *J* = 8.8 Hz, 1H), 3.16-3.09 (m, 1H), 1.99-1.85 (m, 1H), 1.84-1.68 (m, 1H), 0.73 (t, *J* = 7.2 Hz, 3H). ^13^C NMR (100 MHz, CDCl_3_) δ 139.2, 136.05, 135.98, 133.4, 132.8, 128.9, 128.6, 128.5, 127.9, 127.70, 127.67, 127.2, 126.3, 126.1, 125.8, 118.2, 96.4, 52.4, 48.9, 23.2, 12.0. IR (thin film): ν_max_ (cm^-1^) = 3056, 3029, 2975, 2935, 2877, 1543, 1510, 1453, 1367, 1341, 1318, 1247, 1123, 987, 936, 896, 860, 824, 806, 785, 749, 696, 670, 614, 569, 525, 479. HRMS (DART) calcd for C_23_H_24_NO_2_ [M+H]^+^: 346.1802. Found: 346.1801.

9:1 dr, **4s**, light yellow oil, 57.1 mg, 64% combined yield, [α]_D_^29^ = +55.6 (c = 1.0, CHCl_3_). For major diastereomer: 99% ee. [Daicel Chiralcel OD-H (0.46 cm × 25 cm), *n*-hexane/2-propanol = 99/1, *v* = 1.0 mL·min^–1^, λ = 214 nm, t (major) = 40.72 min, t (minor) = 51.37 min]. Two diastereomers can not be separated, and the peaks of the minor one are marked with asterisk. ^1^H NMR (400 MHz, CDCl_3_) δ 8.59-8.42 (m, 1H), 8.28 (s, 1H), 7.48-7.39 (m, 1H), 7.39-7.25 (m, 3H), 7.20 (dd, *J* = 8.0, 4.8 Hz, 1H), 7.15-7.05 (m, 2H), 6.21* (ddd, *J* = 16.8, 10.0, 9.2 Hz, 1H), 5.86 (ddd, *J* = 16.8, 10.4, 8.4 Hz, 1H), 5.27* (dd, *J* = 10.0, 1.2 Hz, 1H), 5.16-5.05 (m, 3H), 5.04-4.93* (m, 2H), 3.89 (app-t, *J* = 8.0 Hz, 1H), 3.60* (dd, *J* = 9.2, 5.6 Hz, 1H), 3.12-2.97 (m, 1H), 1.92-1.75 (m, 1H), 1.78-1.61 (m, 1H), 0.70 (t, *J* = 7.2 Hz, 3H), 0.65* (t, *J* = 7.2 Hz, 3H). ^13^C NMR (100 MHz, CDCl_3_) δ 149.9, 149.2*, 149.0, 148.8*, 138.6, 138.1*, 135.5, 135.1, 134.8*, 134.0*, 132.8*, 129.1, 128.6, 128.5*, 127.8, 127.7*, 123.5, 120.7*, 119.0, 96.9*, 96.0, 49.3, 49.0*, 48.8, 48.7*, 25.4*, 24.0, 11.7, 11.5*. IR (thin film): ν_max_ (cm^-1^) = 3030, 2969, 2932, 2877, 1576, 1546, 1479, 1454, 1427, 1366, 1250, 1026, 992, 929, 795, 751, 701, 626, 594, 523. HRMS (ESI) calcd for C_18_H_21_N_2_O_2_ [M+H]^+^: 297.1598. Found: 297.1594.

9.6:1 dr, for major diastereomer, **4t**, light yellow oil, 57.2 mg, 63% yield, 99% ee. [Daicel Chiralcel OJ-H (0.46 cm × 25 cm), *n*-hexane/2-propanol = 80/20, *v* = 1.0 mL·min^–1^, λ = 214 nm, t (minor) = 9.46 min, t (major) = 10.56 min]. [α]_D_^30^ = +79.8 (c = 1.0, CHCl_3_). ^1^H NMR (400 MHz, CDCl_3_) δ 7.38-7.30 (m, 2H), 7.30-7.25 (m, 1H), 7.18 (dd, *J* = 5.2, 1.2 Hz, 1H), 7.15-7.09 (m, 2H), 6.90 (dd, *J* = 5.2, 3.6 Hz, 1H), 6.76 (dd, *J* = 3.6, 1.2 Hz, 1H), 5.87 (ddd, *J* = 16.8, 10.0, 8.8 Hz, 1H), 5.14 (d, *J* = 17.2 Hz, 1H), 5.13 (d, *J* = 9.6 Hz, 1H), 5.03-4.95 (m, 1H), 4.16 (app-t, *J* = 8.0 Hz, 1H), 3.18-3.08 (m, 1H), 1.89-1.77 (m, 1H), 1.77-1.64 (m, 1H), 0.69 (t, *J* = 7.2 Hz, 3H). ^13^C NMR (100 MHz, CDCl_3_) δ 140.3, 138.8, 135.8, 129.0, 128.6, 127.7, 126.9, 126.0, 125.0, 118.3, 96.9, 48.6, 47.0, 23.7, 11.9. IR (thin film): ν_max_ (cm^-1^) = 3029, 2968, 2932, 2876, 1547, 1494, 1453, 1363, 1321, 1246, 989, 927, 852, 749, 697, 569, 520. HRMS (DART) calcd for C_17_H_20_NO_2_S [M+H]^+^: 302.1209. Found: 302.1209.

11:1 dr, for major diastereomer, **5a**, light yellow oil, 51.3 mg, 52% yield, 98% ee. [Waters upc, SFC system, IG-3 (0.46 cm × 25 cm), CO_2_/MeOH = 95/5, *v* = 1.0 mL•min^-1^, λ = 214 nm, t (minor) = 8.83 min, t (major) = 9.22 min]. [α]_D_^30^ = +155.9 (c = 1.0, CHCl_3_). ^1^H NMR (400 MHz, CDCl_3_) δ 7.48-7.37 (m, 1H), 7.32-7.13 (m, 7H), 7.15-7.06 (m, 1H), 5.90 (app-dt, *J* = 16.8, 10.0 Hz, 1H), 5.28 (d, *J* = 16.8 Hz, 1H), 5.10 (d, *J* = 9.6 Hz, 1H), 5.10-4.97 (m, 1H), 4.18-4.00 (m, 1H), 3.97-3.75 (m, 1H), 2.15-1.95 (m, 1H), 1.92-1.75 (m, 1H), 0.77 (t, *J* = 7.2 Hz, 3H). ^13^C NMR (100 MHz, CDCl_3_) δ 139.1, 136.5, 135.5, 135.1, 130.1, 129.0, 128.7, 128.1, 127.7, 127.6, 127.4, 118.1, 95.1, 52.6, 43.7, 20.5, 11.9. IR (thin film): ν_max_ (cm^-1^) = 3065, 3031, 2970, 2934, 2878, 1547, 1493, 1475, 1455, 1440, 1366, 1034, 990, 925, 751, 700, 676, 596, 519, 469. HRMS (DART) calcd for C_19_H_21_NO_2_Cl [M+H]^+^: 330.1255. Found: 330.1255.

10:1 dr, for major diastereomer, **5b**, light yellow oil, 50.8 mg, 52% yield, >99% ee. [Daicel Chiralcel IG (0.46 cm × 25 cm), *n*-hexane/2-propanol = 99/1, *v* = 1.0 mL·min^–1^, λ = 214 nm, t (major) = 13.74 min, t (minor) = 17.24 min]. [α]_D_^30^ = +79.4 (c = 1.0, CHCl_3_). ^1^H NMR (400 MHz, CDCl_3_) δ 7.31-7.15 (m, 4H), 7.15-7.05 (m, 2H), 6.81 (ddd, *J* = 8.4, 2.8, 0.8 Hz, 1H), 6.73 (d, *J* = 7.6 Hz, 1H), 6.68-6.64 (m, 1H), 5.86 (ddd, *J* = 16.8, 10.0, 9.2 Hz, 1H), 5.12 (d, *J* = 16.8 Hz, 1H), 5.07-5.01 (m, 1H), 5.06 (d, *J* = 10.0 Hz, 1H), 3.93 (app-t, *J* = 9.2 Hz, 1H), 3.79 (s, 3H), 3.10-3.00 (m, 1H), 1.94-1.79 (m, 1H), 1.79-1.63 (m, 1H), 0.72 (t, *J* = 7.2 Hz, 3H). ^13^C NMR (100 MHz, CDCl_3_) δ 160.0, 140.9, 138.7, 136.2, 129.9, 128.8, 128.0, 127.7, 120.8, 117.9, 114.8, 112.6, 96.5, 55.3, 52.4, 48.9, 23.3, 12.0. IR (thin film): ν_max_ (cm^-1^) = 2967, 2934, 2876, 2836, 1600, 1585, 1546, 1489, 1454, 1435, 1368, 1320, 1293, 1261, 1156, 1042, 993, 925, 875, 777, 756, 698, 674, 520. HRMS (ESI) calcd for C_20_H_23_NO_3_Na [M+Na]^+^: 348.1570. Found: 348.1576.

8.6:1 dr, for major diastereomer, **5c**, light yellow oil, 60.3 mg, 64% yield, 99% ee. [Daicel Chiralcel OJ-H (0.46 cm × 25 cm), *n*-hexane/2-propanol = 80/20, *v* = 1.0 mL·min^–1^, λ = 214 nm, t (minor) = 9.77 min, t (major) = 20.41 min]. [α]_D_^30^ = +83.6 (c = 1.0, CHCl_3_). ^1^H NMR (400 MHz, CDCl_3_) δ 7.34-7.16 (m, 4H), 7.13-7.05 (m, 2H), 7.02-6.92 (m, 1H), 6.92 (d, *J* = 8.0 Hz, 1H), 6.89-6.81 (m, 1H), 5.83 (ddd, *J* = 16.8, 10.0, 9.2 Hz, 1H), 5.13 (d, *J* = 16.8 Hz, 1H), 5.07 (d, *J* = 10.0 Hz, 1H), 5.02 (dd, *J* = 9.6, 6.8 Hz, 1H), 3.94 (app-t, *J* = 9.2 Hz, 1H), 3.10 (ddd, *J* = 11.2, 6.8, 3.2 Hz, 1H), 1.98-1.83 (m, 1H), 1.78-1.65 (m, 1H), 0.72 (t, *J* = 7.2 Hz, 3H). ^13^C NMR (100 MHz, CDCl_3_) δ 163.2 (*J* = 245.1 Hz), 141.9 (*J* = 6.9 Hz), 138.6, 136.0, 130.5 (*J* = 8.3 Hz), 128.9, 127.9, 127.8, 124.3 (*J* = 2.9 Hz), 118.2, 115.6 (*J* = 21.4 Hz), 114.6 (*J* = 20.9 Hz), 96.3, 52.5, 48.7 (*J* = 1.7 Hz), 23.0, 12.0. ^19^F NMR (376 MHz, CDCl_3_) δ -112.4 (m). IR (thin film): ν_max_ (cm^-1^) = 3031, 2969, 2934, 2878, 1615, 1589, 1547, 1489, 1451, 1367, 1245, 1146, 991, 927, 872, 781, 757, 697, 520. HRMS (DART) calcd for C_19_H_21_NO_2_F [M+H]^+^: 314.1551. Found: 314.1550.

7.2:1 dr, for major diastereomer, **5d**, light yellow oil, 59.7 mg, 53% yield, 99% ee. [Daicel Chiralcel OJ-H (0.46 cm × 25 cm), *n*-hexane/2-propanol = 80/20, *v* = 1.0 mL·min^–1^, λ = 214 nm, t (minor) = 12.64 min, t (major) = 22.65 min]. [α]_D_^30^ = +81.3 (c = 1.0, CHCl_3_). ^1^H NMR (400 MHz, CDCl_3_) δ 7.41 (d, *J* = 7.6 Hz, 1H), 7.32-7.16 (m, 5H), 7.12-7.07 (m, 2H), 7.05 (d, *J* = 7.6 Hz, 1H), 5.82 (app-dt, *J* = 16.8, 10.0 Hz, 1H), 5.13 (d, *J* = 16.8 Hz, 1H), 5.08 (d, *J* = 10.0 Hz, 1H), 5.01 (dd, *J* = 9.2, 6.4 Hz, 1H), 3.93 (app-t, *J* = 9.2 Hz, 1H), 3.07 (ddd, *J* = 11.6, 6.4, 3.2 Hz, 1H), 1.96-1.82 (m, 1H), 1.78-1.64 (m, 1H), 0.72 (t, *J* = 7.2 Hz, 3H). ^13^C NMR (100 MHz, CDCl_3_) δ 141.7, 138.5, 135.9, 131.9, 130.8, 130.5, 128.9, 127.9, 127.8, 127.0, 122.9, 118.3, 96.2, 52.5, 48.7, 22.9, 12.0. IR (thin film): ν_max_ (cm^-1^) = 3030, 2968, 2932, 2876, 1593, 1546, 1493, 1454, 1426, 1367, 1319, 1248, 1072, 994, 926, 884, 754, 696, 664, 596, 519, 443. HRMS (DART) calcd for C_19_H_21_NO_2_^79^Br [M+H]^+^: 374.0750. Found: 374.0749.

12:1 dr, for major diastereomer, **5e**, light yellow solid, m.p. = 48.3–49.0 ^o^C, 59.4 mg, 64% yield, 98% ee. [Daicel Chiralcel OJ-H (0.46 cm × 25 cm), *n*-hexane/2-propanol = 80/20, *v* = 1.0 mL·min^–1^, λ = 214 nm, t (minor) = 8.68 min, t (major) = 13.45 min]. [α]_D_^30^ = +75.9 (c = 1.0, CHCl_3_). ^1^H NMR (400 MHz, CDCl_3_) δ 7.30-7.16 (m, 3H), 7.14 (d, *J* = 8.0 Hz, 2H), 7.12-7.05 (m, 2H), 7.01 (d, *J* = 8.0 Hz, 2H), 5.86 (ddd, *J* = 16.8, 10.0, 9.2 Hz, 1H), 5.13 (d, *J* = 16.8 Hz, 1H), 5.07 (d, *J* = 10.0 Hz, 1H), 5.03 (dd, *J* = 9.2, 6.8 Hz, 1H), 3.94 (app-t, *J* = 9.2 Hz, 1H), 3.04 (ddd, *J* = 11.6, 6.8, 3.2 Hz, 1H), 2.33 (s, 3H), 1.95-1.81 (m, 1H), 1.79-1.63 (m, 1H), 0.71 (t, *J* = 7.2 Hz, 3H). ^13^C NMR (100 MHz, CDCl_3_) δ 138.7, 137.2, 136.3, 136.2, 129.6, 128.8, 128.4, 128.0, 127.7, 117.9, 96.7, 52.2, 48.4, 23.0, 21.2, 12.1. IR (thin film): ν_max_ (cm^-1^) = 3028, 2972, 2922, 2877, 1545, 1514, 1492, 1453, 1373, 1319, 1250, 1078, 996, 941, 840, 818, 784, 756, 700, 675, 578, 519. HRMS (DART) calcd for C_20_H_24_NO_2_ [M+H]^+^: 310.1802. Found: 310.1801.

12:1 dr, for major diastereomer, **5f**, light yellow oil, 64.4 mg, 66% yield, 98% ee. [Daicel Chiralcel OD-H (0.46 cm × 25 cm), *n*-hexane/2-propanol = 99/1, *v* = 1.0 mL·min^–1^, λ = 214 nm, t (major) = 8.92 min, t (minor) = 10.02 min]. [α]_D_^30^ = +76.6 (c = 1.0, CHCl_3_). ^1^H NMR (400 MHz, CDCl_3_) δ 7.30-7.17 (m, 3H), 7.13-7.07 (m, 2H), 7.07-7.01 (m, 2H), 6.90-6.83 (m, 2H), 5.84 (ddd, *J* = 16.8, 10.0, 9.2 Hz, 1H), 5.12 (d, *J* = 16.8 Hz, 1H), 5.07 (d, *J* = 10.0 Hz, 1H), 5.01 (dd, *J* = 9.2, 6.8 Hz, 1H), 3.93 (app-t, *J* = 9.2 Hz, 1H), 3.79 (s, 3H), 3.04 (ddd, *J* = 11.6, 6.8, 3.2 Hz, 1H), 1.94-1.79 (m, 1H), 1.77-1.62 (m, 1H), 0.71 (t, *J* = 7.2 Hz, 3H). ^13^C NMR (100 MHz, CDCl_3_) δ 159.0, 138.8, 136.3, 131.1, 129.5, 128.8, 128.0, 127.6, 117.9, 114.3, 96.8, 55.3, 52.3, 48.1, 23.1, 12.0. IR (thin film): ν_max_ (cm^-1^) = 3031, 2966, 2933, 2876, 2837, 1611, 1546, 1512, 1456, 1368, 1302, 1249, 1180, 1127, 1031, 992, 925, 828, 794, 757, 700, 673, 572, 521. HRMS (ESI) calcd for C_20_H_23_NO_3_Na [M+Na]^+^: 348.1570. Found: 348.1573.

**5g**, white solid, m.p. = 66.0–67.1 °C, 59.2 mg, 63% yield, 12:1 dr. [α]_D_^30^ = +85.8 (c = 1.0, CHCl_3_). Two diastereomers can not be separated. For major diastereomer: 99% ee. [Daicel Chiralcel OJ-H (0.46 cm × 25 cm), *n*-hexane/2-propanol = 70/30, *v* = 1.0 mL·min^–1^, λ = 214 nm, t (minor) = 10.79 min, t (major) = 19.31 min]. ^1^H NMR (400 MHz, CDCl_3_) δ 7.31-7.20 (m, 3H), 7.14-7.06 (m, 4H), 7.06-7.00 (m, 2H), 5.83 (app-dt, *J* = 16.8, 9.6 Hz, 1H), 5.14 (d, *J* = 17.2 Hz, 1H), 5.09 (d, *J* = 10.0 Hz, 1H), 5.00 (dd, *J* = 9.6, 6.8 Hz, 1H), 3.94 (app-t, *J* = 9.6 Hz, 1H), 3.09 (ddd, *J* = 11.6, 6.8, 3.2 Hz, 1H), 1.96-1.85 (m, 1H), 1.78-1.64 (m, 1H), 0.71 (t, *J* = 7.2 Hz, 3H). ^13^C NMR (100 MHz, CDCl_3_) δ 162.2 (d, *J* = 244.6 Hz), 138.7, 136.0, 135.0 (d, *J* = 3.2 Hz), 130.1 (d, *J* = 7.9 Hz), 128.9, 127.9, 127.7, 118.2, 115.9 (d, *J* = 21.3 Hz), 96.6, 52.5, 48.2, 23.0, 12.0. ^19^F NMR (376 MHz, CDCl_3_) δ -114.8 (m). IR (thin film): ν_max_ (cm^-1^) = 3067, 3031, 2970, 2932, 2878, 1644, 1603, 1542, 1508, 1493, 1456, 1423, 1370, 1355, 1335, 1316, 1221, 1160, 1137, 1079, 992, 926, 845, 812, 791, 757, 717, 700, 676, 601, 577, 551, 520, 436. HRMS (DART) calcd for C_19_H_24_N_2_O_2_F [M+NH_4_]^+^: 331.1816. Found: 331.1815.

9.4:1 dr, for major diastereomer, **5h**, light yellow oil, 58.6 mg, 59% yield, 98% ee. [Daicel Chiralcel OJ-H (0.46 cm × 25 cm), *n*-hexane/2-propanol = 80/20, *v* = 1.0 mL·min^–1^, λ = 214 nm, t (minor) = 10.23 min, t (major) = 14.02 min]. [α]_D_^30^ = +76.3 (c = 1.0, CHCl_3_). ^1^H NMR (400 MHz, CDCl_3_) δ 7.34-7.29 (m, 2H), 7.29-7.20 (m, 3H), 7.12-7.02 (m, 4H), 5.83 (app-dt, *J* = 16.8, 9.6 Hz, 1H), 5.15 (d, *J* = 16.8 Hz, 1H), 5.10 (d, *J* = 10.0 Hz, 1H), 5.00 (dd, *J* = 9.6, 6.8 Hz, 1H), 3.93 (app-t, *J* = 9.2 Hz, 1H), 3.08 (ddd, *J* = 11.6, 6.8, 3.2 Hz, 1H), 1.97-1.83 (m, 1H), 1.78-1.63 (m, 1H), 0.71 (t, *J* = 7.2 Hz, 3H). ^13^C NMR (100 MHz, CDCl_3_) δ 138.6, 137.8, 136.0, 133.4, 129.9, 129.1, 128.9, 127.9, 127.8, 118.3, 96.4, 52.4, 48.3, 22.9, 12.0. IR (thin film): ν_max_ (cm^-1^) = 3030, 2969, 2932, 2877, 1546, 1492, 1454, 1411, 1367, 1319, 1248, 1092, 1013, 991, 925, 822, 790, 752, 700, 674, 577, 518. HRMS (DART) calcd for C_19_H_24_N_2_O_2_Cl [M+NH_4_]^+^: 347.1521. Found: 347.1518.

10:1 dr, for major diastereomer, **5i**, light yellow oil, 68.3 mg, 61% yield, 99% ee. [Daicel Chiralcel OJ-H (0.46 cm × 25 cm), *n*-hexane/2-propanol = 70/30, *v* = 1.0 mL·min^–1^, λ = 214 nm, t (minor) = 10.49 min, t (major) = 14.00 min]. [α]_D_^30^ = +70.7 (c = 1.0, CHCl_3_). ^1^H NMR (400 MHz, CDCl_3_) δ 7.48-7.42 (m, 2H), 7.30-7.17 (m, 3H), 7.12-7.06 (m, 2H), 7.04-6.96 (m, 2H), 5.83 (ddd, *J* = 16.8, 10.0, 9.2 Hz, 1H), 5.14 (d, *J* = 16.8 Hz, 1H), 5.08 (d, *J* = 10.0 Hz, 1H), 5.00 (dd, *J* = 9.2, 6.4 Hz, 1H), 3.93 (app-t, *J* = 9.2 Hz, 1H), 3.07 (ddd, *J* = 11.6, 6.4, 3.2 Hz, 1H), 1.98-1.82 (m, 1H), 1.78-1.61 (m, 1H), 0.70 (t, *J* = 7.2 Hz, 3H). ^13^C NMR (100 MHz, CDCl_3_) δ 138.5, 138.4, 135.9, 132.1, 130.3, 128.9, 127.9, 127.8, 121.5, 118.3, 96.3, 52.4, 48.4, 22.8, 12.0. IR (thin film): ν_max_ (cm^-1^) = 3030, 2968, 2932, 2876, 1546, 1488, 1454, 1407, 1367, 1319, 1248, 1073, 1009, 991, 925, 819, 788, 747, 699, 673, 576, 517. HRMS (DART) calcd for C_19_H_21_NO_2_^79^Br [M+H]^+^: 374.0750. Found: 374.0750.

11:1 dr, for major diastereomer, **5j**, light yellow oil, 77.1 mg, 61% yield, 99% ee. [Daicel Chiralcel OJ-H (0.46 cm × 25 cm), *n*-hexane/2-propanol = 70/30, *v* = 1.0 mL·min^–1^, λ = 214 nm, t (minor) = 11.66 min, t (major) = 14.25 min]. [α]_D_^30^ = +60.3 (c = 1.0, CHCl_3_). ^1^H NMR (400 MHz, CDCl_3_) δ 7.65 (d, *J* = 8.4 Hz, 2H), 7.30-7.17 (m, 3H), 7.13-7.05 (m, 2H), 6.87 (d, *J* = 8.4 Hz, 2H), 5.83 (app-dt, *J* = 16.8, 9.6 Hz, 1H), 5.14 (d, *J* = 16.8 Hz, 1H), 5.09 (d, *J* = 10.0 Hz, 1H), 4.99 (dd, *J* = 9.6, 6.8 Hz, 1H), 3.93 (app-t, *J* = 9.6 Hz, 1H), 3.05 (ddd, *J* = 11.6, 6.8, 3.2 Hz, 1H), 1.97-1.82 (m, 1H), 1.78-1.60 (m, 1H), 0.70 (t, *J* = 7.2 Hz, 3H). ^13^C NMR (100 MHz, CDCl_3_) δ 139.0, 138.5, 138.0, 135.9, 130.5, 128.9, 127.9, 127.8, 118.3, 96.2, 93.1, 52.4, 48.5, 22.8, 12.0. IR (thin film): ν_max_ (cm^-1^) = 3029, 2967, 2931, 2875, 1545, 1485, 1454, 1403, 1367, 1319, 1063, 1004, 925, 816, 787, 758, 745, 699, 673, 576, 517. HRMS (DART) calcd for C_19_H_21_NO_2_I [M+H]^+^: 422.0611. Found: 422.0612.

8.0:1 dr, for major diastereomer, **5k**, light yellow solid, m.p. = 69.7–71.5 °C, 73.2 mg, 63% yield, 98% ee. [Waters upc, SFC system, IG-3 (0.46 cm × 25 cm), CO_2_/MeOH = 90/10, *v* = 1.0 mL•min^-1^, λ = 214 nm, t (minor) = 13.96 min, t (major) = 15.11 min]. [α]_D_^31^ = +56.4 (c = 1.0, CHCl_3_). ^1^H NMR (400 MHz, CDCl_3_) δ 7.30-7.18 (m, 3H), 7.14-7.08 (m, 2H), 6.31 (s, 2H), 5.89 (ddd, *J* = 16.8, 10.0, 9.2 Hz, 1H), 5.13 (d, *J* = 16.8 Hz, 1H), 5.09 (d, *J* = 9.2 Hz, 1H), 5.03 (dd, *J* = 8.8, 6.8 Hz, 1H), 3.92 (app-t, *J* = 9.2 Hz, 1H), 3.85 (s, 3H), 3.83 (s, 6H), 3.04-2.94 (m, 1H), 1.95-1.81 (m, 1H), 1.77-1.60 (m, 1H), 0.75 (t, *J* = 7.2 Hz, 3H). ^13^C NMR (100 MHz, CDCl_3_) δ 153.5, 138.6, 137.5, 136.0, 134.7, 128.7, 128.0, 127.6, 117.9, 105.5, 96.5, 61.0, 56.3, 52.4, 49.2, 23.5, 12.0. IR (thin film): ν_max_ (cm^-1^) = 2963, 2937, 2877, 2840, 1588, 1545, 1506, 1453, 1421, 1356, 1325, 1238, 1184, 1126, 1005, 987, 936, 912, 852, 791, 758, 740, 702, 658, 615, 518. HRMS (ESI) calcd for C_22_H_27_NO_5_Na [M+Na]^+^: 408.1781. Found: 408.1789.

10:1 dr, for major diastereomer, **5l**, white solid, m.p. = 97.4–99.1 °C, 71.5 mg, 69% yield, 99% ee. [Daicel Chiralcel OJ-H (0.46 cm × 25 cm), *n*-hexane/2-propanol = 80/20, *v* = 1.0 mL·min^–1^, λ = 214 nm, t (major) = 15.99 min, t (major) = 19.34 min]. [α]_D_^30^ = +89.2 (c = 1.0, CHCl_3_). ^1^H NMR (400 MHz, CDCl_3_) δ 7.87-7.74 (m, 3H), 7.59 (s, 1H), 7.51-7.42 (m, 2H), 7.29-7.16 (m, 4H), 7.13-7.04 (m, 2H), 5.85 (ddd, *J* = 16.8, 10.0, 9.2 Hz, 1H), 5.17 (dd, *J* = 8.8, 6.8 Hz, 1H), 5.11 (d, *J* = 16.8 Hz, 1H), 5.02 (d, *J* = 10.0 Hz, 1H), 3.97 (app-t, *J* = 9.2 Hz, 1H), 3.32-3.22 (m, 1H), 2.02-1.91 (m, 1H), 1.91-1.77 (m, 1H), 0.72 (t, *J* = 7.2 Hz, 3H). ^13^C NMR (100 MHz, CDCl_3_) δ 138.6, 136.7, 136.2, 133.6, 132.9, 128.79, 128.76, 128.02, 127.96, 127.9, 127.8, 127.7, 126.4, 126.1, 118.0, 96.5, 52.5, 49.0, 23.2, 12.1. IR (thin film): ν_max_ (cm^-1^) = 2975, 2931, 2874, 1549, 1454, 1417, 1370, 1316, 1251, 991, 932, 907, 866, 825, 792, 750, 695, 674, 657, 582, 517, 481. HRMS (DART) calcd for C_23_H_24_NO_2_ [M+H]^+^: 346.1802. Found: 346.1802.

9:1 dr, for major diastereomer, **5m**, light yellow oil, 53.5 mg, 59% yield, 99% ee. [Daicel Chiralcel OJ-H (0.46 cm × 25 cm), *n*-hexane/2-propanol = 80/20, *v* = 1.0 mL·min^–1^, λ = 214 nm, t (minor) = 12.91 min, t (major) = 19.61 min]. [α]_D_^31^ = +70.7 (c = 1.0, CHCl_3_). ^1^H NMR (400 MHz, CDCl_3_) δ 7.33-7.18 (m, 4H), 7.17-7.10 (m, 2H), 6.97 (dd, *J* = 5.2, 3.6 Hz, 1H), 6.85 (d, *J* = 3.6 Hz, 1H), 5.90 (app-dt, *J* = 16.8, 9.6 Hz, 1H), 5.15 (d, *J* = 16.8 Hz, 1H), 5.09 (d, *J* = 10.0 Hz, 1H), 5.04 (dd, *J* = 9.2, 6.8 Hz, 1H), 3.97 (app-t, *J* = 9.2 Hz, 1H), 3.40 (ddd, *J* = 11.2, 6.8, 3.2 Hz, 1H), 2.06-1.88 (m, 1H), 1.77-1.60 (m, 1H), 0.82 (t, *J* = 7.2 Hz, 3H). ^13^C NMR (100 MHz, CDCl_3_) δ 142.3, 138.5, 135.8, 128.9, 128.0, 127.8, 127.2, 126.1, 124.6, 118.0, 96.8, 52.2, 44.3, 24.7, 12.1. IR (thin film): ν_max_ (cm^-1^) = 2973, 2934, 2876, 1546, 1491, 1454, 1418, 1366, 1319, 1244, 1137, 995, 931, 848, 791, 755, 696, 596, 553, 519, 505. HRMS (DART) calcd for C_17_H_20_NO_2_S [M+H]^+^: 302.1209. Found: 302.1209.

10:1 dr, for major diastereomer, **5n**, white solid, m.p. = 117.1–118.2 °C, 49.1 mg, 58% yield, 99% ee. [Daicel Chiralcel IG (0.46 cm × 25 cm), *n*-hexane/2-propanol = 99/1, *v* = 1.0 mL·min^–1^, λ = 214 nm, t (major) = 7.37 min, t (minor) = 10.52 min]. [α]_D_^29^ = +126.9 (c = 1.0, CHCl_3_). ^1^H NMR (400 MHz, CDCl_3_) δ 7.36-7.29 (m, 2H), 7.29-7.23 (m, 3H), 7.23-7.16 (m, 3H), 7.16-7.10 (m, 2H), 5.87 (app-dt, *J* = 16.8, 9.6 Hz, 1H), 5.20 (d, *J* = 16.8 Hz, 1H), 5.11 (d, *J* = 10.4 Hz, 1H), 5.06 (dd, *J* = 10.0, 6.0 Hz, 1H), 3.97 (app-t, *J* = 9.6 Hz, 1H), 3.46-3.33 (m, 1H), 1.43 (d, *J* = 7.2 Hz, 3H). ^13^C NMR (100 MHz, CDCl_3_) δ 141.6, 138.8, 136.0, 129.0, 128.9, 127.8, 127.7, 127.63, 127.59, 118.3, 96.7, 52.3, 41.2, 15.4. IR (thin film): ν_max_ (cm^-1^) = 2990, 2975, 2945, 2902, 1544, 1492, 1452, 1368, 1332, 1278, 1269, 1073, 993, 927, 788, 774, 752, 696, 672, 603, 560, 520. HRMS (DART) calcd for C_18_H_20_NO_2_ [M+H]^+^: 282.1489. Found: 282.1488.

# 5. General procedure for the stereodivergent synthesis

Under Ar atmosphere, Cu(OTf)_2_ (5.4 mg, 0.015 mmol, 5 mol%) and **L1** (10.7 mg, 0.015 mmol, 5 mol%) were added to an oven-dried Schlenk tube. The reaction tube was evacuated and refilled with argon three times. Freshly distilled toluene (6.0 mL) was added to the flask. After the mixture was stirred for 30 min, **1a** (44.7 mg, 0.30 mmol, 1.0 equiv) and **2a** (0.36 mL, 0.36 mmol, 1.2 equiv) were successively added to the flask. The reaction was stirred at rt for 12 h, **K1** (12.3 mg, 0.012 mmol, 4 mol%), LiO*^t^*Bu (48.0 mg, 0.6 mmol, 2.0 equiv) and **3a** (69.2 mg, 0.36 mmol, 1.2 equiv) were then added to the flask. The reaction was stirred at 50 ºC for 12 h and quenched with saturated NH_4_Cl solution (15 mL), and then the mixture was extracted with EtOAc (15 mL x 3). The combined organic fractions were washed with brine (15 mL), dried over Na_2_SO_4_, filtered, and concentrated by rotary evaporation. The diastereomeric ratio was determined by ^1^H NMR of the crude reaction mixture. Then the residue was purified by silica gel column chromatography (PE/EtOAc = 100/1-40/1) or PTLC (PE/EtOAc = 40/1) to afford the desired product **4a** or **4a′**.

1:6.3:1 dr, for major diastereomer, (1*R*,2*S*,3*S*)-**4a′**, light yellow solid, m.p. = 77.6–79.6 °C, 49.0 mg, 55% yield, 99% ee. [Daicel Chiralcel OJ-H (0.46 cm × 25 cm), *n*-hexane/2-propanol = 90/10, *v* = 1.0 mL·min^–1^, λ = 214 nm, t (minor) = 5.63 min, t (major) = 6.75 min]. [α]_D_^30^ = -1.72 (c = 1.0, CHCl_3_). ^1^H NMR (400 MHz, CDCl_3_) δ 7.34-7.19 (m, 6H), 7.11-7.02 (m, 4H), 6.24 (ddd, *J* = 16.8, 10.0, 9.2 Hz, 1H), 5.22 (dd, *J* = 10.0, 1.6 Hz, 1H), 5.01-4.95 (m, 1H), 4.96 (d, *J* = 16.8, 1H), 3.61 (dd, *J* = 9.2, 6.4 Hz, 1H), 2.98 (ddd, *J* = 11.2, 9.2, 3.6 Hz, 1H), 1.83-1.59 (m, 2H), 0.63 (t, *J* = 7.2 Hz, 3H). ^13^C NMR (100 MHz, CDCl_3_) δ 139.3, 138.8, 134.1, 129.1, 129.0, 128.5, 127.62, 127.59, 127.58, 119.7, 97.6, 51.0, 48.6, 24.5, 11.8. IR (thin film): ν_max_ (cm^-1^) = 3030, 2965, 2926, 2874, 1545, 1492, 1452, 1417, 1368, 1316, 1263, 1142, 1078, 1028, 1000, 932, 834, 768, 749, 699, 609, 588, 557, 519, 473. HRMS (DART) calcd for C_19_H_22_NO_2_ [M+H]^+^: 296.1645. Found: 296.1644.

12:1 dr, for major diastereomer, (1*R*,2*S*,3*R*)-**4a**, light yellow solid, 55.0 mg, 62% yield, 99% ee. [Daicel Chiralcel IG (0.46 cm × 25 cm), *n*-hexane/2-propanol = 99/1, *v* = 1.0 mL·min^–1^, λ = 214 nm, t (minor) = 6.22 min, t (major) = 7.49 min]. [α]_D_^30^ = -87.0 (c = 1.0, CHCl_3_). Spectral data were in agreement with those of the enantiomer reported above.

1:6.5:1 dr, for major diastereomer, (1*S*,2*R*,3*R*)-**4a′**, light yellow solid, 51.5 mg, 58% yield, 99% ee. [Daicel Chiralcel OJ-H (0.46 cm × 25 cm), *n*-hexane/2-propanol = 90/10, *v* = 1.0 mL·min^–1^, λ = 214 nm, t (major) = 5.61 min, t (minor) = 6.76 min]. [α]_D_^31^ = +0.81 (c = 1.0, CHCl_3_). Spectral data were in agreement with those of the enantiomer reported above.

1:6.3:1 dr, for minor diastereomer, **4a′′**, light yellow oil, 8.1 mg, 9% yield. ^1^H NMR (400 MHz, CDCl_3_) δ 7.34-7.28 (m, 3H), 7.28-7.23 (m, 2H), 7.22-7.18 (m, 1H), 7.17-7.11 (m, 4H), 5.99 (app-dt, *J* = 17.2, 9.6 Hz, 1H), 5.28 (d, *J* = 10.0 Hz, 1H), 5.26 (d, *J* = 17.2 Hz, 1H), 5.17 (dd, *J* = 10.8, 4.8 Hz, 1H), 3.79 (app-t, *J* = 9.6 Hz, 1H), 3.22-3.13 (m, 1H), 2.09-1.96 (m, 1H), 1.91-1.77 (m, 1H), 0.85 (t, *J* = 7.2 Hz, 3H). ^13^C NMR (100 MHz, CDCl_3_) δ 139.5, 137.8, 136.8, 129.0, 128.9, 128.4, 127.8, 127.7, 127.5, 118.9, 94.4, 51.5, 48.4, 26.2, 12.4. IR (thin film): ν_max_ (cm^-1^) = 3084, 3063, 3031, 2966, 2932, 2875, 1547, 1493, 1453, 1364, 992, 926, 748, 699, 673, 515. HRMS (DART) calcd for C_19_H_22_NO_2_ [M+H]^+^: 296.1645. Found: 296.1644.

# 6. Gram-scale reaction and product transformations

(a) Gram-scale reaction

Under Ar atmosphere, Cu(OTf)_2_ (108.5 mg, 0.3 mmol, 5 mol%) and (*S*,*S*)-**L1** (213.6 mg, 0.6 mmol, 5 mol%) were added to an oven-dried Schlenk tube. The reaction tube was evacuated and refilled with argon three times. Freshly distilled toluene (120.0 mL) was added to the flask. After the mixture was stirred for 30 min, **1a** (894.9 mg, 6.0 mmol, 1.0 equiv) and **2a** (7.2 mL, 7.2 mmol, 1.2 equiv) were successively added to the flask. The reaction was stirred at rt for 12 h, (*S*,*S*,*S_a_*)-**K1** (247.0 mg, 0.24 mmol, 4 mol%), LiO*^t^*Bu (960.6 mg, 12.0 mmol, 2.0 equiv) and **3a** (1.38 g, 7.2 mmol, 1.2 equiv) were then added to the flask. The reaction was stirred at 50 ºC for 12 h and quenched with saturated NH_4_Cl solution (100 mL), and then the mixture was extracted with EtOAc (100 mL x 3). The combined organic fractions were washed with brine (100 mL), dried over Na_2_SO_4_, filtered, and concentrated by rotary evaporation. The diastereomeric ratio was determined by ^1^H NMR of the crude reaction mixture. Then the residue was purified by silica gel column chromatography (PE/EtOAc = 100/1-40/1) to afford the desired product **4a**.

(b) Product transformations

Under Ar atmosphere, Pd_2_dba_3_ (5.5 mg, 0.006 mmol, 3 mol%), PhN_2_BF_4_ (76.8 mg, 0.4 mmol, 2 equiv) were added to an oven-dried Schlenk tube. The reaction tube was evacuated and refilled with argon three times. DMA (2.0 mL) was added to the flask, then **4a** (59.1 mg, 0.2 mmol, 1.0 equiv) was added to the flask. The reaction was stirred at 50 °C for 12 h. After completion (monitored by TLC), the mixture was quenched with H_2_O (10 mL) and extracted with EtOAc (10 mL x 3). The combined organic fractions were washed with brine (10 mL), dried over Na_2_SO_4_, filtered, and concentrated by rotary evaporation. The diastereomeric ratio was determined by ^1^H NMR of the crude reaction mixture. Then the residue was purified by silica gel column chromatography (PE/EtOAc = 100/1 to 40/1) to afford the desired product **6**.

>20:1 dr, for major diastereomer, **6**, white solid, m.p. = 91.2–92.4 °C, 49.7 mg, 67% yield, 97% ee. [Daicel Chiralcel IG (0.46 cm × 25 cm), *n*-hexane/2-propanol = 99/1, *v* = 1.0 mL·min^–1^, λ = 214 nm, t (major) = 9.12 min, t (minor) = 10.34 min]. [α]_D_^30^ = +15.4 (c = 1.0, CHCl_3_). ^1^H NMR (400 MHz, CDCl_3_) δ 7.33-7.23 (m, 5H), 7.23-7.17 (m, 4H), 7.17-7.10 (m, 6H), 6.38 (d, *J* = 15.6 Hz, 1H), 6.00 (dd, *J* = 15.6, 9.2 Hz, 1H), 5.17 (dd, *J* = 9.2, 7.6 Hz, 1H), 4.10 (app-t, *J* = 9.2 Hz, 1H), 3.16-3.05 (m, 1H), 1.93-1.79 (m, 1H), 1.79-1.64 (m, 1H), 0.68 (t, *J* = 7.2 Hz, 3H). ^13^C NMR (100 MHz, CDCl_3_) δ 139.3, 139.0, 136.6, 132.6, 129.0, 128.9, 128.8, 128.5, 127.89, 127.86, 127.71, 127.68, 127.5, 126.5, 97.0, 52.5, 49.5, 24.2, 11.9. IR (thin film): ν_max_ (cm^-1^) = 3058, 3026, 2961, 2928, 2872, 1548, 1492, 1452, 1371, 1263, 1113, 1070, 1028, 955, 773, 736, 693, 620, 551, 523, 491. HRMS (DART) calcd for C_25_H_29_N_2_O_2_ [M+NH_4_]^+^: 389.2224. Found: 389.2223.

Pd/C (20.0 mg, palladium on activated carbon, 10% Pd basis, 0.1 equiv) was added to a solution of **4a** (59.1 mg, 0.2 mmol, 1.0 equiv) in EtOAc (2.0 mL) under argon. Then the reaction mixture was exchanged with H_2_ atmosphere (1 atm) and stirred at rt for 24 h. After completion (monitored by TLC), the crude mixture was filtered with a pad of celite and washed with EtOAc (10 mL x 3). The filtrate was concentrated by rotary evaporation. The diastereomeric ratio was determined by ^1^H NMR of the crude reaction mixture. Then the residue was purified by silica gel column chromatography (PE/EtOAc = 50/1-20/1) to afford the desired product **7**.

>20:1 dr, for major diastereomer, **7**, white solid, m.p. = 67.0–68.6 °C, 57.8 mg, 97% yield, 99% ee. [Waters upc, SFC system, IE-3 (0.30 cm × 15 cm), CO_2_/MeOH = 98/2, *v* = 1.0 mL•min^-1^, λ = 214 nm, t (major) = 3.07 min, t (minor) = 3.77 min]. [α]_D_^30^ = -9.8 (c = 1.0, CHCl_3_). ^1^H NMR (400 MHz, CDCl_3_) δ 7.36-7.25 (m, 2H), 7.27-7.18 (m, 1H), 7.21-7.13 (m, 3H), 7.06 (d, *J* = 7.2 Hz, 2H), 6.91-6.83 (m, 2H), 4.96 (dd, *J* = 9.6, 6.0 Hz, 1H), 2.89 (td, *J* = 10.4, 3.6 Hz, 1H), 2.81 (dt, *J* = 10.0, 5.6 Hz, 1H), 1.79-1.47 (m, 4H), 0.62 (t, *J* = 7.2 Hz, 3H), 0.58 (t, *J* = 7.2 Hz, 3H). ^13^C NMR (100 MHz, CDCl_3_) δ 139.4, 138.2, 129.0, 128.9, 128.7, 128.3, 127.59, 127.56, 96.6, 48.5, 48.4, 25.7, 25.4, 12.2, 11.6. IR (thin film): ν_max_ (cm^-1^) = 2964, 2928, 2873, 1543, 1494, 1453, 1379, 1314, 1259, 1084, 1027, 798, 767, 749, 701, 572, 549, 524. HRMS (DART) calcd for C_19_H_27_N_2_O_2_ [M+NH_4_]^+^: 315.2067. Found: 315.2065.

Under Ar atmosphere, [Ir(cod)Cl]_2_ (4.0 mg, 0.006 mmol, 3 mol %) and dppm (4.6 mg, 0.012 mmol, 6 mol %) were added to an oven-dried Schlenk tube. The reaction tube was evacuated and refilled with argon three times. Freshly distilled DCM (2.0 mL) was added to the flask, then **4a** (59.1 mg, 0.2 mmol, 1.0 equiv) and HBpin (51.2 mg, 0.4 mmol, 2.0 equiv) were successively added to the flask. The reaction was stirred at rt for 12 h. After completion (monitored by TLC), the crude mixture was filtered with a pad of celite and washed with EtOAc (10 mL x 3). The filtrate was concentrated by rotary evaporation. The diastereomeric ratio was determined by ^1^H NMR of the crude reaction mixture. Then the residue was purified by silica gel column chromatography (PE/EtOAc = 20/1-10/1) to afford the desired product **8**.

>20:1 dr, for major diastereomer, **8**, white solid, m.p. = 68.8–70.7 °C, 67.3 mg, 79% yield, 97% ee. [Waters upc, SFC system, IE-3 (0.46 cm × 25 cm), CO_2_/MeOH = 95/5, *v* = 1.0 mL•min^-1^, λ = 214 nm, t (major) = 9.30 min, t (minor) = 10.34 min]. [α]_D_^30^ = -1.4 (c = 1.0, CHCl_3_). ^1^H NMR (400 MHz, CDCl_3_) δ 7.40-7.33 (m, 2H), 7.32-7.26 (m, 1H), 7.25-7.20 (m, 3H), 7.16-7.10 (m, 2H), 6.97-6.90 (m, 2H), 5.03 (dd, *J* = 9.2, 6.0 Hz, 1H), 3.07-2.92 (m, 2H), 1.93-1.82 (m, 1H), 1.81-1.68 (m, 2H), 1.67-1.56 (m, 1H), 1.16 (s, 12H), 0.66 (t, *J* = 7.2 Hz, 3H), 0.53 (t, *J* = 7.6 Hz, 2H). ^13^C NMR (100 MHz, CDCl_3_) δ 139.4, 138.1, 129.00, 128.97, 128.7, 128.2, 127.52, 127.46, 96.7, 83.1, 48.4, 48.3, 26.9, 25.0, 24.9, 24.8, 11.7. IR (thin film): ν_max_ (cm^-1^) = 2970, 2936, 2897, 2874, 1545, 1495, 1454, 1413, 1380, 1309, 1233, 1165, 1139, 1080, 966, 866, 839, 758, 740, 699, 671, 572, 539, 516. HRMS (ESI) calcd for C_25_H_34_BNO_4_Na [M+Na]^+^: 446.2473. Found: 446.2479.

Zn powder (140.6 mg, 2.15 mmol, 21.5 eq) was slowly added to a solution of **4a** (41.3 mg, 0.1 mmol, 1.0 equiv) in CH_3_COOH (2.5 mL) at rt. The reaction was stirred at rt for 3.5 h. After completion (monitored by TLC), the crude mixture was filtered with a pad of celite and washed with EtOAc. Then the filtrate was washed with saturated NaHCO_3_ solution, and extracted with EtOAc (15 mL x 3). The combined organic fractions were dried over Na_2_SO_4_, filtered and concentrated by rotary evaporation. The diastereomeric ratio was determined by ^1^H NMR of the crude reaction mixture. Then the residue was purified by silica gel column chromatography (PE/EtOAc = 1/1) to afford the desired product **9**.

>20:1 dr, for major diastereomer, **9**, yellow solid, m.p. = 87.6–88.1 °C, 46.6 mg, 88% yield, >99% ee. [Waters upc, SFC system, OD-3 (0.46 cm × 25 cm), CO_2_/MeOH = 90/10, *v* = 1.0 mL•min^-1^, λ = 214 nm, t (major) = 6.93 min, t (minor) = 8.09 min]. [α]_D_^30^ = +64.5 (c = 1.0, CHCl_3_). ^1^H NMR (400 MHz, CDCl_3_) δ 7.37-7.15 (m, 10H), 6.17-6.04 (m, 1H), 5.104 (d, *J* = 10.0 Hz, 1H), 5.096 (d, *J* = 17.2 Hz, 1H), 3.34 (app-t, *J* = 8.0 Hz, 1H), 3.23 (dd, *J* = 8.0, 4.8 Hz, 1H), 2.68 (dt, *J* = 11.2, 4.0 Hz, 1H), 1.93-1.68 (m, 2H), 1.07 (s, 2H), 0.73 (t, *J* = 7.2 Hz, 3H). ^13^C NMR (100 MHz, CDCl_3_) δ 143.8, 141.8, 140.5, 128.72, 128.69, 128.65, 128.5, 126.7, 126.3, 115.8, 60.2, 54.7, 50.0, 20.4, 12.3. IR (thin film): ν_max_ (cm^-1^) = 3026, 2979, 2937, 2877, 2834, 1638, 1595, 1491, 1452, 1380, 995, 909, 758, 734, 698, 535, 517. HRMS (ESI) calcd for C_19_H_24_N [M+H]^+^: 266.1903. Found: 266.1903.

# 7. X-Ray crystal structures of 4a′ and 5n and the assignment of configuration of 4a′′

(a) X-Ray Crystallographic Analysis Data of **4a′** (CCDC 2346690)

The crystal was obtained by slow solvent evaporation of a solution of **4a′** in n-hexane/ diethyl ether (1:1) at rt.


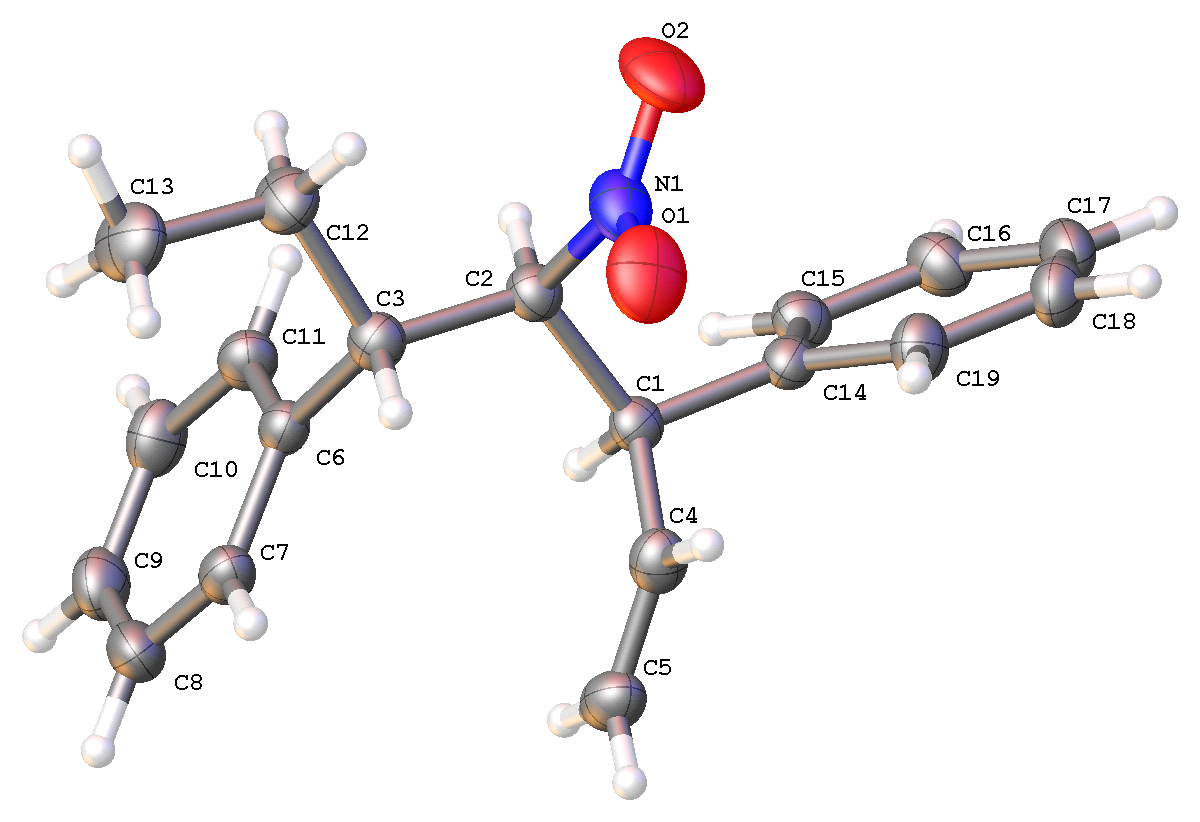


**Figure S2.** X-ray analysis of **4a′**.

Table S8. Crystal data and structure refinement for 4a′.

Identification code mj21502_0m

Empirical formula C19 H21 N O2

Formula weight 295.37

Temperature 213 K

Wavelength 1.34139 Å

Crystal system Monoclinic

Space group P 1 21 1

Unit cell dimensions a = 10.2249(8) Å a= 90°.

b = 8.0771(6) Å b= 102.393(4)°.

c = 10.4322(7) Å g = 90°.

Volume 841.49(11) Å3

Z 2

Density (calculated) 1.166 Mg/m3

Absorption coefficient 0.379 mm-1

F(000) 316

Crystal size 0.07 x 0.07 x 0.05 mm3

Theta range for data collection 3.774 to 55.044°.

Index ranges -12<=h<=12, -9<=k<=9, -12<=l<=12

Reflections collected 10419

Independent reflections 3164 [R(int) = 0.0451]

Completeness to theta = 53.594° 98.8 %

Absorption correction Semi-empirical from equivalents

Max. and min. transmission 0.7508 and 0.5118

Refinement method Full-matrix least-squares on F2

Data / restraints / parameters 3164 / 1 / 200

Goodness-of-fit on F2 1.053

Final R indices [I>2sigma(I)] R1 = 0.0383, wR2 = 0.0976

R indices (all data) R1 = 0.0415, wR2 = 0.1005

Absolute structure parameter 0.06(18)

Extinction coefficient n/a

Largest diff. peak and hole 0.125 and -0.134 e.Å-3

(b) X-Ray Crystallographic Analysis Data of **5n** (CCDC 2346691)

The crystal was obtained by slow solvent evaporation of a solution of **5n** in CHCl_3_ at rt.


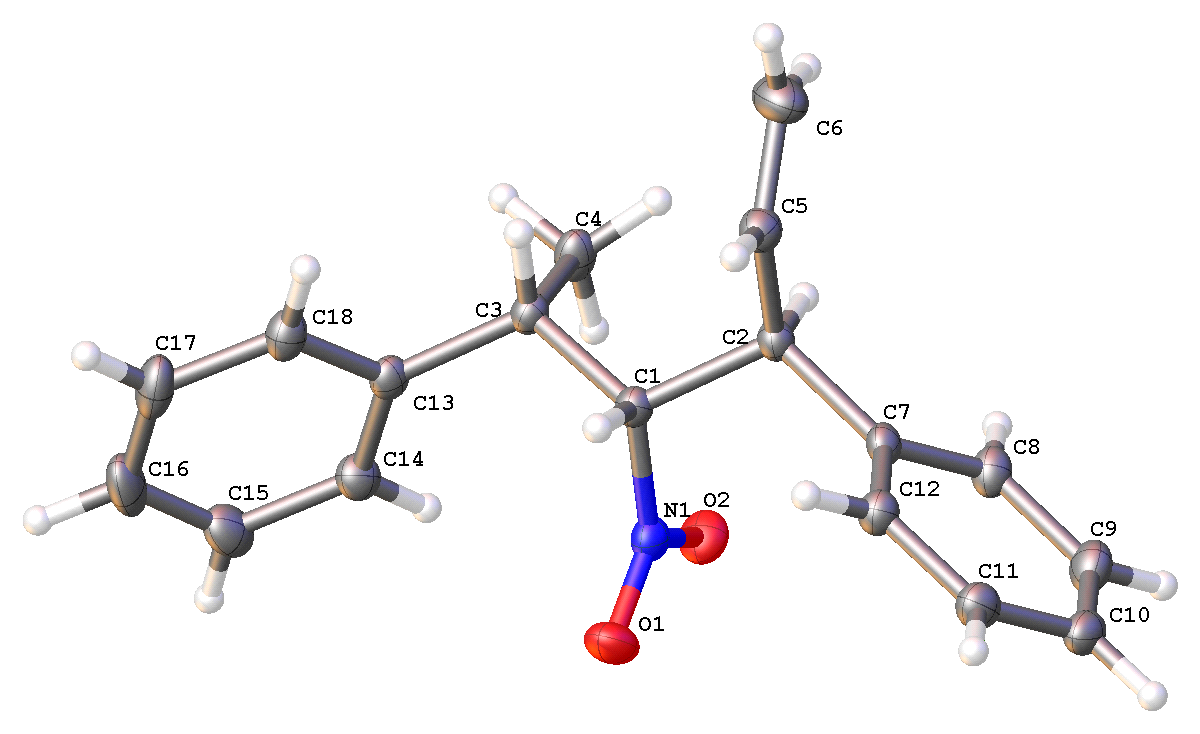


**Figure S3.** X-ray analysis of **5n**.

Table S9. Crystal data and structure refinement for 5n.

Identification code mj21531

Empirical formula C18 H19 N O2

Formula weight 281.34

Temperature 213.01 K

Wavelength 1.34139 Å

Crystal system Orthorhombic

Space group P2**_1_**2**_1_**2

Unit cell dimensions a = 13.4859(2) Å a= 90°.

b = 19.4208(3) Å b= 90°.

c = 5.78210(10) Å g = 90°.

Volume 1514.37(4) Å3

Z 4

Density (calculated) 1.234 Mg/m3

Absorption coefficient 0.406 mm-1

F(000) 600

Crystal size 0.07 x 0.07 x 0.05 mm3

Theta range for data collection 3.471 to 54.867°.

Index ranges -16<=h<=16, -23<=k<=23, -6<=l<=7

Reflections collected 10472

Independent reflections 2848 [R(int) = 0.0386]

Completeness to theta = 53.594° 99.1 %

Absorption correction Semi-empirical from equivalents

Max. and min. transmission 0.7508 and 0.5732

Refinement method Full-matrix least-squares on F2

Data / restraints / parameters 2848 / 0 / 191

Goodness-of-fit on F2 1.093

Final R indices [I>2sigma(I)] R1 = 0.0448, wR2 = 0.1199

R indices (all data) R1 = 0.0479, wR2 = 0.1230

Absolute structure parameter 0.01(16)

Extinction coefficient n/a

Largest diff. peak and hole 0.283 and -0.236 e.Å-3

(c) The assignment of the configuration of **4a′′**.


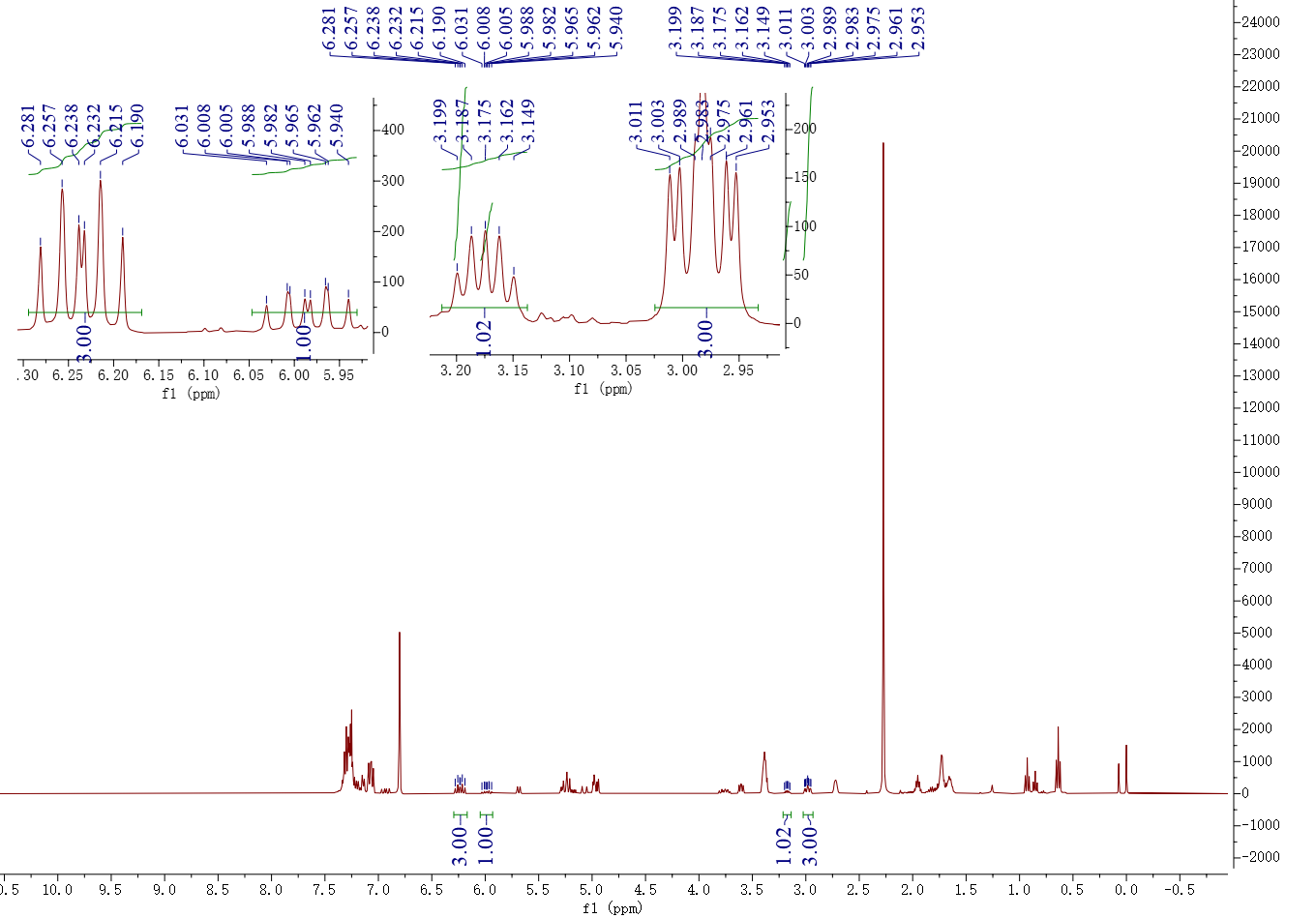


Figure S4. The assignment of the configuration of 4a′′.

Under Ar atmosphere, **4a′** (14.8 mg, 0.05 mmol, 1 equiv), Et_2_O (1.0 mL) and DBU (7.6 mg, 0.05 mmol, 1 equiv) were added to an oven-dried Schlenk tube. The reaction was stirred at rt for 24 h. Then the crude mixture was filtered with a pad of celite and washed with EtOAc (10 mL x 3). The filtrate was concentrated by rotary evaporation. In the crude ^1^H NMR spectrum, the formation of **4a′′** can be observed, and the ratio of **4a′** to **4a′′** is 3:1. The corresponding characteristic peaks have been partially enlarged in the crude ^1^H NMR spectrum (Figure **S4**). Therefore, the configuration of the α-position of the nitro group in **4a′** was proposed to be reversed under the basic conditions to obtain product **4a′′**.

# 8. References

[1] a) J. Rentner, R. Breinbauer, *Chem. Commun.* **2012**, *48*, 10343-10345; b) J. J. Galano, M. Alías, R. Pérez, A. Velázquez-Campoy, P. S. Hoffman, J. Sancho, *J. Med. Chem.* **2013**, *56*, 6248-6258; c) A. J. Simpson, H. W. Lam, *Org. Lett.* **2013**, *15*, 2586-2589; d) V. Ashokkumar, A. Siva, *Org. Biomol. Chem.* **2015**, *13*, 10216-10225; e) S. Guo, X. Mi, *Tetrahedron Lett.* **2017**, *58*, 2881-2884.

[2] a) H. Ito, Y. Kosaka, K. Nonoyama, Y. Sasaki, M. Sawamura, *Angew. Chem. Int. Ed.* **2008**, *47*, 7424-7427; *Angew. Chem.* **2008**, *120*, 7534-7537; b) X.-d. Bai, J. Wang, Y. He, *Adv. Synth. Catal.* **2018**, *361*, 496-501; c) C.-Y. Shi, J.-Z. Xiao, L. Yin, *Chem. Commun.* **2018**, *54*, 11957-11960; d) T. Schlatzer, H. Schröder, M. Trobe, C. Lembacher-Fadum, S. Stangl, C. Schlögl, H. Weber, R. Breinbauer, *Adv. Synth. Catal.* **2020**, *362*, 331-336; e) Y. Chen, X. Song, L. Gao, Z. Song, *Org. Lett.* **2021**, *23*, 124-128; f) Z. Kang, W. Chang, X. Tian, X. Fu, W. Zhao, X. Xu, Y. Liang, W. Hu, *J. Am. Chem. Soc.* **2021**, *143*, 20818-20827.

[3] For iridium catalyst prepared via *^n^*PrNH_2_ activation, see: C. Shu, A. Leitner, J. F. Hartwig, *Angew. Chem. Int. Ed.* **2004**, *43*, 4797-4800; *Angew. Chem.* **2004**, *116*, 4901-4904.

# 9. Copies of NMR spectra

# 4a ^1^H NMR


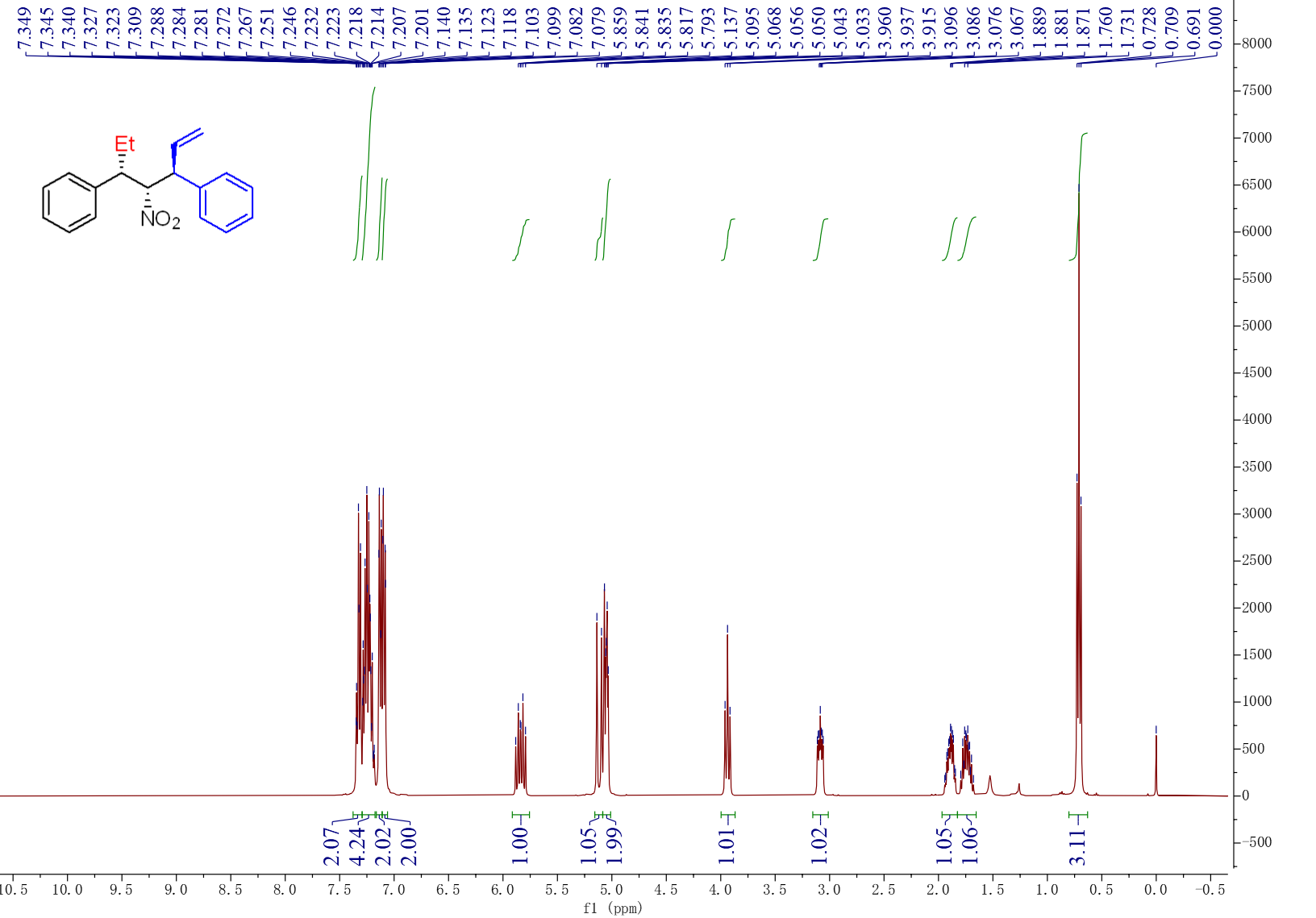


**4a** ^13^C NMR


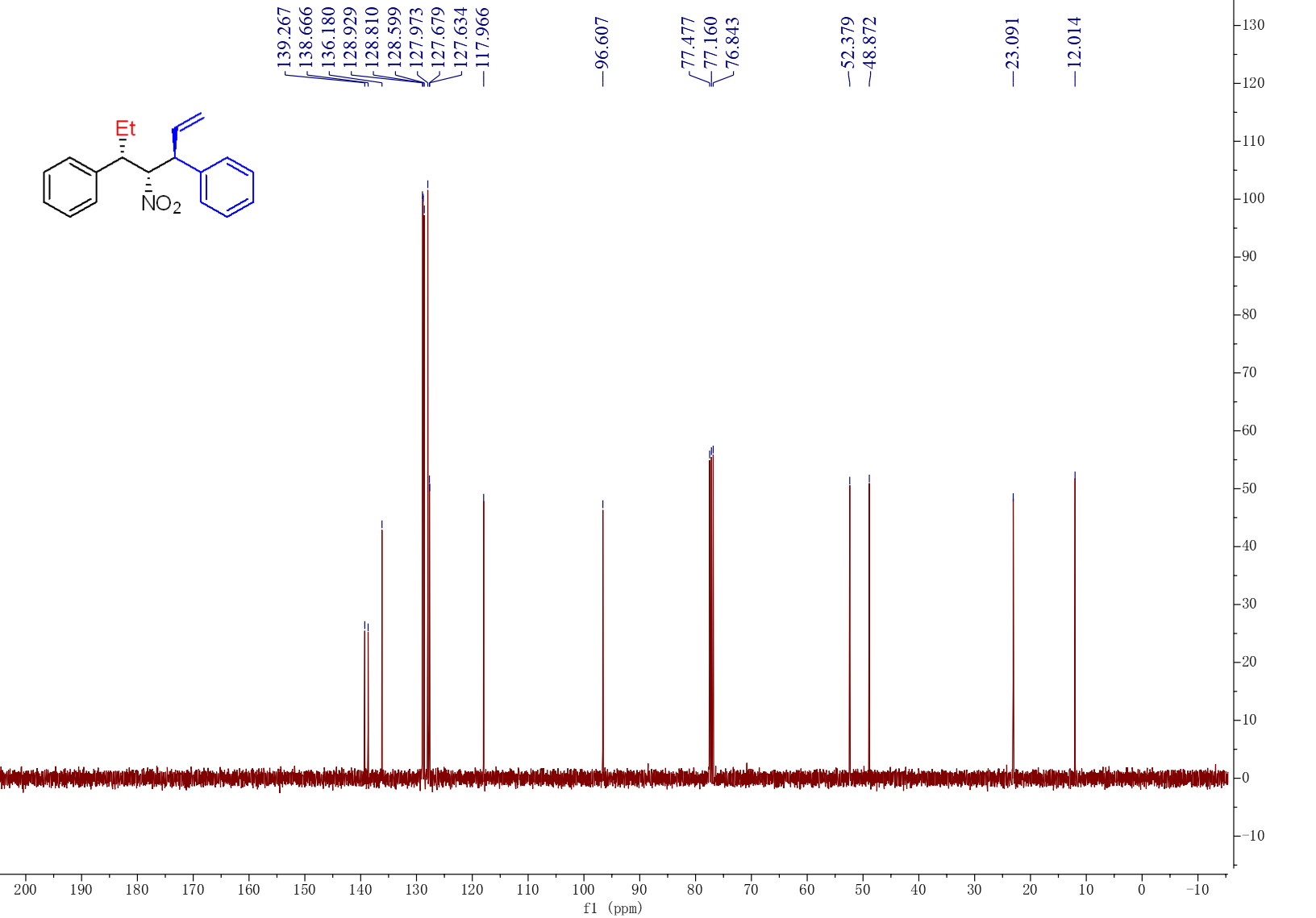


**4b** ^1^H NMR


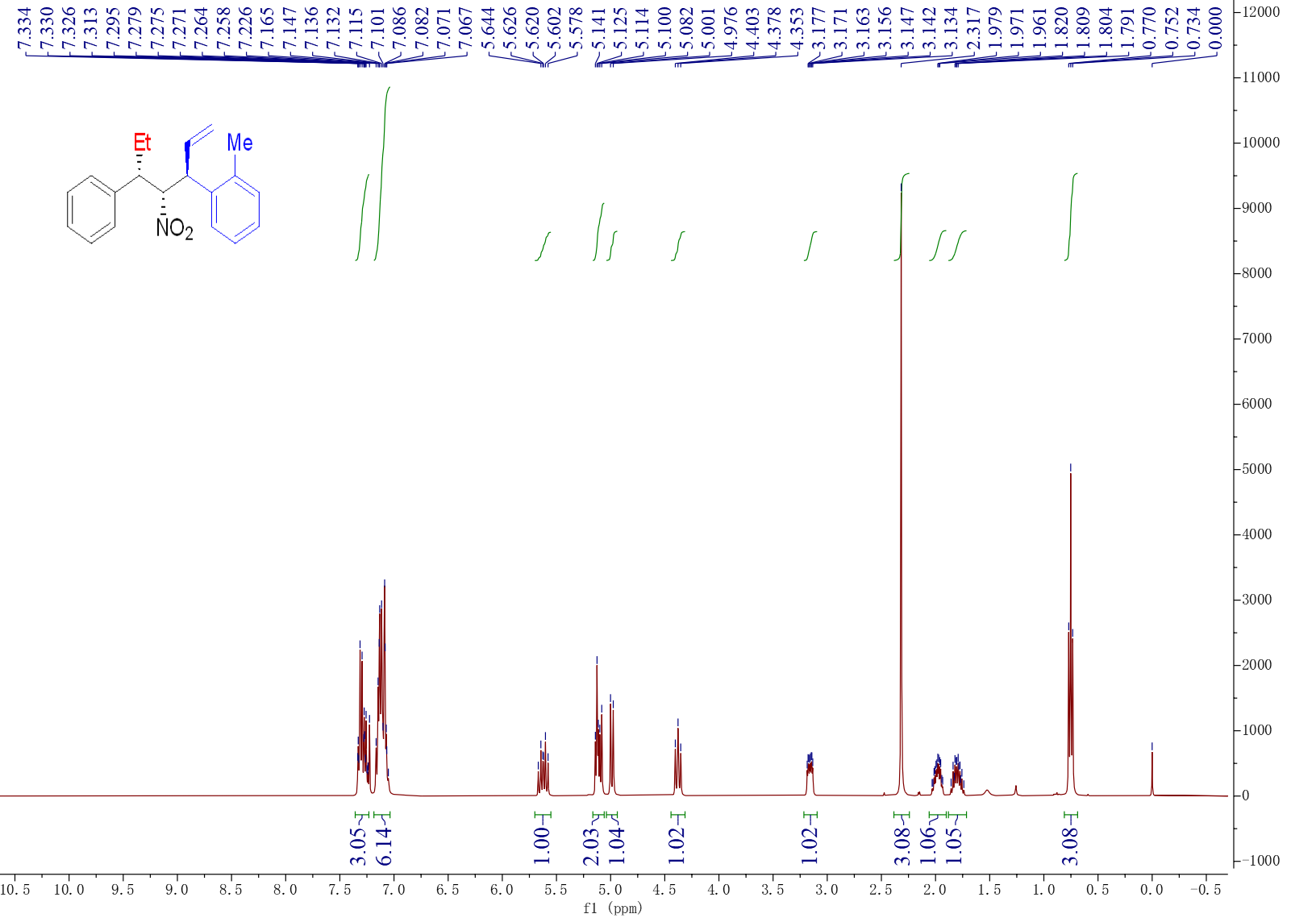


**4b** ^13^C NMR


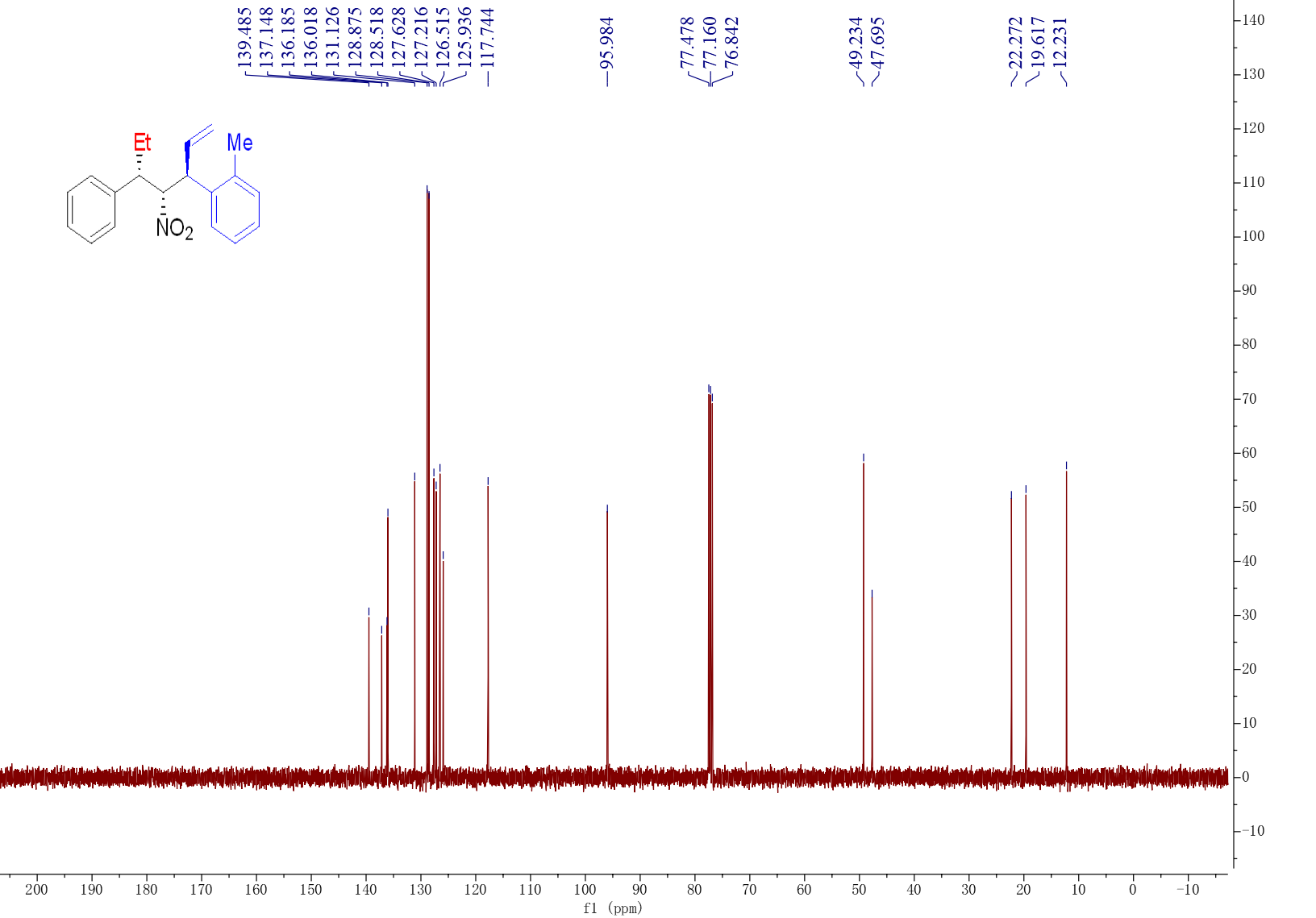


**4c** ^1^H NMR


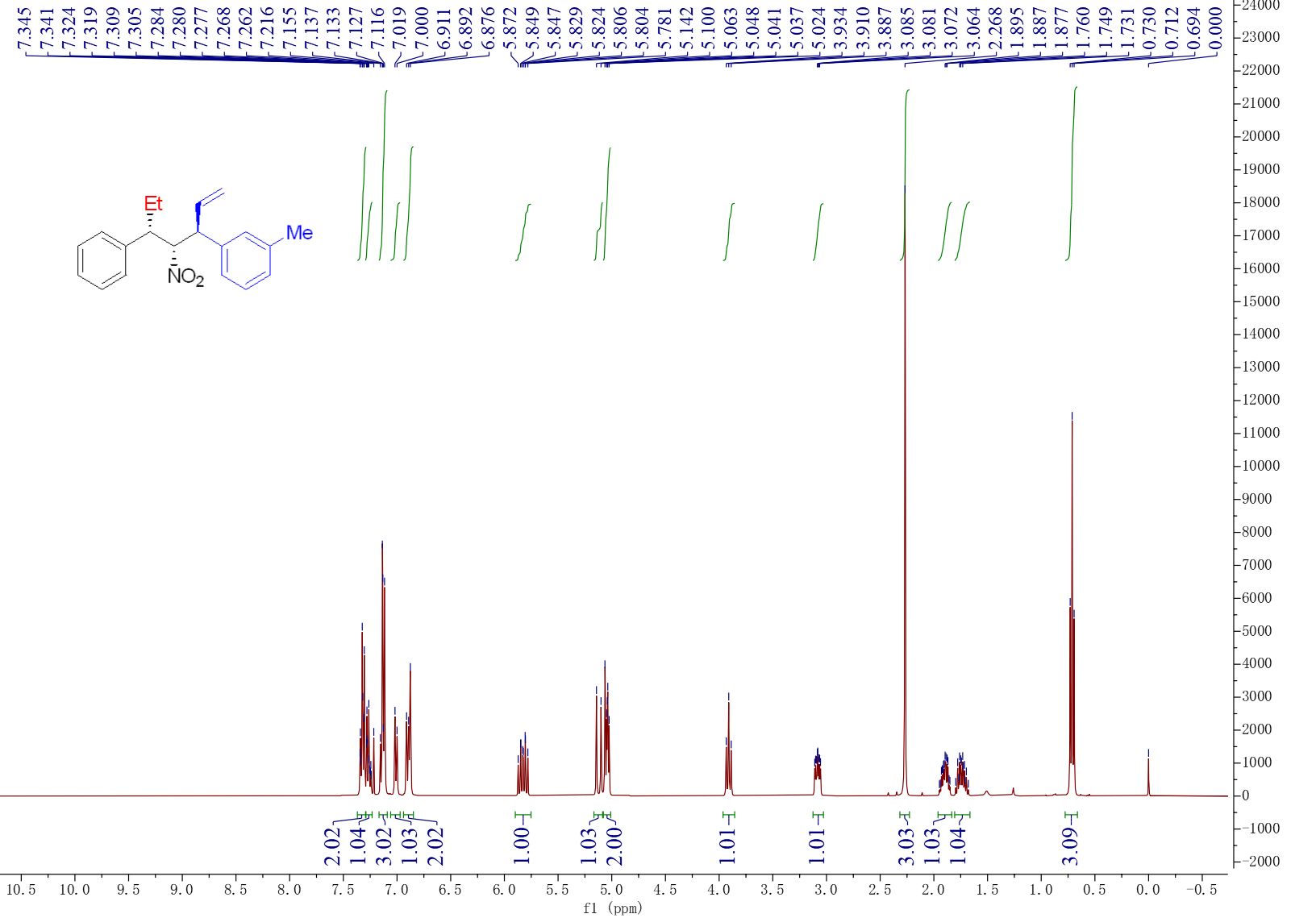


**4c** ^13^C NMR


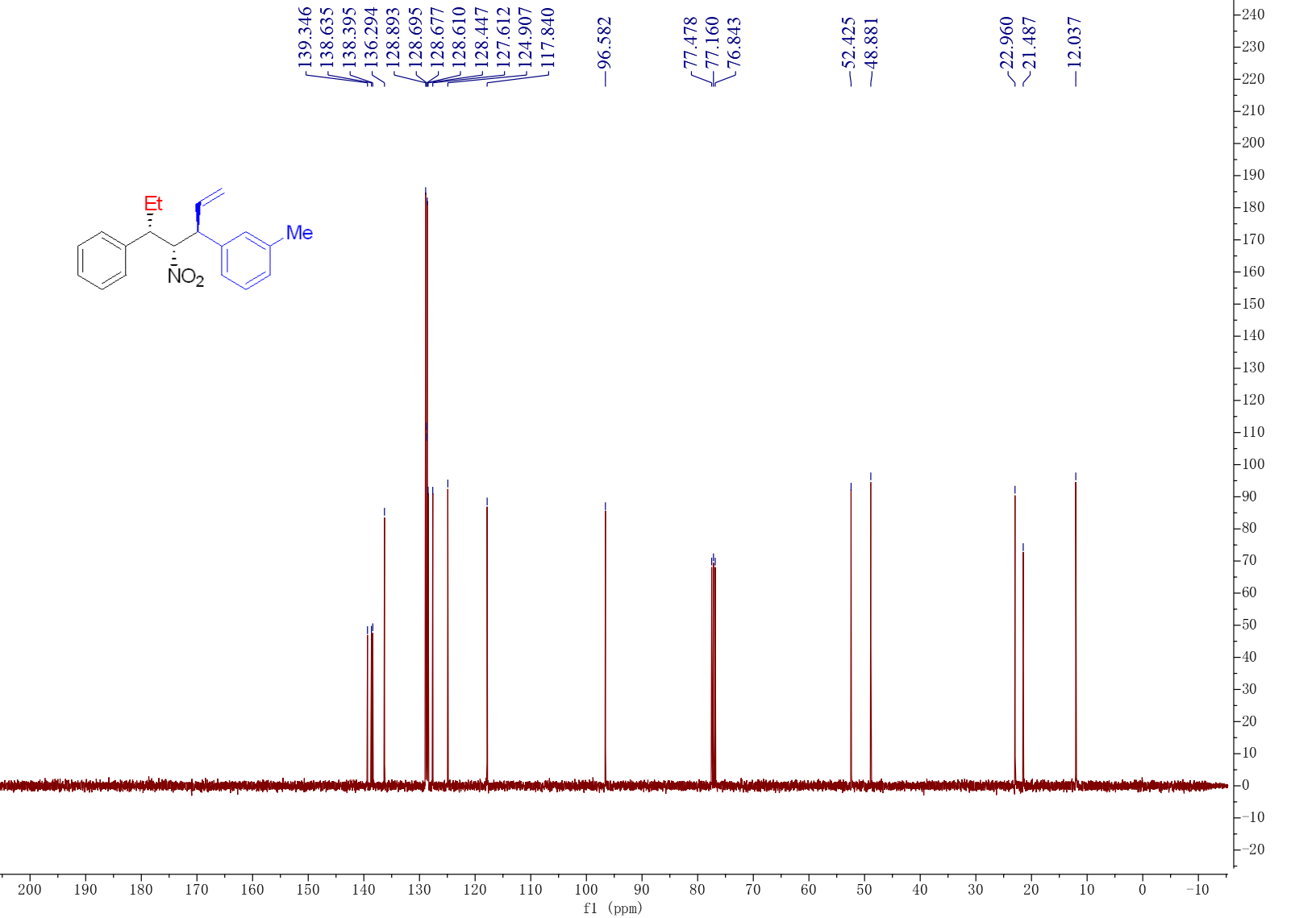


**4d** ^1^H NMR


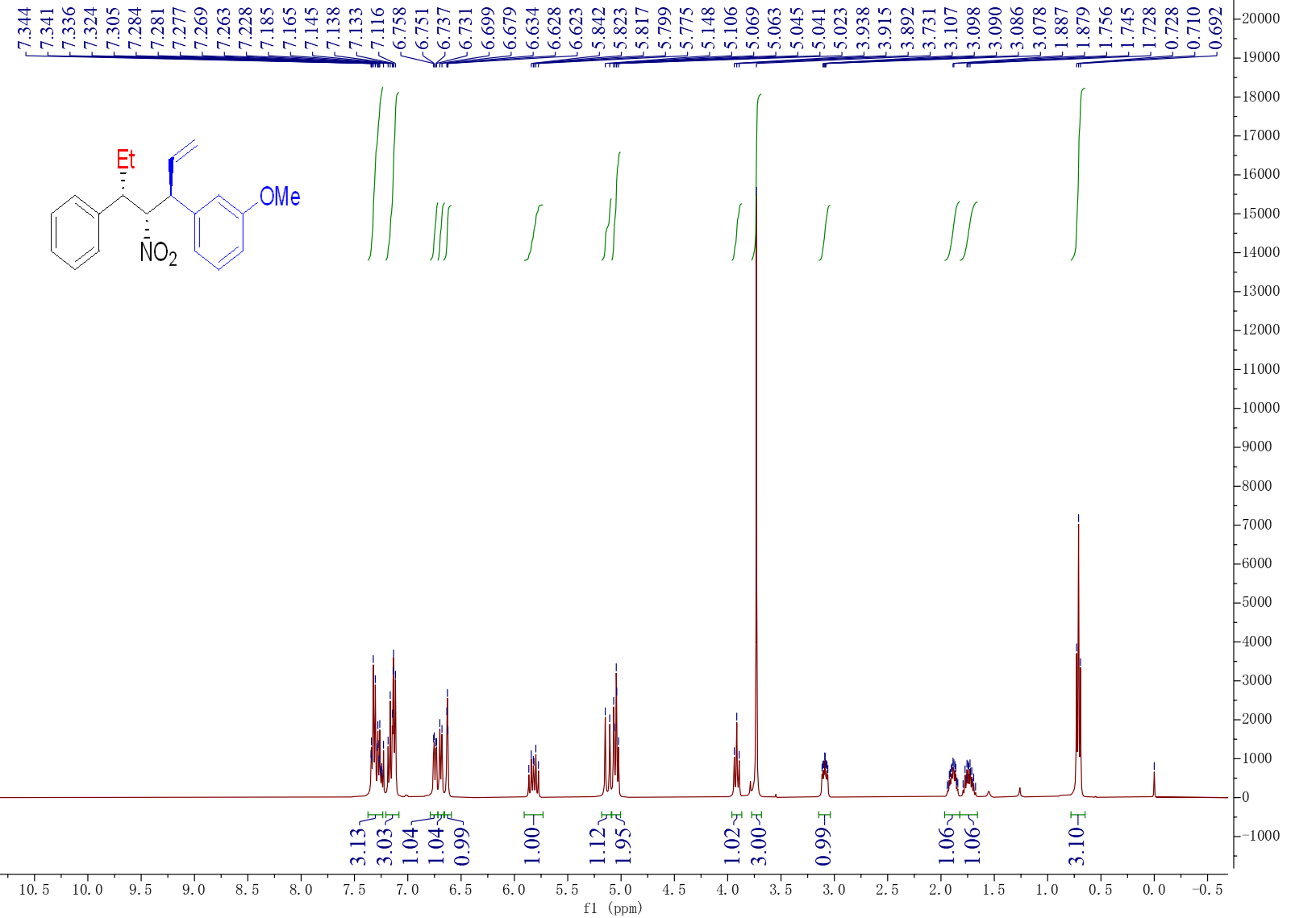


**4d** ^13^C NMR


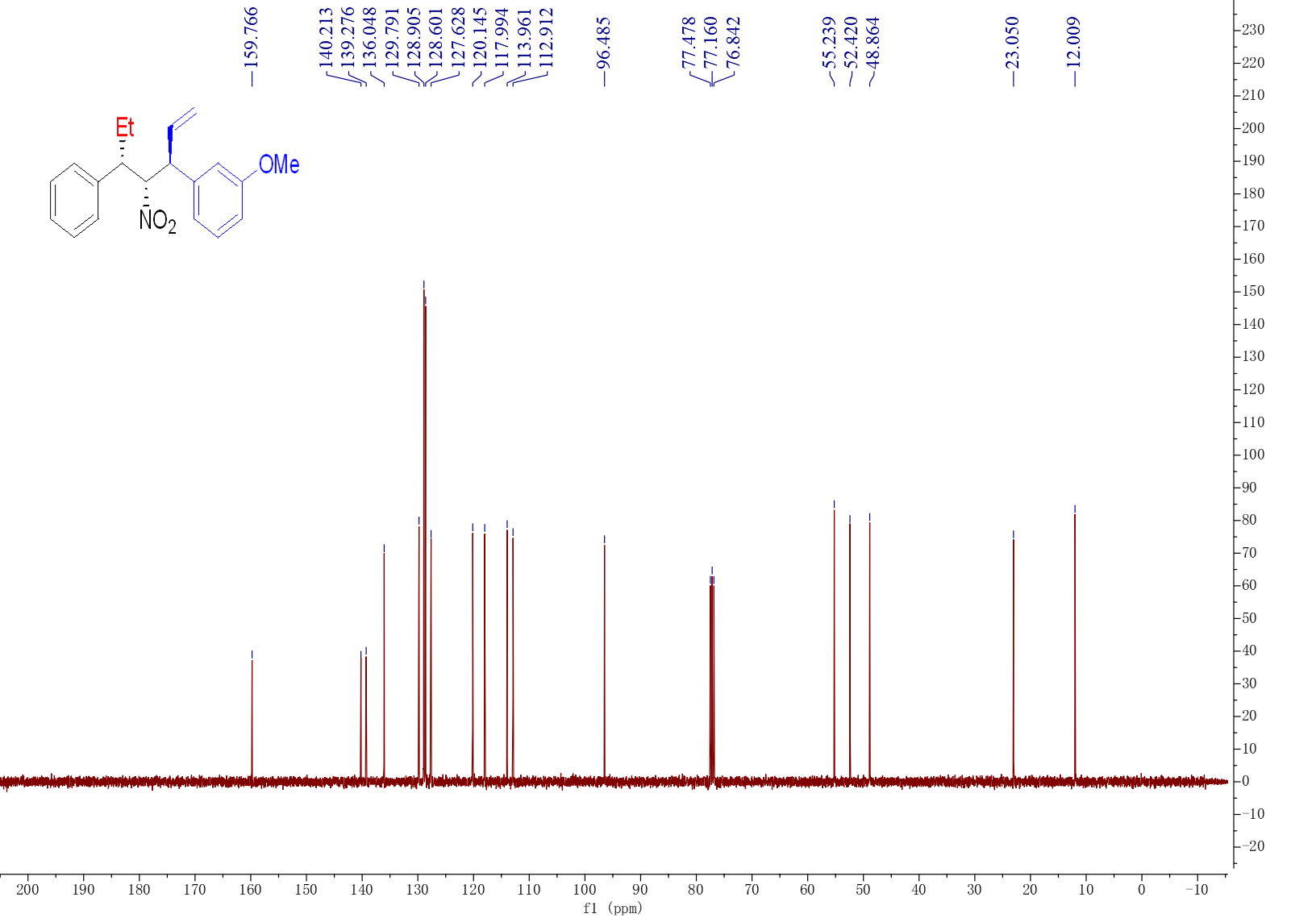


**4e** ^1^H NMR


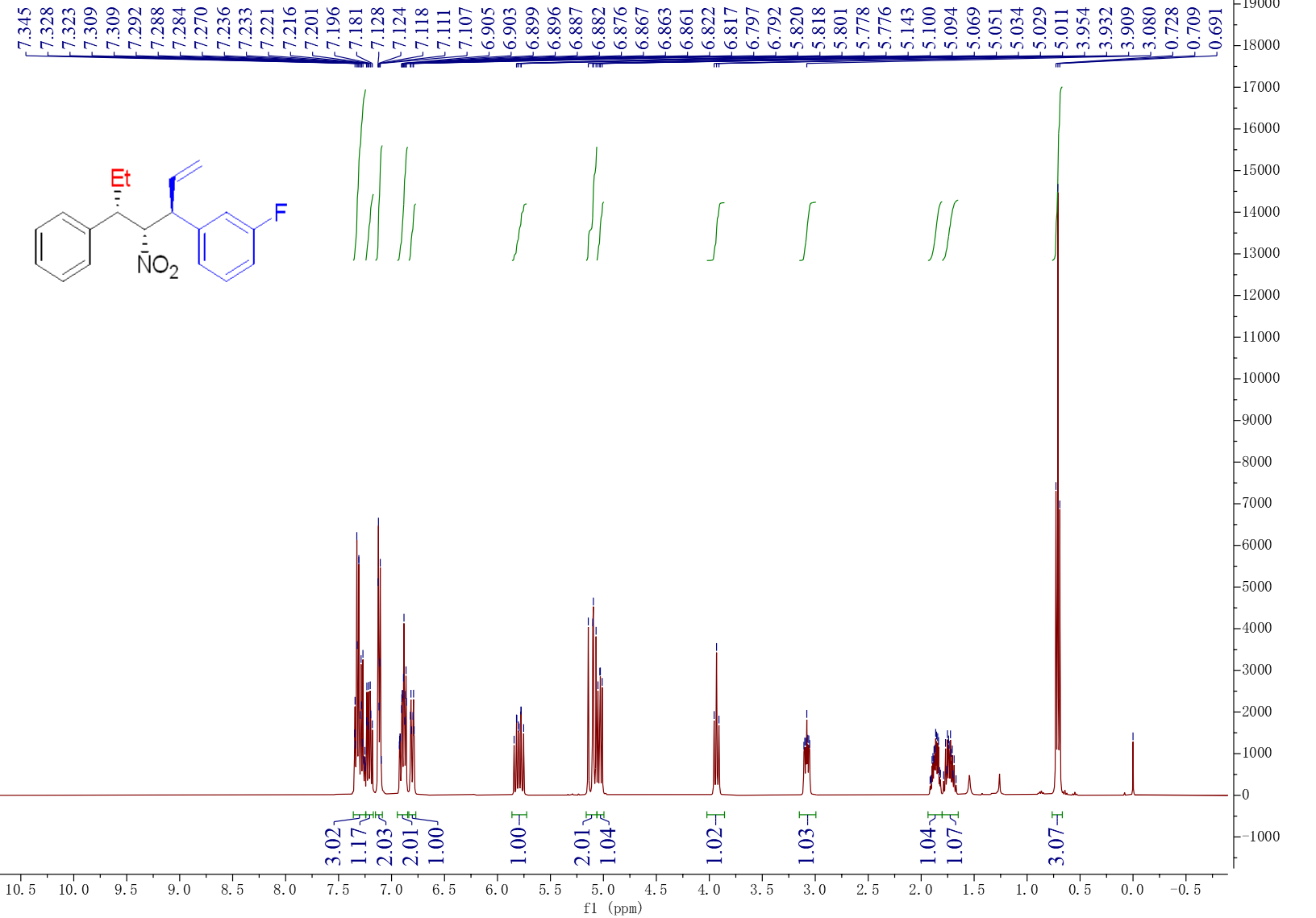


**4e** ^13^C NMR


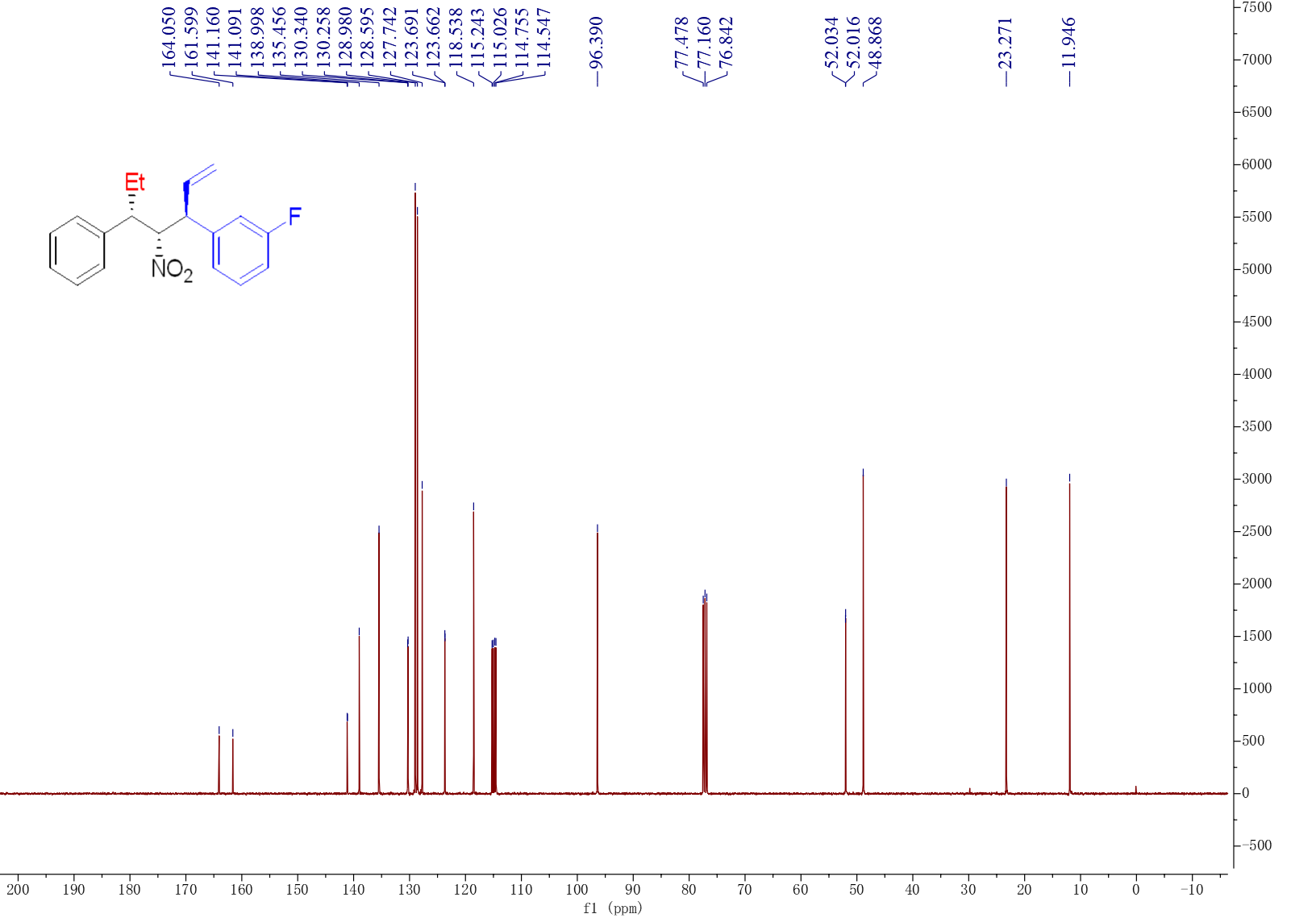


**4e** ^19^F NMR


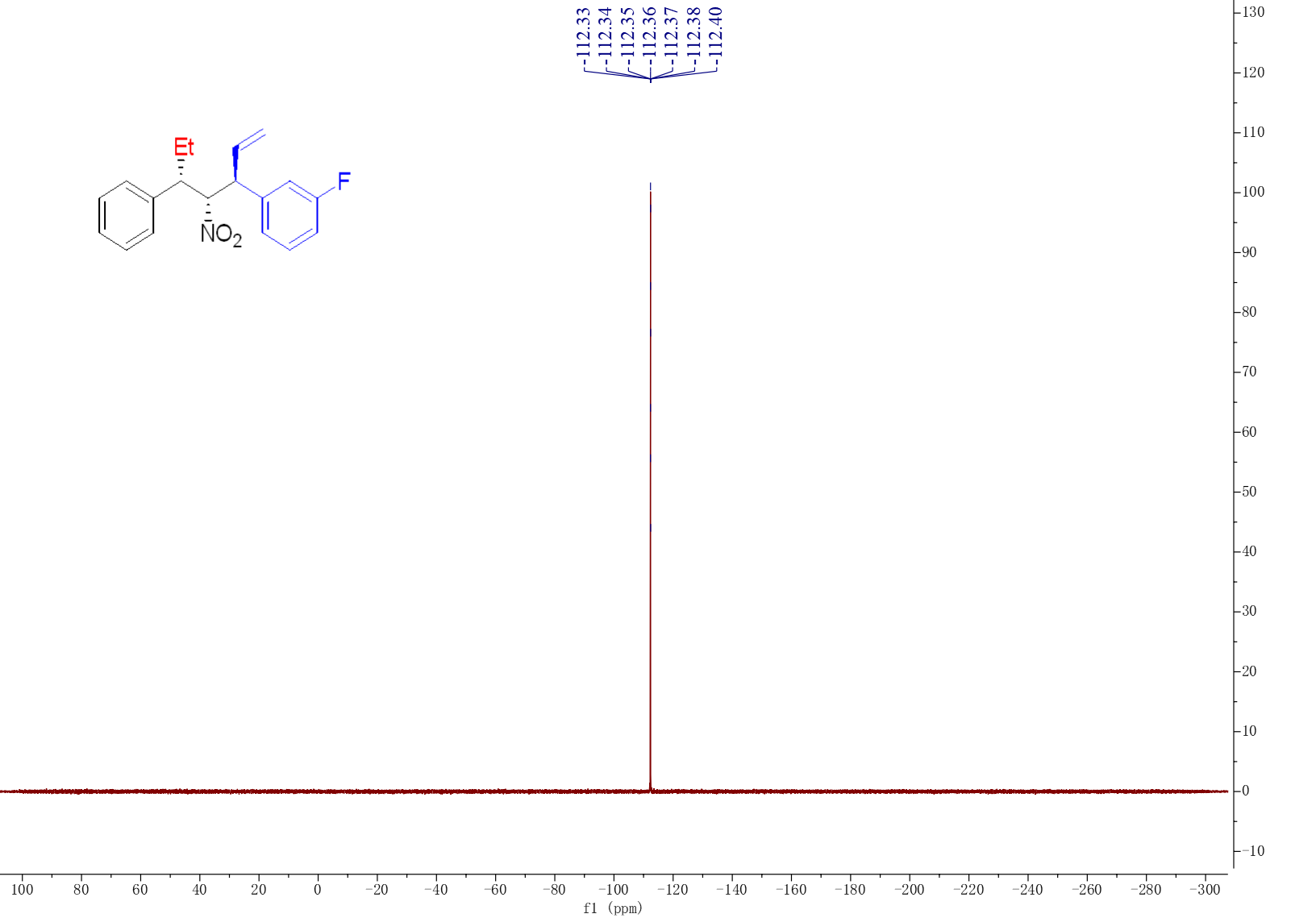


**4f** ^1^H NMR


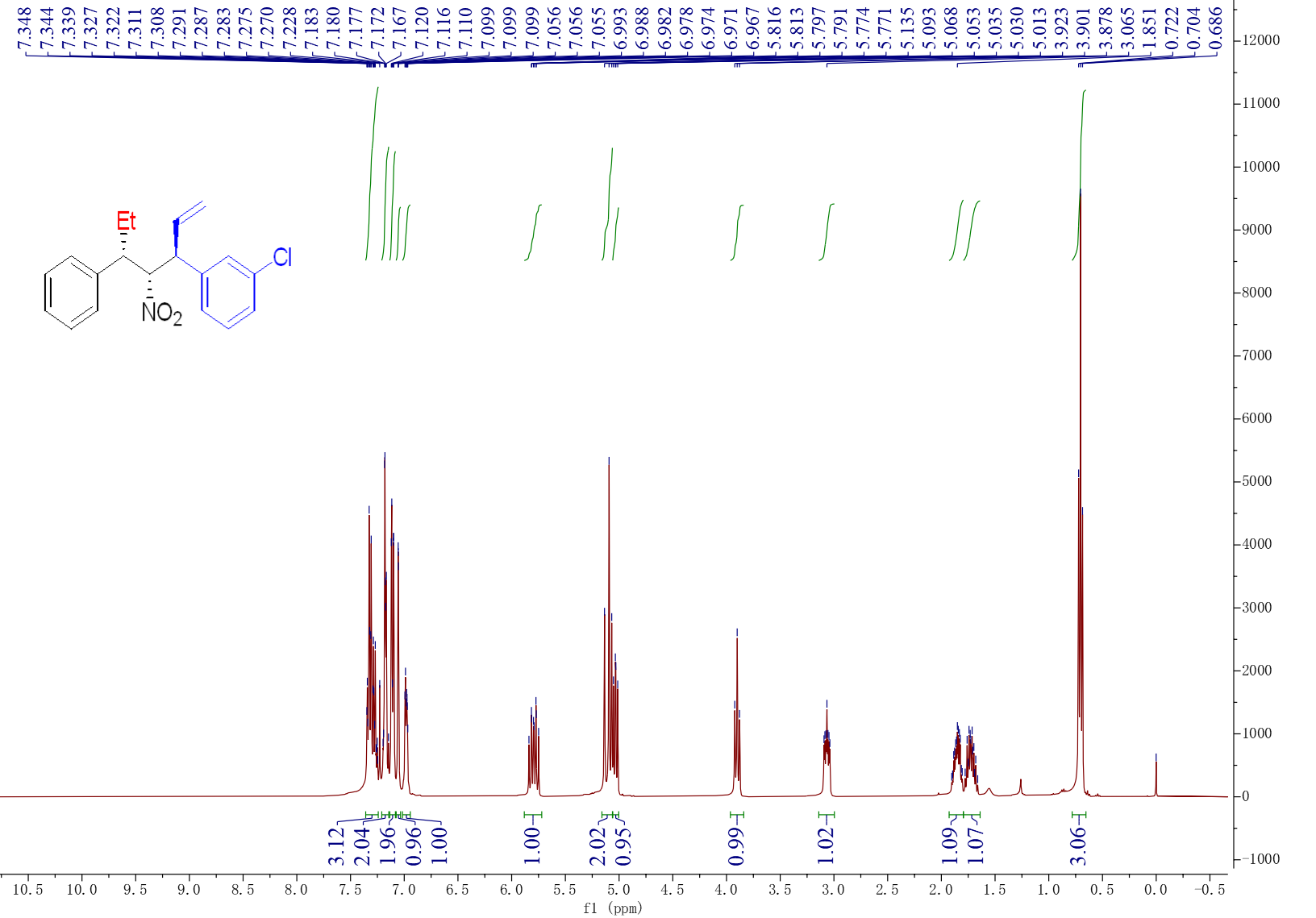


**4f** ^13^C NMR


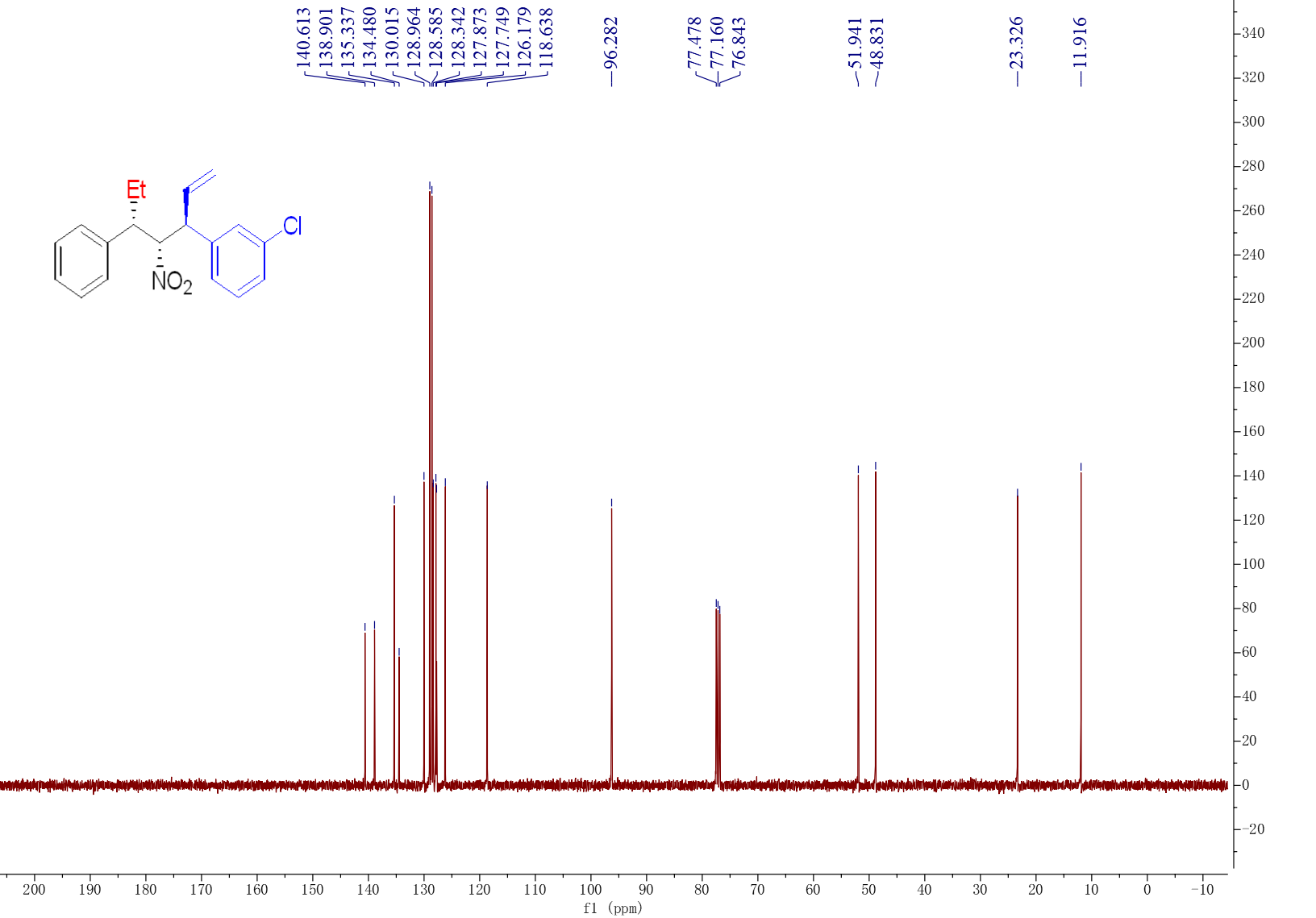


**4g** ^1^H NMR


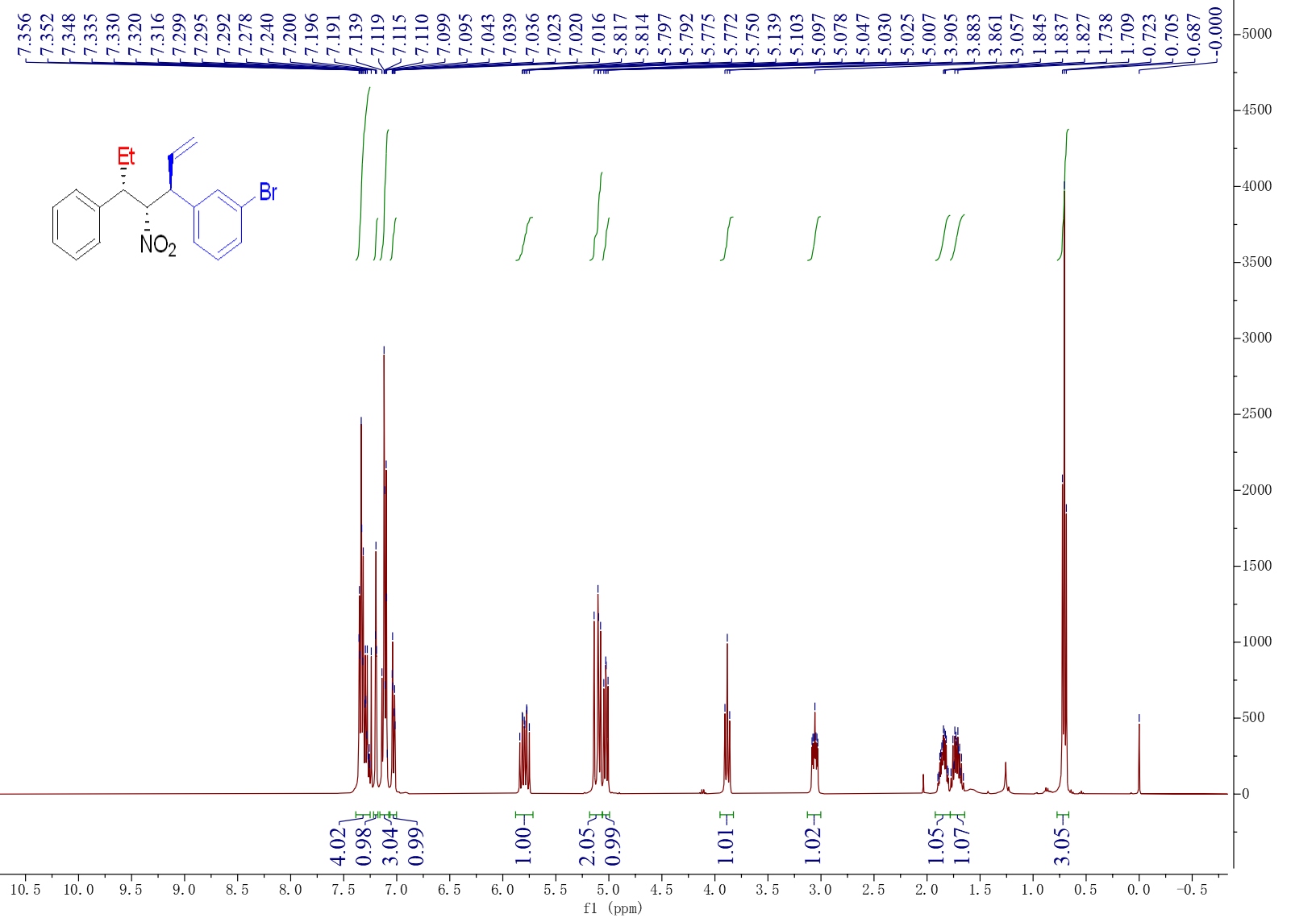


**4g** ^13^C NMR


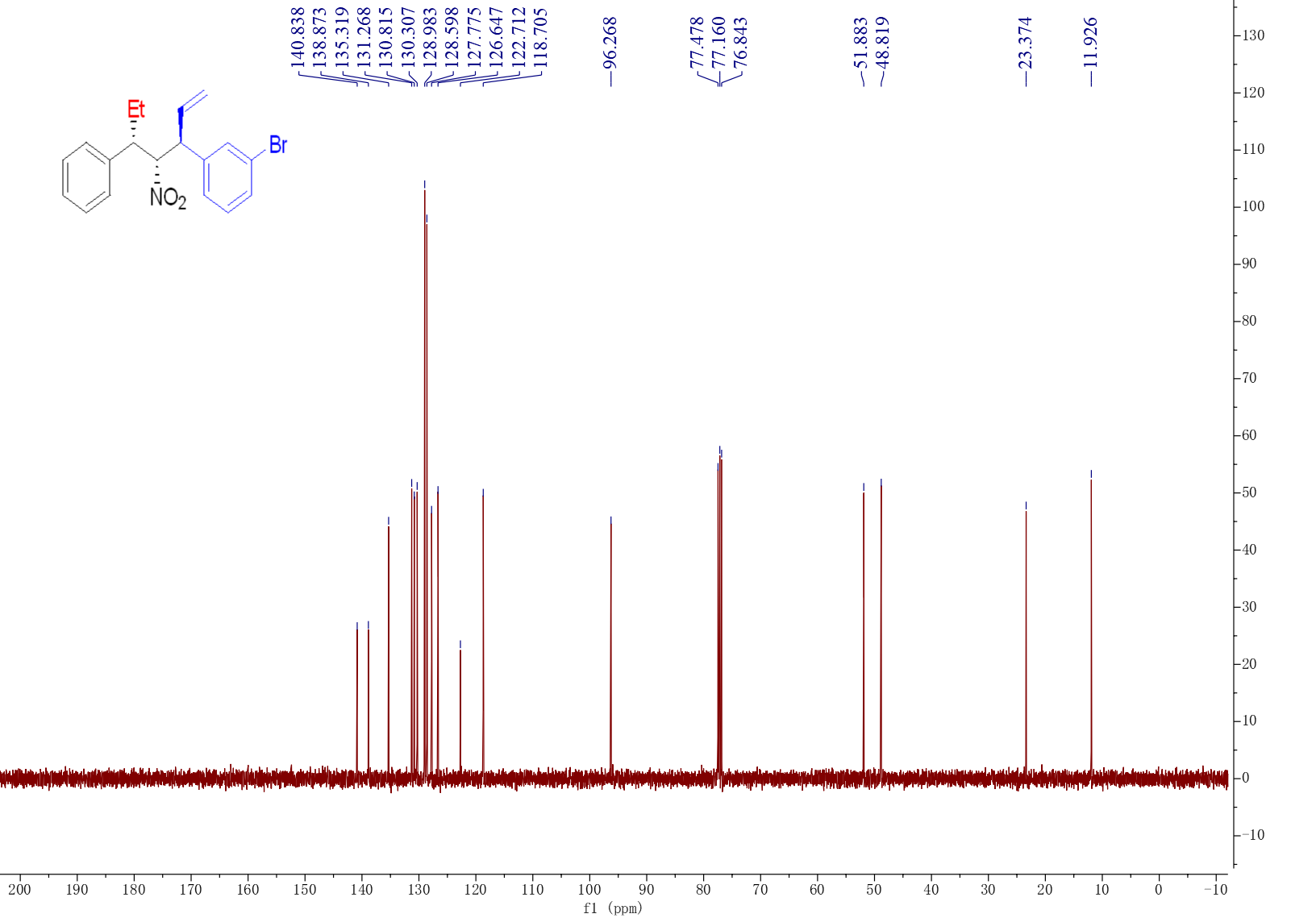


**4h** ^1^H NMR


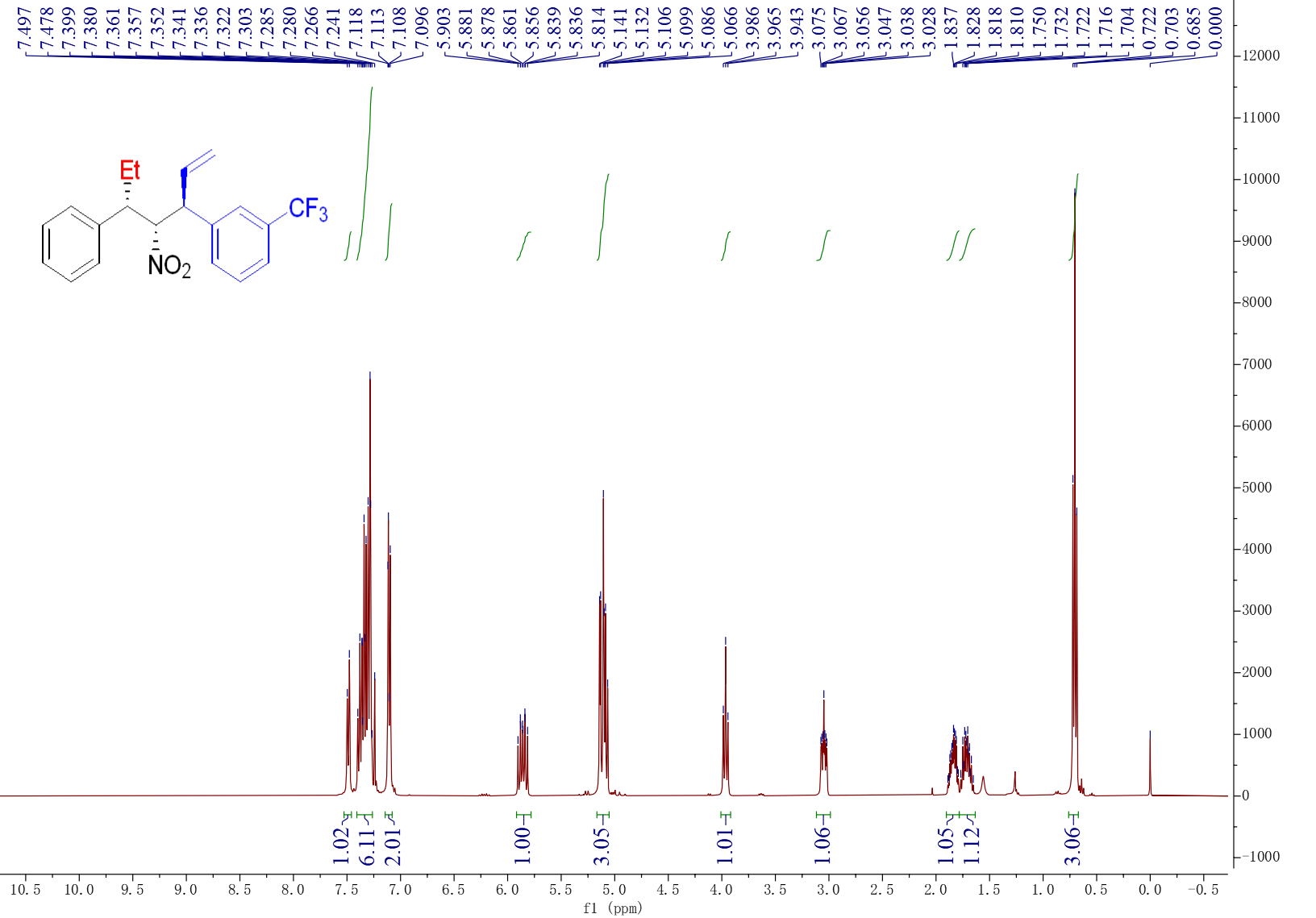


**4h** ^13^C NMR


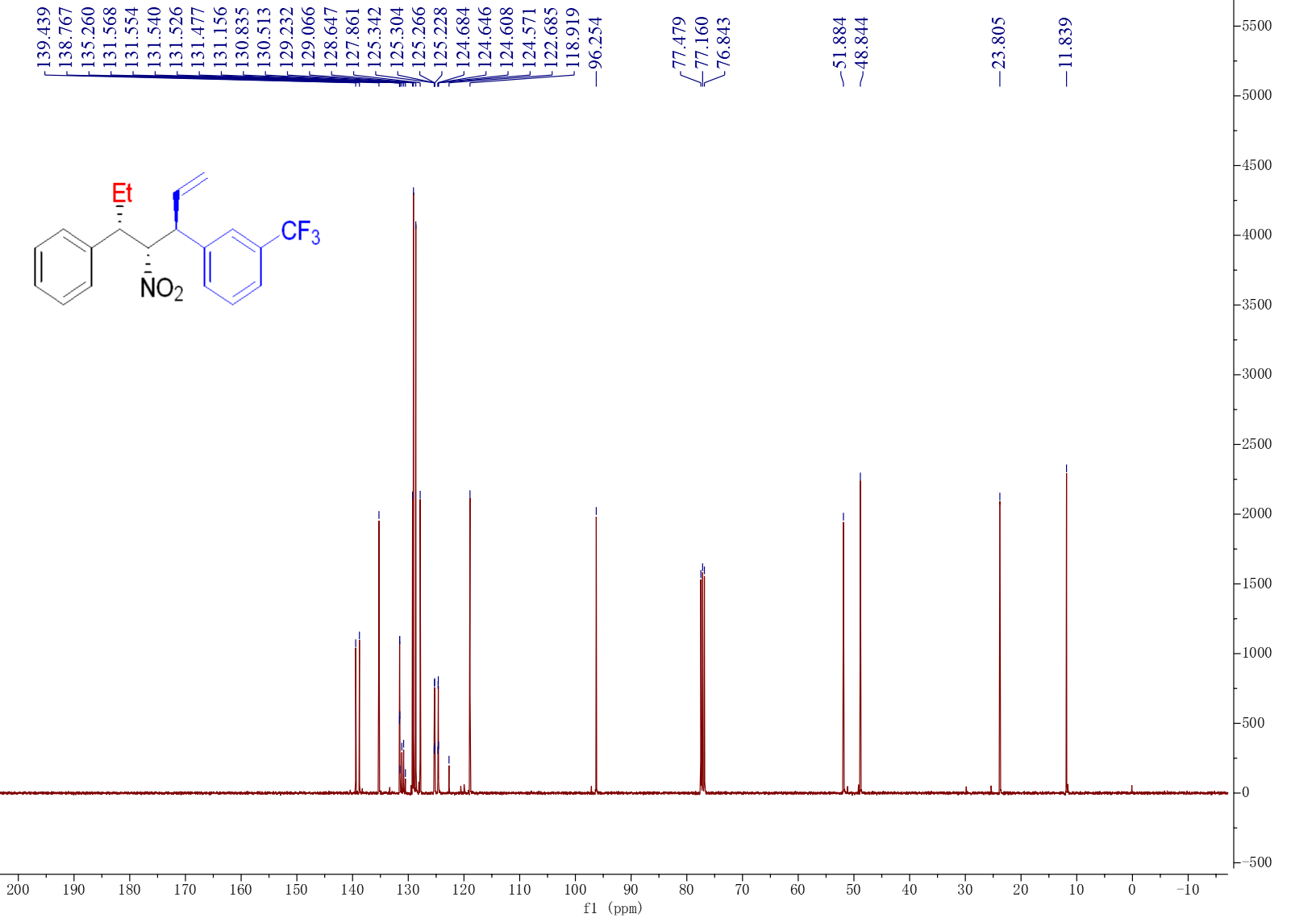


**4h** ^19^F NMR


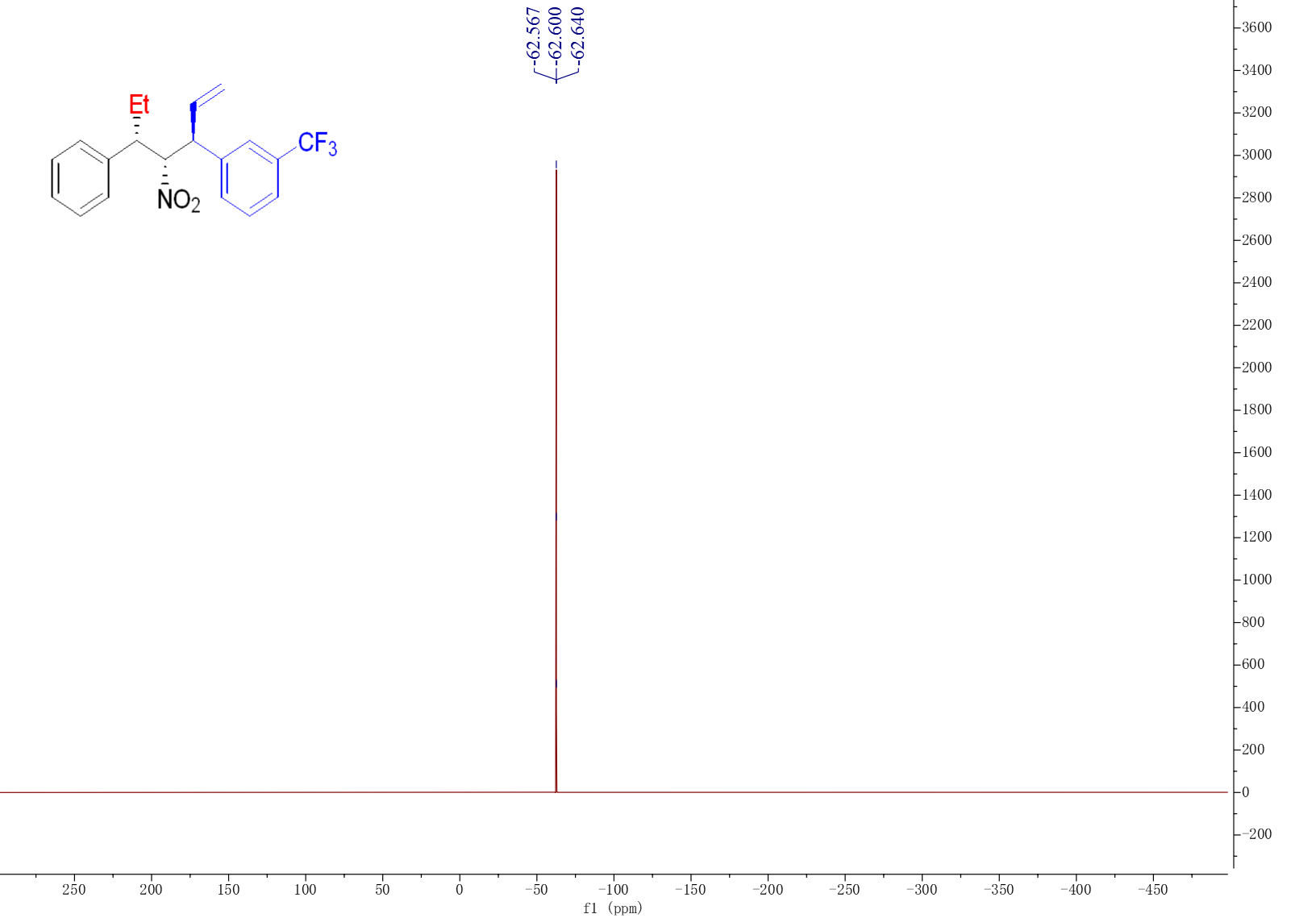


**4i** ^1^H NMR


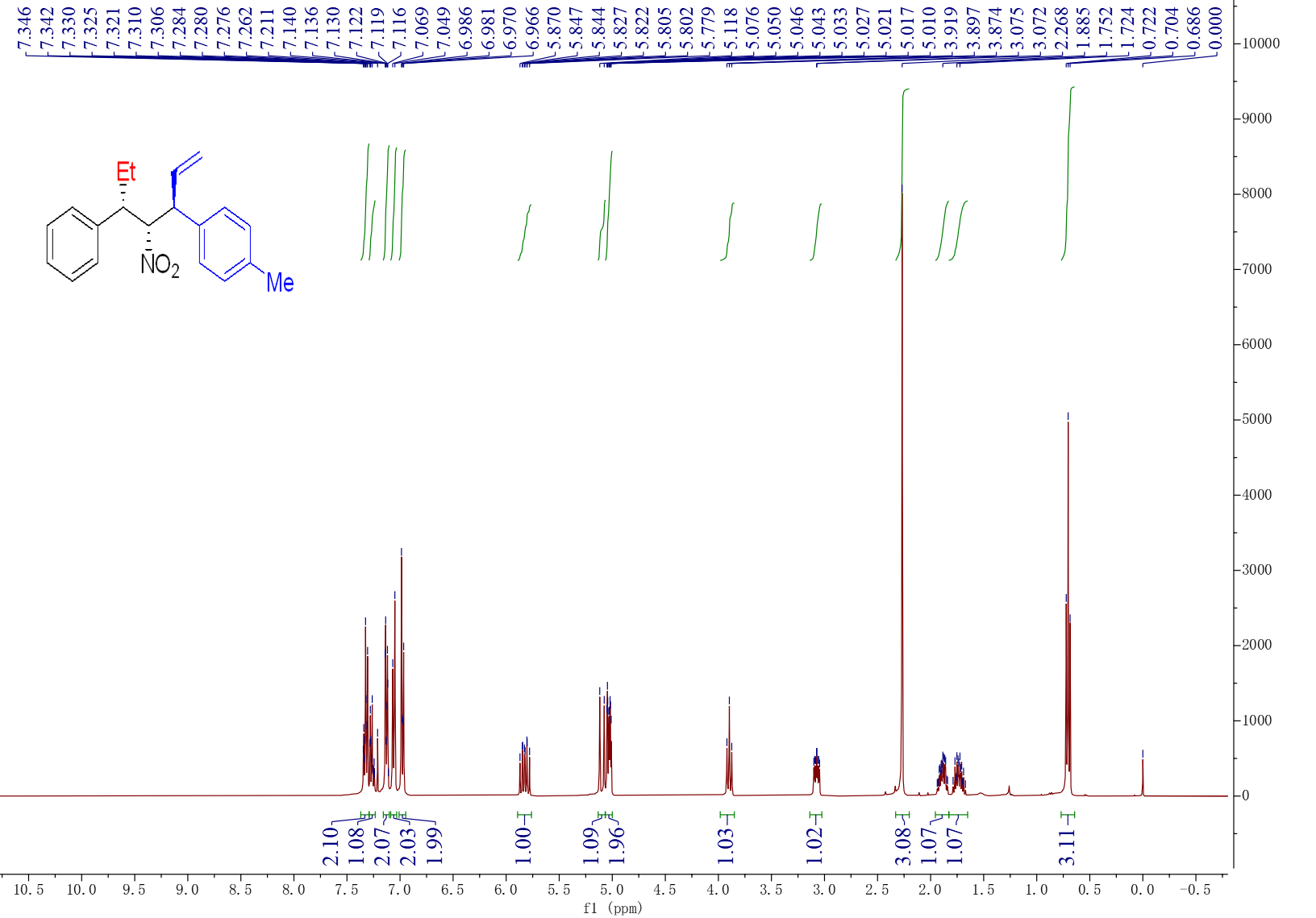


**4i** ^13^C NMR


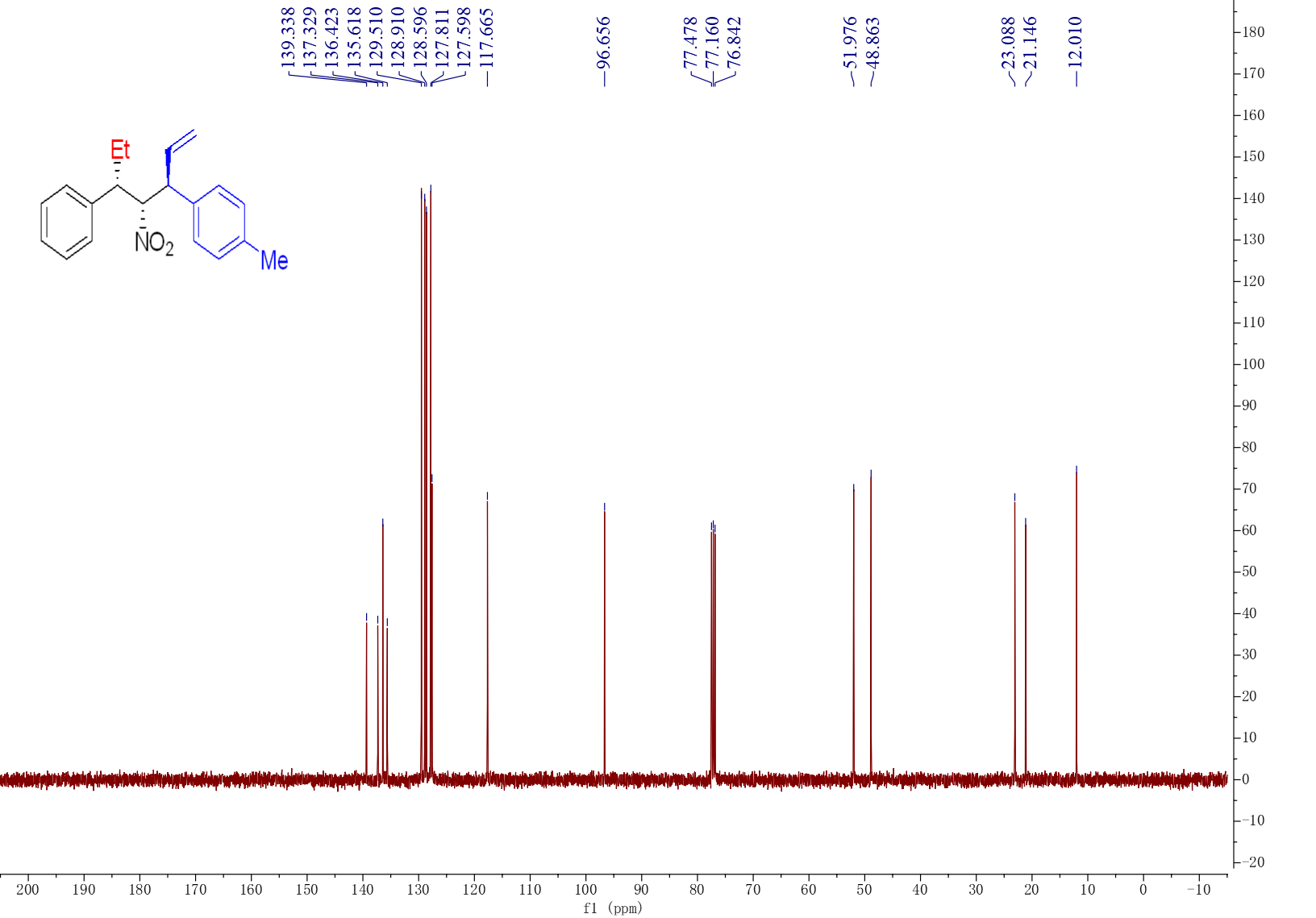


**4j** ^1^H NMR


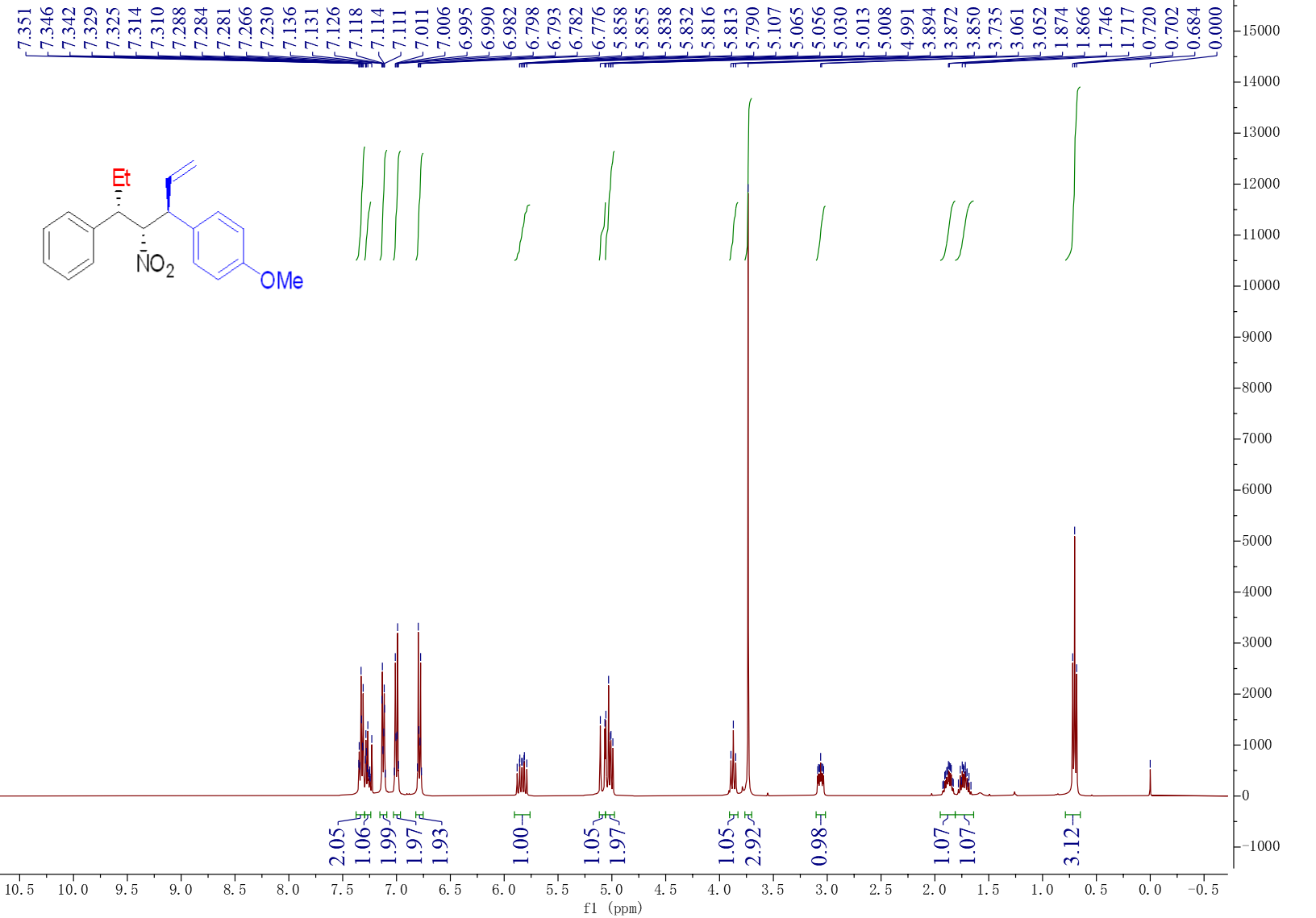


**4j** ^13^C NMR


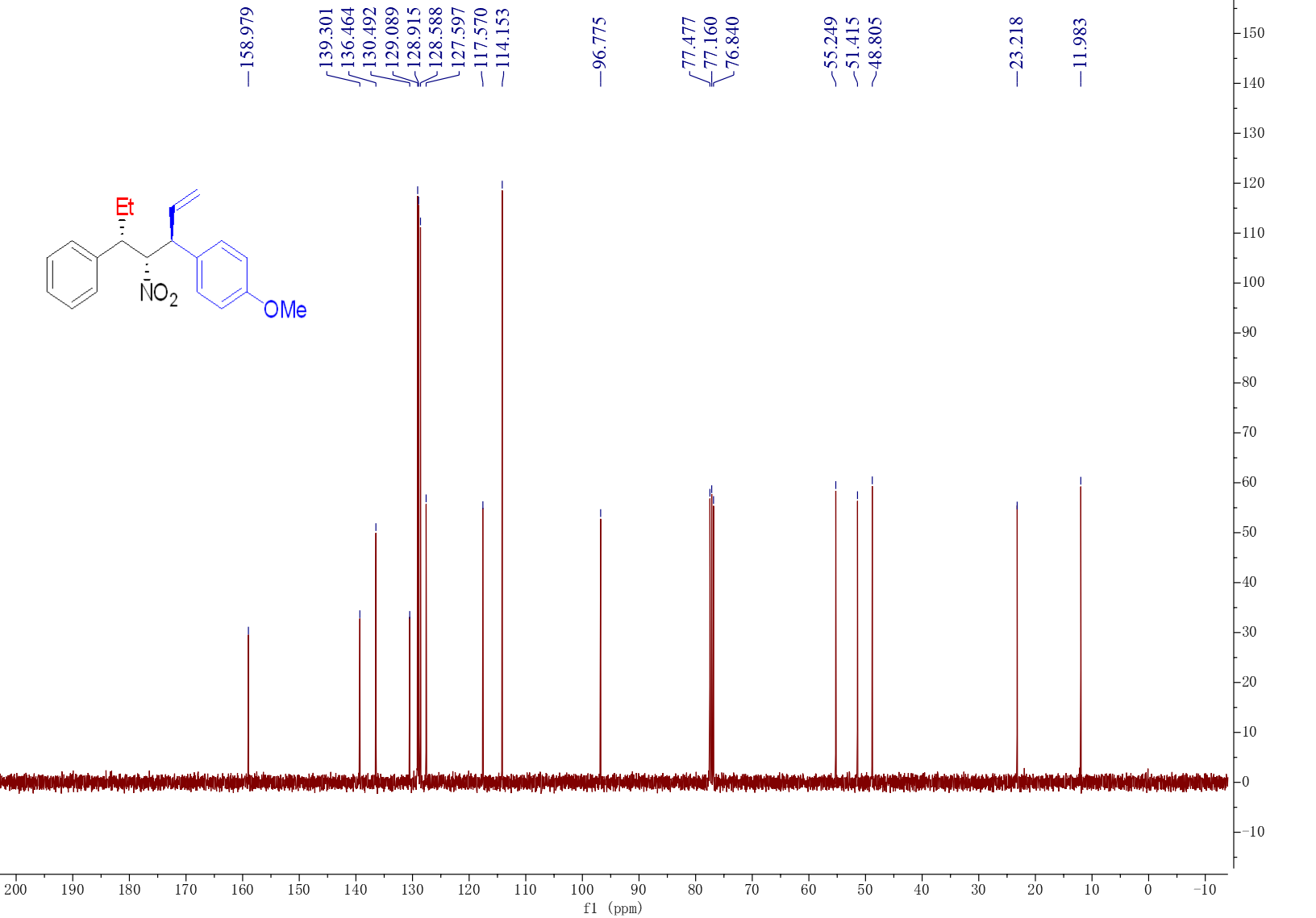


**4k** ^1^H NMR


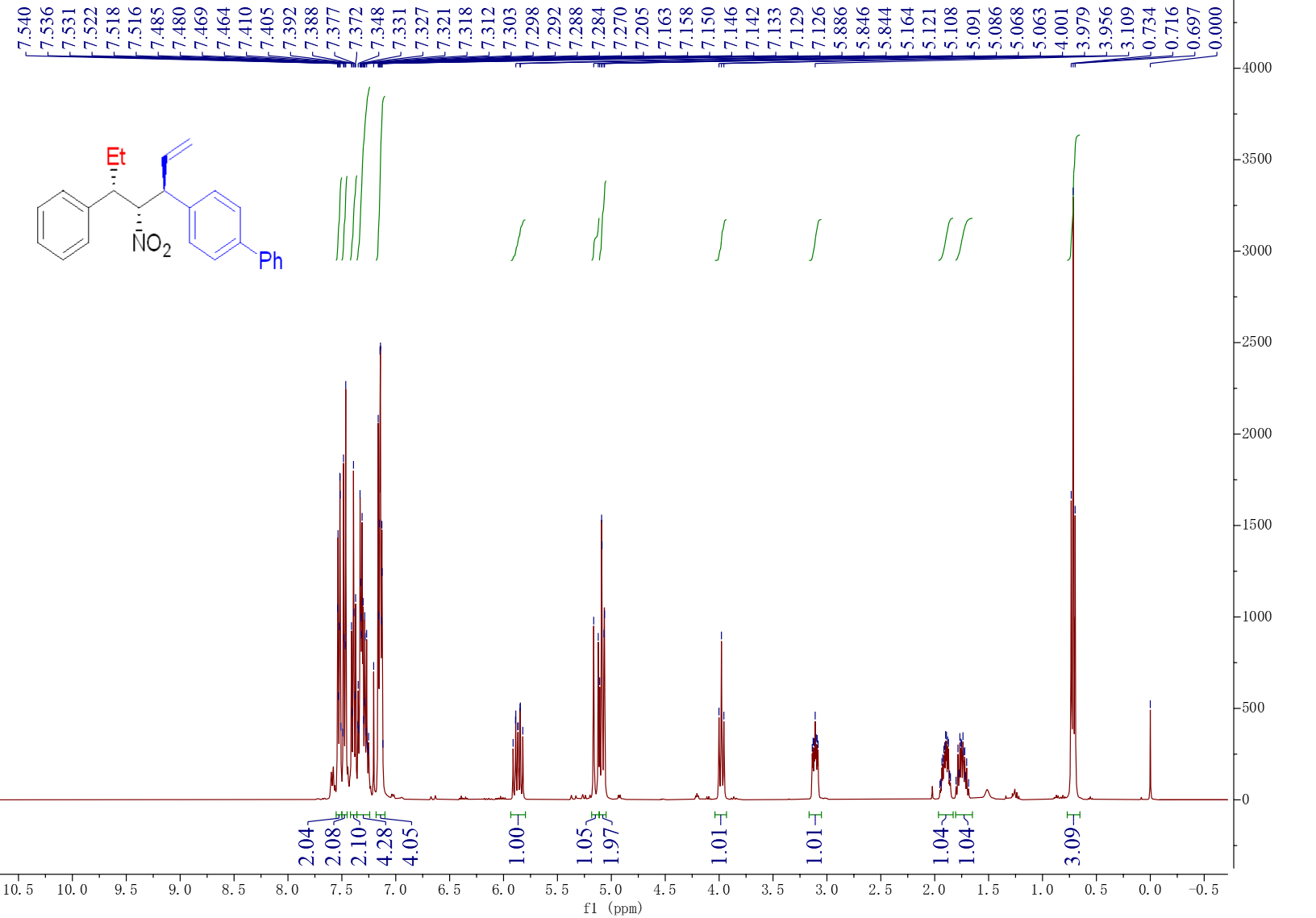


**4k** ^13^C NMR


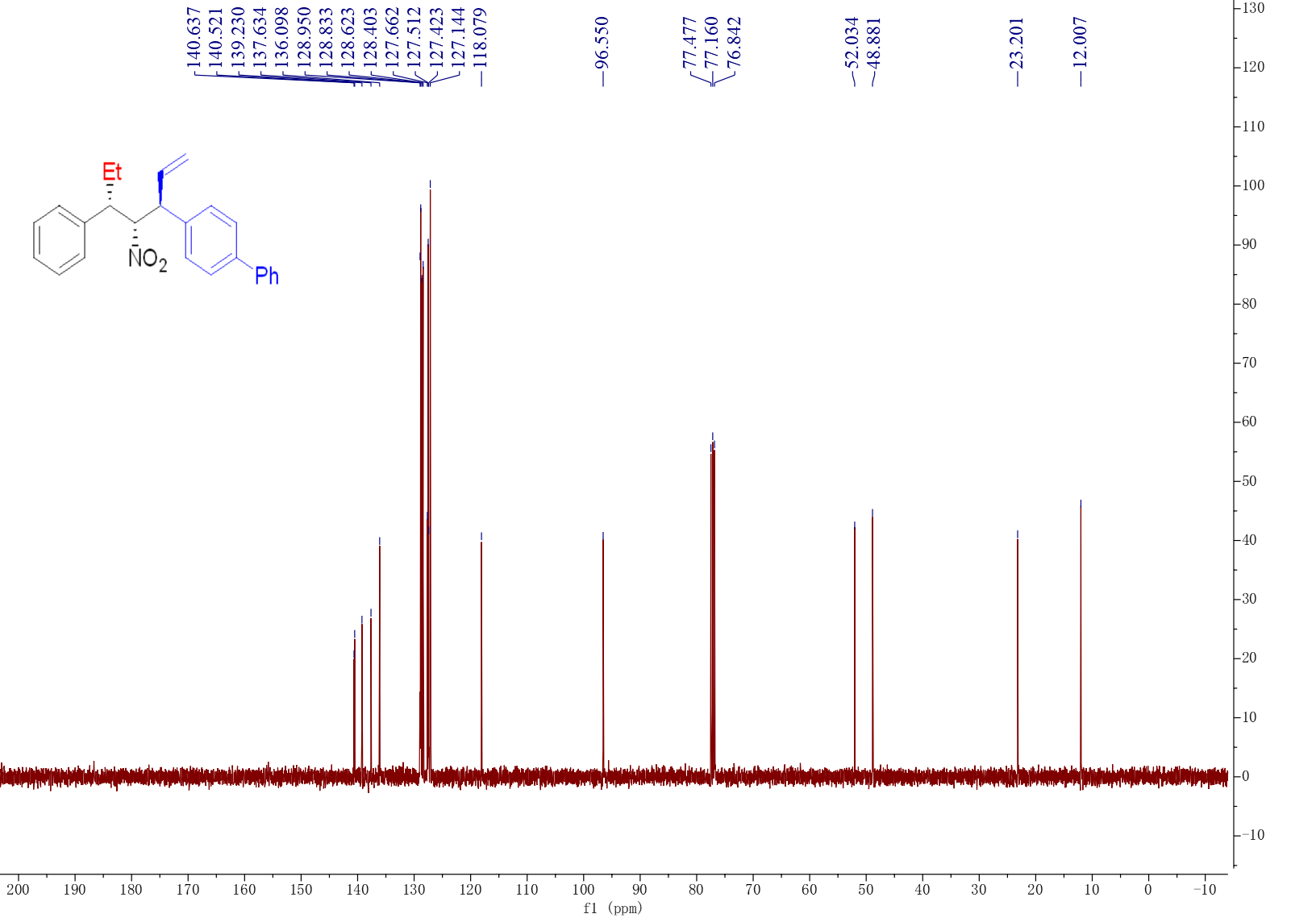


**4l** ^1^H NMR


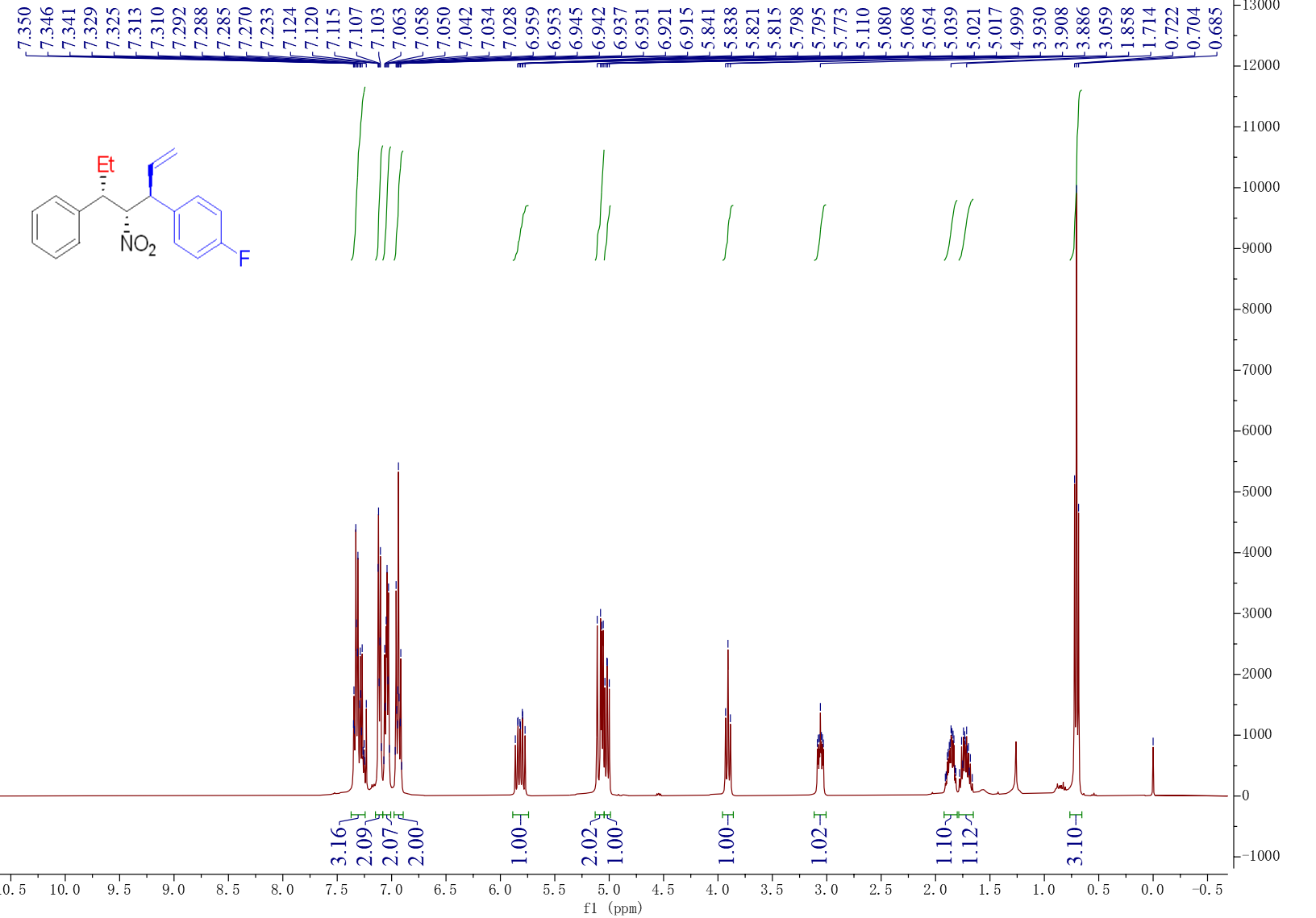


**4l** ^13^C NMR


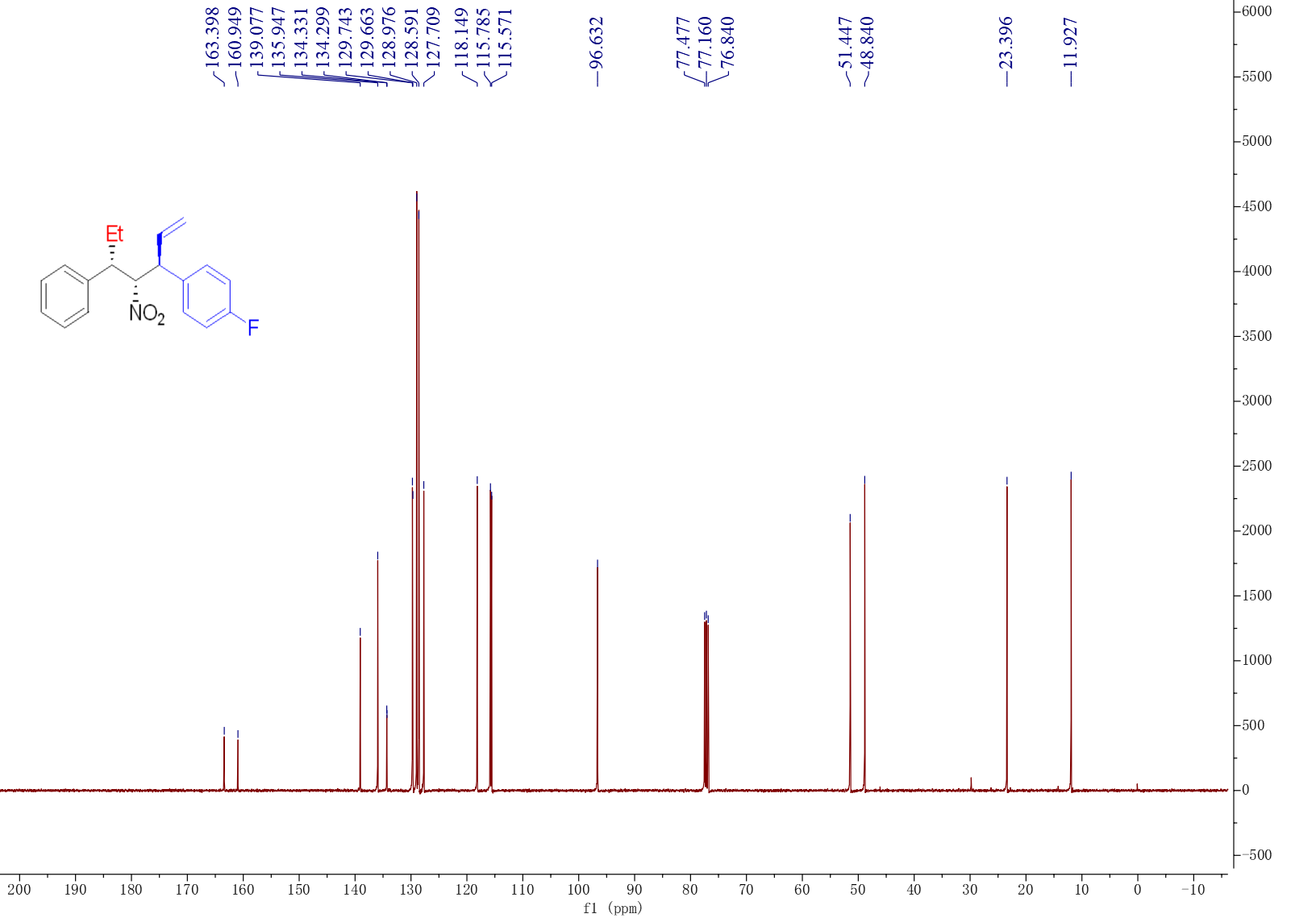


**4l** ^19^F NMR


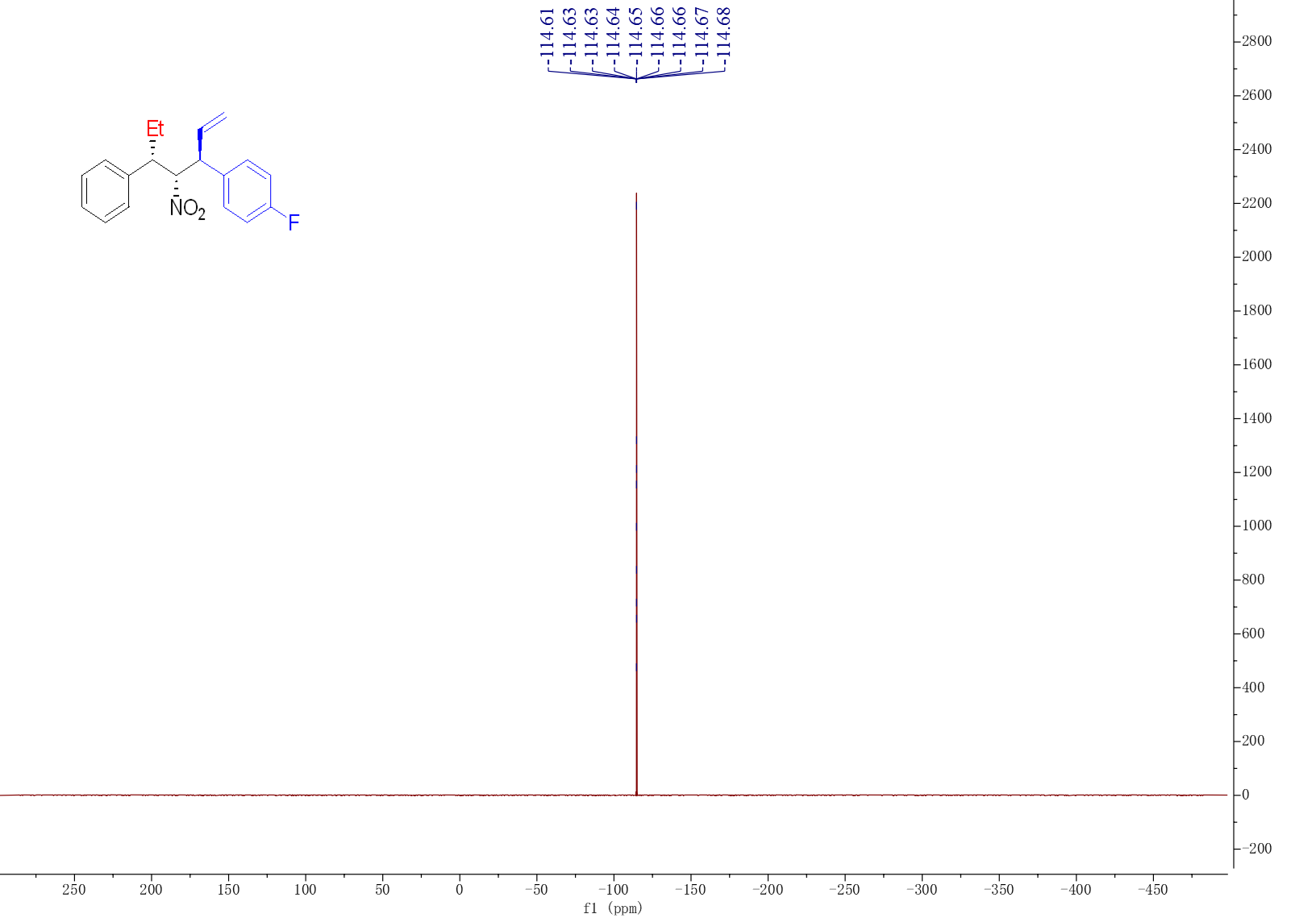


**4m** ^1^H NMR


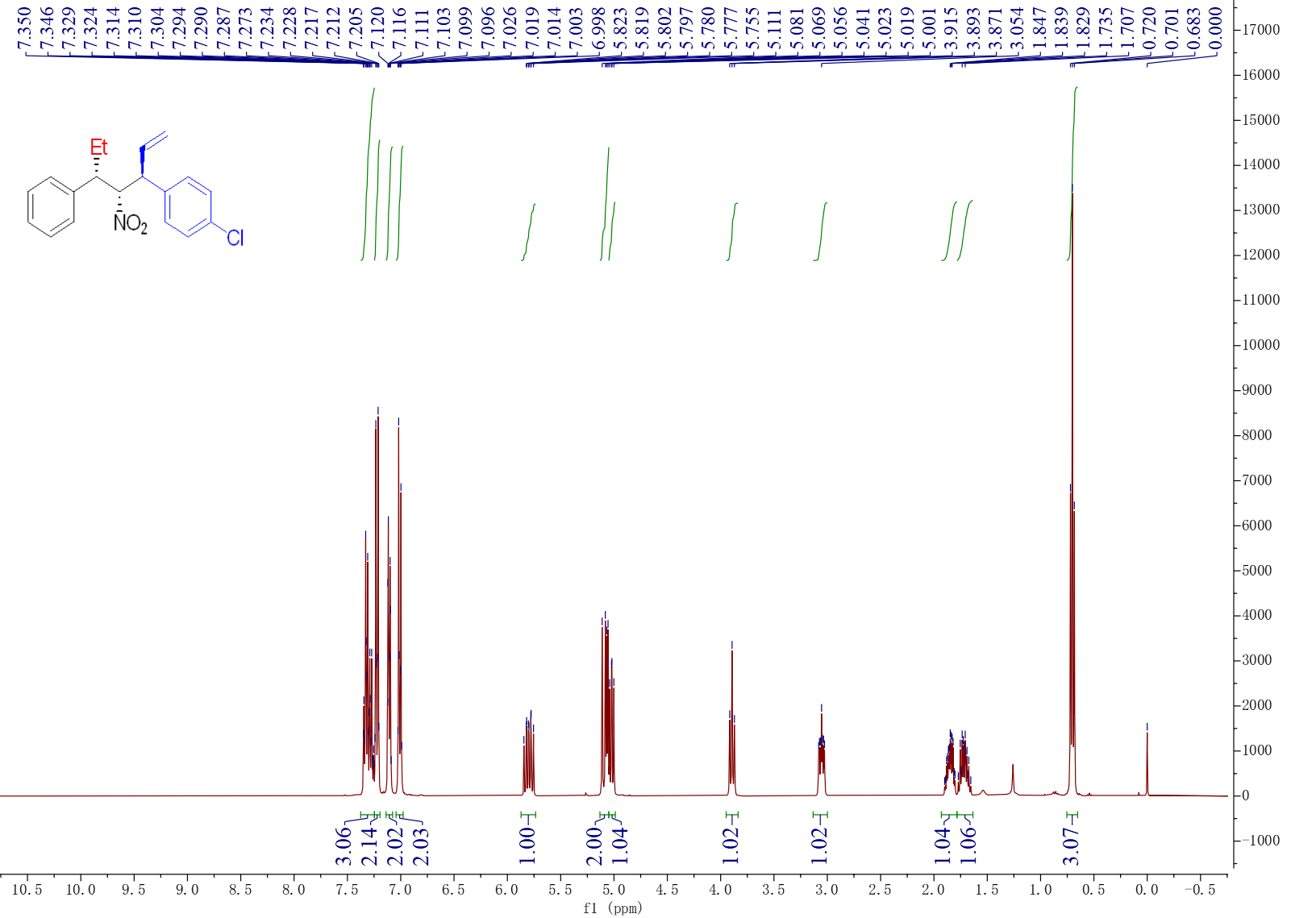


**4m** ^13^C NMR


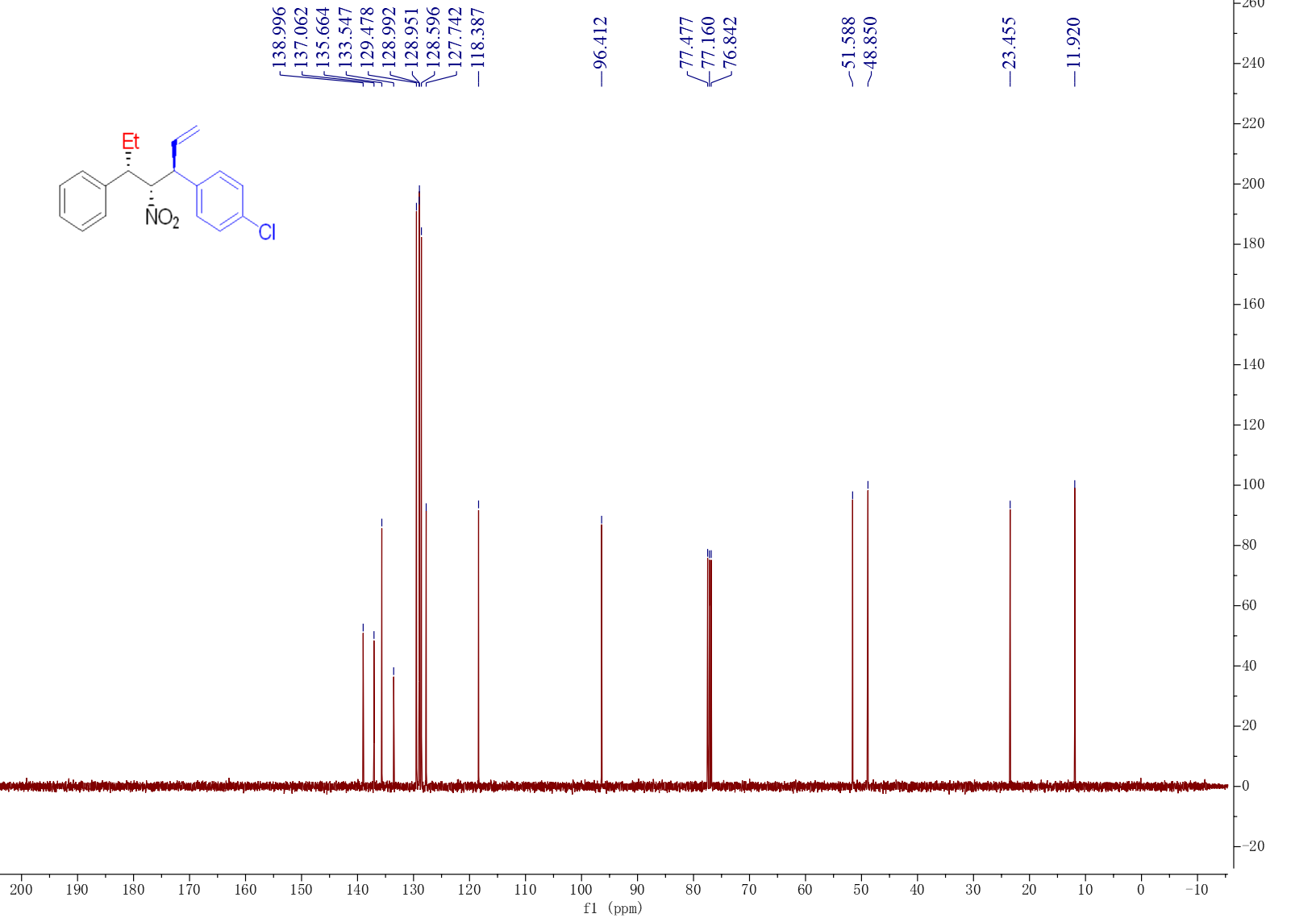


**4n** ^1^H NMR


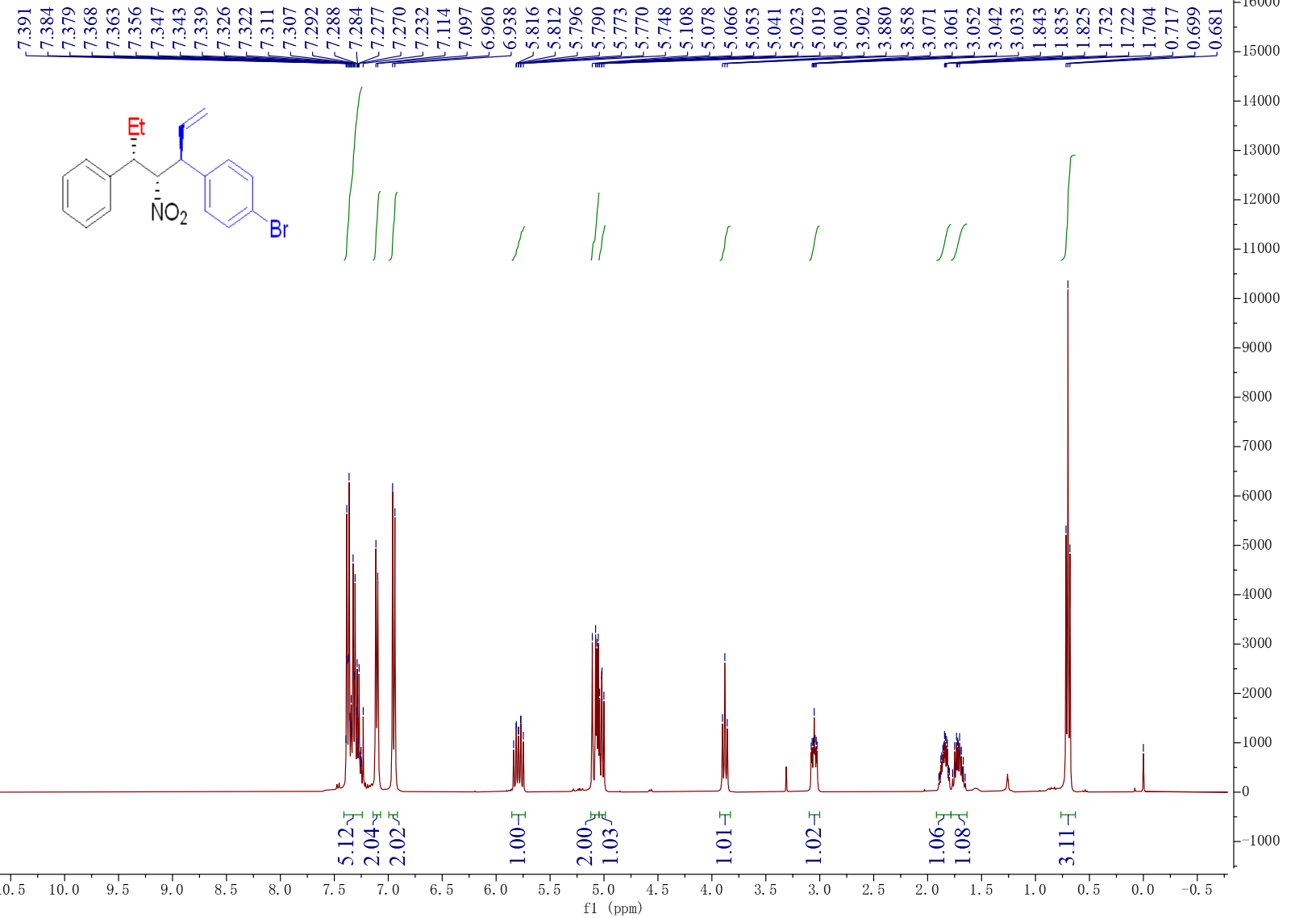


**4n** ^13^C NMR


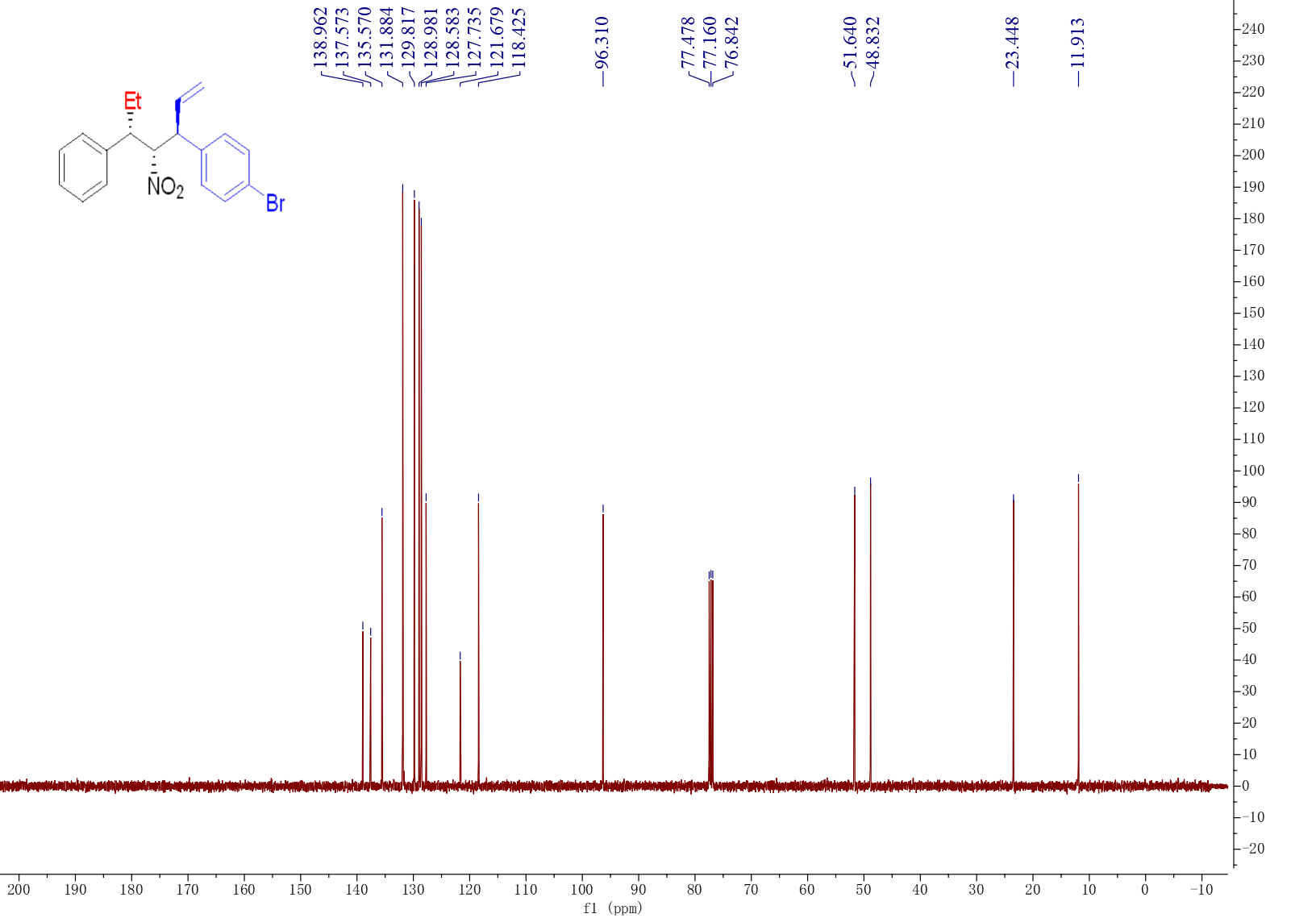


**4o** ^1^H NMR


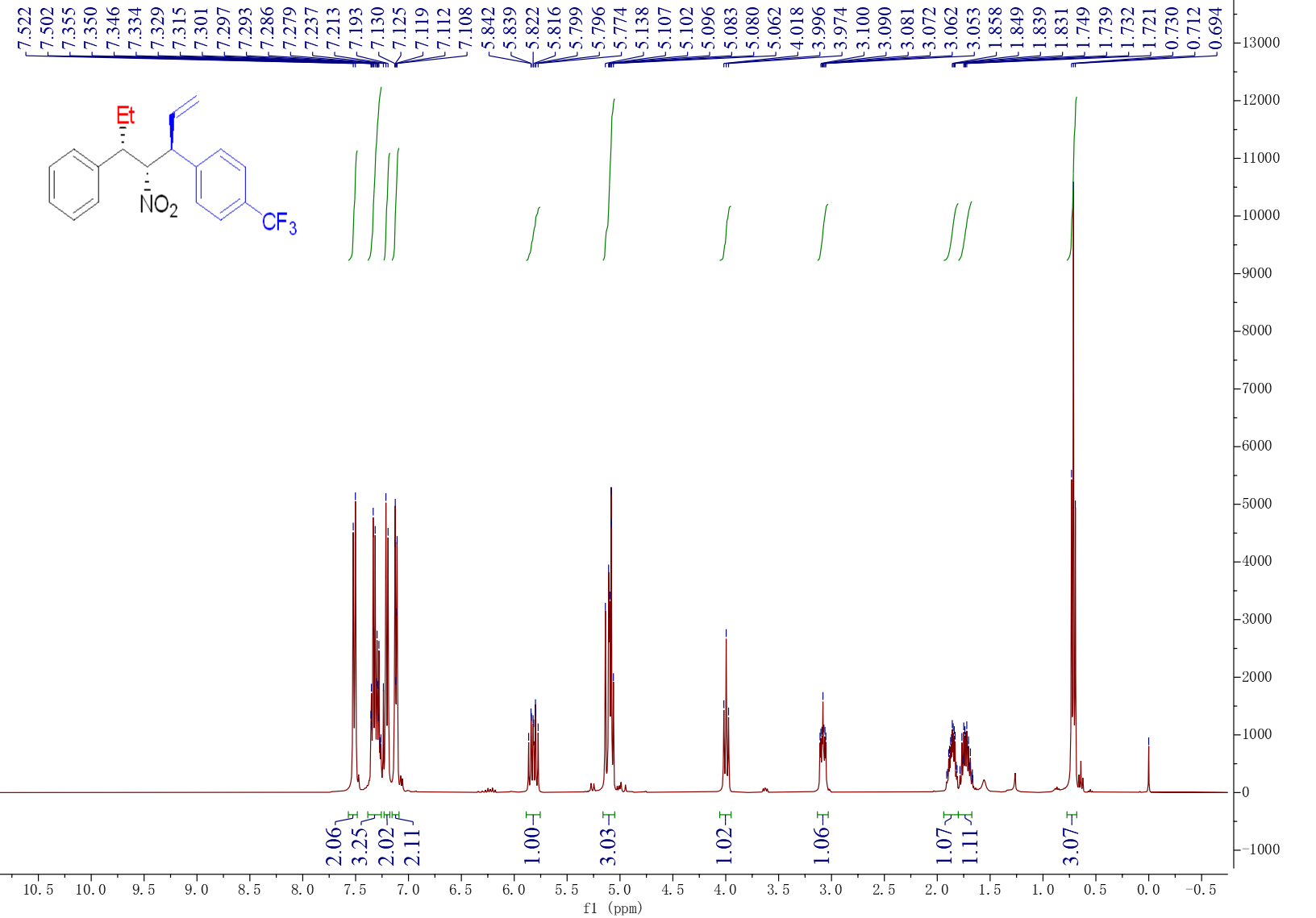


**4o** ^13^C NMR


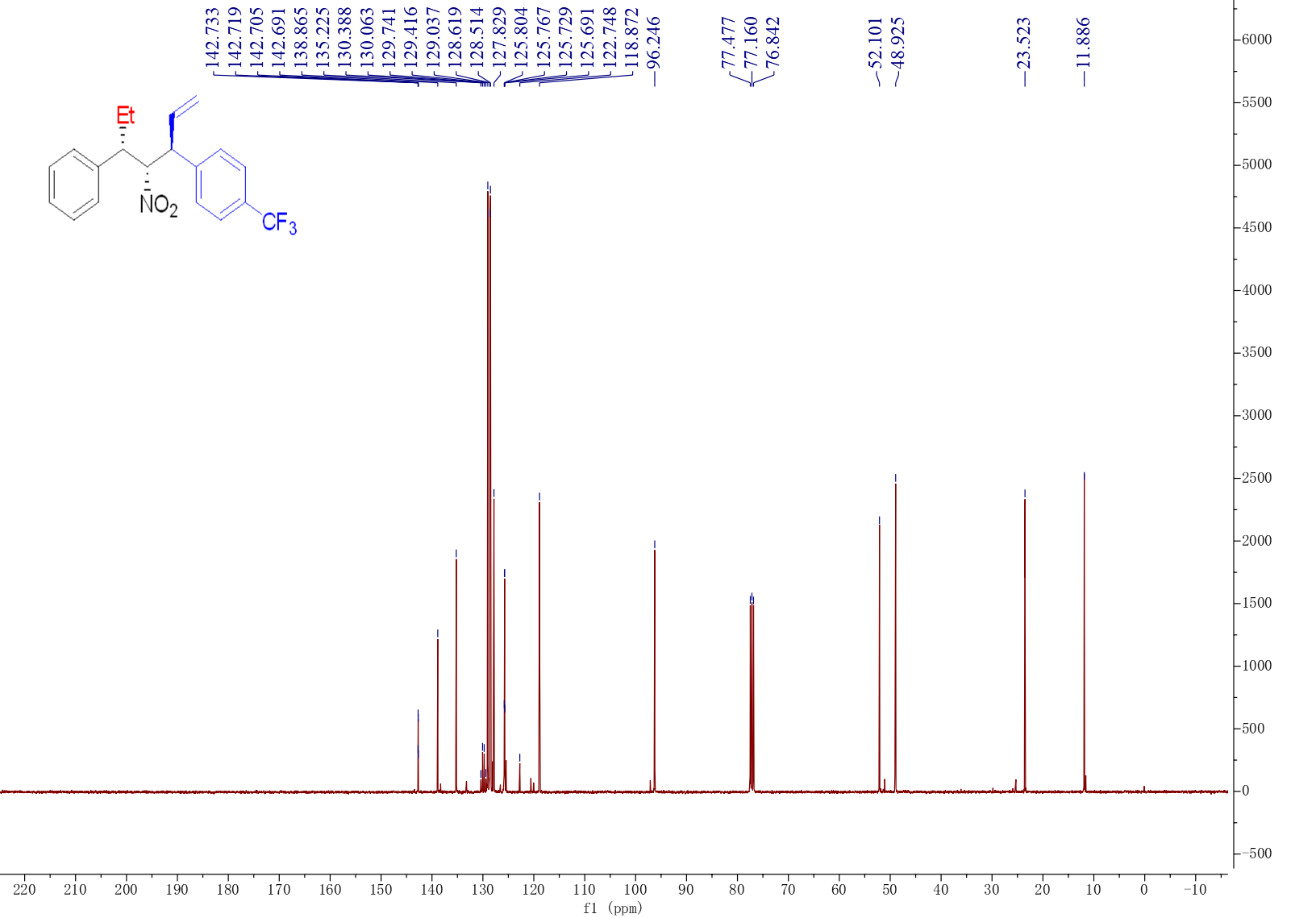


**4o** ^19^F NMR


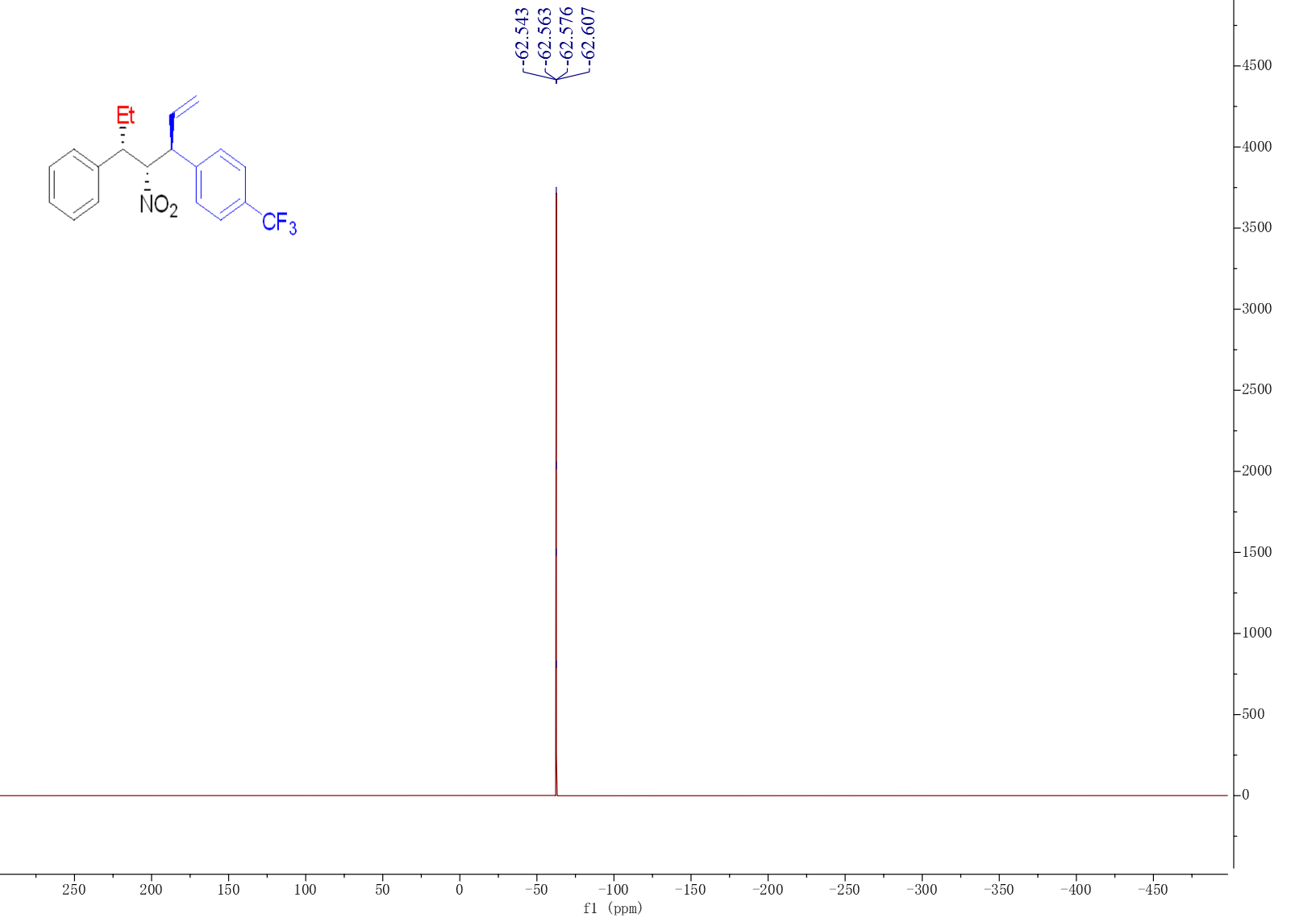


**4p** ^1^H NMR


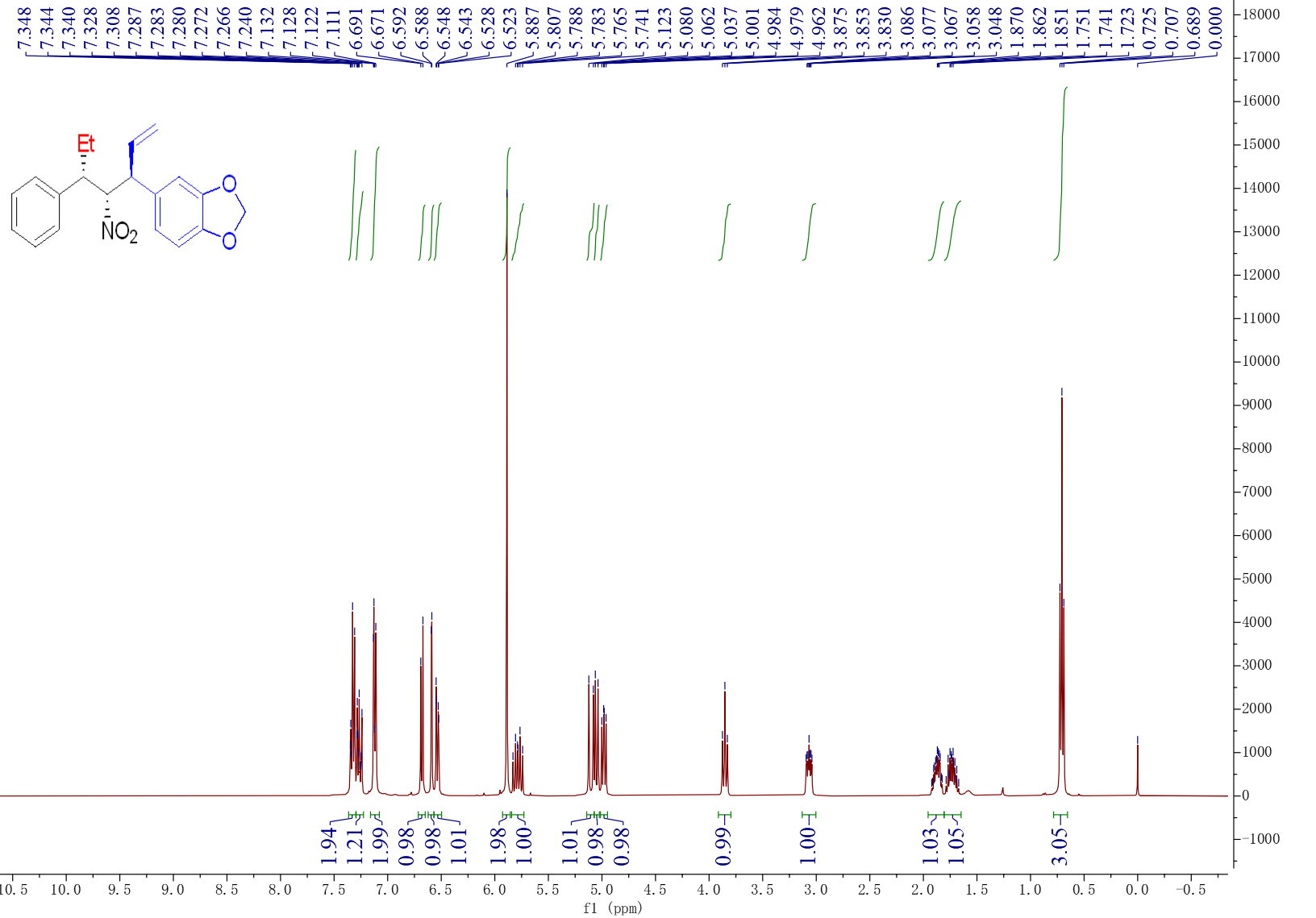


**4p** ^13^C NMR


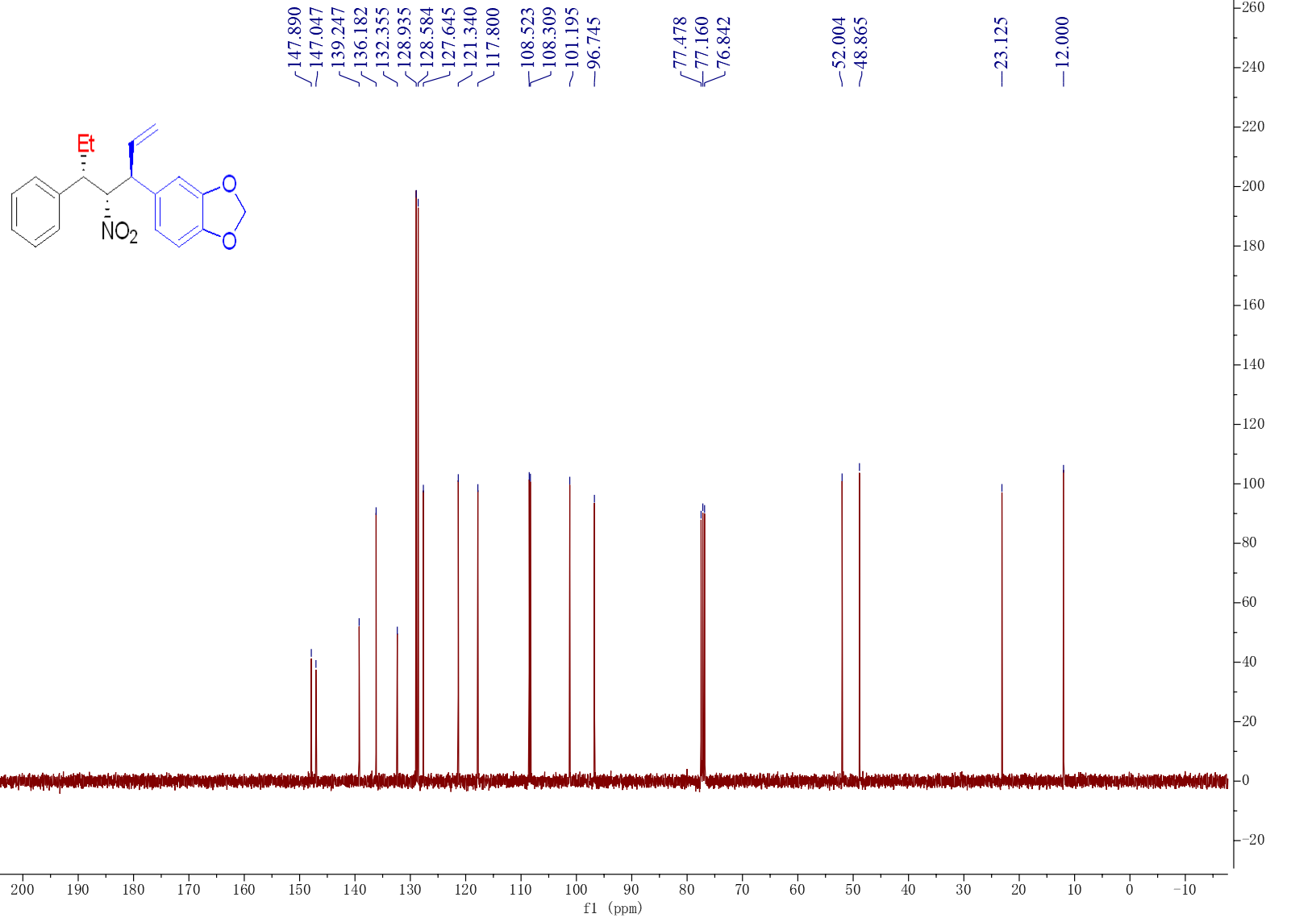


**4q** ^1^H NMR


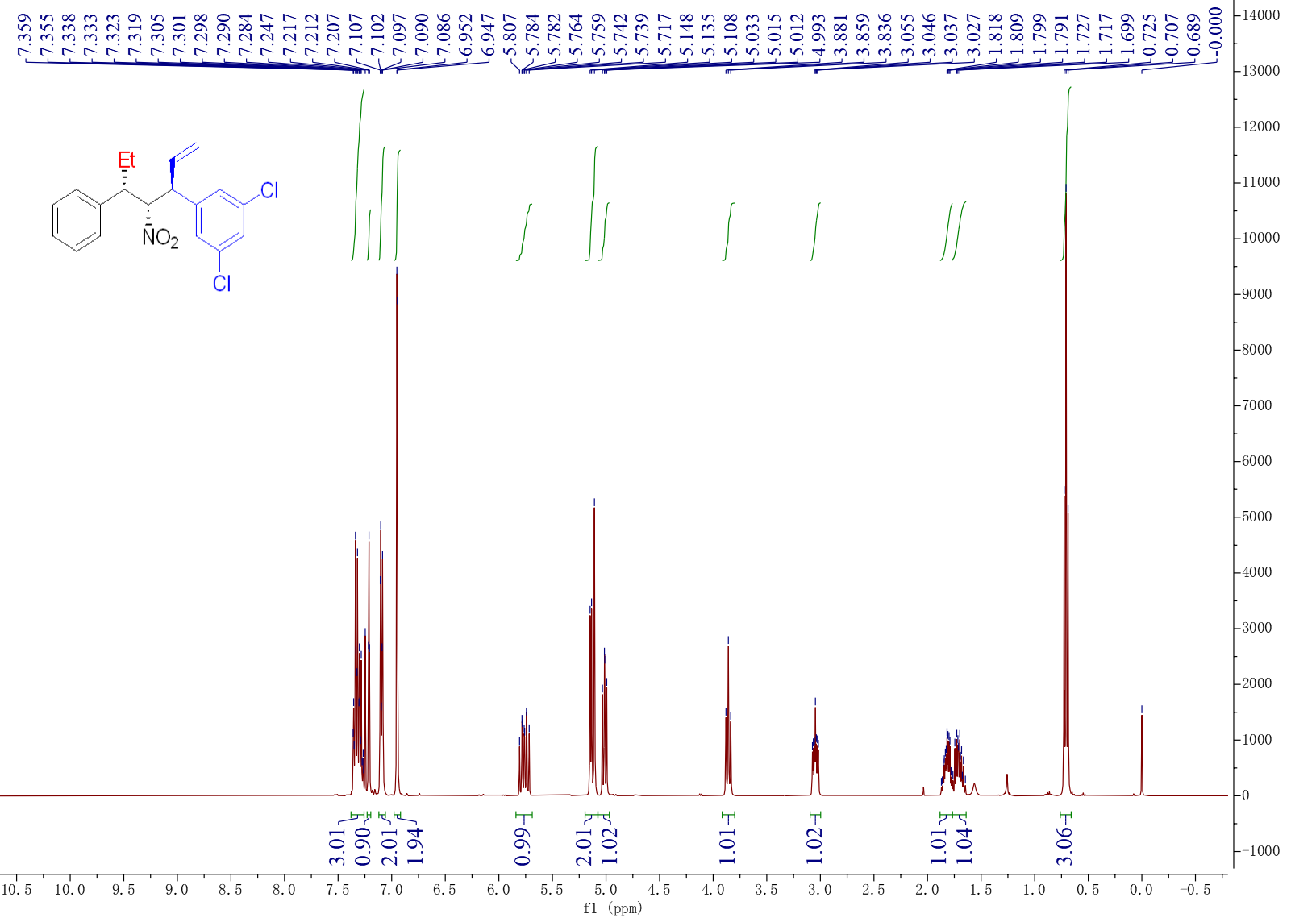


**4q** ^13^C NMR


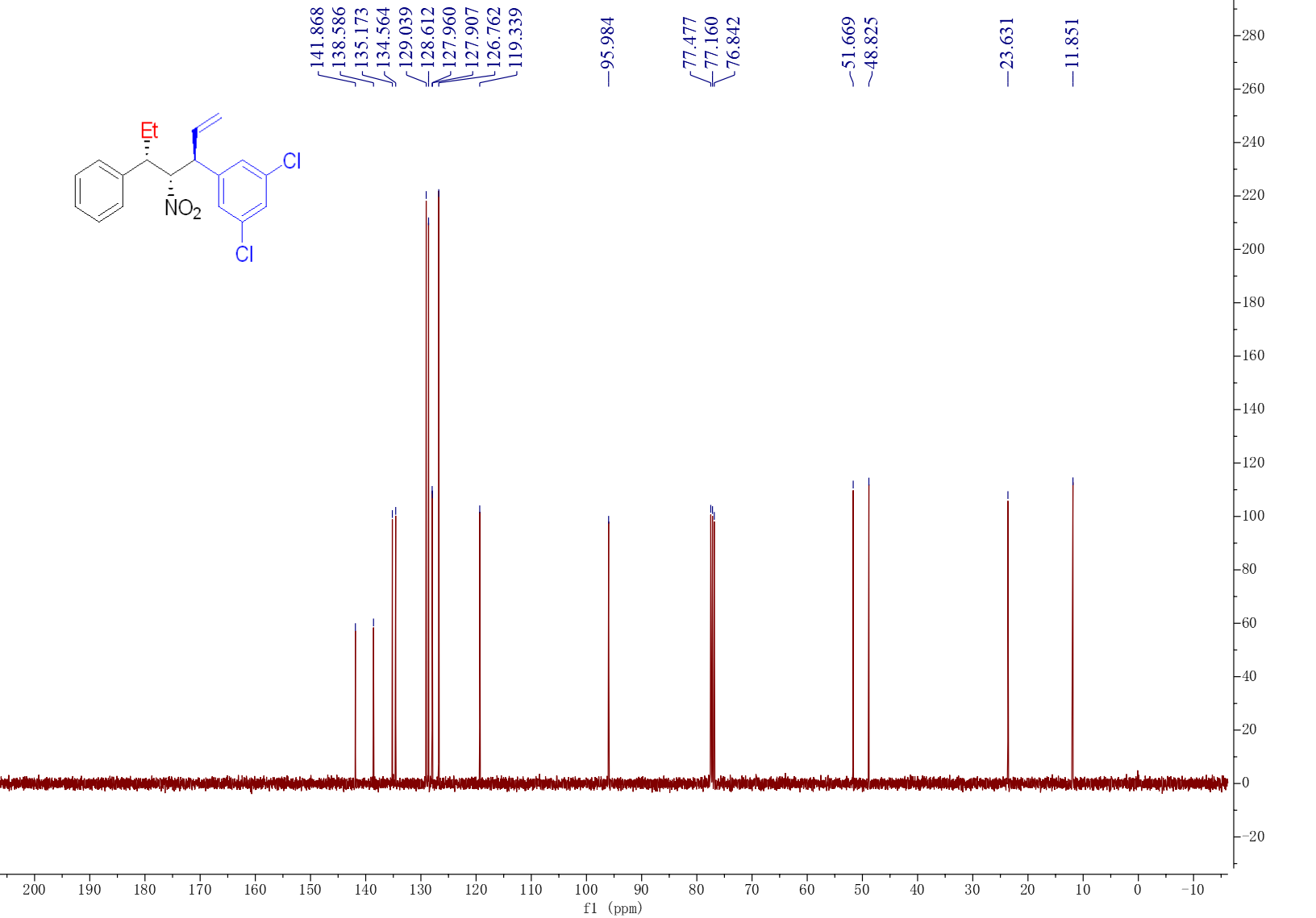


**4r** ^1^H NMR


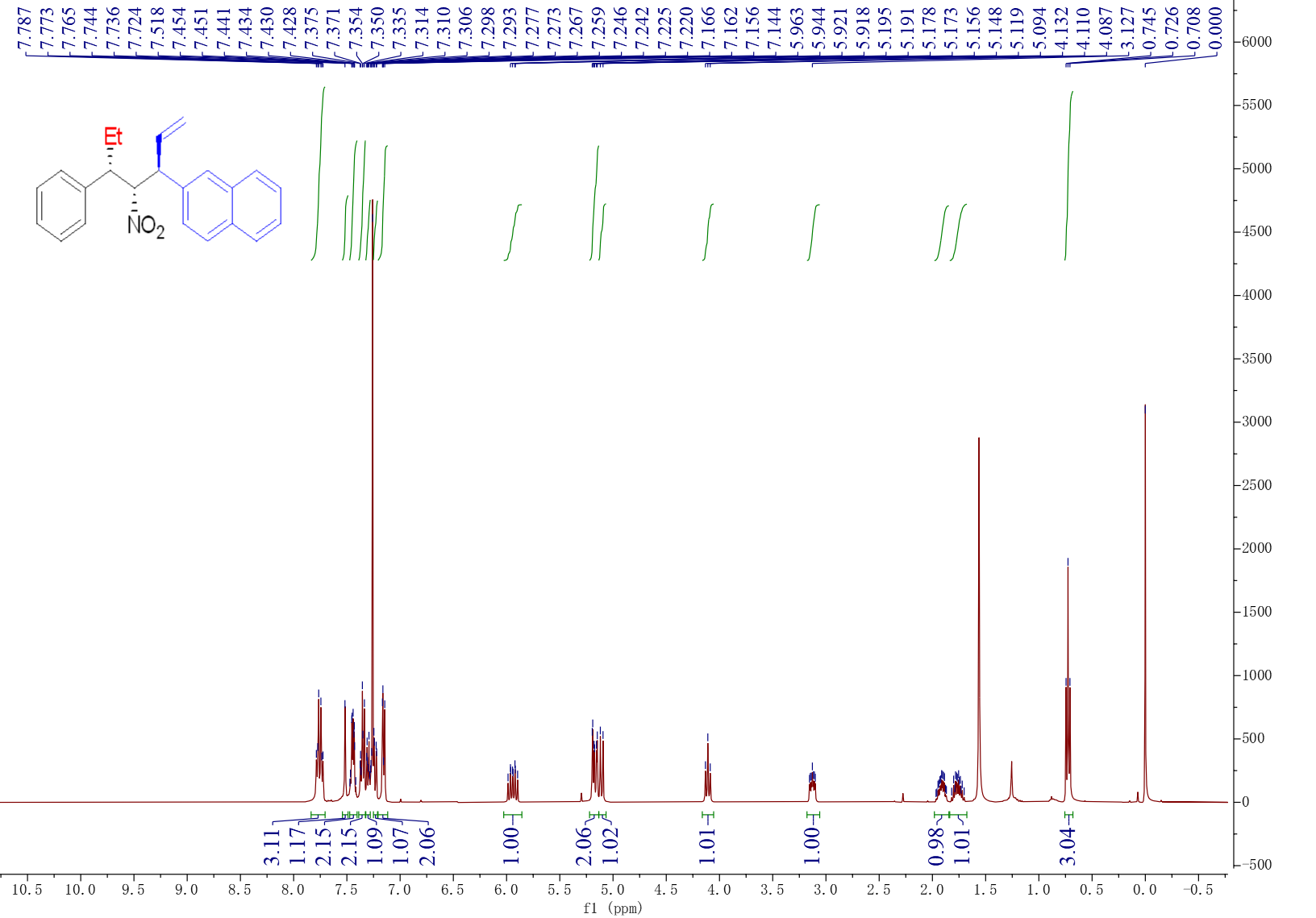


**4r** ^13^C NMR


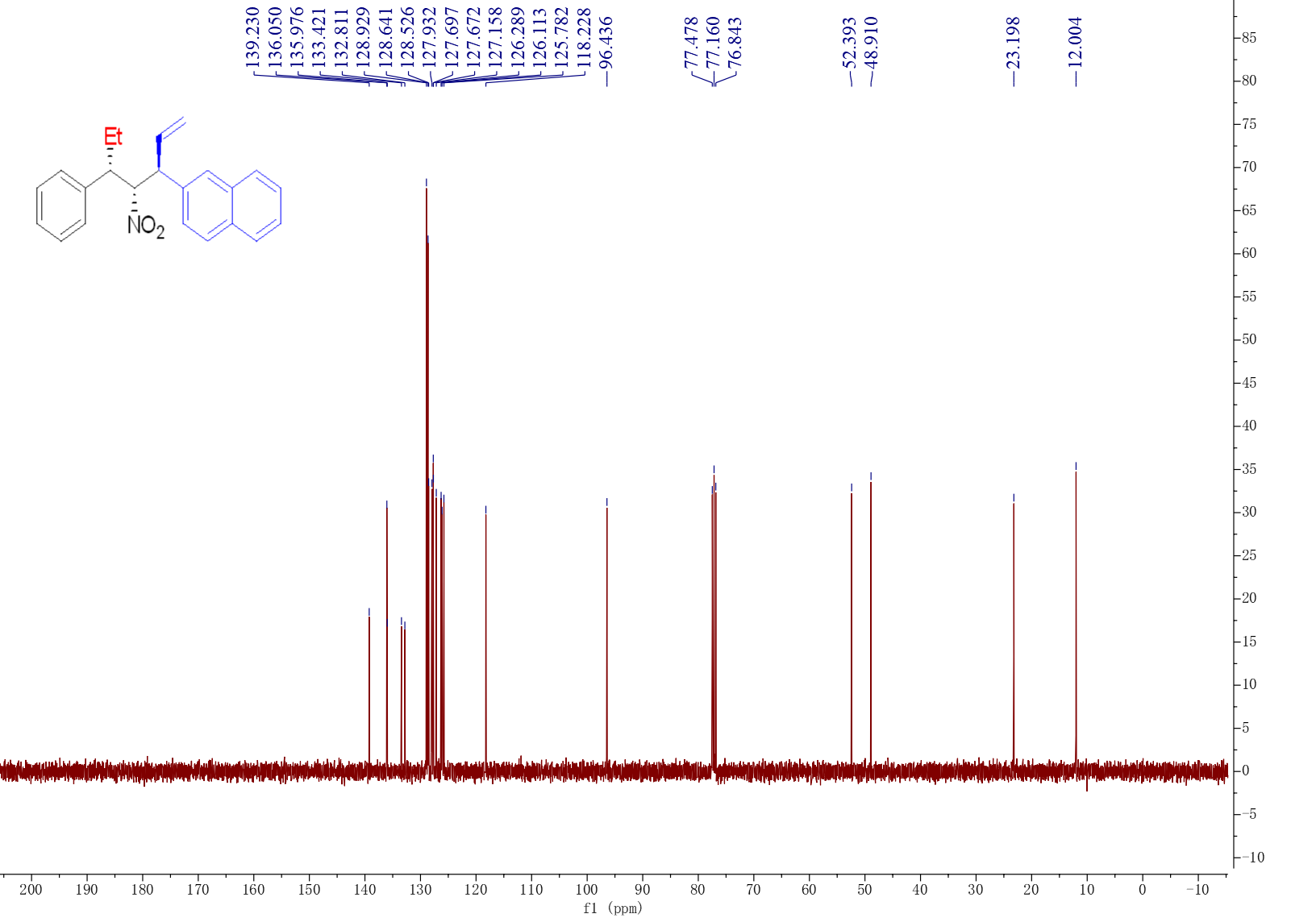


**4s** ^1^H NMR


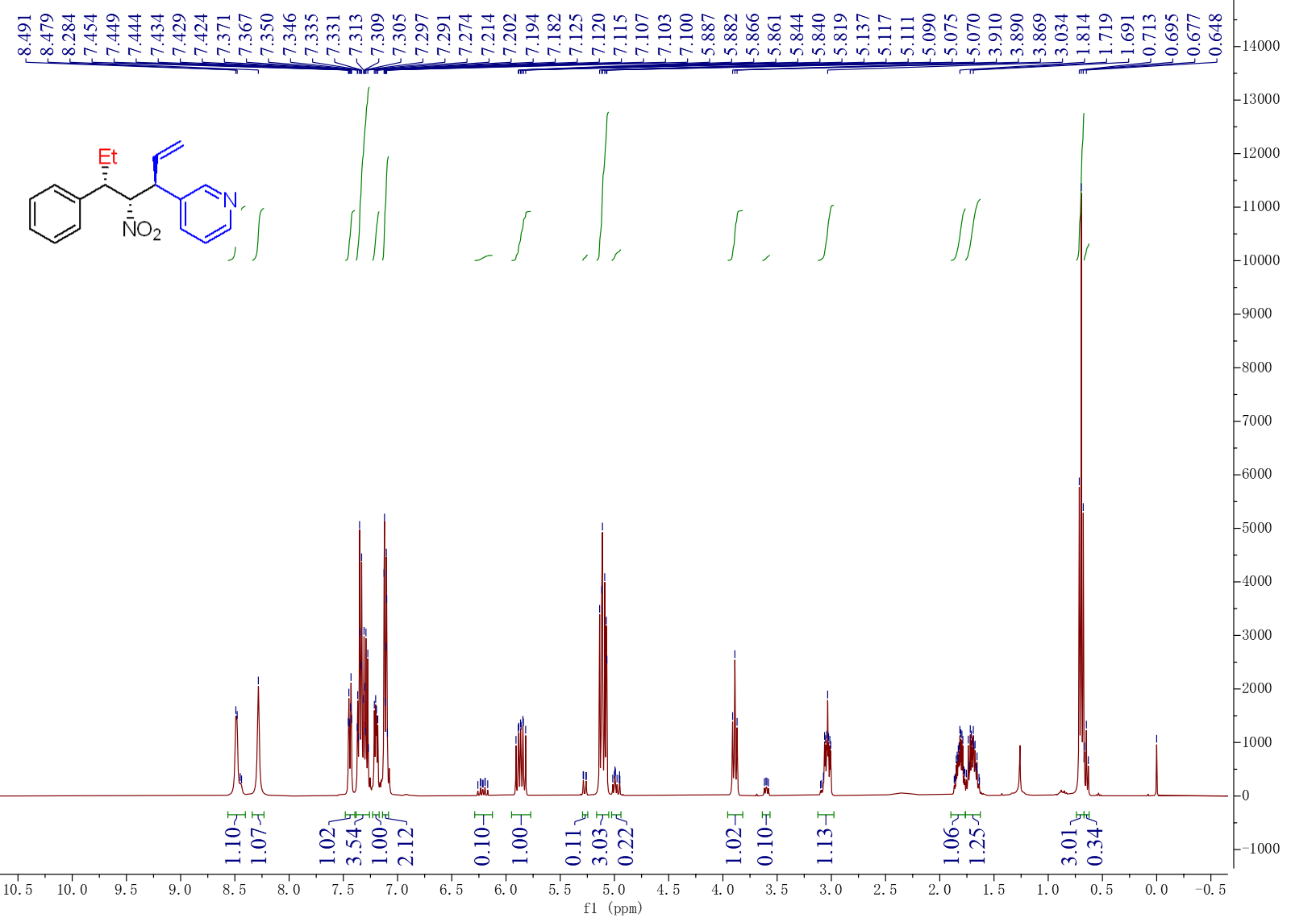


**4s** ^13^C NMR


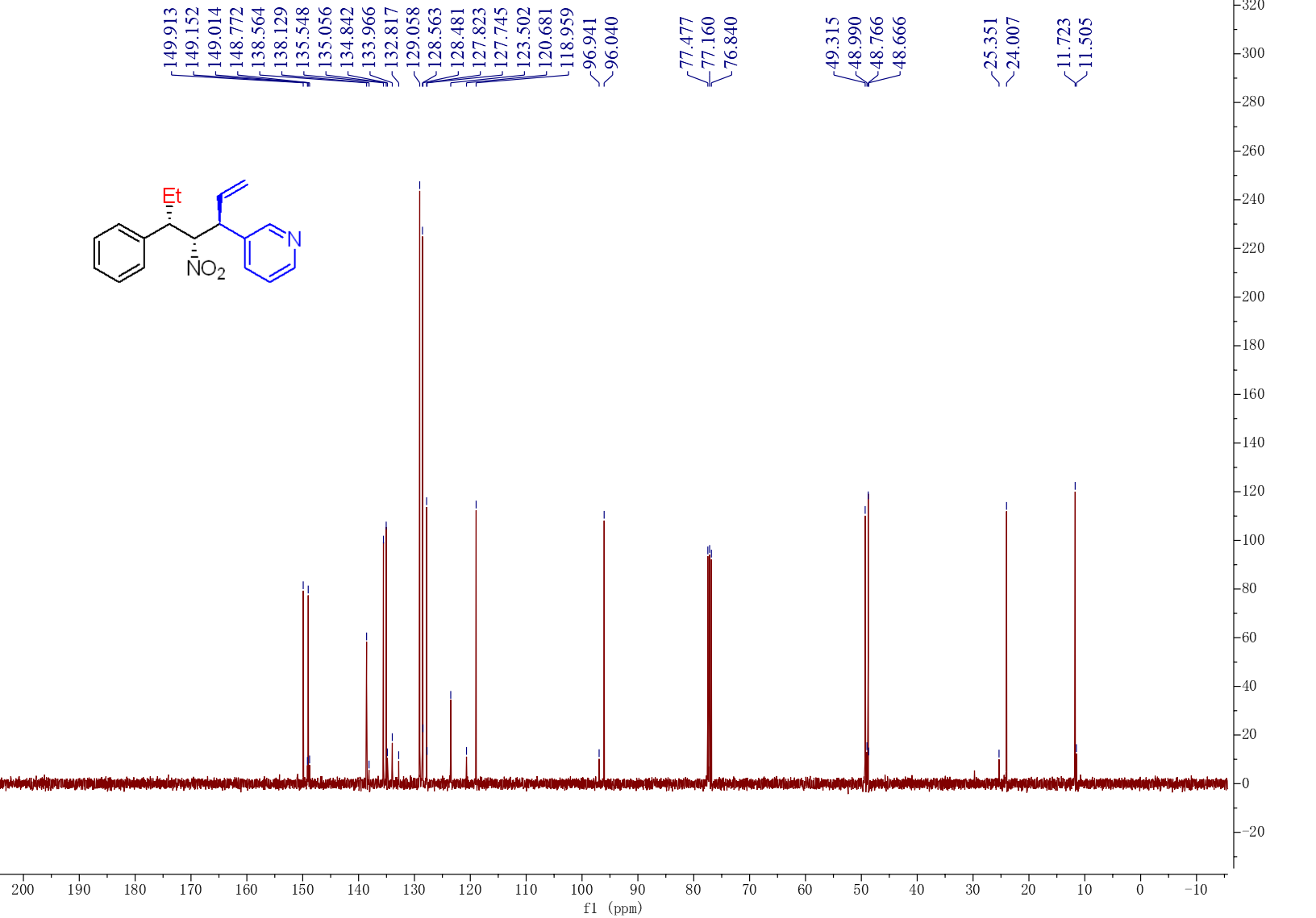


**4t** ^1^H NMR


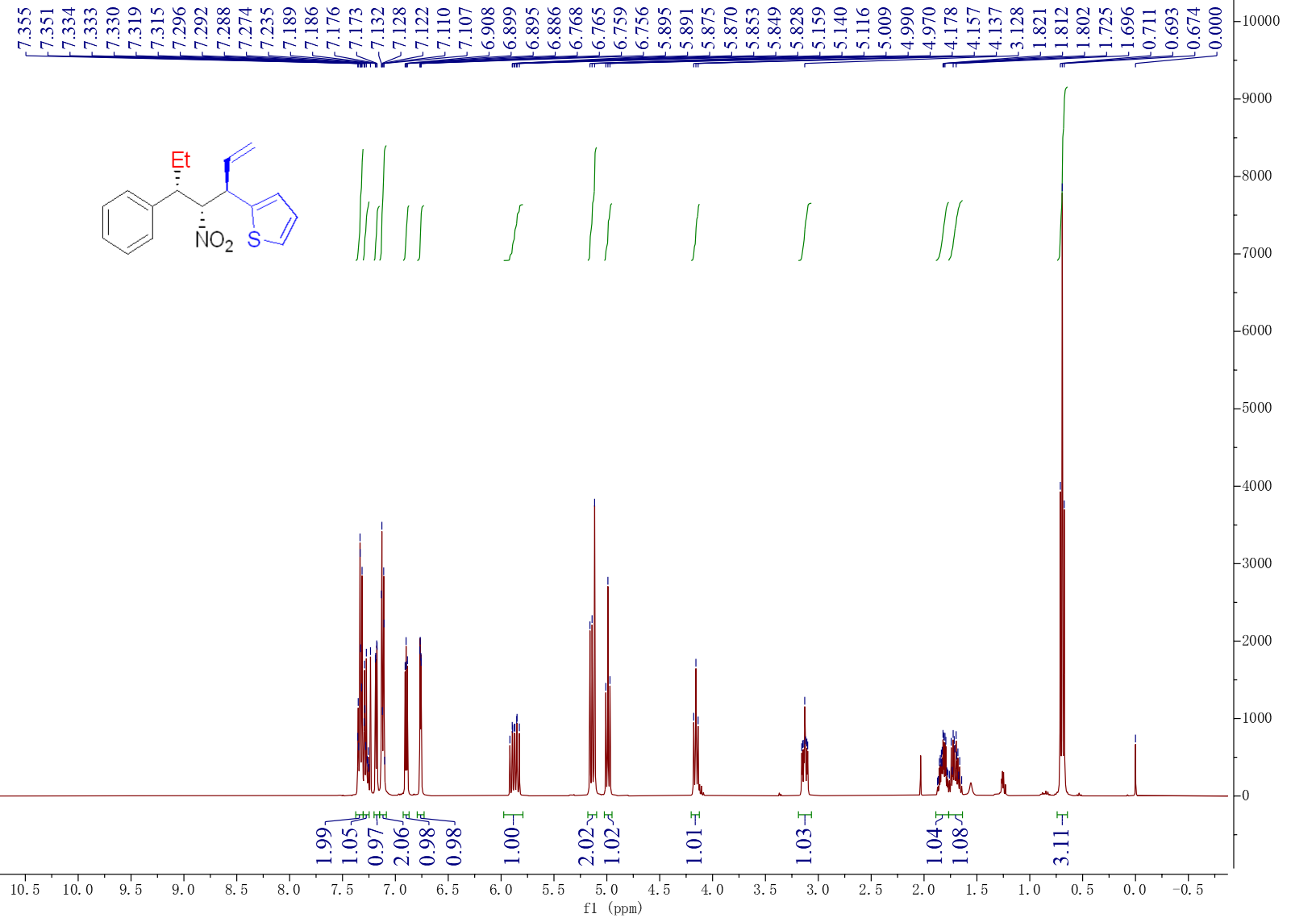


**4t** ^13^C NMR


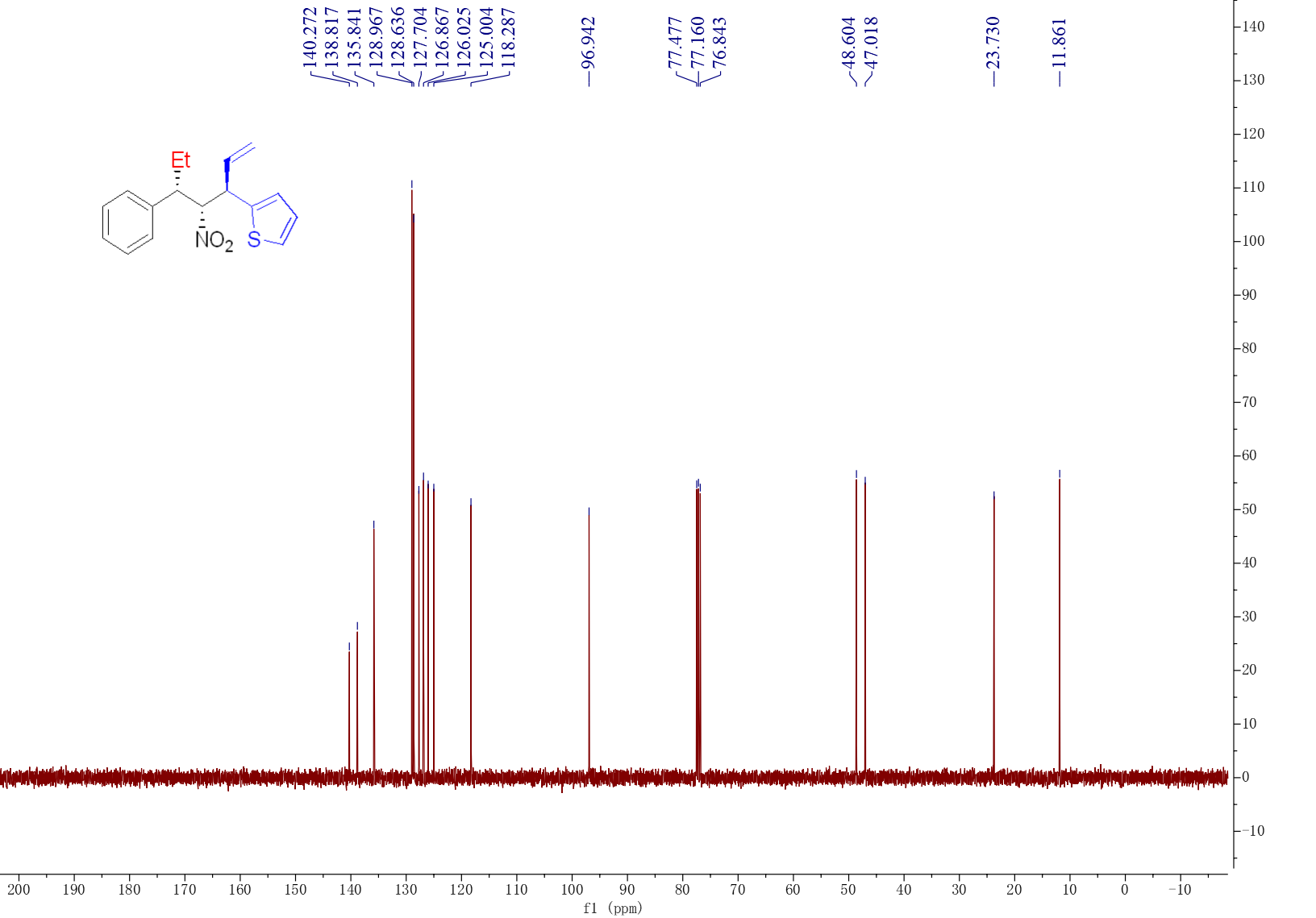


**5a** ^1^H NMR


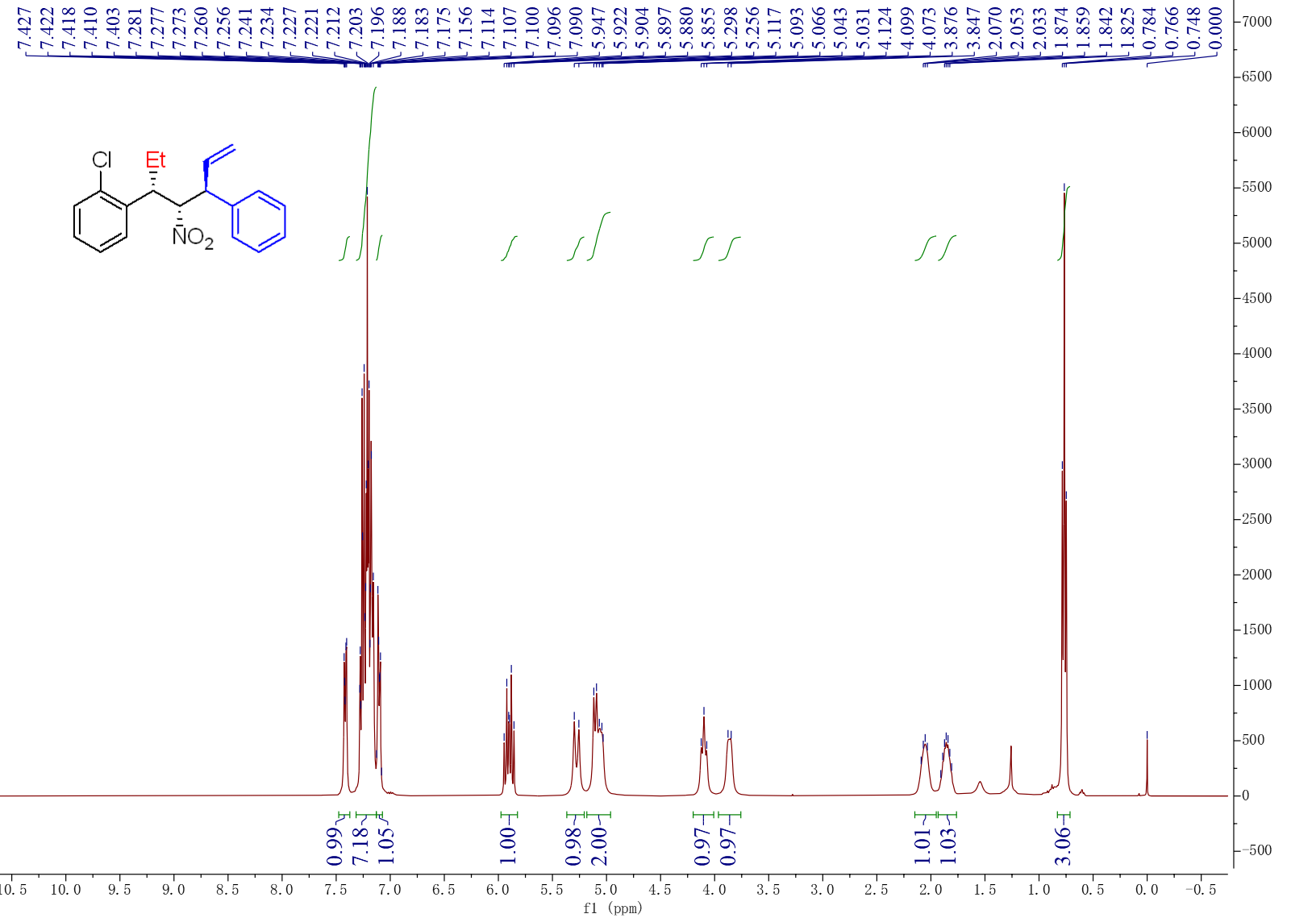


**5a** ^13^C NMR


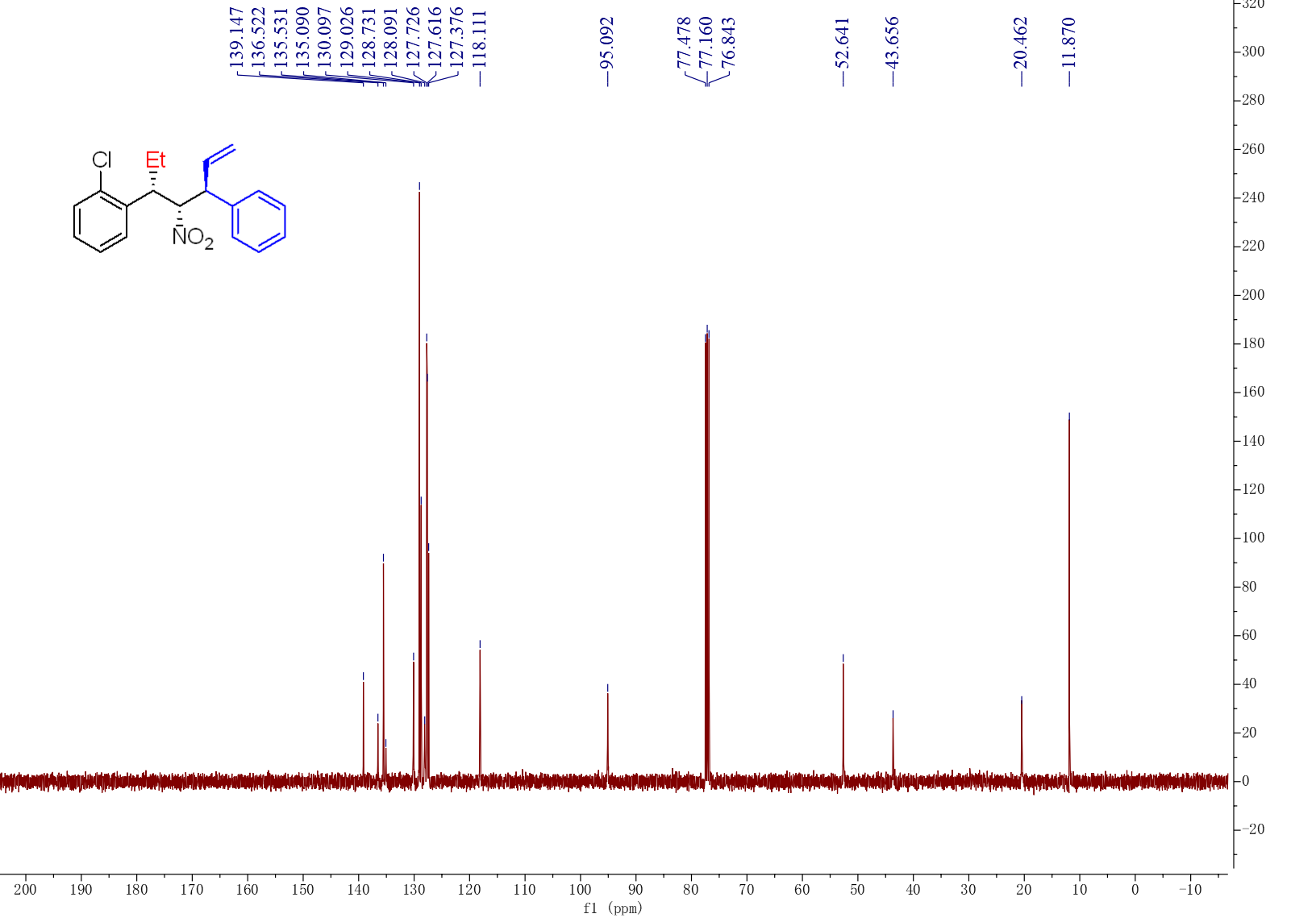


**5b** ^1^H NMR


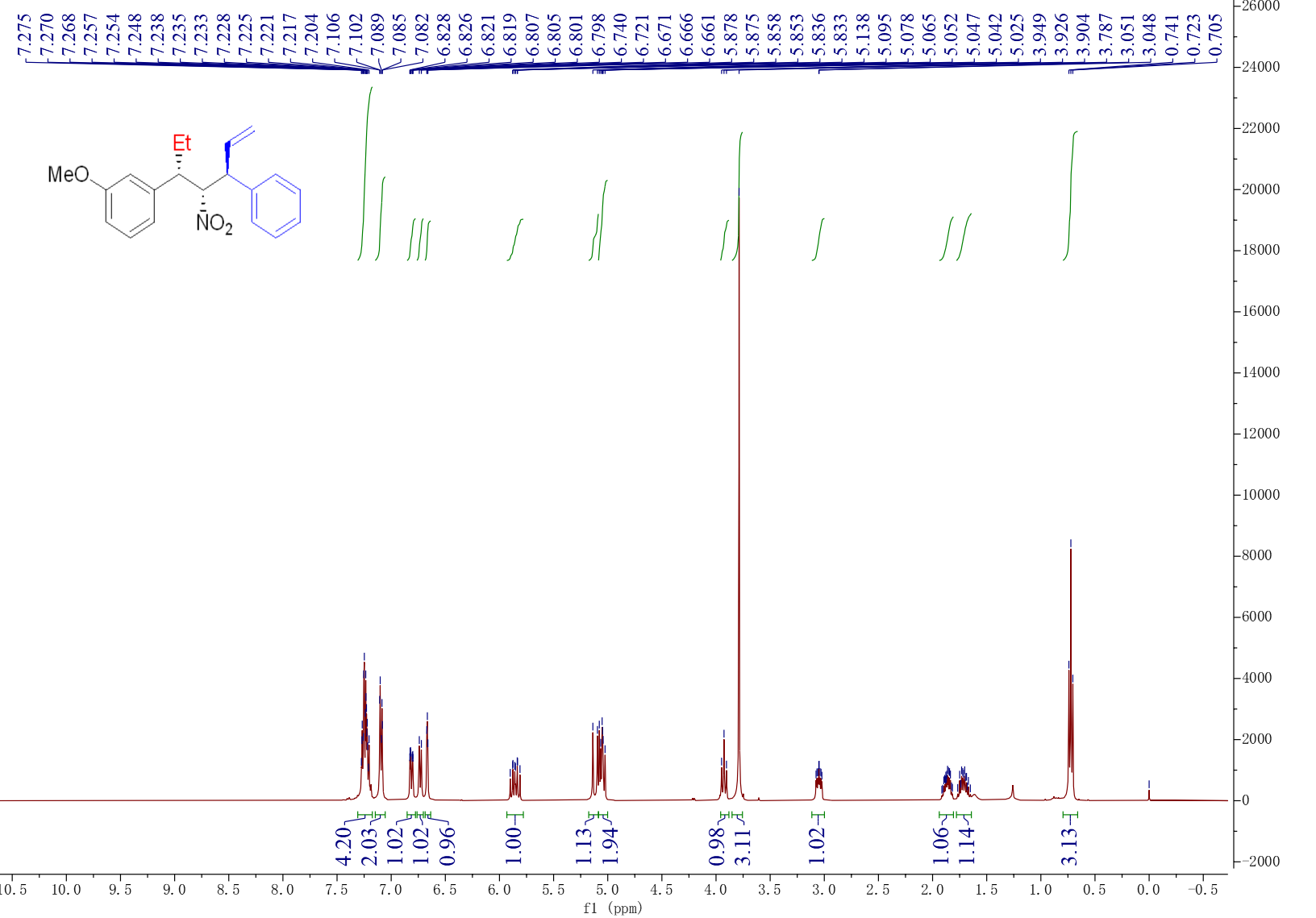


**5b** ^13^C NMR


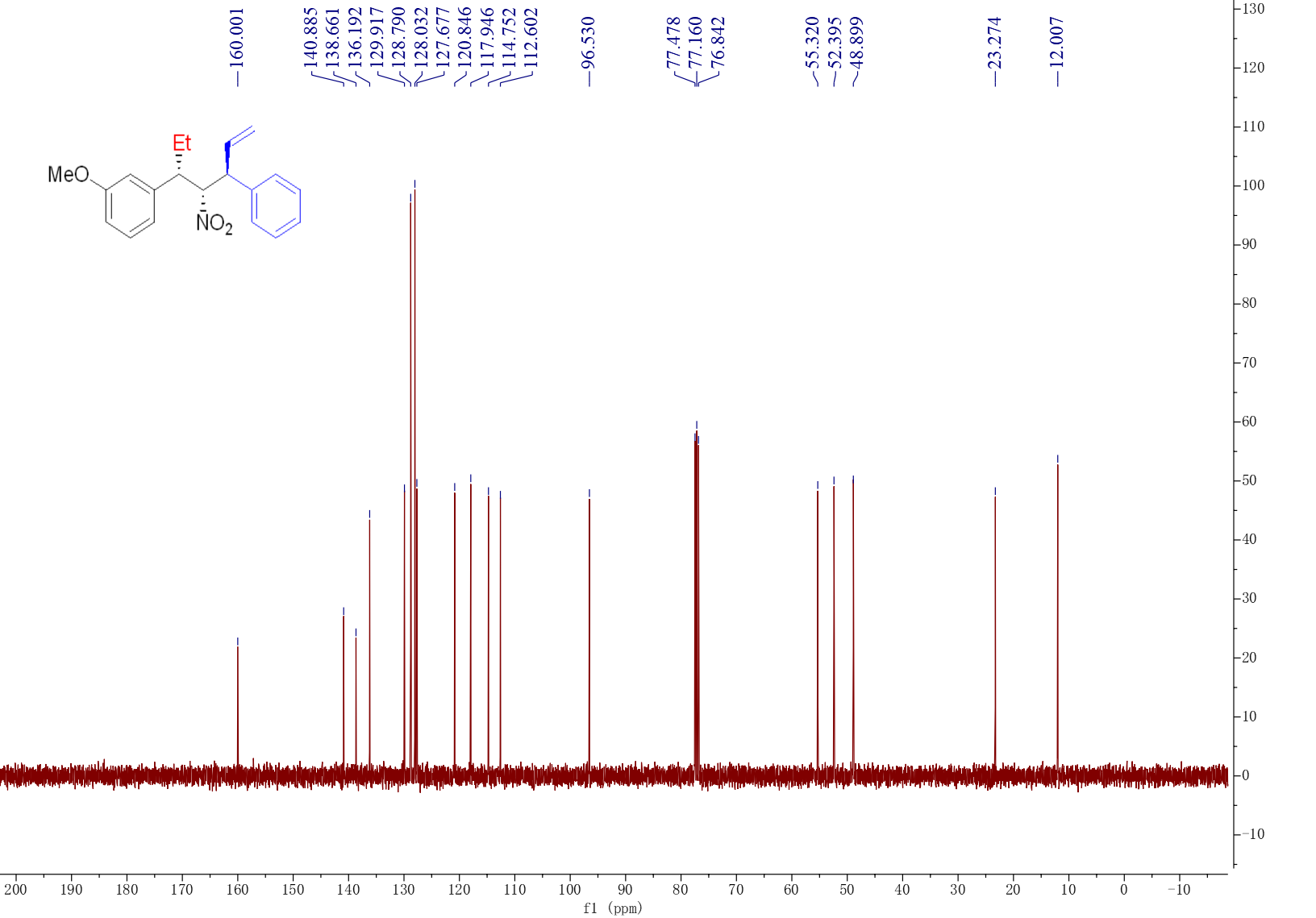


**5c** ^1^H NMR


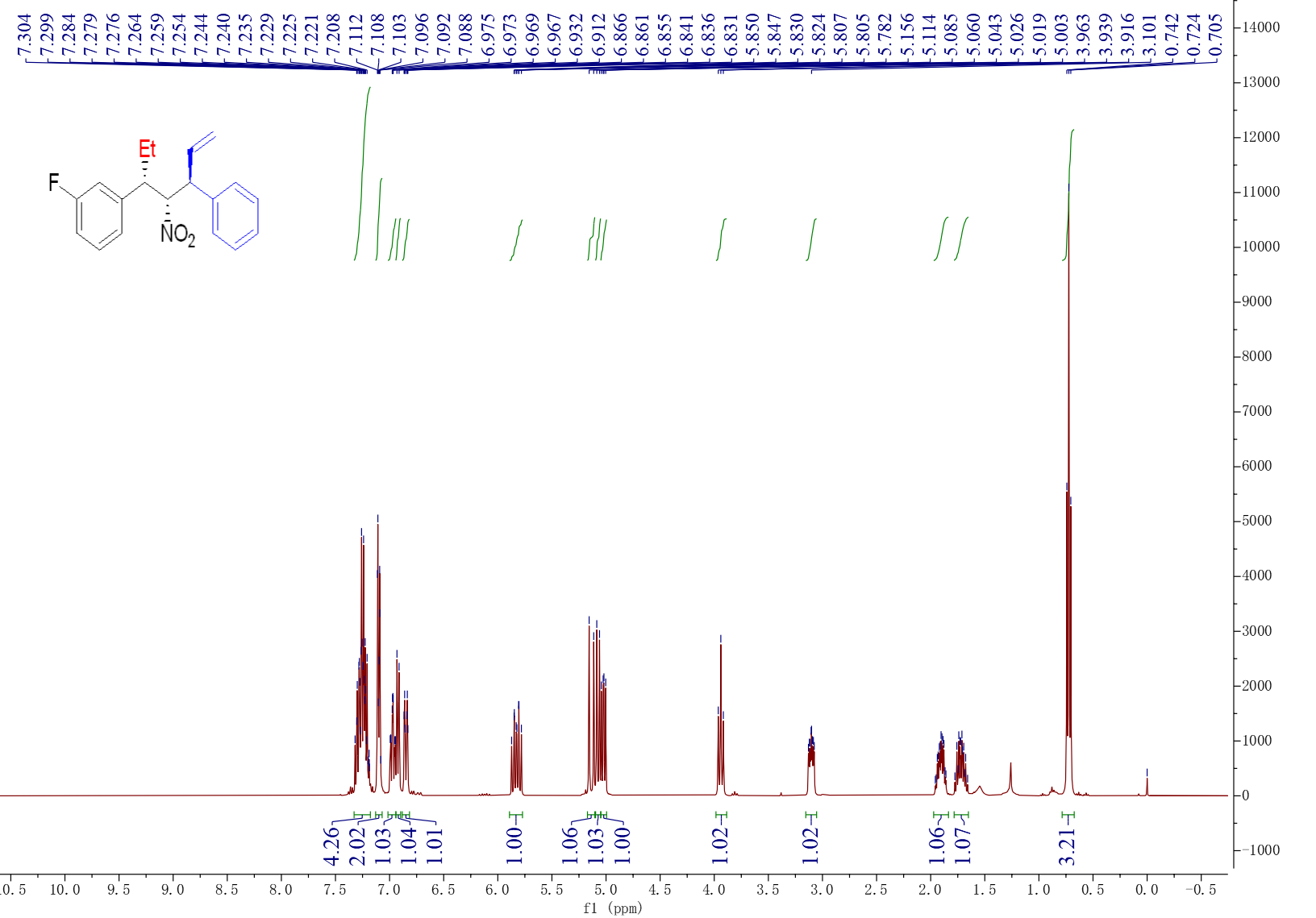


**5c** ^13^C NMR


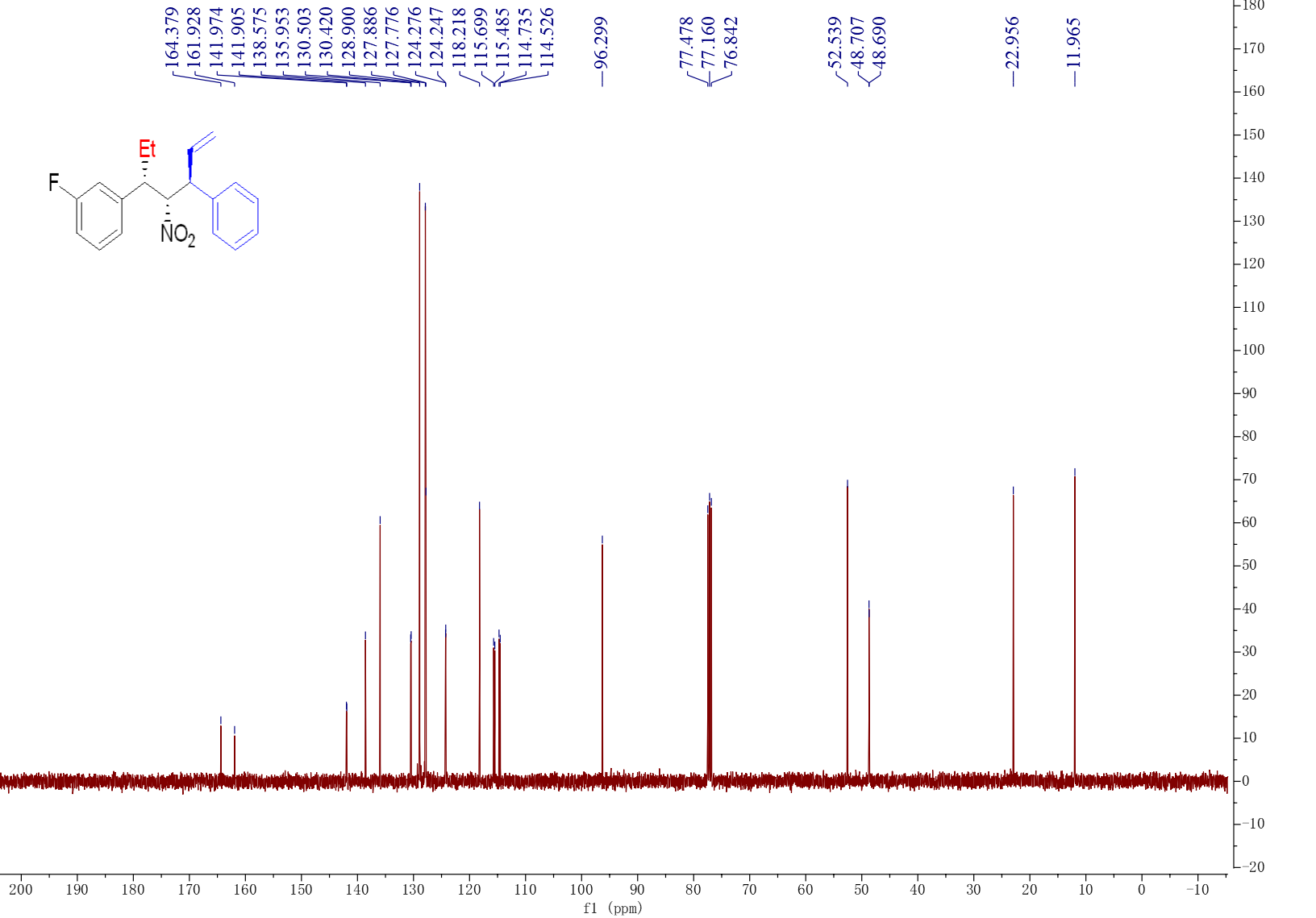


**5c** ^19^F NMR


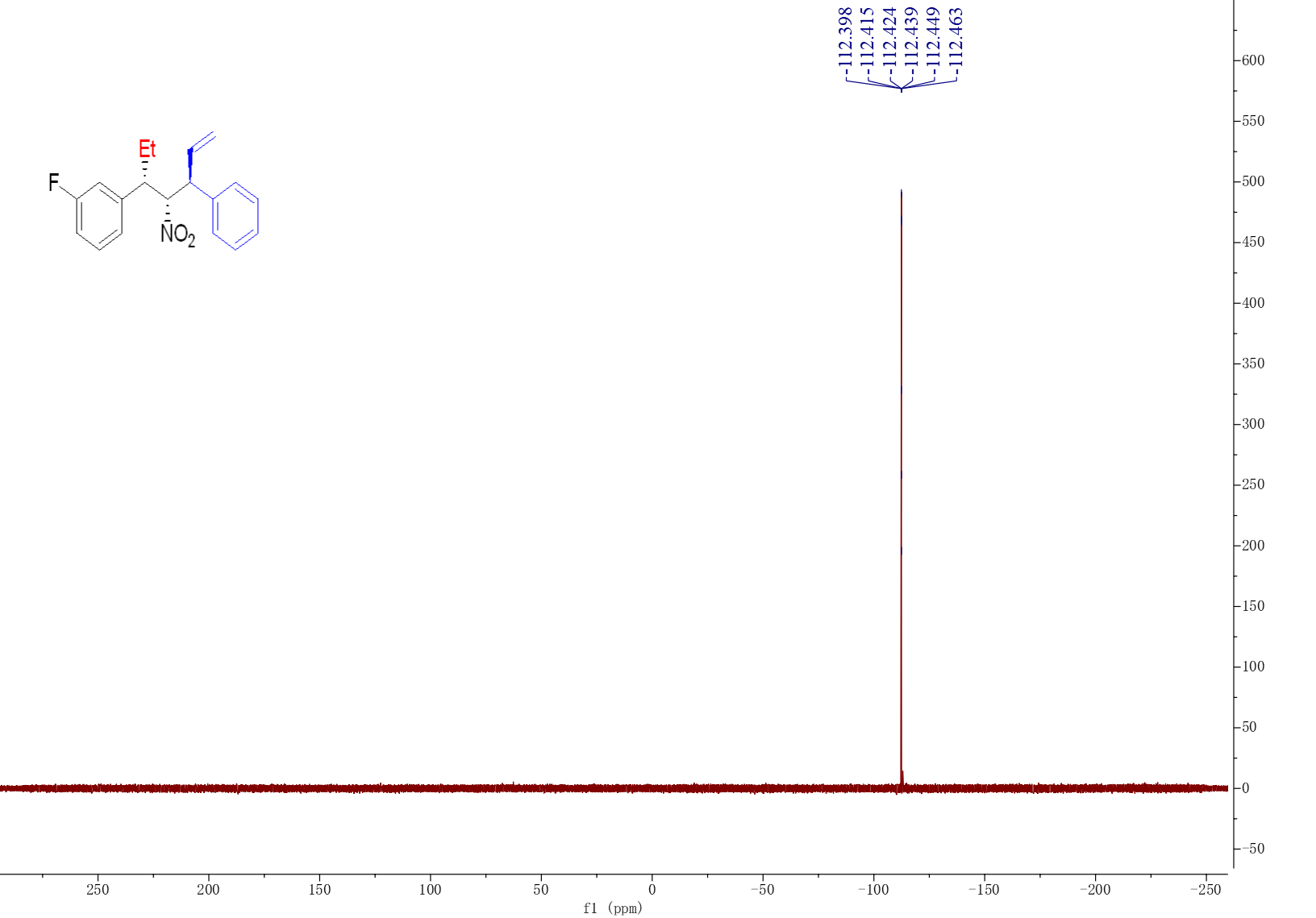


**5d** ^1^H NMR


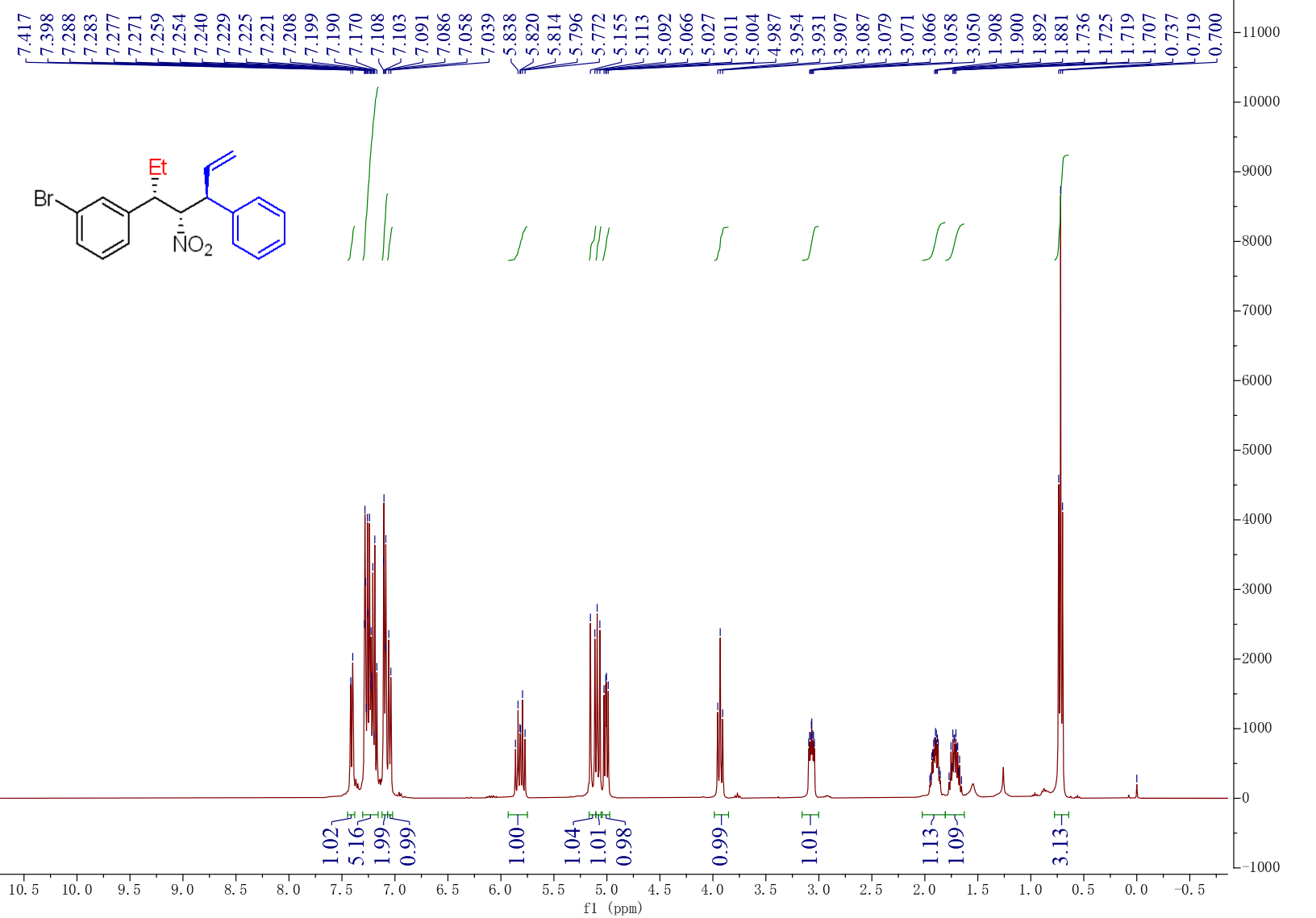


**5d** ^13^C NMR


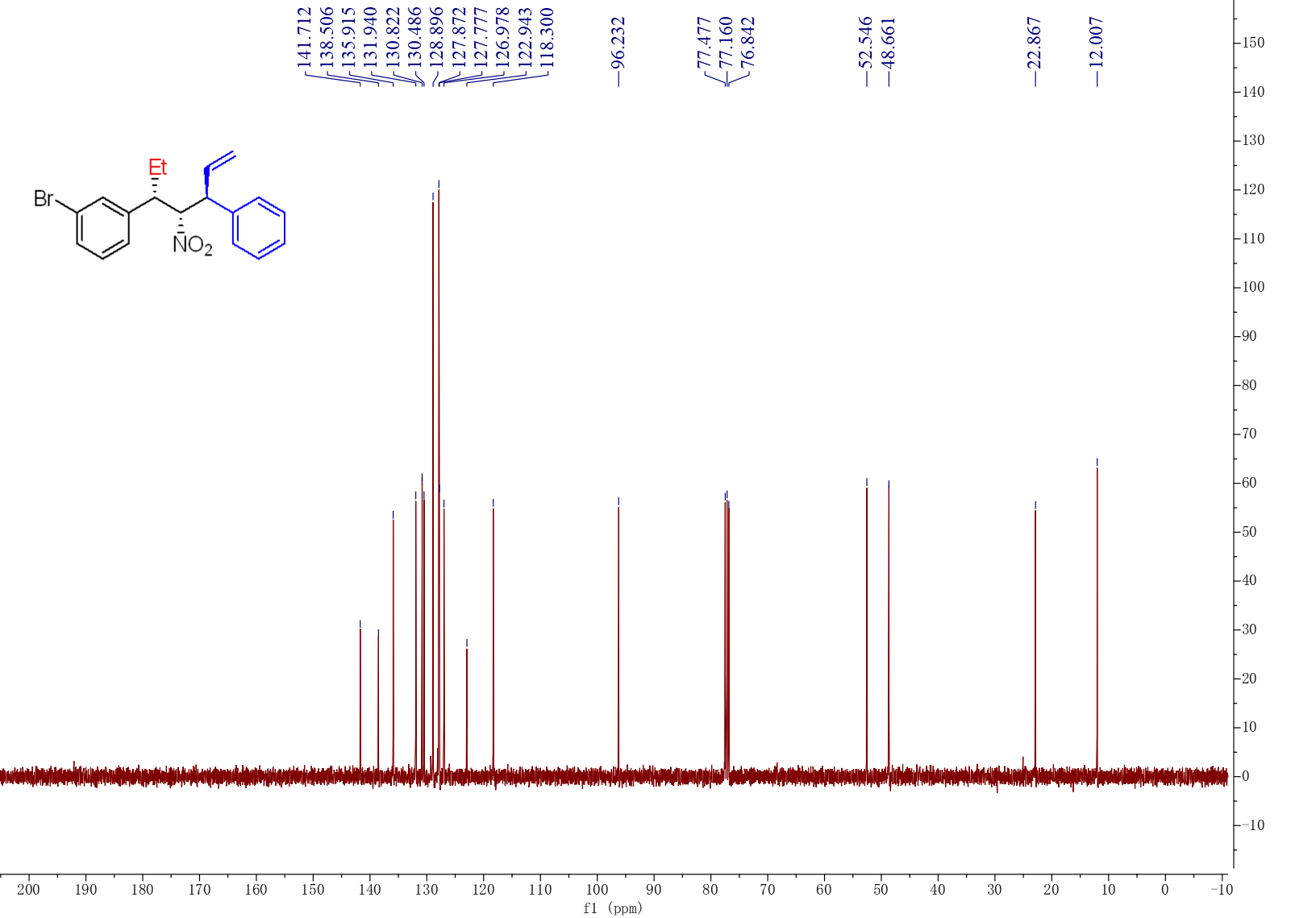


**5e** ^1^H NMR


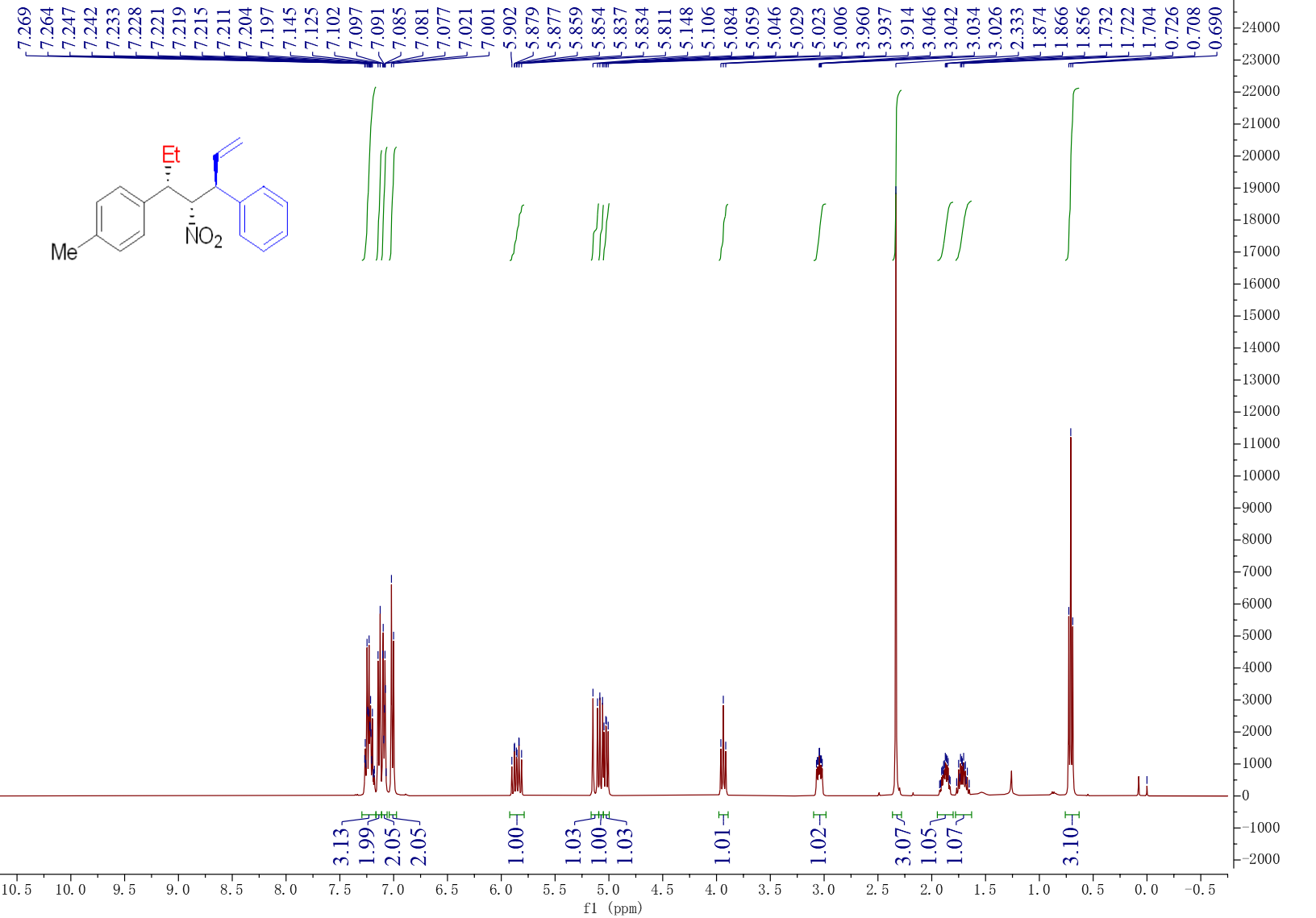


**5e** ^13^C NMR


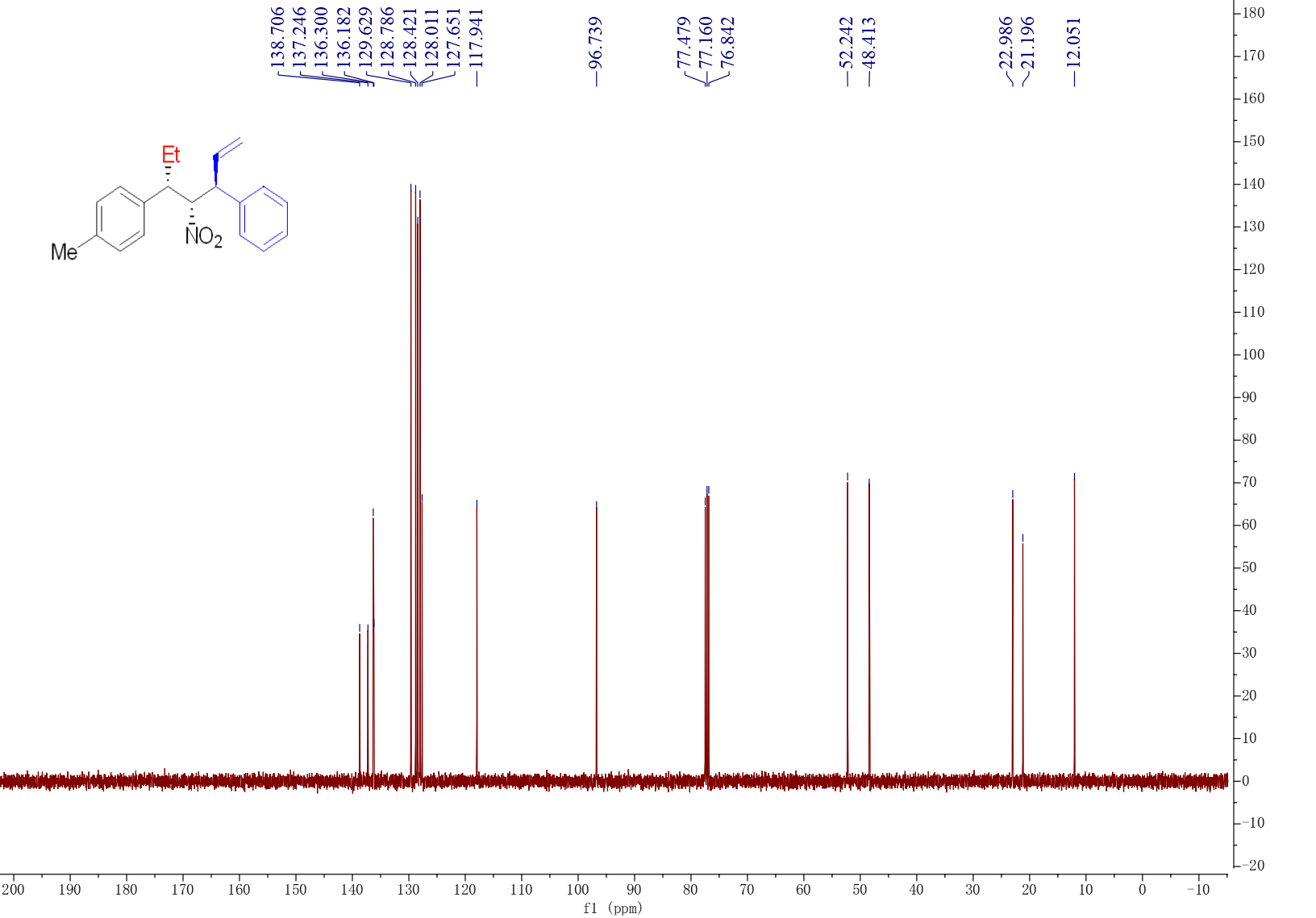


**5f** ^1^H NMR


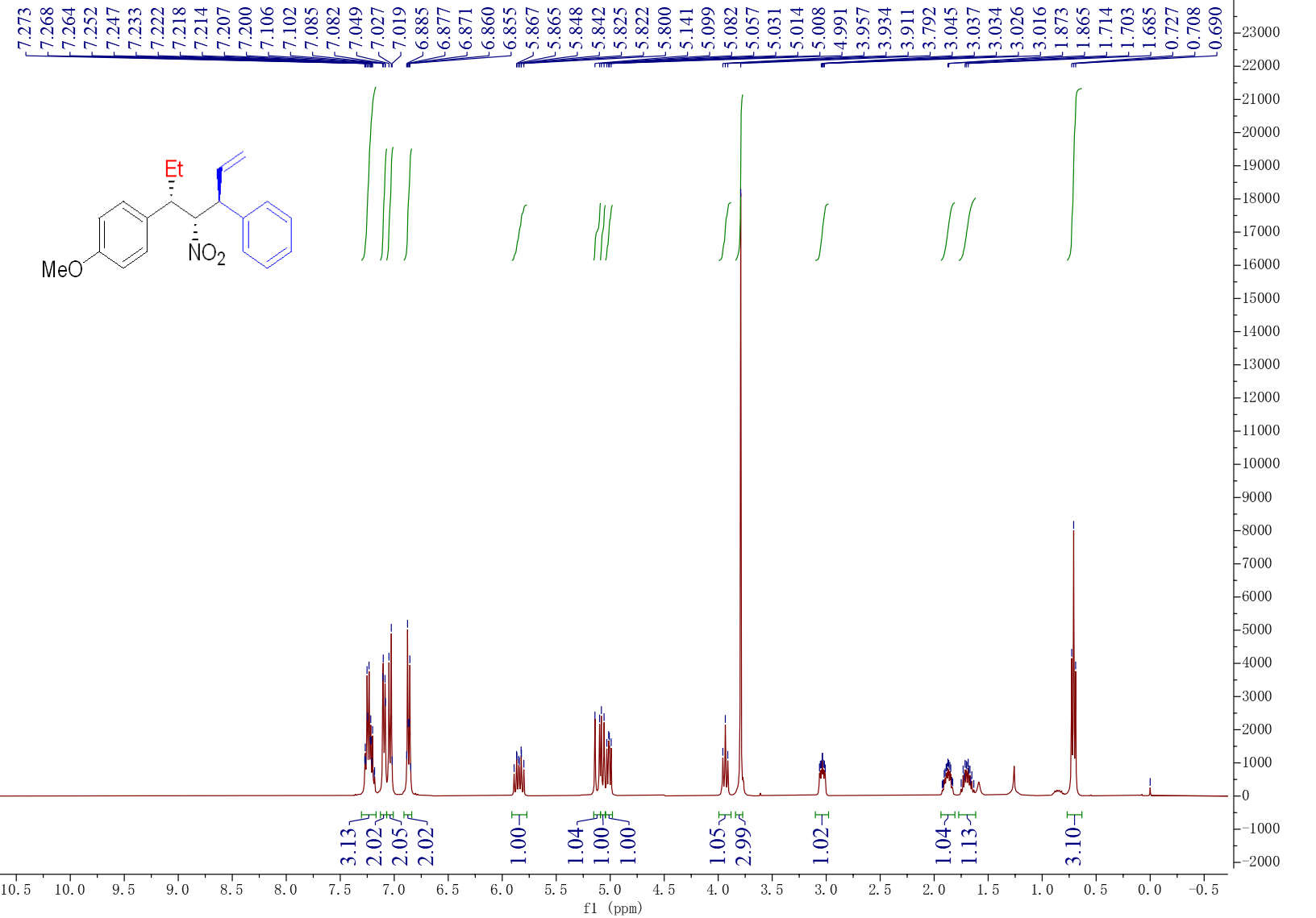


**5f** ^13^C NMR


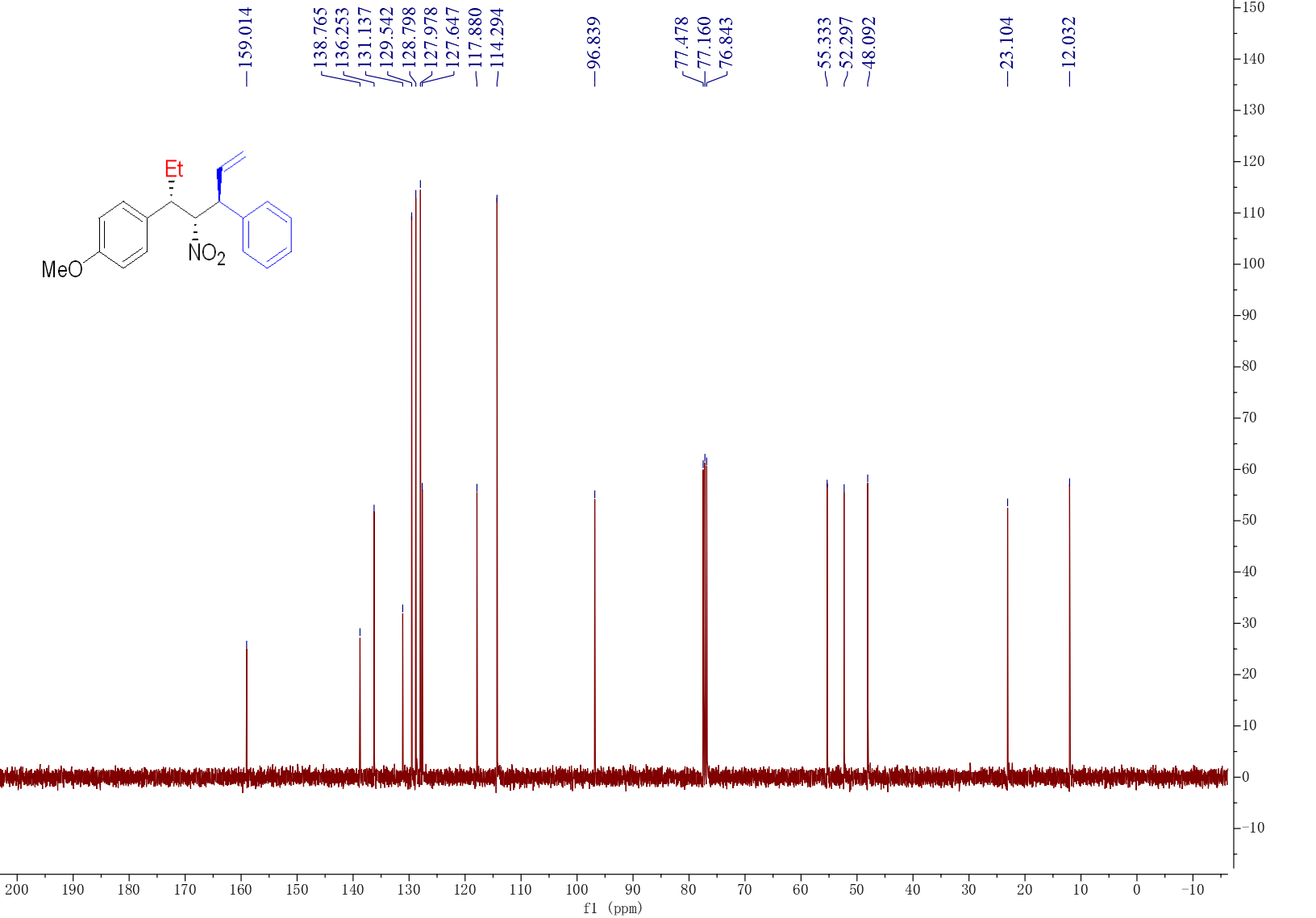


**5g** ^1^H NMR


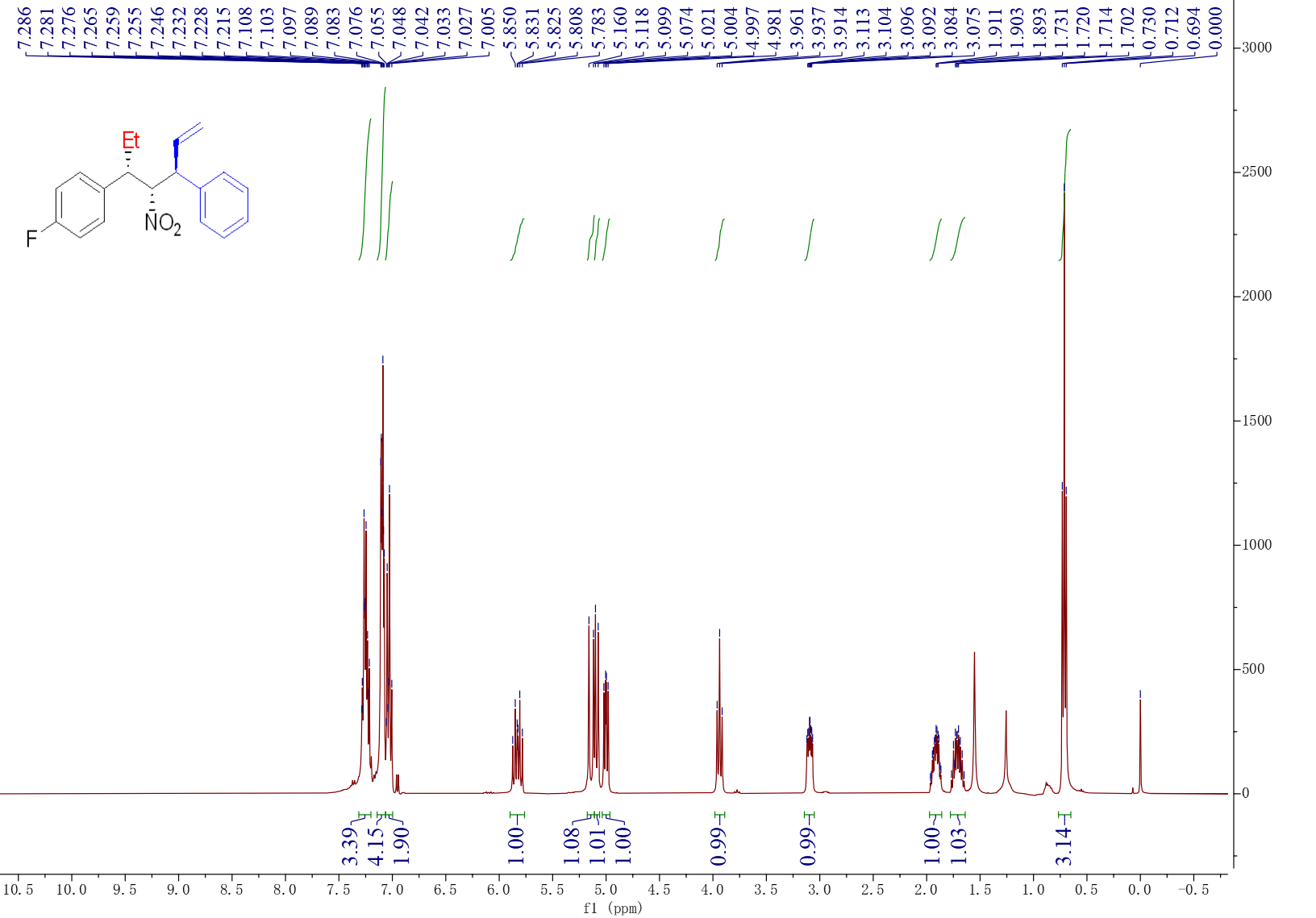


**5g** ^13^C NMR


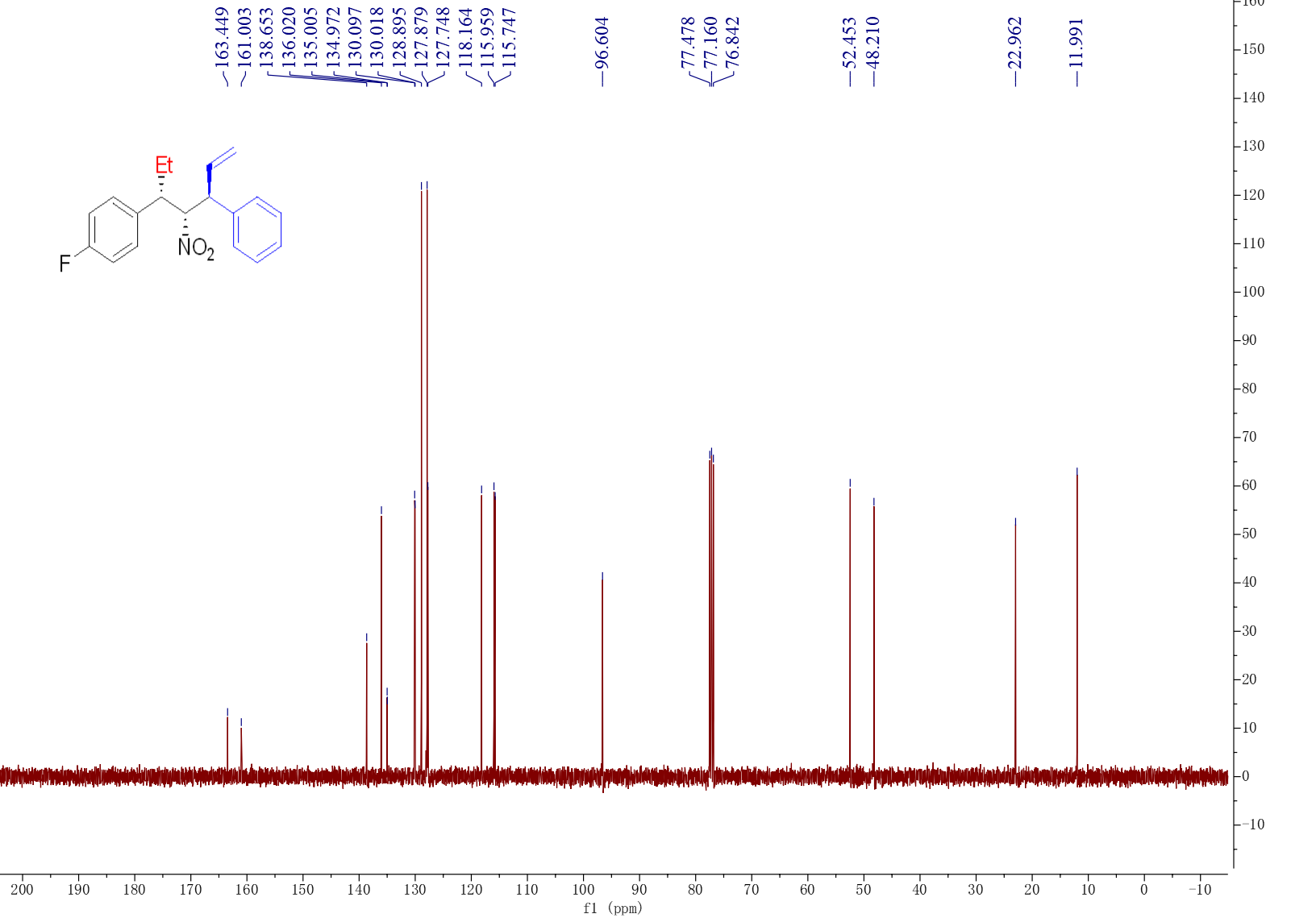


**5g** ^19^F NMR


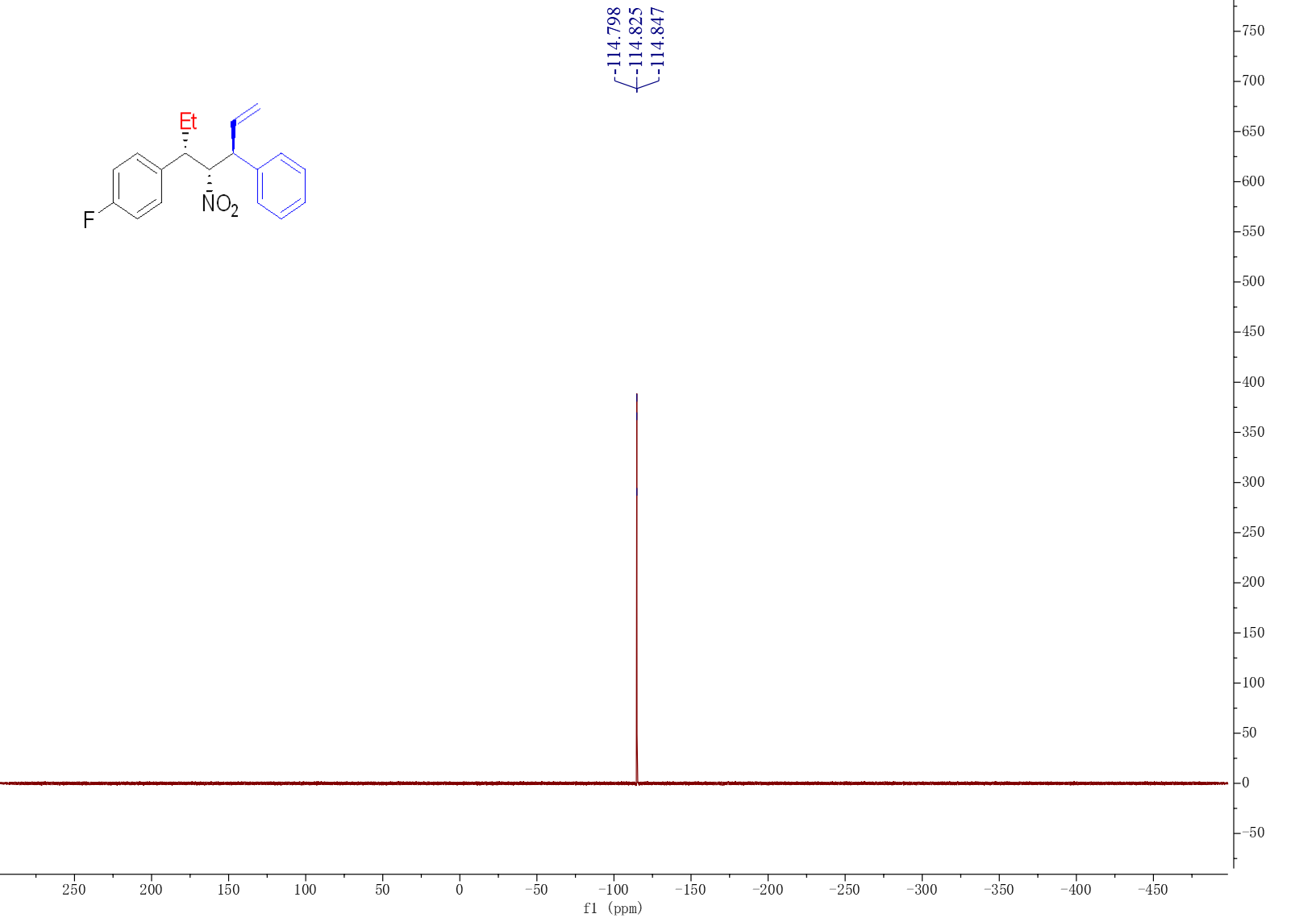


**5h** ^1^H NMR


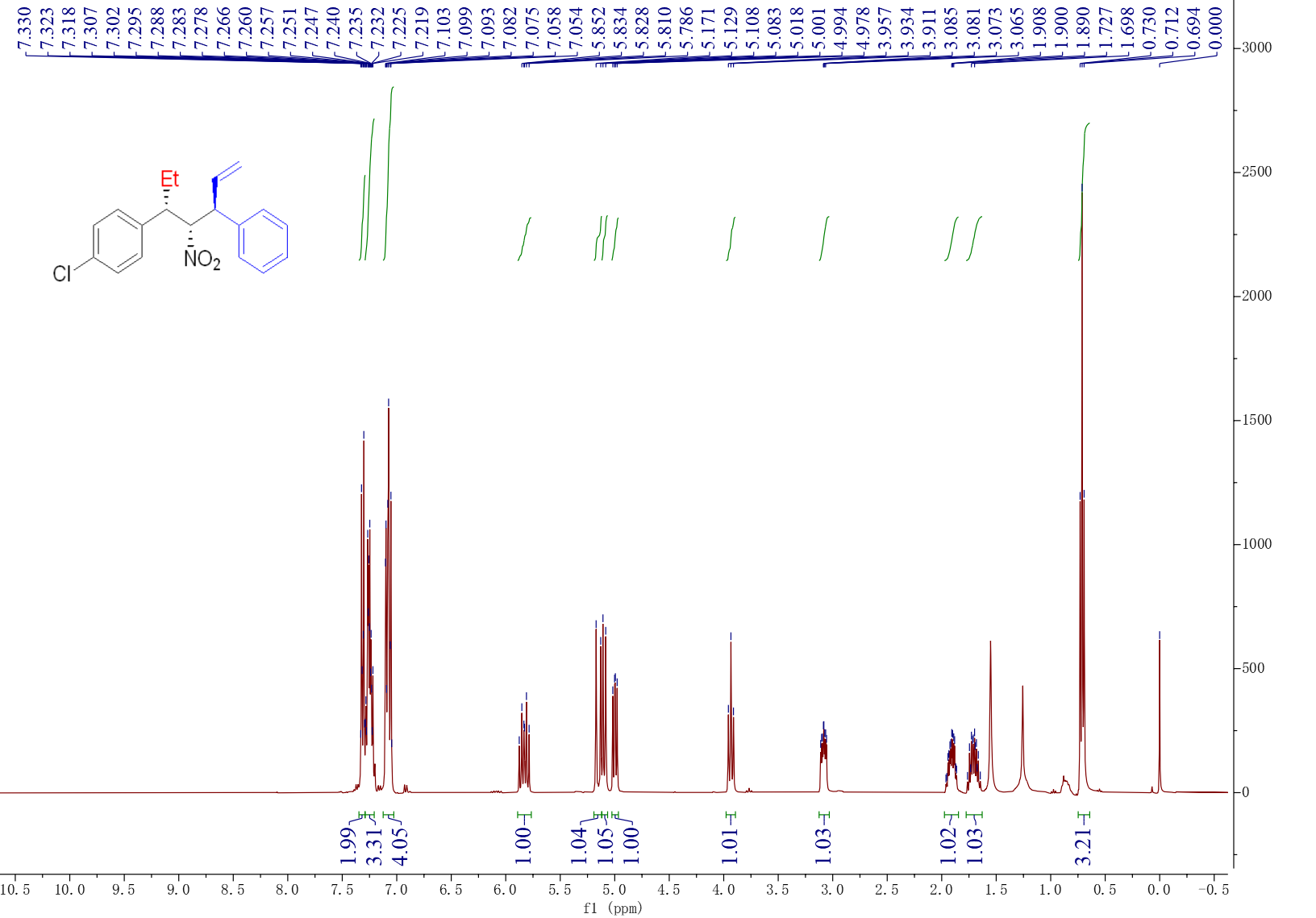


**5h** ^13^C NMR


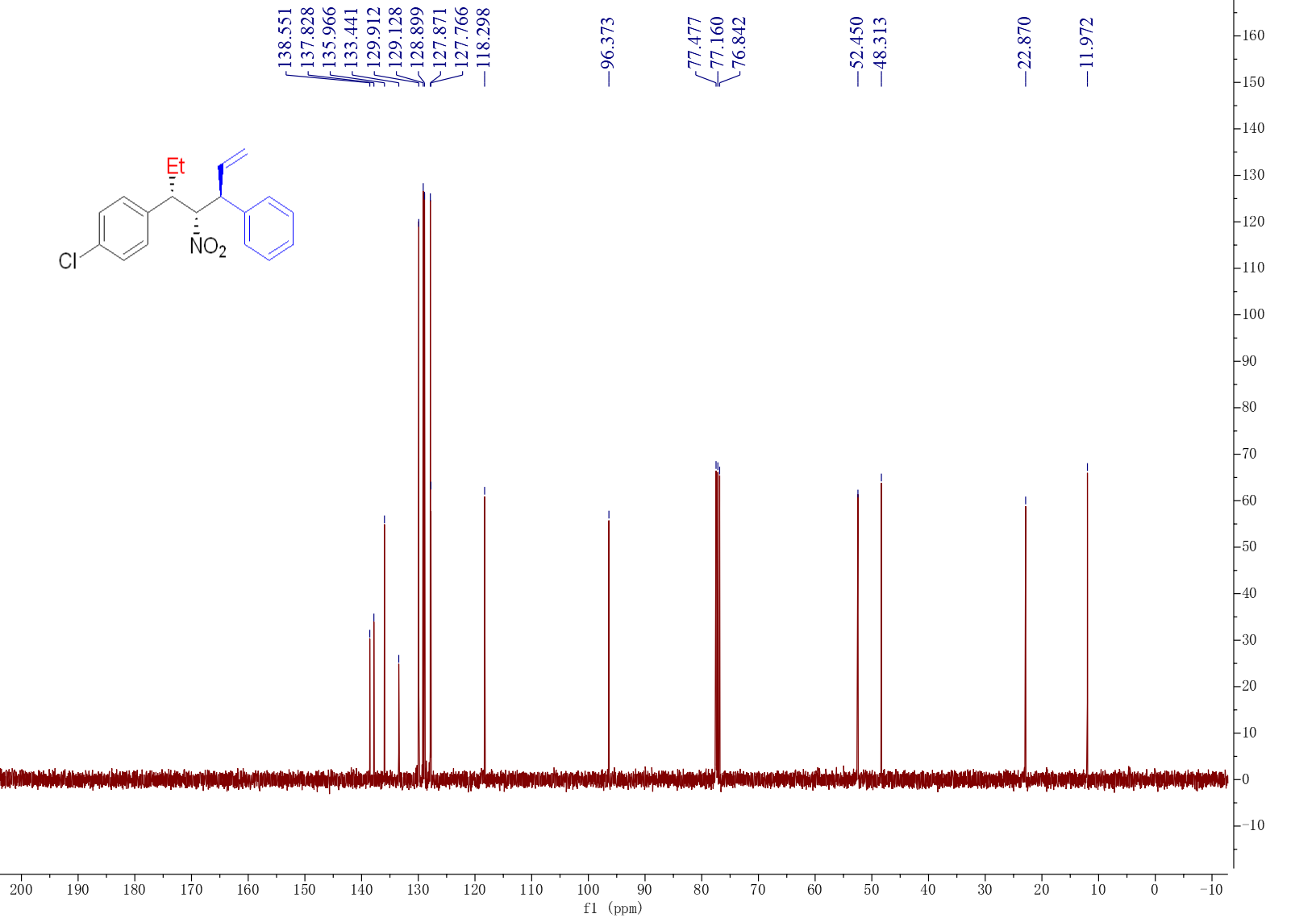


**5i** ^1^H NMR


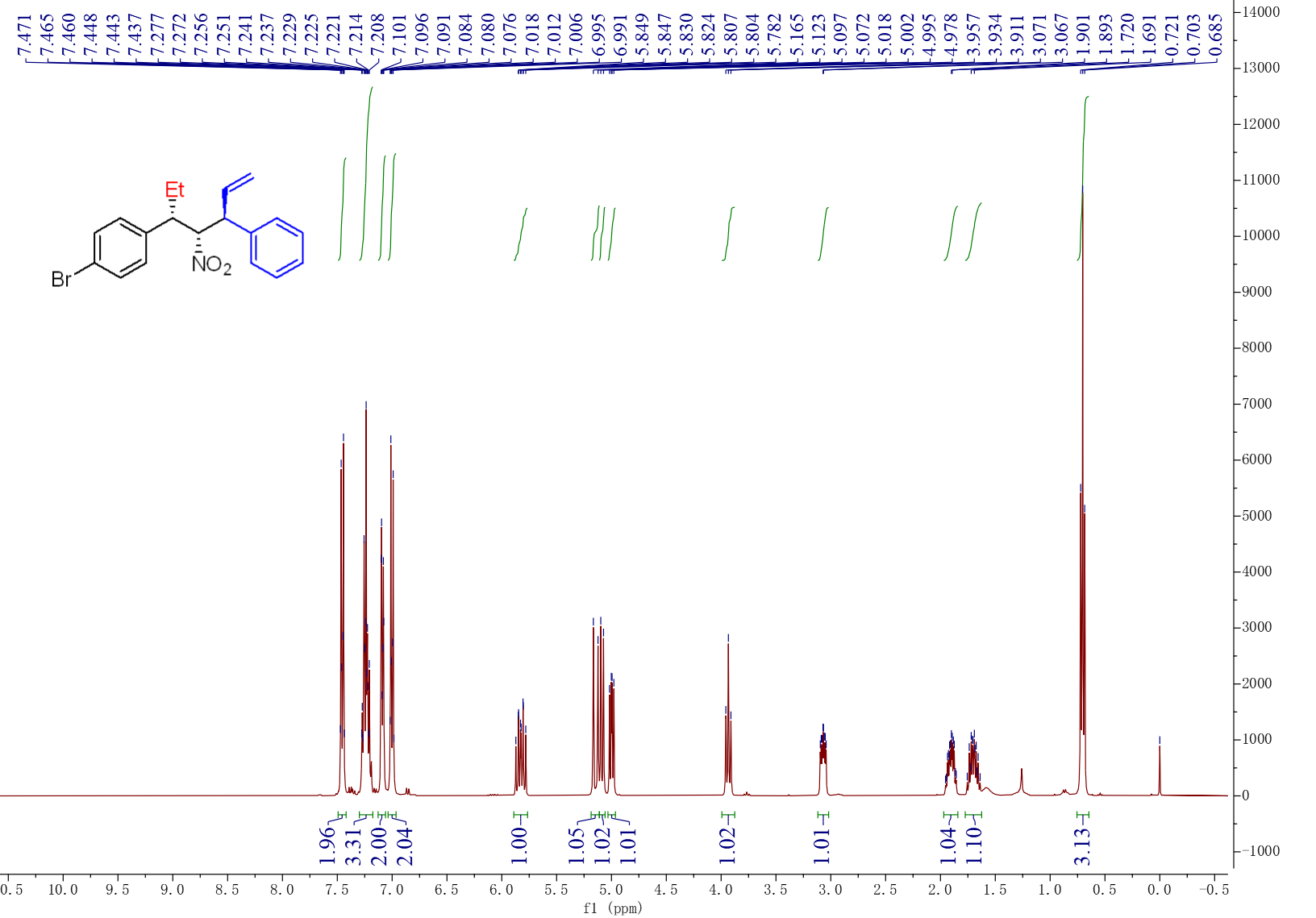


**5i** ^13^C NMR


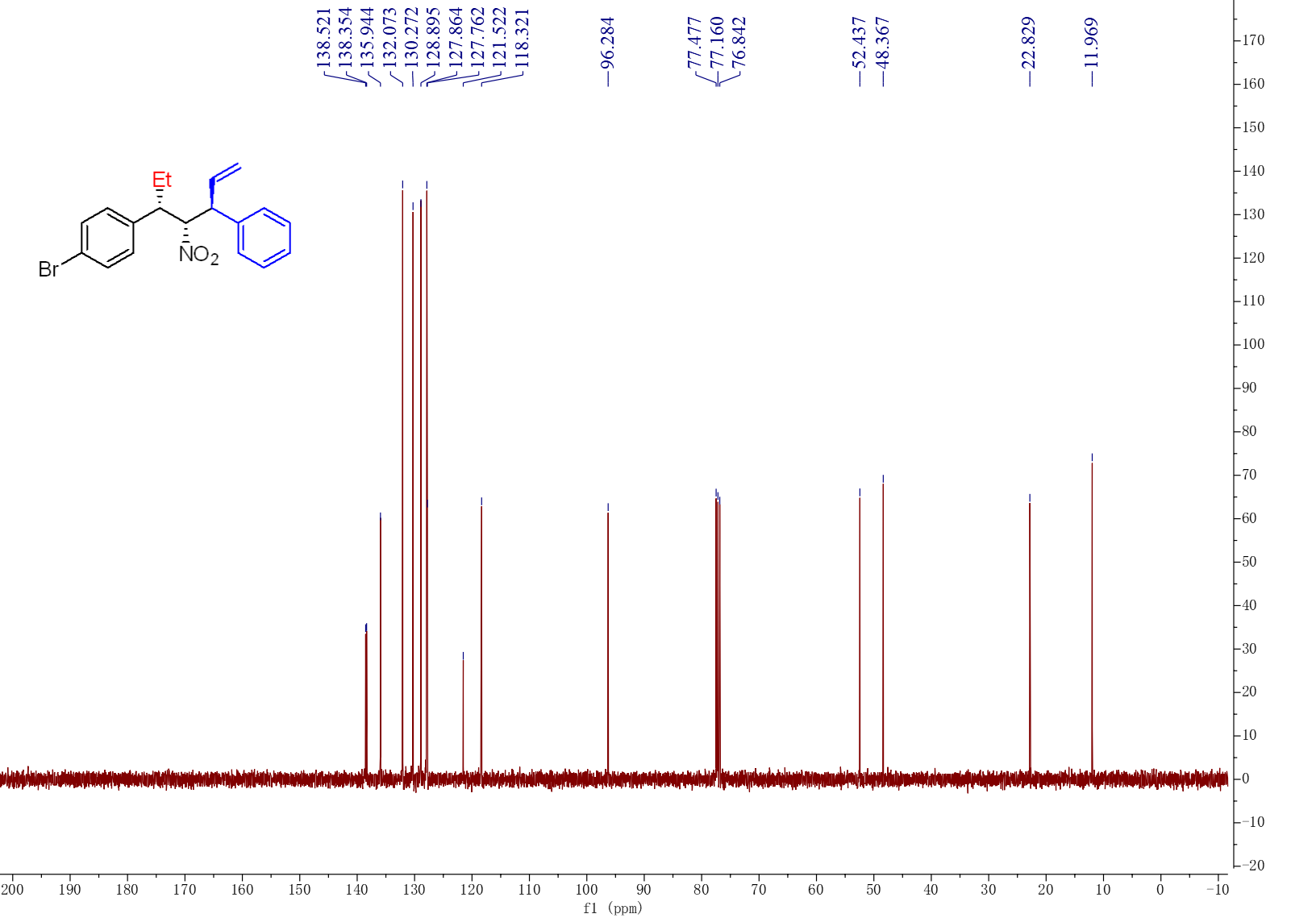


**5j** ^1^H NMR


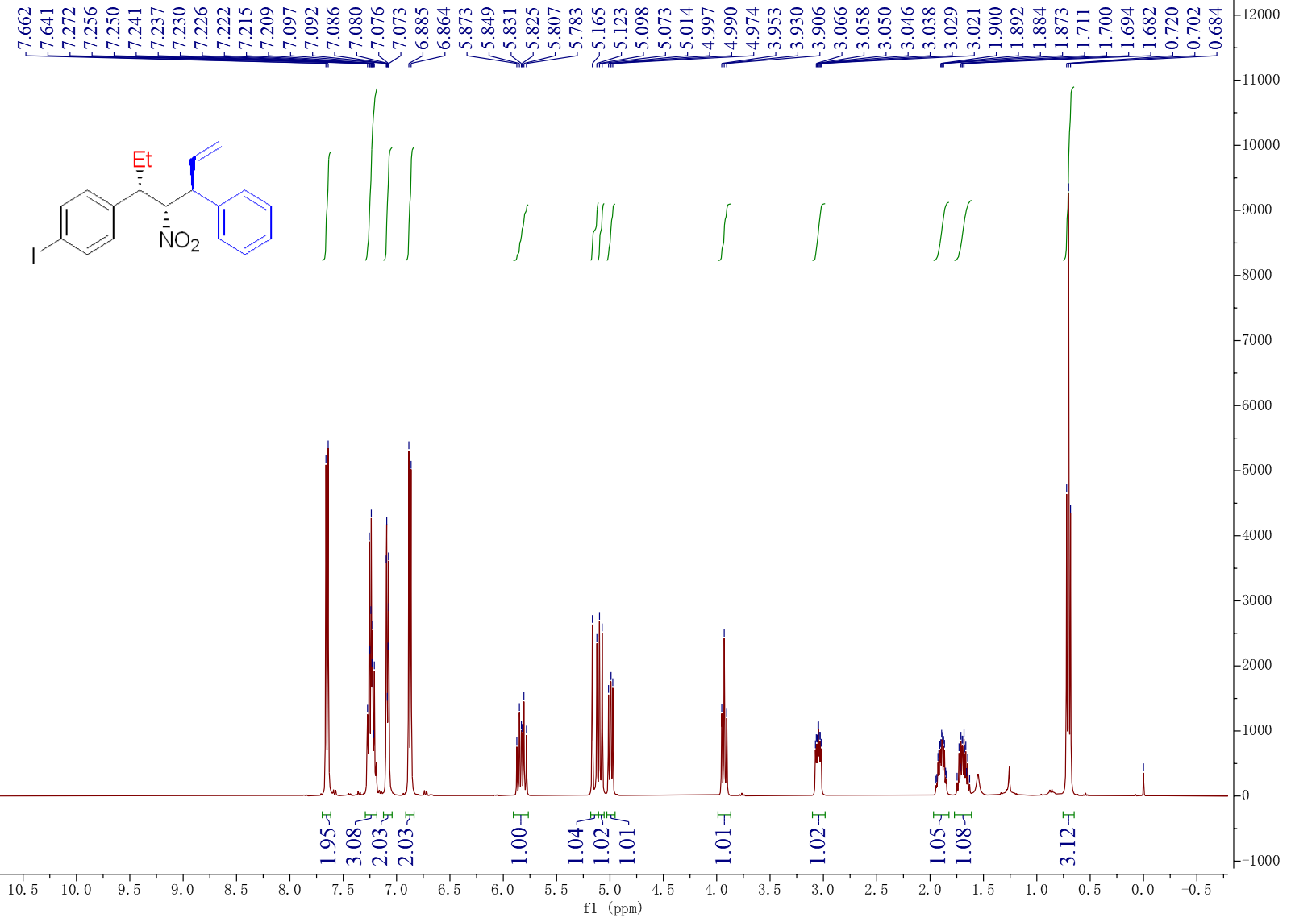


**5j** ^13^C NMR


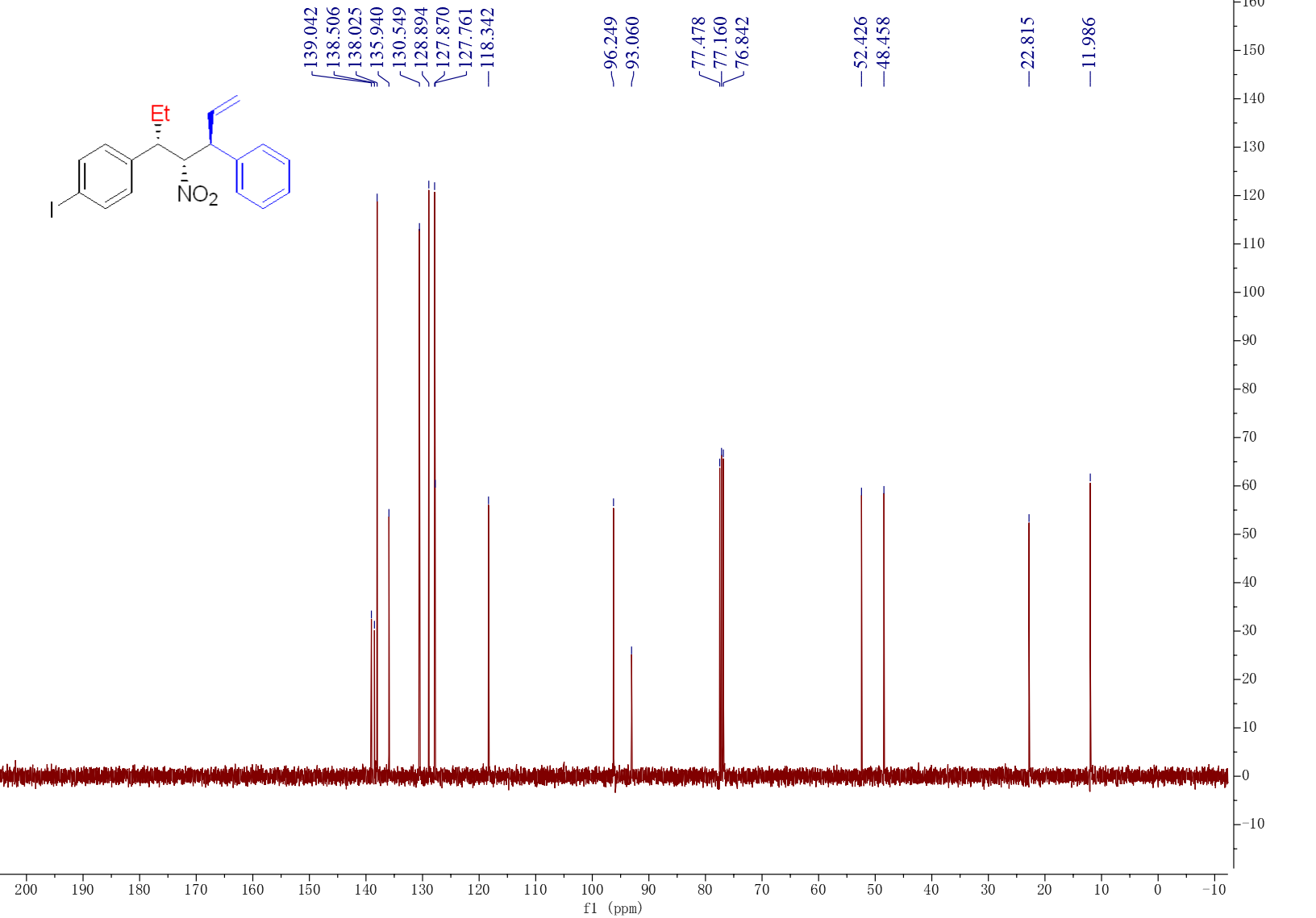


**5k** ^1^H NMR


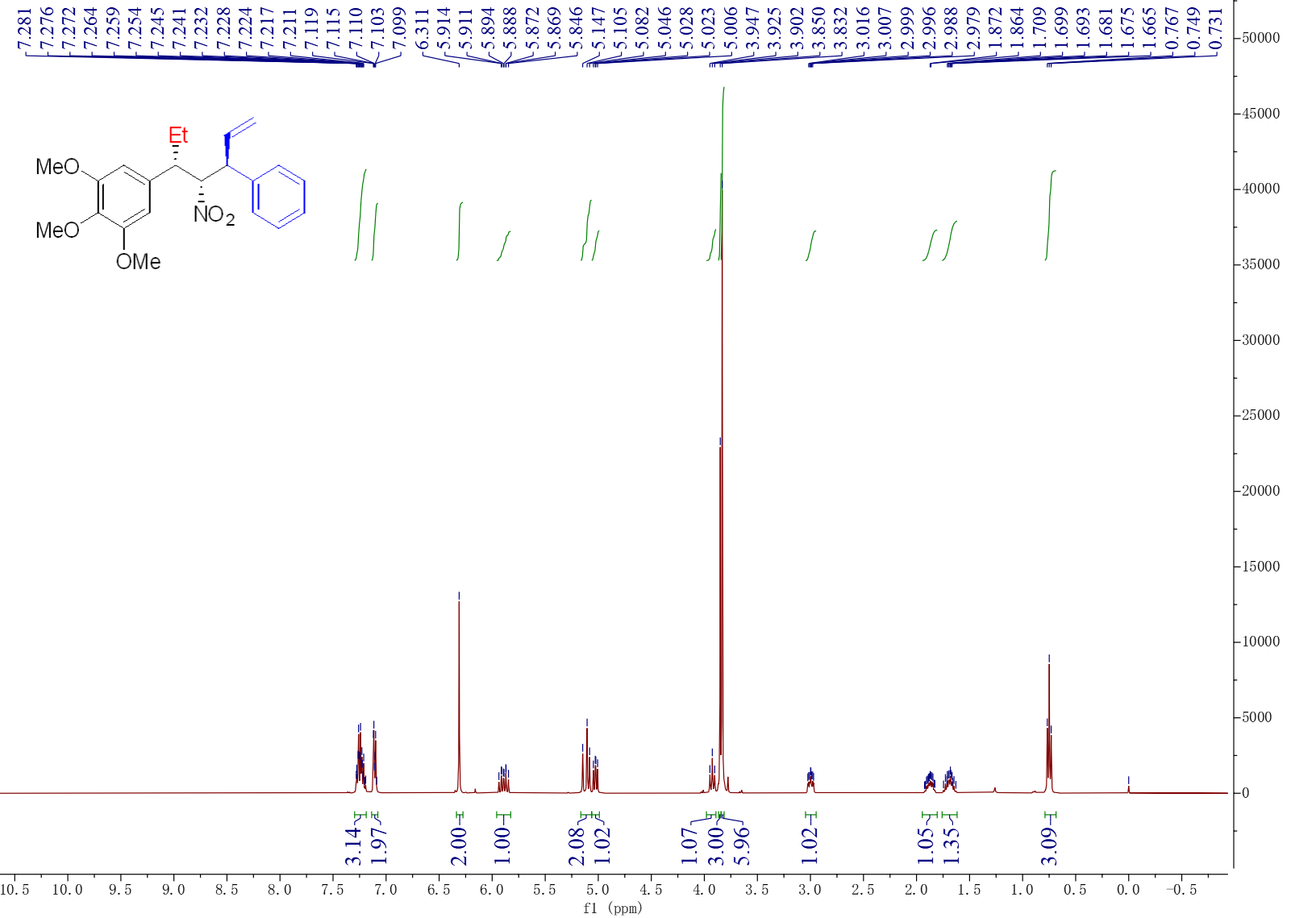


**5k** ^13^C NMR


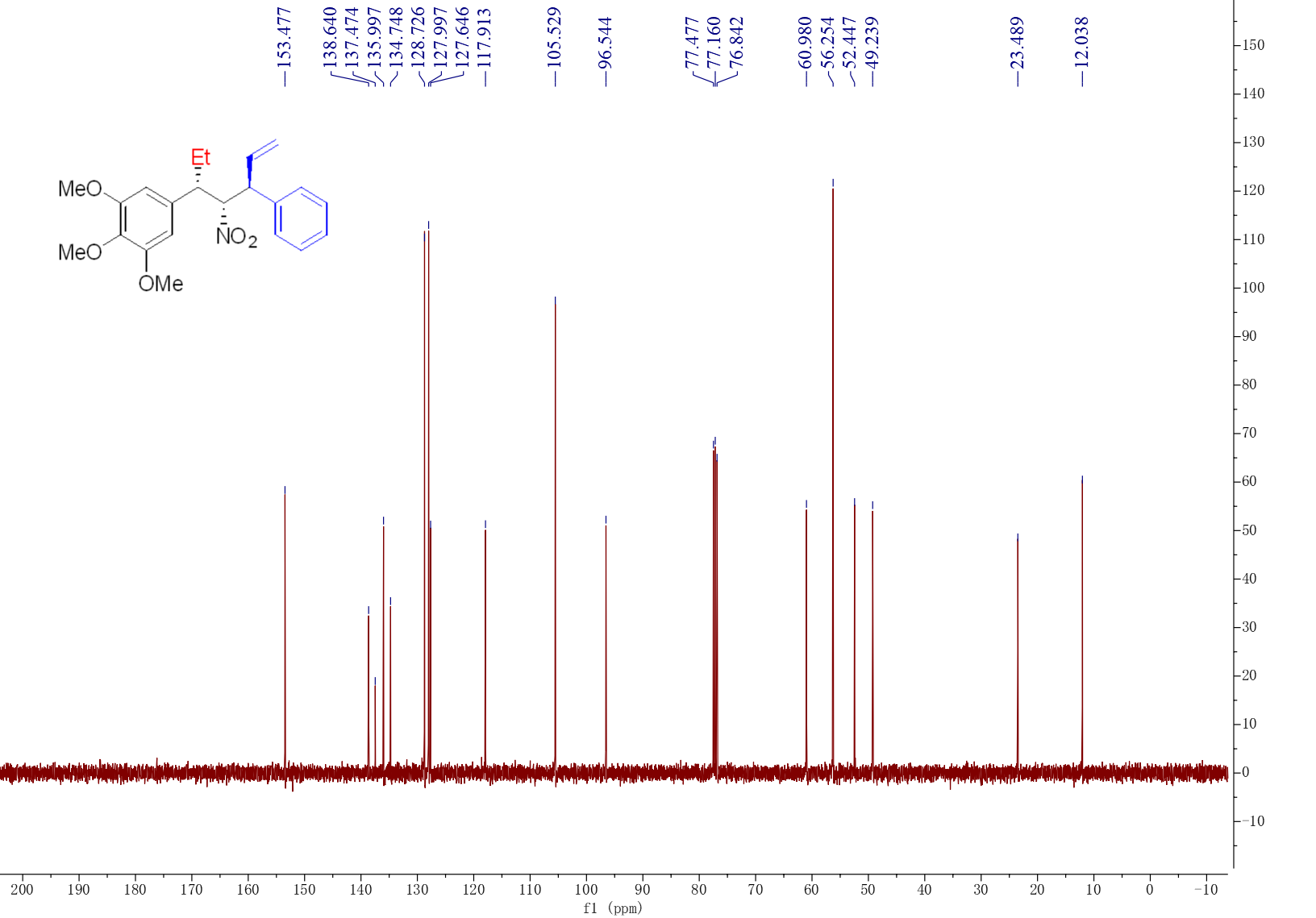


**5l** ^1^H NMR


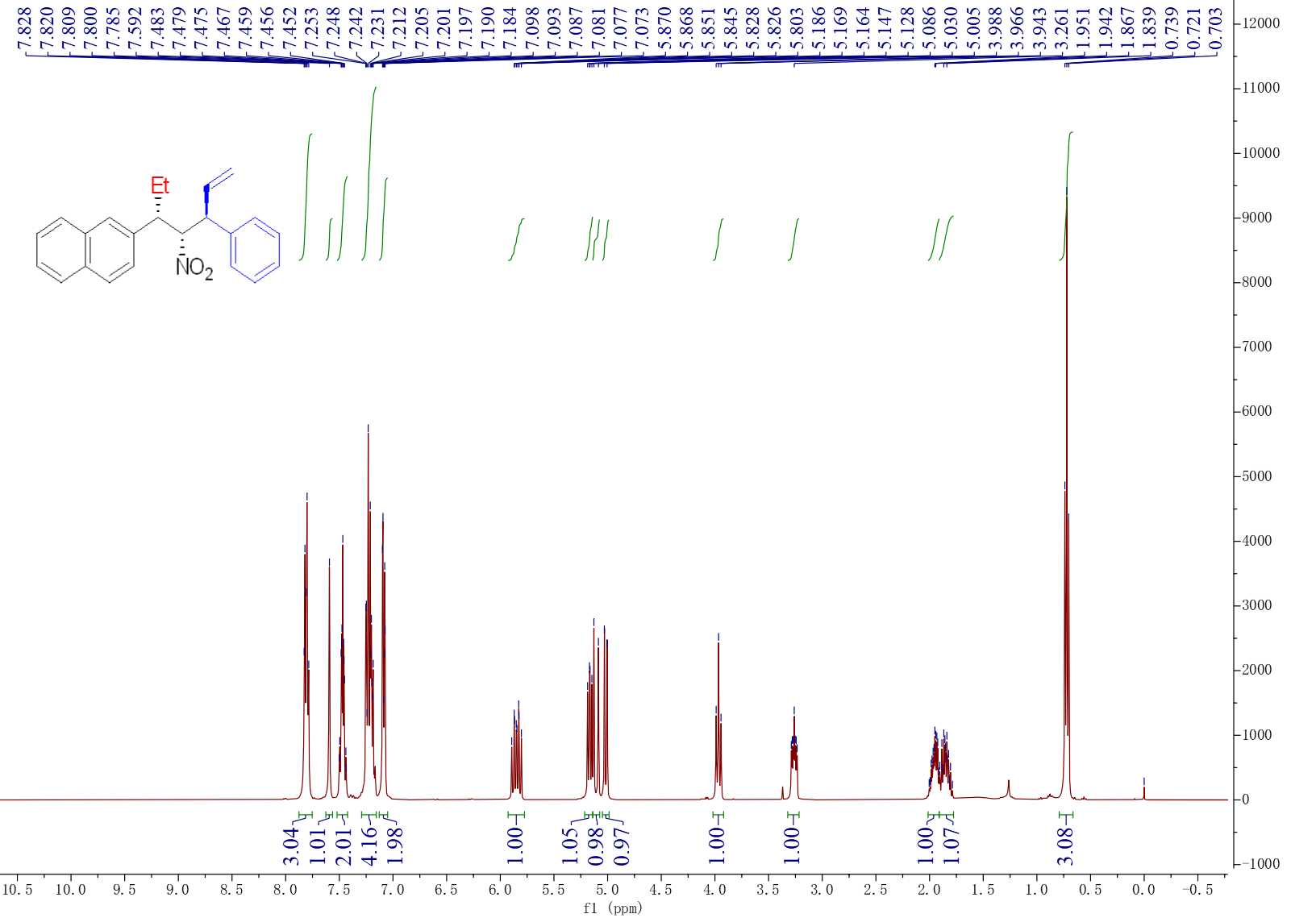


**5l** ^13^C NMR


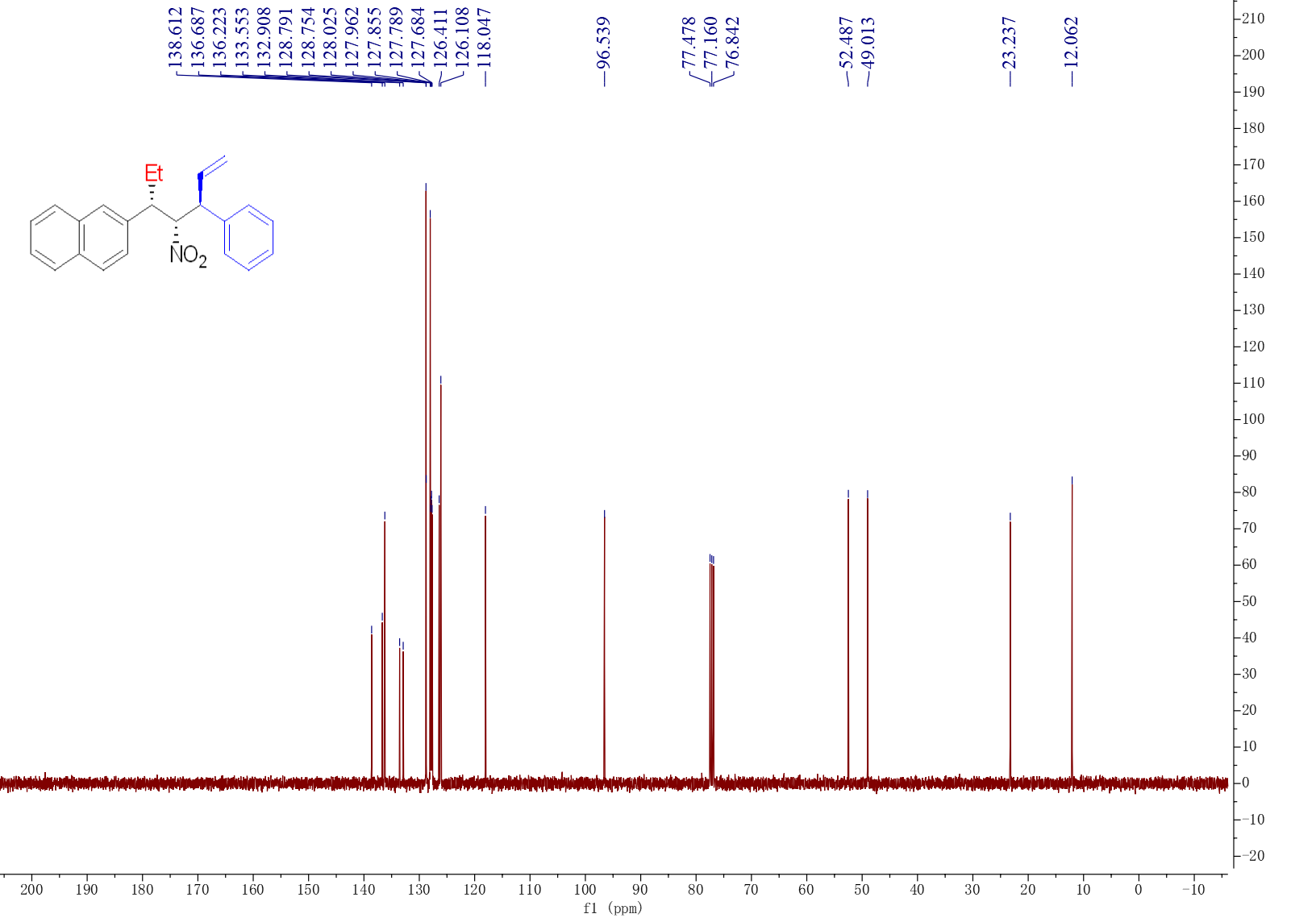


**5m** ^1^H NMR


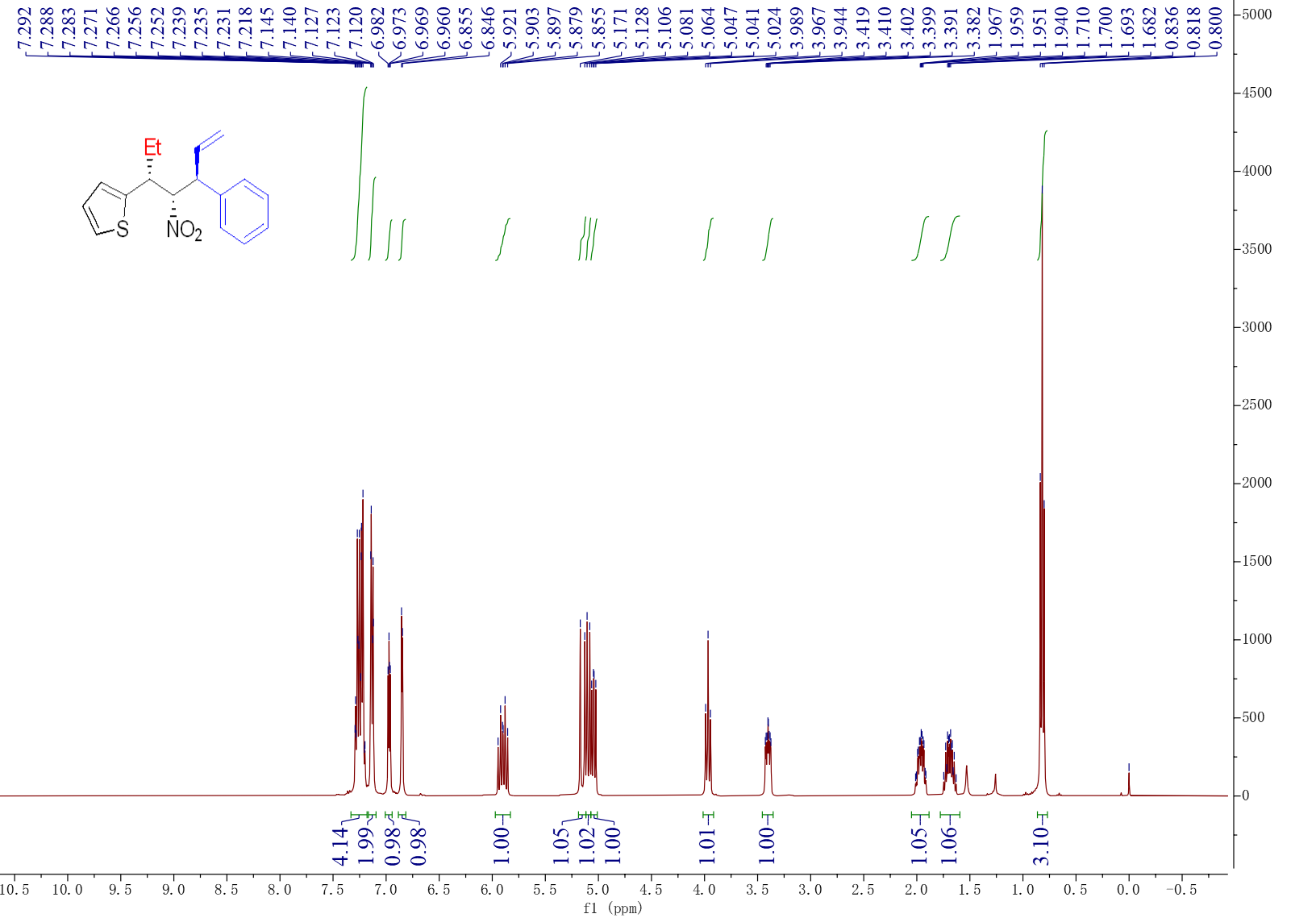


**5m** ^13^C NMR


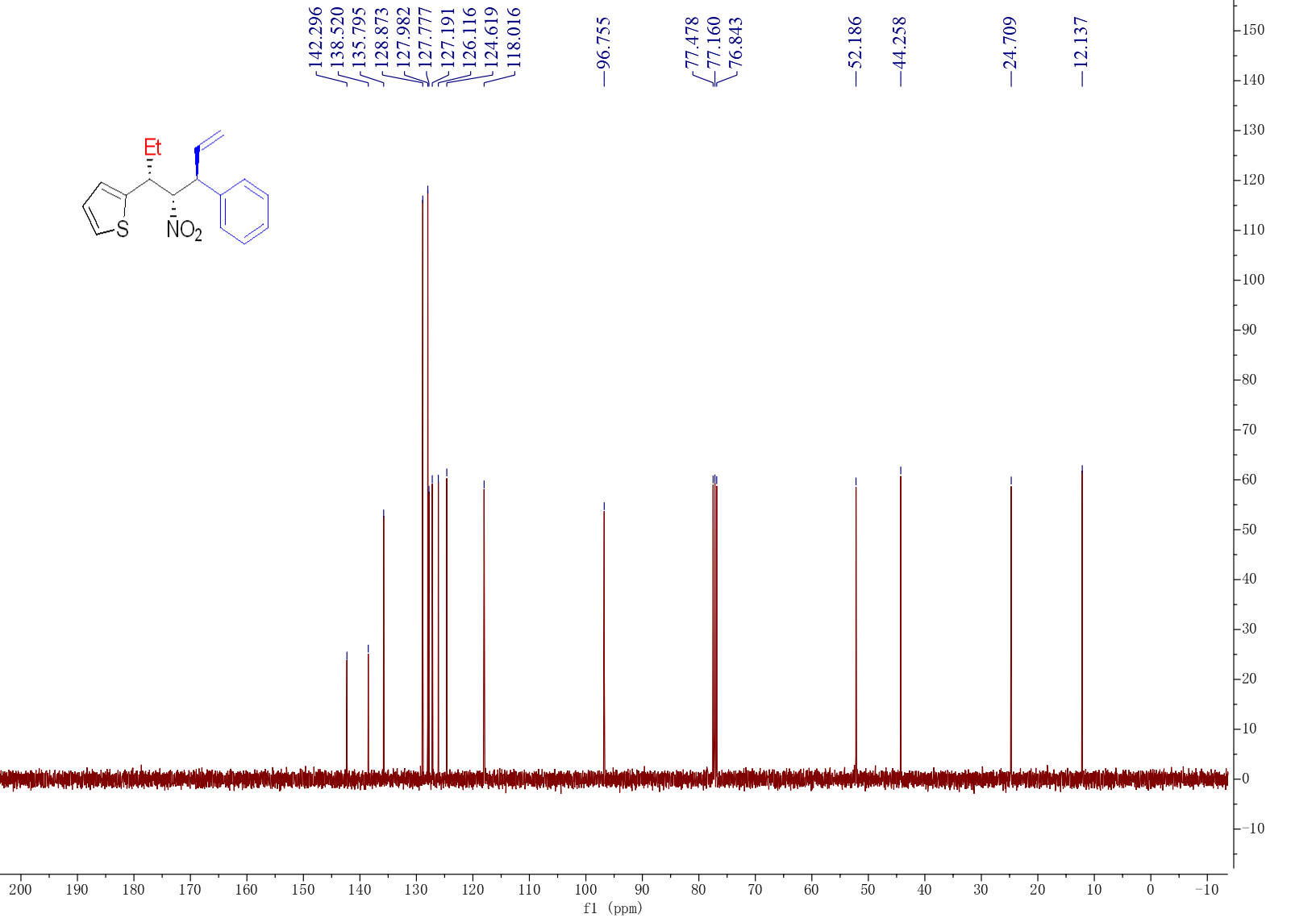


**5n** ^1^H NMR


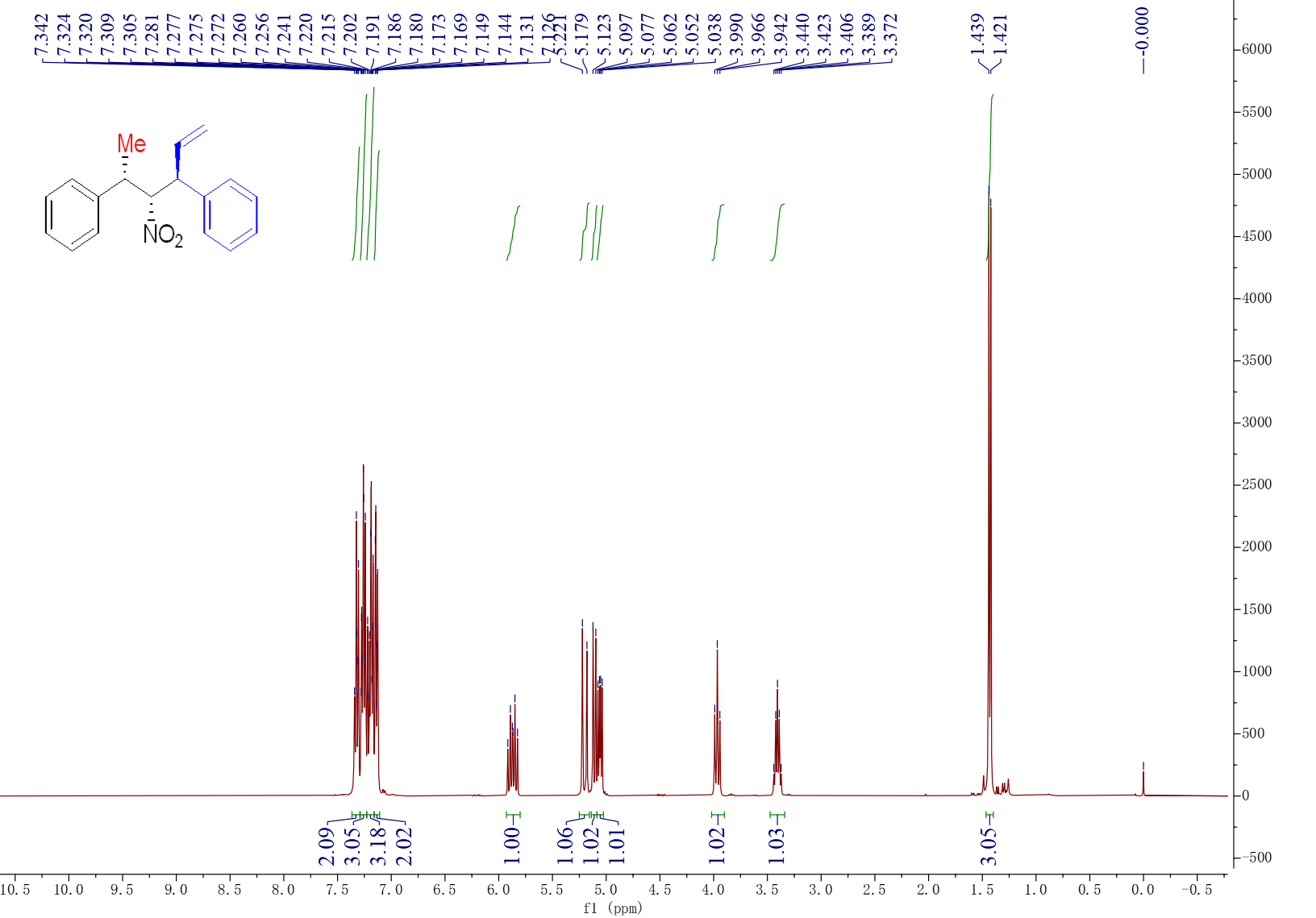


**5n** ^13^C NMR


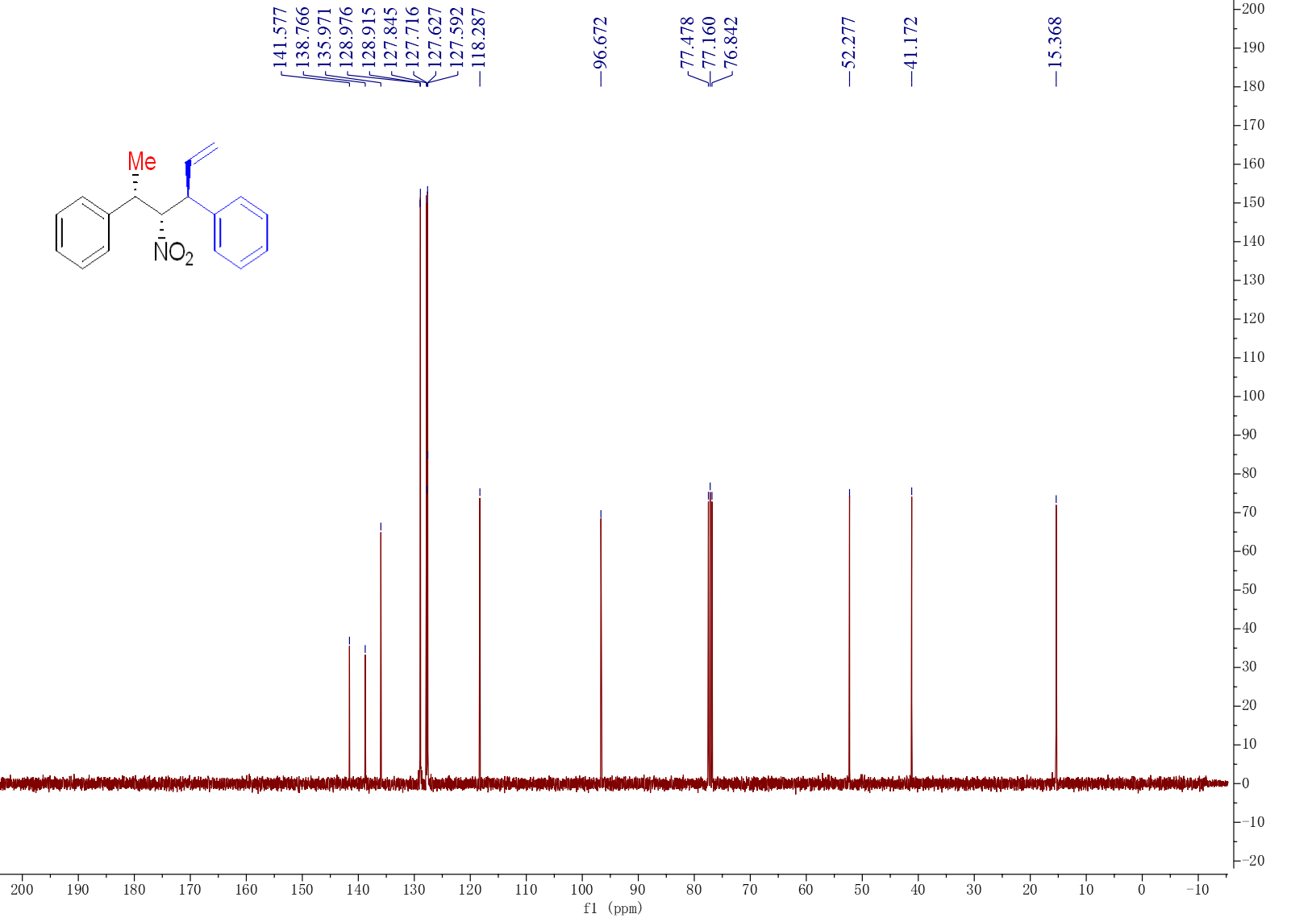


**4a′** ^1^H NMR


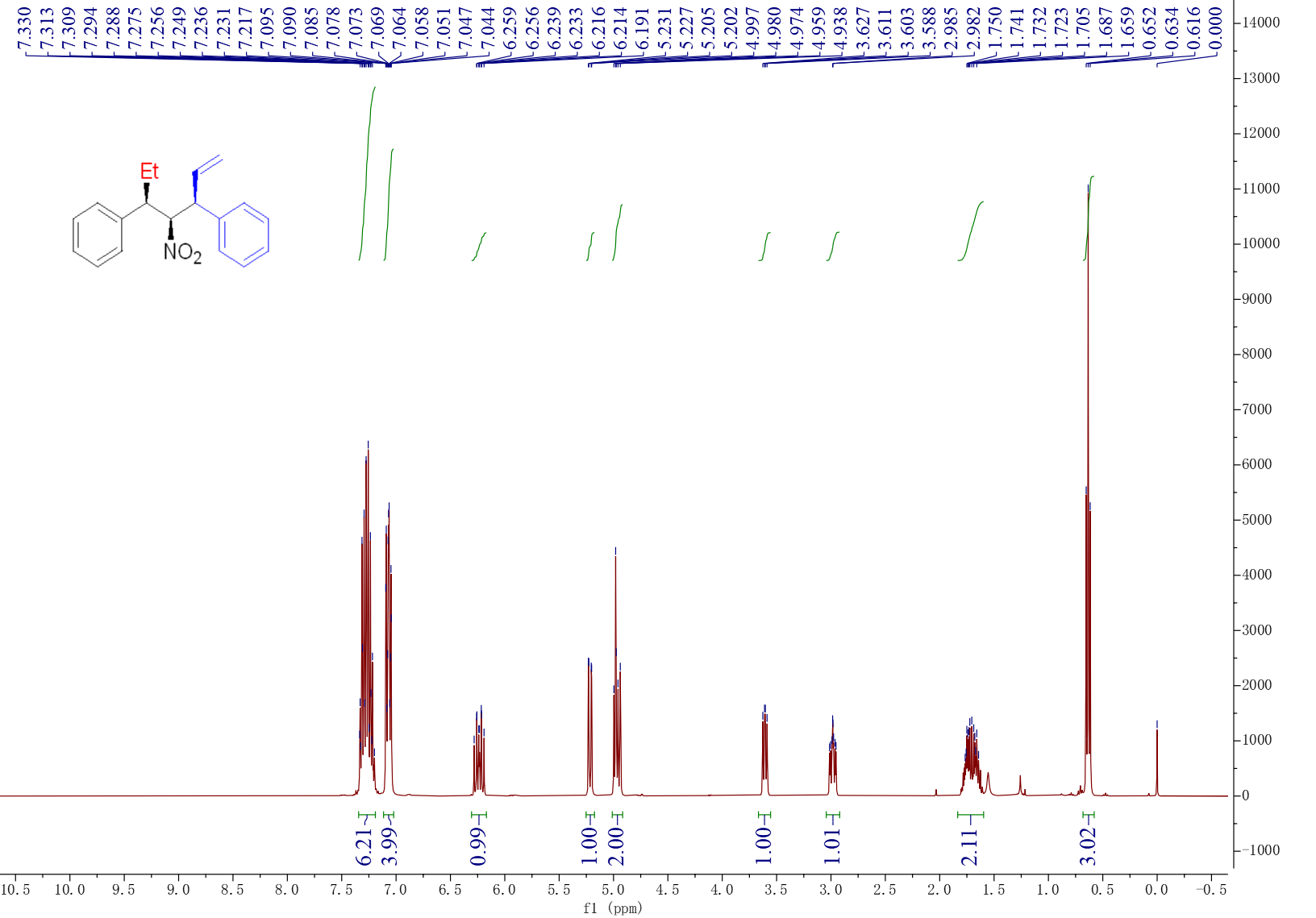


**4a′** ^13^C NMR


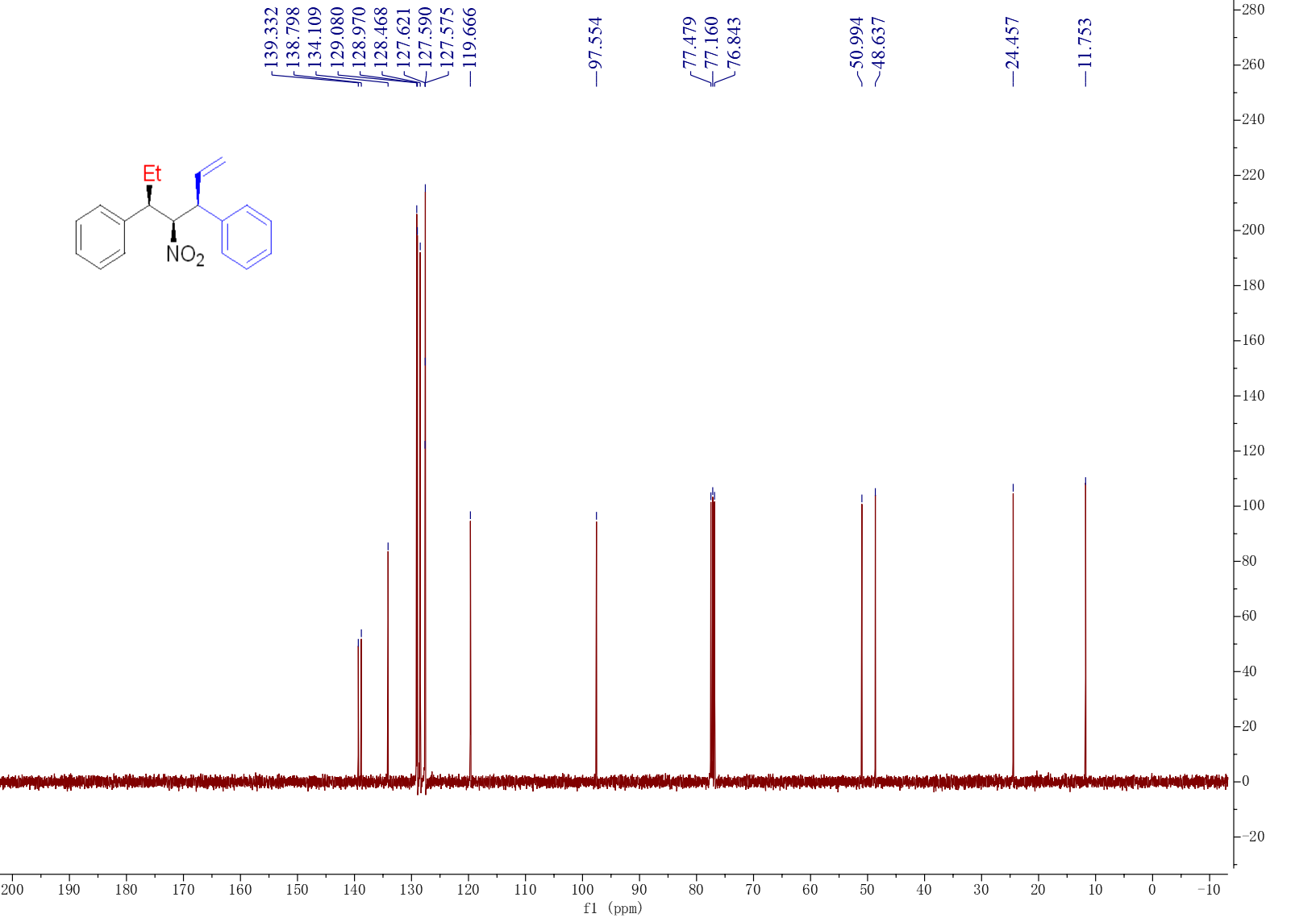


**4a′′** ^1^H NMR


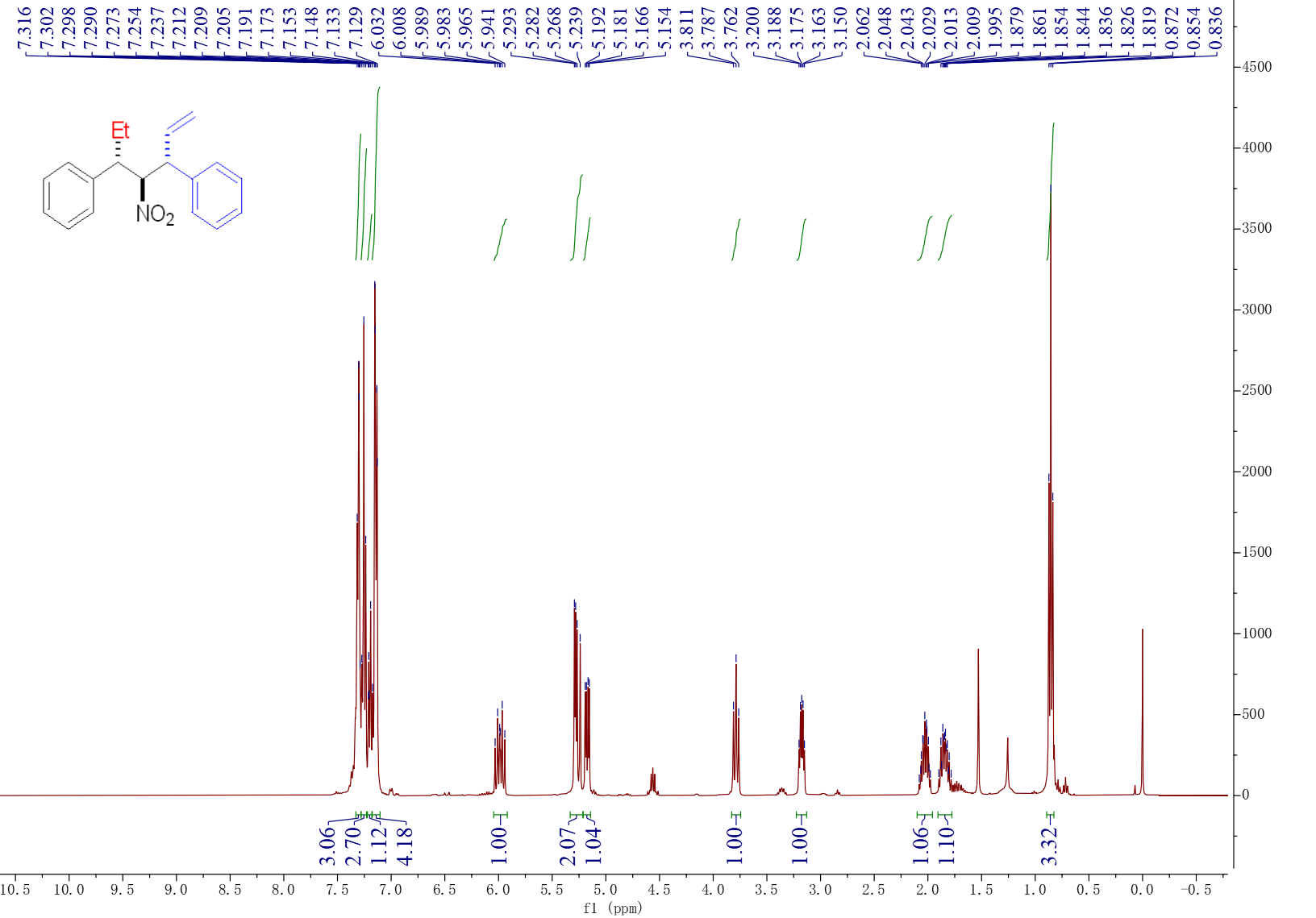


**4a′′** ^13^C NMR


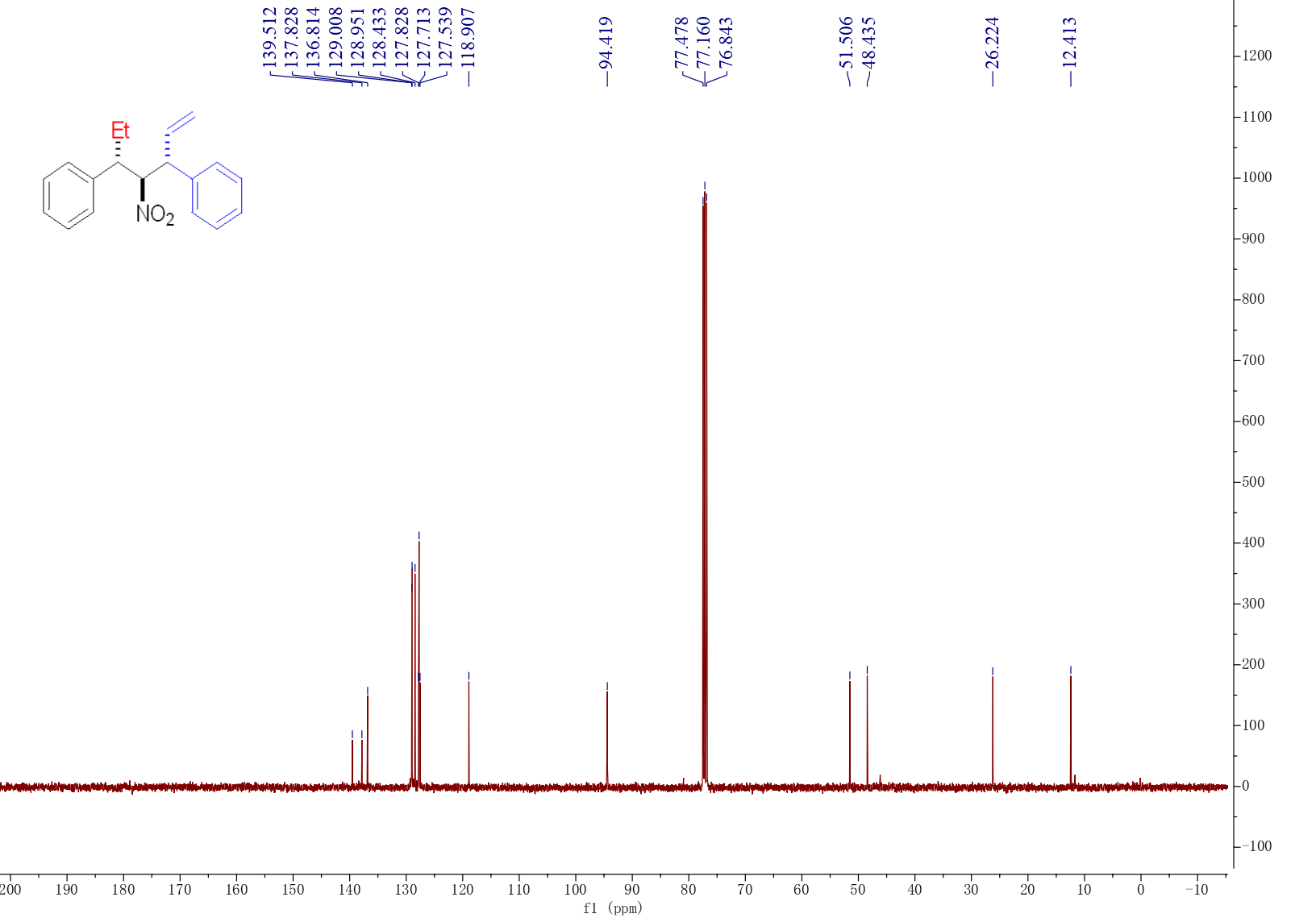


**6** ^1^H NMR


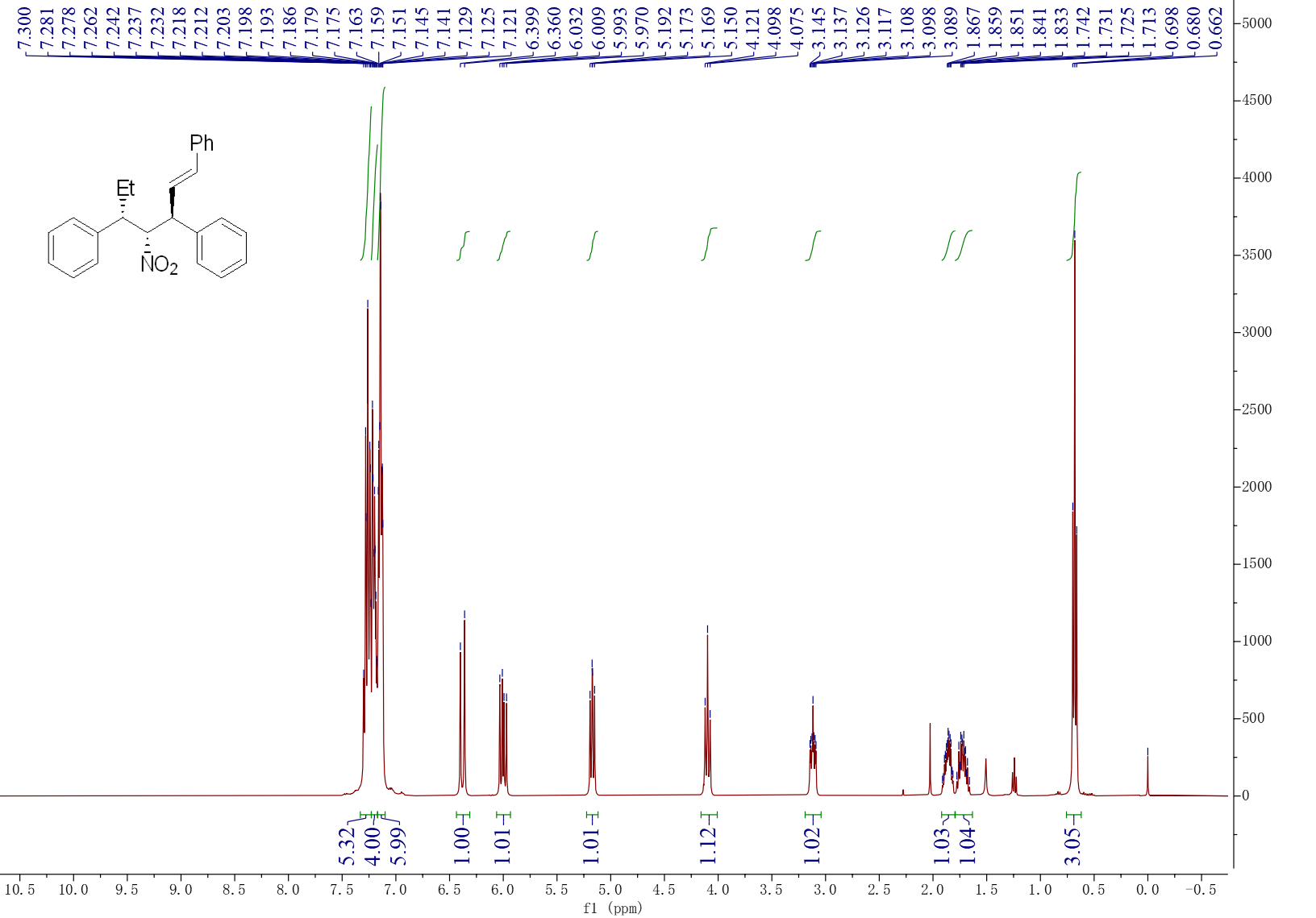


**6** ^13^C NMR


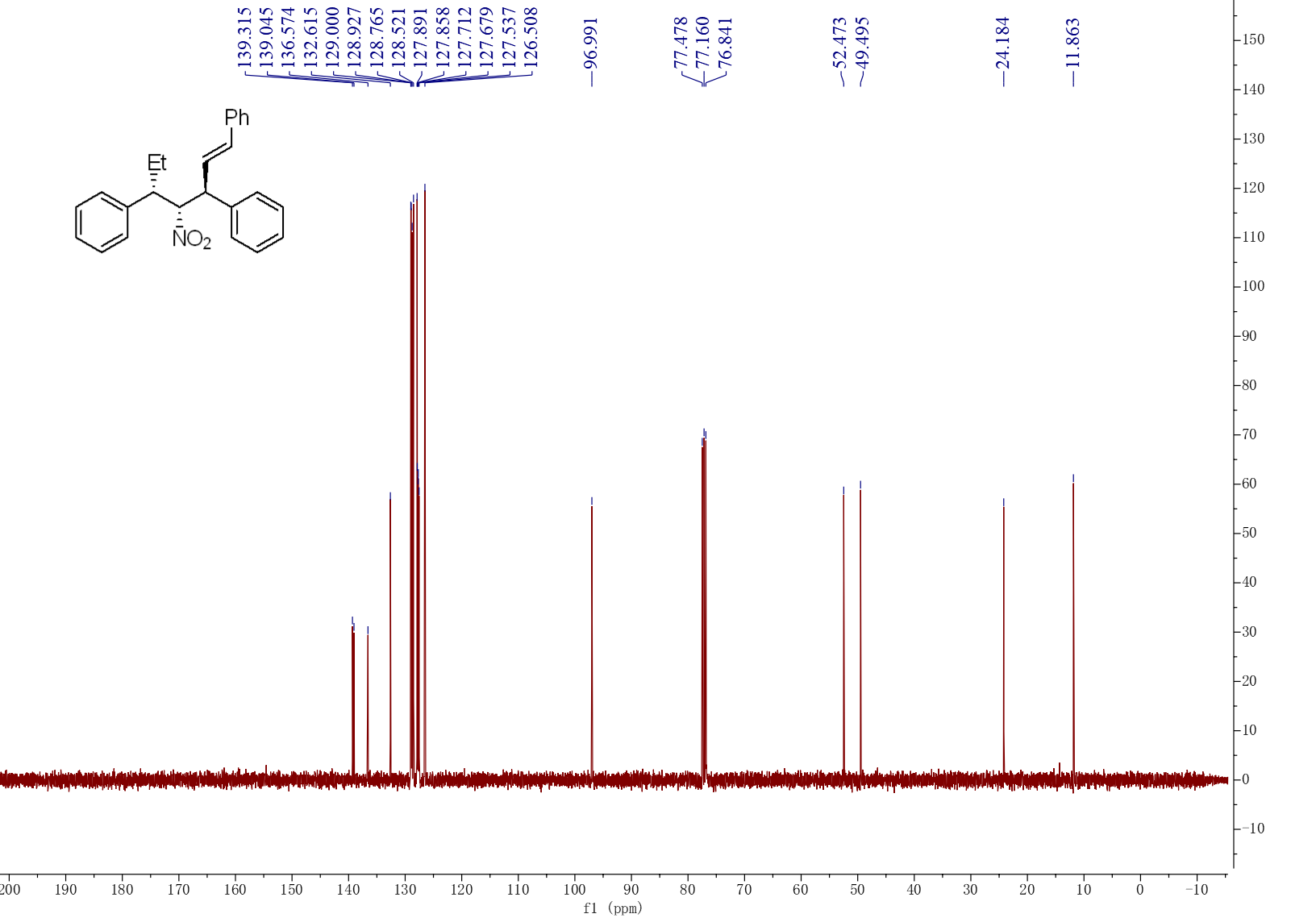


**7** ^1^H NMR


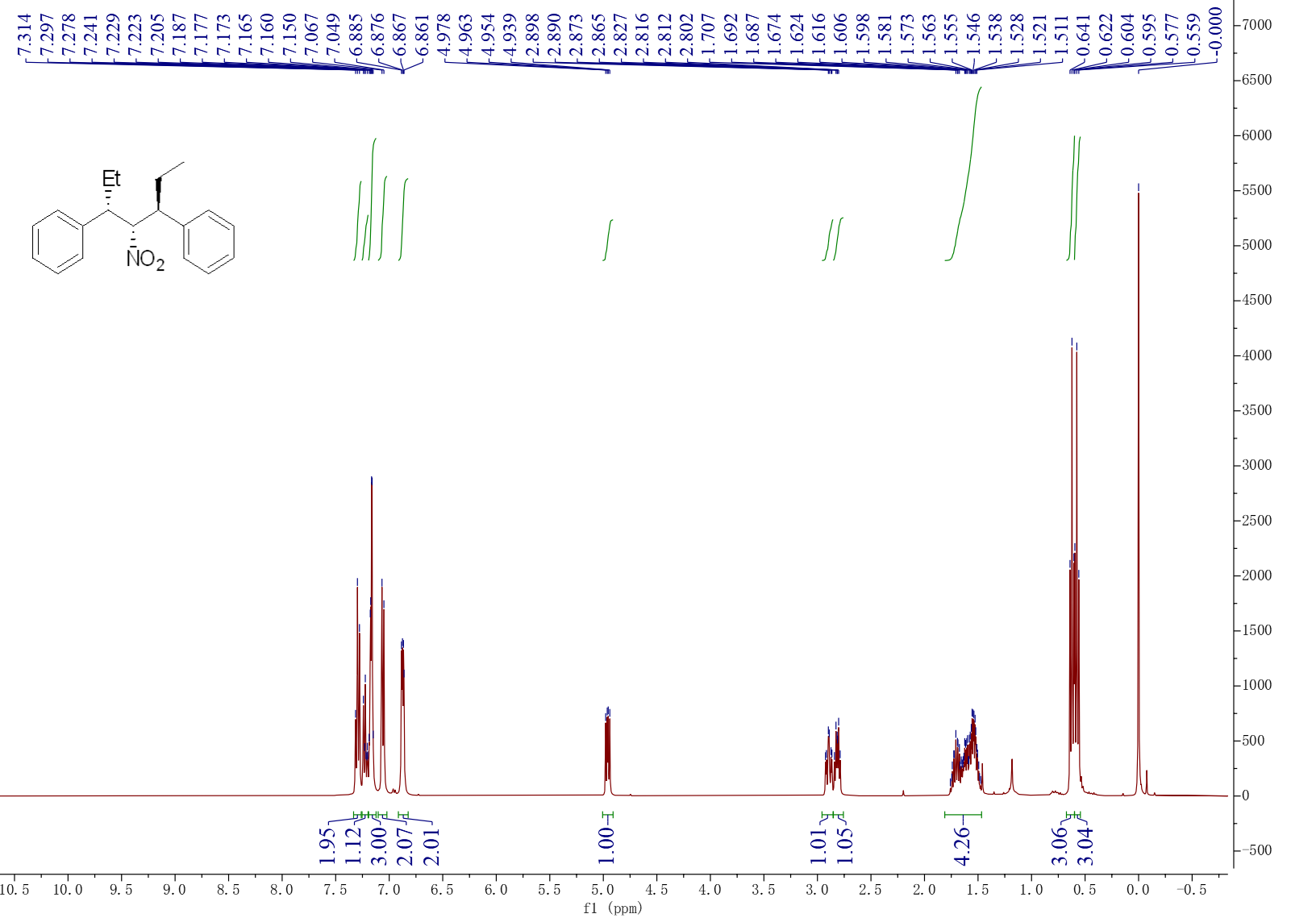


**7** ^13^C NMR


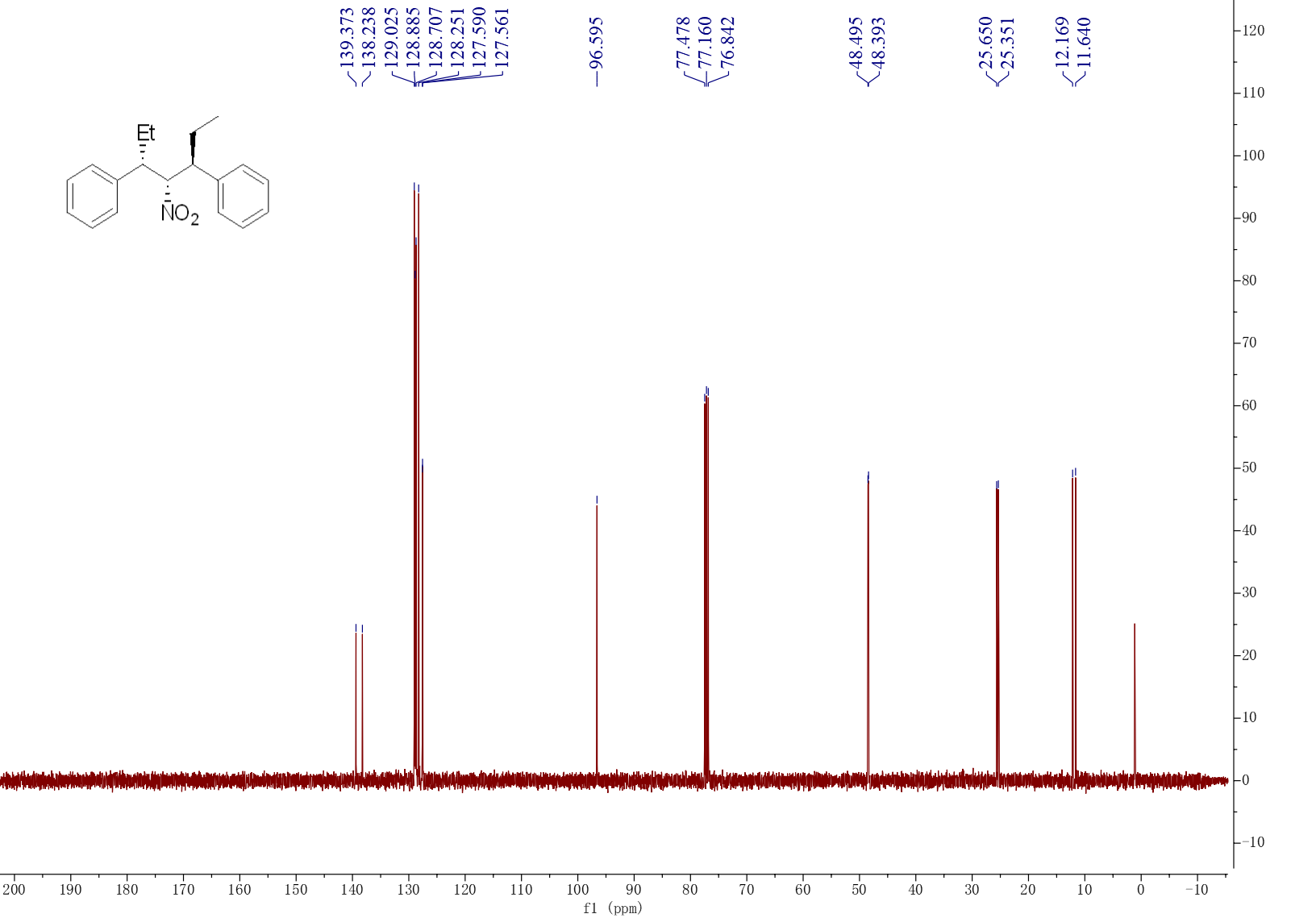


**8** ^1^H NMR


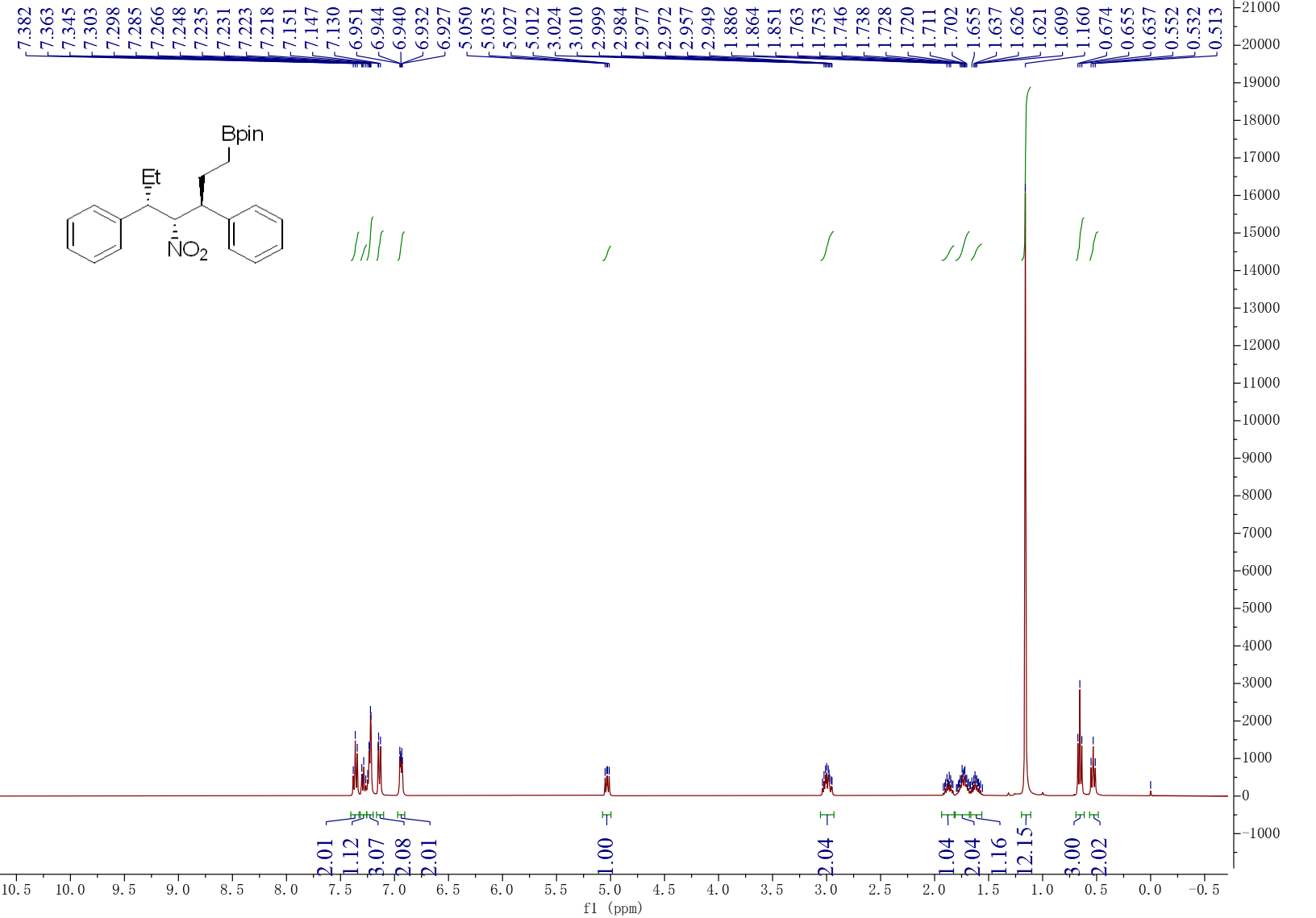


**8** ^13^C NMR


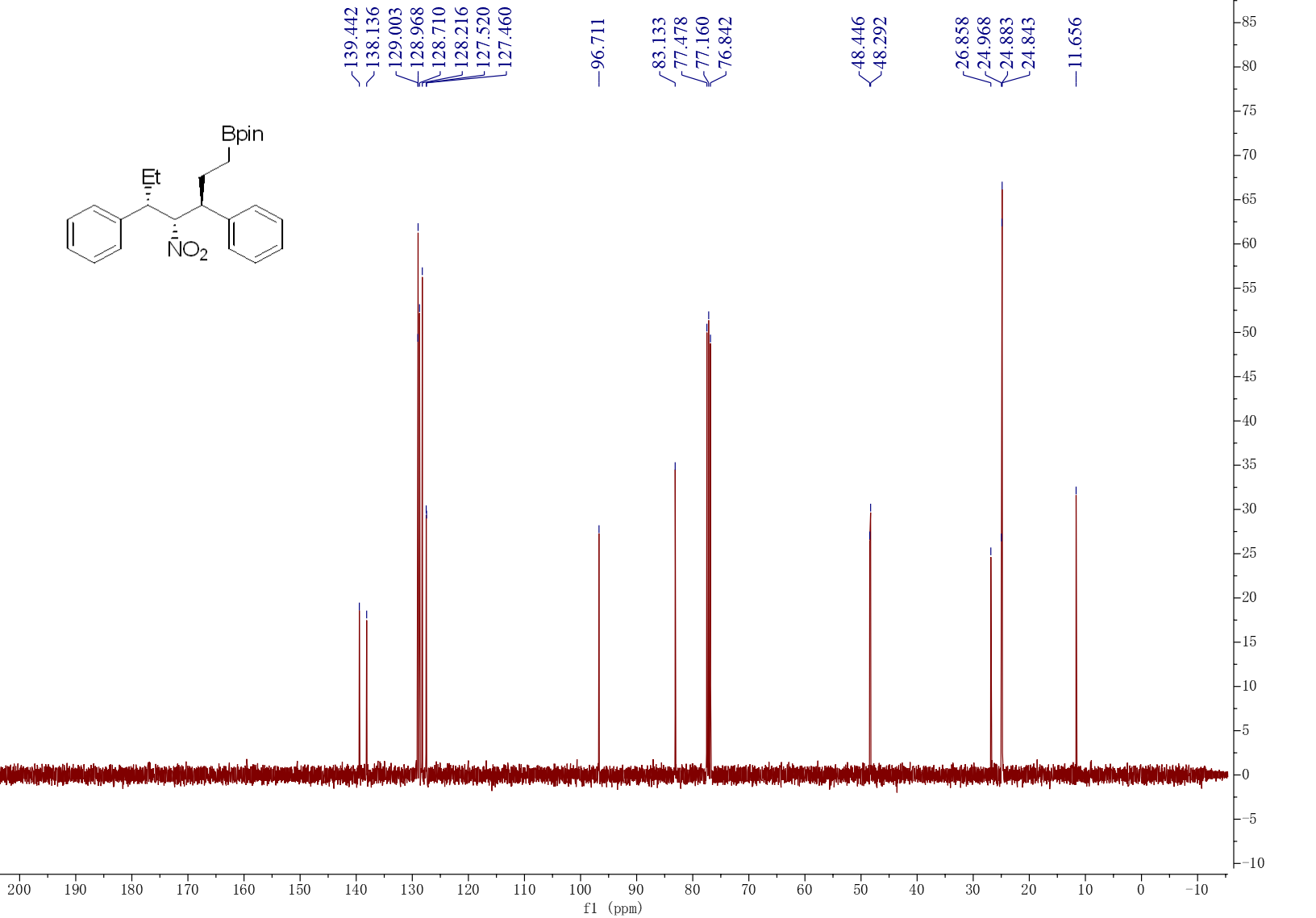


**9** ^1^H NMR


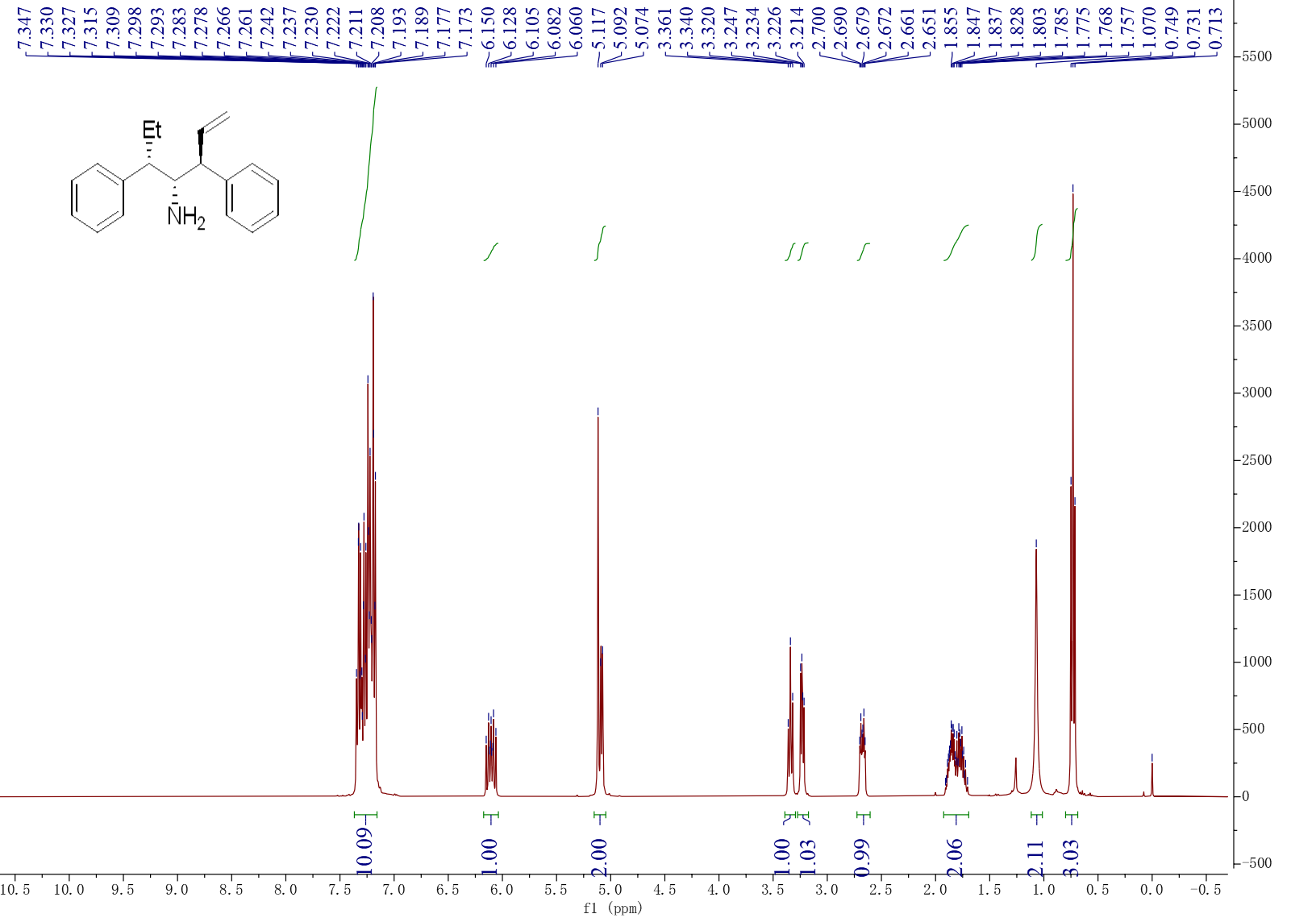


**9** ^13^C NMR


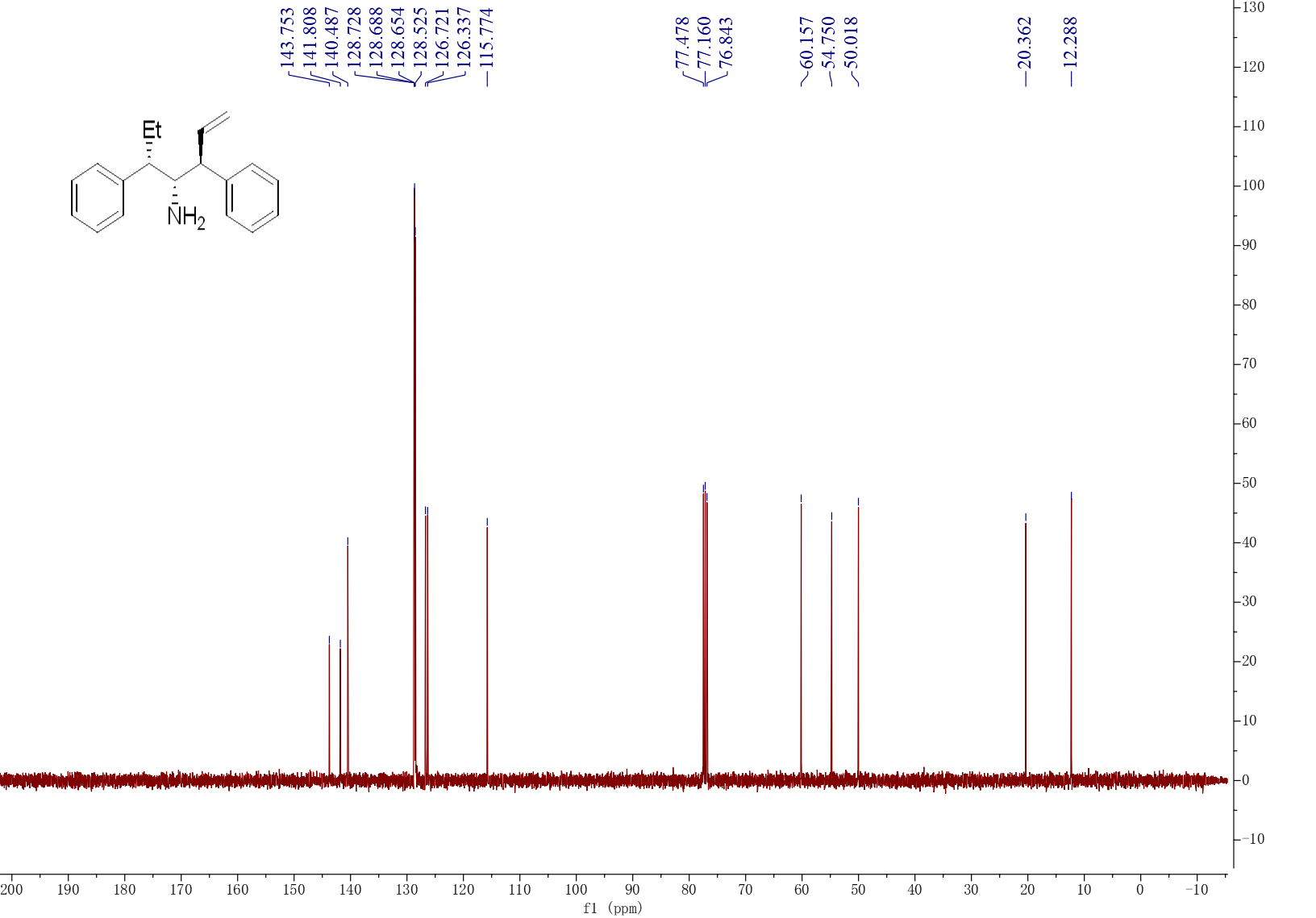


# 10. Copies of HPLC chromatograms

**4a** HPLC

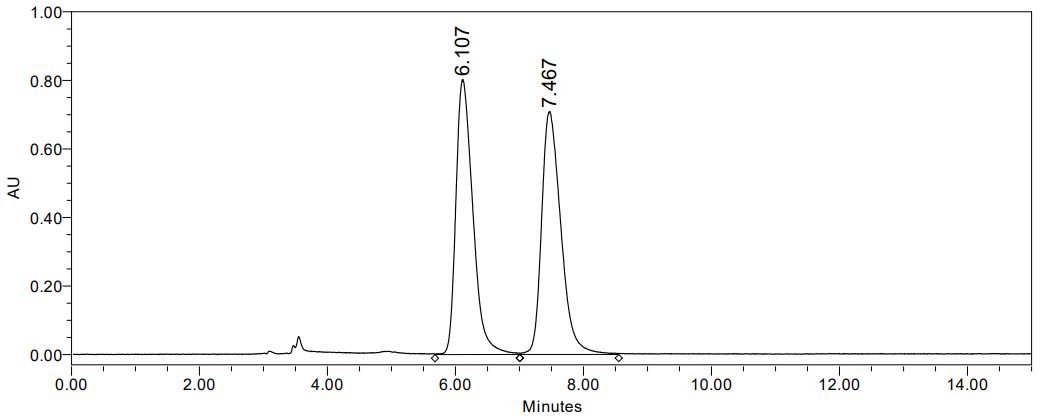


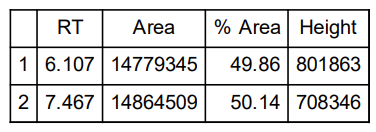


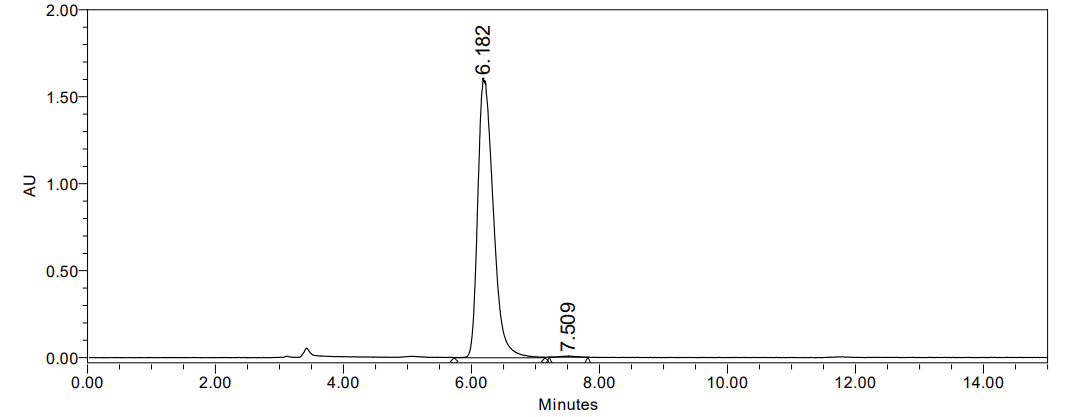


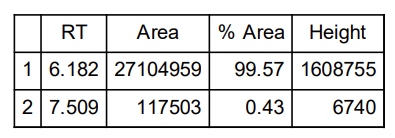


**4b** HPLC

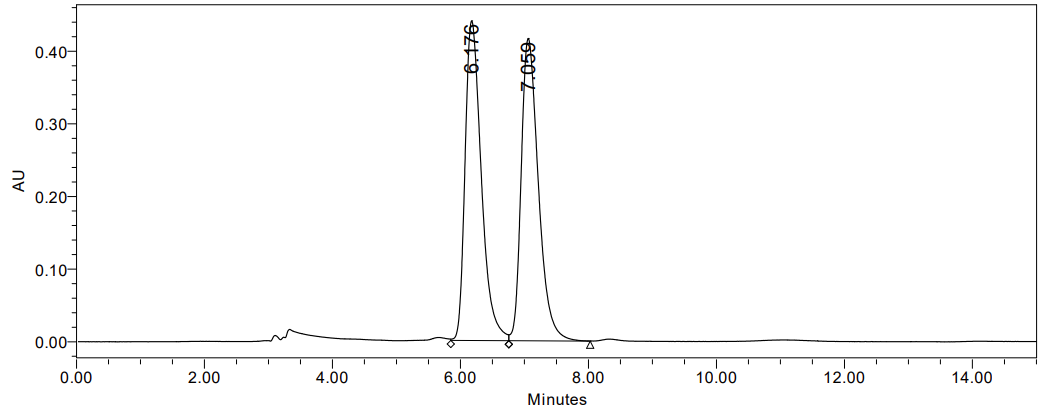


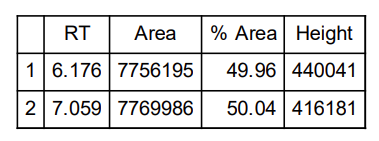


**
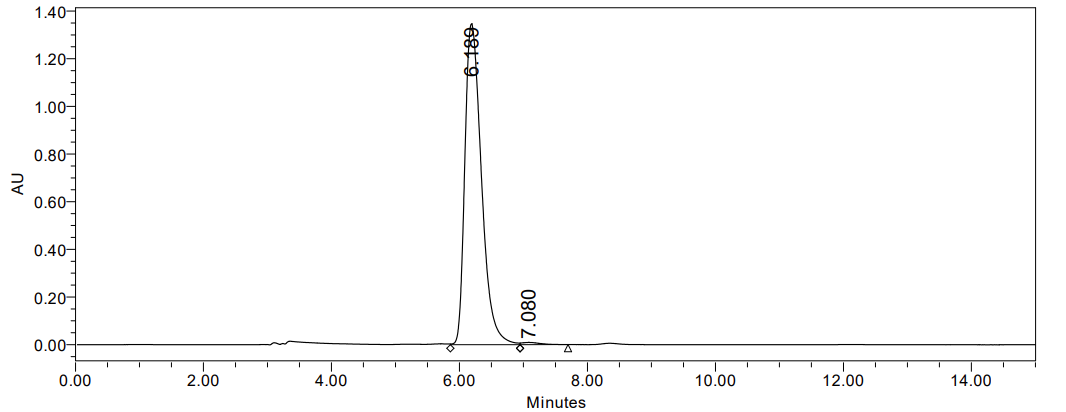
**


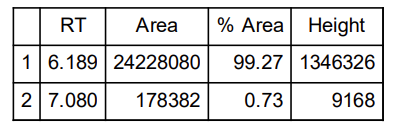


**4c** HPLC

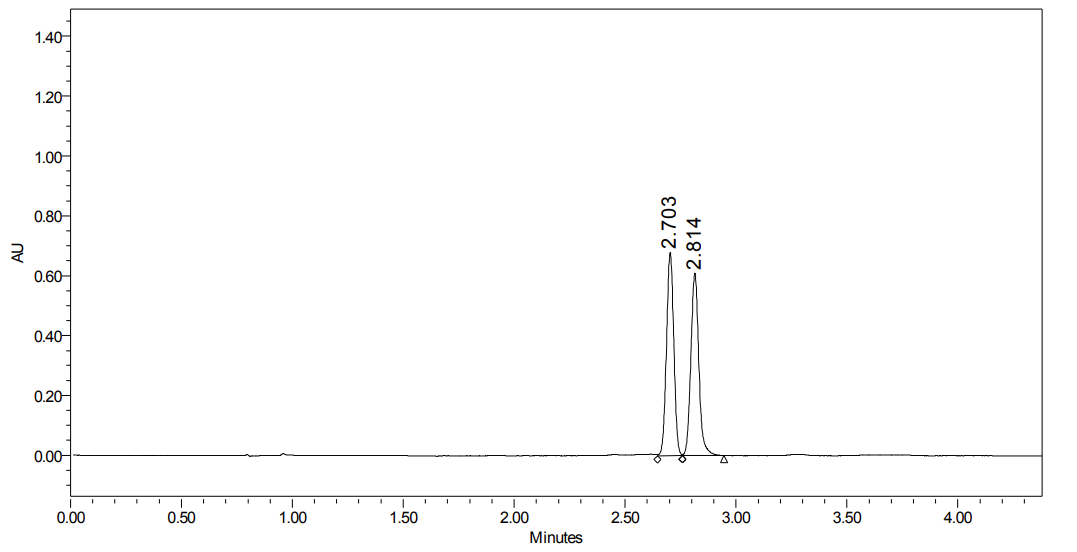

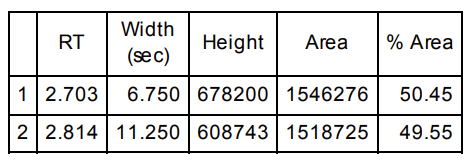


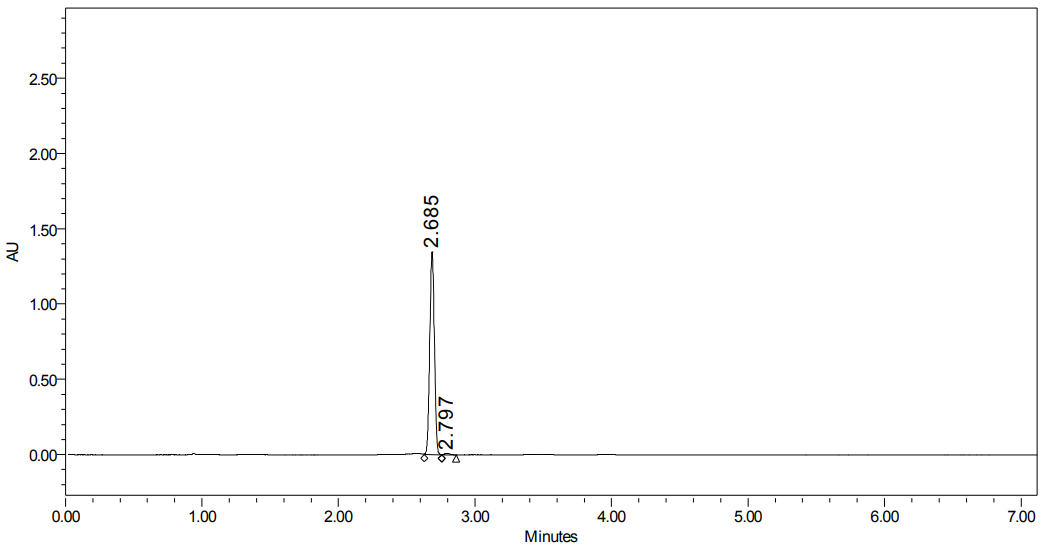


**4d** HPLC

**4e** HPLC

**4f** HPLC

**4g** HPLC

**4h** HPLC

**4i** HPLC

**4j** HPLC

**4k** HPLC

**4l** HPLC

**4m** HPLC

**4n** HPLC

**4o** HPLC

**4p** HPLC

**4q** HPLC

**4r** HPLC

**4s** HPLC

**4t** HPLC

**5a** HPLC

**5b** HPLC

**5c** HPLC

**5d** HPLC

**5e** HPLC

**5f** HPLC

**5g** HPLC

**5h** HPLC

**5i** HPLC

**5j** HPLC

**5k** HPLC

**5l** HPLC

**5m** HPLC

**5n** HPLC

(1*R*,2*S*,3*S*)-**4a′** HPLC

(1*S*,2*R*,3*R*)-**4a′** HPLC

(1*R*,2*S*,3*R*)-**4a** HPLC

**6** HPLC

**7** HPLC

**8** HPLC

**9** HPLC
